# Peracid Oxidation of Unactivated sp^3^ C−H Bonds: An Important Solvent Effect

**DOI:** 10.1002/chem.202204007

**Published:** 2023-04-24

**Authors:** Sergej Maciuk, Susanna H. Wood, Vipulkumar K. Patel, Peter D. P. Shapland, Nicholas C. O. Tomkinson

**Affiliations:** ^1^ Department Pure and Applied Chemistry Thomas Graham Building University of Strathclyde Glasgow G1 1XL UK; ^2^ GlaxoSmithKline R&D Gunnels Wood Road Stevenage SG1 2NY UK

**Keywords:** C−H Oxidation, DFT, fluorinated alcohol, peracid, oxidation

## Abstract

The peracid oxidation of hydrocarbons in chlorinated solvents is a low yielding and poorly selective process. Through a combination of DFT calculations, spectroscopic studies, and kinetic measurement it is shown that the origin of this is electronic in nature and can be influenced through the addition of hydrogen bond donors (HBD) and hydrogen bond acceptors (HBA). Performing the reaction of a cycloalkane with *m*CPBA in a fluorinated alcohol solvent such as nonafluoro‐*tert*‐butanol (NFTB) or hexafluoroisopropanol (HFIP), which act as strong HBD and poor HBA, leads to significantly higher yields and selectivities being observed for the alcohol product. Application of the optimised reaction conditions allows for the selective oxidation of both cyclic and linear alkane substrates delivering the corresponding alcohol in up to 86 % yield. The transformation shows selectivity for tertiary centres over secondary centres and the oxidation of secondary centres is strongly influenced by stereoelectronic effects. Primary centres are not oxidised by this method. A simple computational model developed to understand this transformation provides a powerful tool to reliably predict the influence of substitution and functionality on reaction outcome.

## Graphical abstract

**The solvent effect**: The peracid oxidation of unactivated alkanes in fluorinated alcohol solvents leads to the corresponding alcohol in high yield and with excellent levels of selectivity.

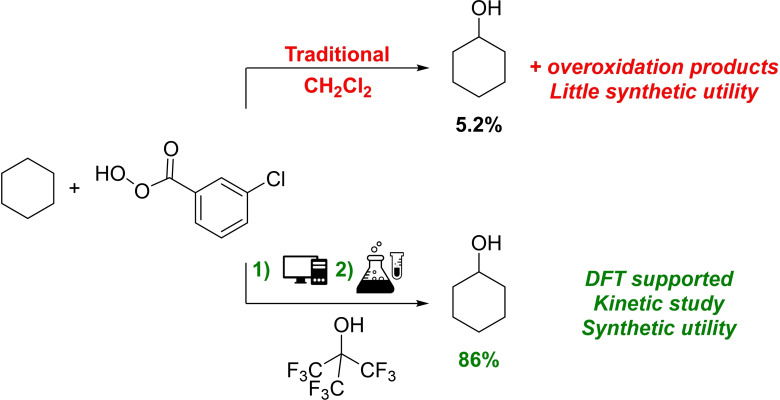

## Introduction

**Figure 1:**
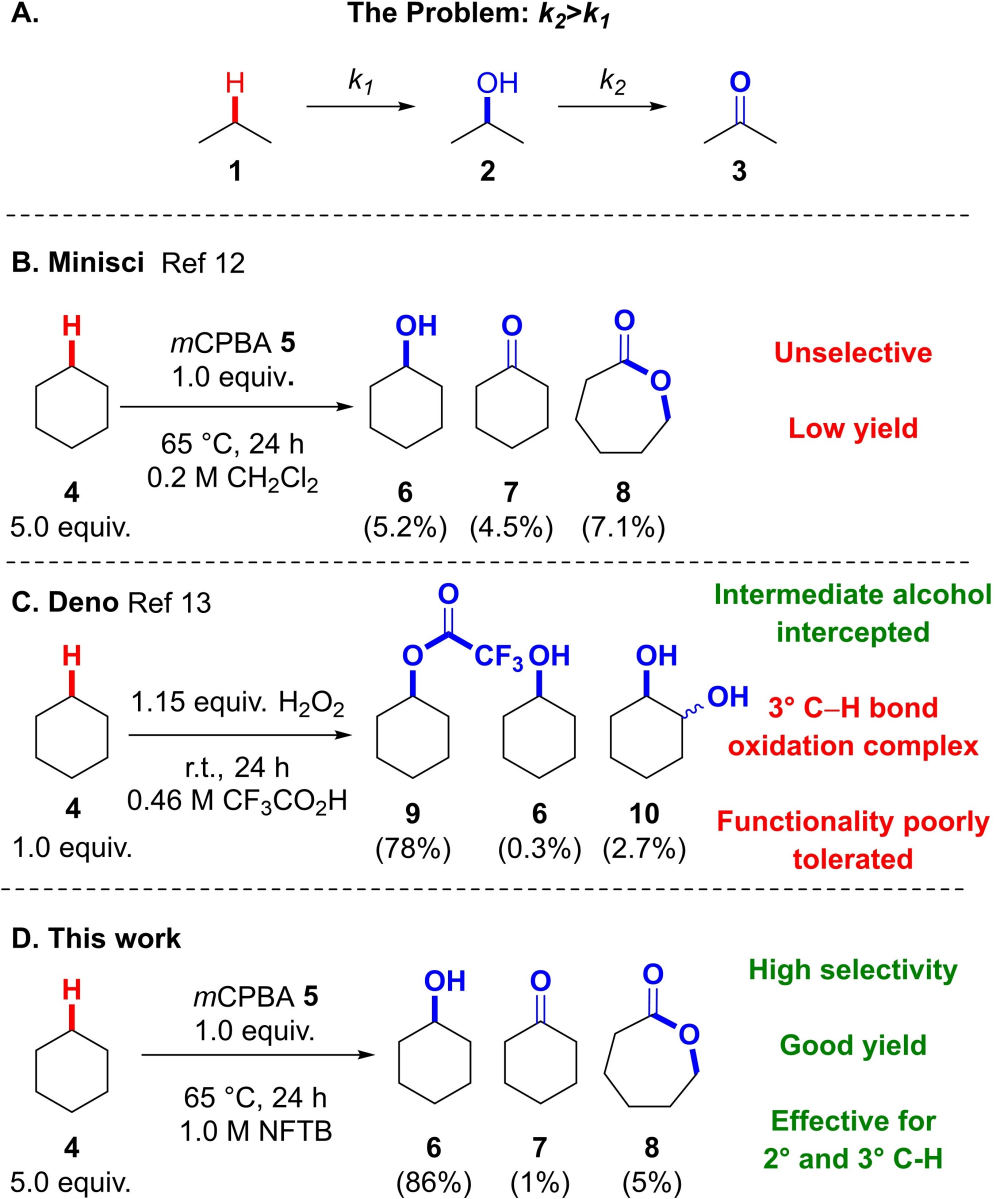
The peracid oxidation of alkanes.

Unactivated sp^3^ C−H bonds are ubiquitous within organic molecules. Their high pKa,^[1]^ bond dissociation energy^[2]^ and HOMO‐LUMO energy gap^[3]^ render them inert to the majority of chemical reactions which has resulted in the logic of chemical synthesis.^[4]^ In recent years these C−H bonds have been viewed as opportunities to create new science, revolutionising approaches to complex synthetic targets.^[5]^ Through evolutionary refinement nature has developed enzymes such as cytochrome P450^[6]^ and methane monooxygenases^[7]^ to oxidise unactivated C−H bonds. Chemical processes able to mimic these transformations have multiple applications in areas including the processing of hydrocarbon feedstocks, the oxidation of small molecules for fine chemical research and the generation of new methods for late‐stage functionalisation.^[8]^ A long‐standing obstacle in developing efficient chemical methods to oxidise unactivated C−H bonds is the fact that the products of oxidation are frequently more reactive than the parent substrate leading to overoxidation, reducing the efficiency of the overall process (Figure 1A).^[9]^ The ability to understand and prevent this overoxidation in a reliable and predictable manner would be of significant use to those working in this pioneering area of research.

Organic peracids are powerful oxidising agents which are used in a broad variety of oxidation procedures.^[10]^ Surprisingly, these reagents have received little attention in the oxidation of unactivated C−H bonds, despite their potential impact within the area.^[11]^ The challenge with using peracids as reagents to oxidise unactivated secondary C−H bonds resides in the fact that the initial alcohol product **2** is more susceptible to oxidation than the starting alkane **1** (Figure 1A, *k2*>*k1*). For example, Minisci reported a low yielding and unselective method for the oxidation of cyclohexane **4** to cyclohexanol **6** using *m*CPBA **5** as the oxidant (Figure 1B).^[12]^ An exciting discovery by Deno showed that reaction of hydrogen peroxide and a cycloalkane substrate such as cyclohexane **4** in trifluoroacetic acid led to the corresponding trifluoroacetate derivative **9**, through the in‐situ generation of trifluoroperacetic acid, oxidation to cyclohexanol **6** and subsequent esterification by solvent under the acidic reaction conditions (Figure 1C).^[13]^ This circumvented the problem of overoxidation by intercepting the cyclohexanol product **6** before it was oxidised further. Whilst this strategy was effective for unfunctionalised cycloalkanes, functionality was not tolerated and products from the oxidation of tertiary centres were unstable under the reaction conditions.[^13^, ^14^]

Fluorinated alcohols are polar molecules that are used in organic synthesis as solvents, cosolvents, additives and Brønsted acidic promotors. Due to their ability to act as strong H‐bond donors, stabilise cations, solubilise anions and facilitate the transfer of protons they have shown extraordinary effects in diverse areas of synthesis.[^15^, ^16^, ^17^, ^18^, ^19^, ^20^, ^21^, ^22^, ^23^, ^24^, ^25^, ^26^, ^27^, ^28^] Of note is the use of fluorinated alcohols in oxidation processes.[^17^, ^21^, ^23^, ^27^, ^28^] For example, Berkessel has shown that use of 1,1,1,3,3,3‐hexafluoro‐2‐propanol (HFIP) as the solvent can dramatically accelerate the hydrogen peroxide mediated epoxidation of alkenes through multiple H‐bond networks.[^17^, ^29^] Along with accelerating the rate it has been shown that fluorinated alcohol solvents can retard overoxidation in unactivated sp^3^ C−H bond oxidations^[30]^ promoted by a variety of oxidants including manganese−oxo species,^[31]^ dioxiranes,^[32]^ oxaziridinium ions,^[33]^ aminoxyl radicals,^[34]^ and alkoxy radicals.^[35]^

To overcome the problem of overoxidation in the peracid oxidation of alkanes we believed that an intimate understanding of the reaction mechanism could provide the insight necessary to develop a selective procedure. Within this publication we present a detailed analysis of the peracid oxidation of unactivated aliphatic sp^3^ C−H bonds. Through a combination of electronic structural calculations, reaction kinetics and NMR analysis we identify the origin of overoxidation as an overlooked solvent effect. Based upon this insight we show that conducting the reaction in a fluorinated alcohol retards overoxidation, providing a simple and effective solution to this longstanding challenge in synthetic chemistry.

## Results and Discussion

**Scheme 1:**
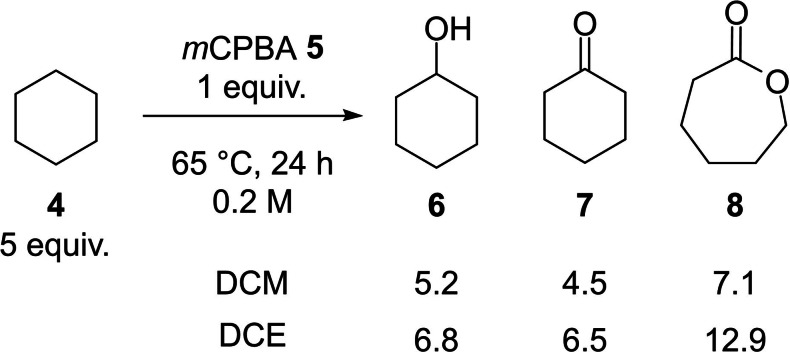
The *m*CPBA oxidation of cyclohexane reported by Minisci showing % conversion to products (results taken from Ref. [12]).

As a starting point to the investigation we revisited the *m*CPBA **5** oxidation of cyclohexane **4** previously examined by Minisci (Scheme 1).^[12]^ Performing this reaction under optimised conditions in dichloromethane (DCM) or dichloroethane (DCE) led to poor overall yields and a complex mixture of products including cyclohexanol **6**, cyclohexanone **7** and caprolactone **8**. Whilst these results embodied the net oxidation of unactivated sp^3^ C−H bonds they did not represent a synthetically tractable transformation and overoxidation clearly provided a challenge which needed to be addressed. In order to understand the competitive processes involved we elected to develop a computational model for the conversion whilst also considering important, unexplained and overlooked observations in the literature concerning the peracid oxidation of alkanes.

**Scheme 2:**
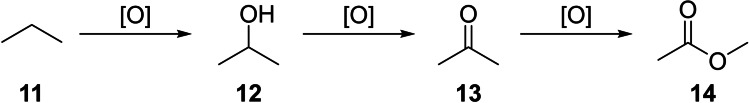
The oxidation of propane **11** to methyl acetate **14**.

**Figure 2:**
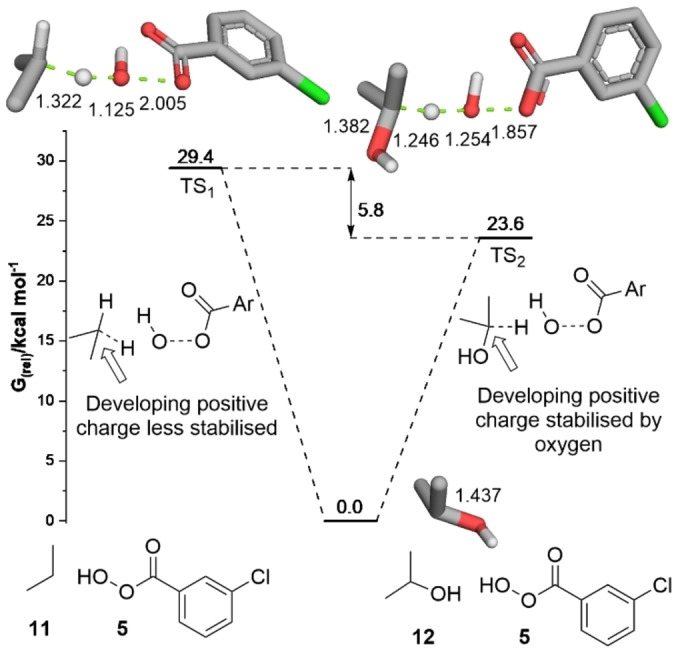
Calculated transition state barriers for the *m*CPBA oxidation of propane **11** and isopropanol **12** in the gas phase at B3LYP‐GD3/6‐311++G(d,p) level of theory.

The oxidation of propane **11** with a peracid is a three‐step process. First, the conversion of the parent alkane **11** leads to the secondary alcohol **12**, which is followed by a second oxidation to the ketone **13** and subsequent Baeyer‐Villiger reaction provides the ester **14** (Scheme 2). Gas phase DFT modelling (B3LYP‐GD3/6‐311++G(d,p)) of the reaction between *m*CPBA **5** and propane **11** to give isopropanol **12** revealed a transition state energy that was 5.8 kcal mol^−1^ higher in energy than the transition state found for the conversion of isopropanol **12** to acetone **13** (Figure 2).^[36]^ Comparison of the C−O bond length in isopropanol **12** (Figure 2) to the bond length in the oxidation transition state indicates a shortening of the C−O distance from 1.437 Å in **12** to 1.382 Å in the transition state TS_2_, consistent with stabilisation of a developing positive charge by the oxygen atom. This effect was thought to be key to the lower energy barrier for the second oxidation. We postulated that disruption of this stabilising interaction by a hydrogen bond donor (HBD) could promote the selective oxidation of alkanes to the corresponding alcohol.

It has been reported that the peracid oxidation of alkanes is inhibited by the presence of hydrogen bond acceptor (HBA) solvents such as THF, dioxane, *tert*‐butanol and ethyl acetate, although the origins of this inhibition has not been discussed.[^12^, ^37^] Whilst these HBA solvents are unable to efficiently interact with cyclohexane, we postulated that the reduced activity was the result of a hydrogen bonding interaction between the solvent and *m*CPBA **5**.^[38]^ Based upon these combined observations we hypothesised that conditions for the selective oxidation of alkanes to the corresponding alcohol which suppressed subsequent oxidation to the ketone could be identified through judicious solvent selection. We speculated that a solvent which could act as an effective hydrogen bond donor (HBD) would interact with the lone pair of electrons on the alcohol product (e.g. isopropanol **12**) disfavoring overoxidation to the ketone (e.g. acetone **13**). It was also important the solvent did not function as an efficient HBA that was known to slow down the overall transformation.^[37]^ We believed we could achieve these criteria by conducting the reaction in a fluorinated alcohol medium.

**Scheme 3:**
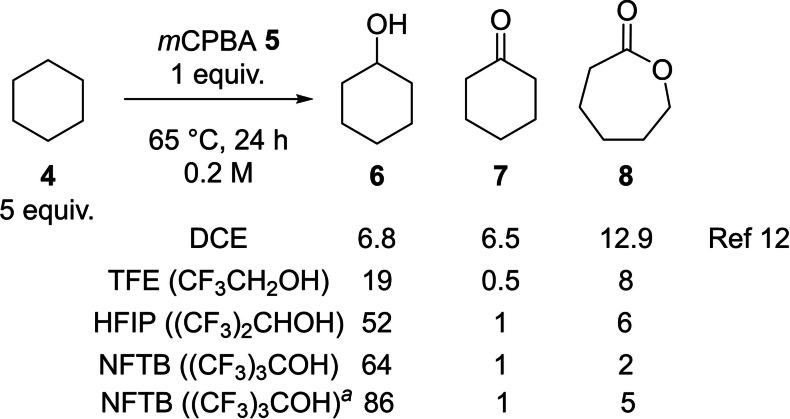
Oxidation of cyclohexane **4** in fluorinated alcohol solvents showing % conversion to products. ^*a*^ Reaction conducted at 1.0 M concentration.

We examined the *m*CPBA **5** oxidation of cyclohexane **4** in three common fluorinated alcoholic solvents: trifluoroethanol (TFE), HFIP and NFTB.[^15^, ^16^, ^17^, ^18^, ^19^, ^20^, ^21^, ^22^, ^23^, ^24^, ^25^, ^26^, ^27^, ^28^] The results obtained under standard reaction conditions reported by Minisci (0.2 M, 65 °C, 24 h) are outlined in Scheme 3.^[12]^ Whilst a marginal improvement in selectivity was seen with TFE, the use of the strong HBD solvents HFIP and NFTB showed an outstanding improvement in both yield and selectivity, with NFTB delivering the cyclohexanol product **6** in 64 % yield along with minor quantities of the over‐oxidised products cyclohexanone **7** (1 %) and caprolactone **8** (2 %). We believe this remarkable change was brought about by the strong HBD ability of the fluorinated alcohol binding to the oxygen lone pair of cyclohexanol **6**, raising the transition state energy for the subsequent oxidation of the alcohol to the ketone **7**. The effect of fluorinated alcohols in a number of transformations has been described and includes remarkable influences of the solvent on aliphatic C−H functionalisations including metal‐free and transition metal catalyzed processes.[^21^, ^23^, ^27^, ^28^] A brief optimisation of the reaction conditions showed the yield of **6** could be improved further by conducting the transformation at higher concentration (1.0 M, 86 %).

**Figure 3:**
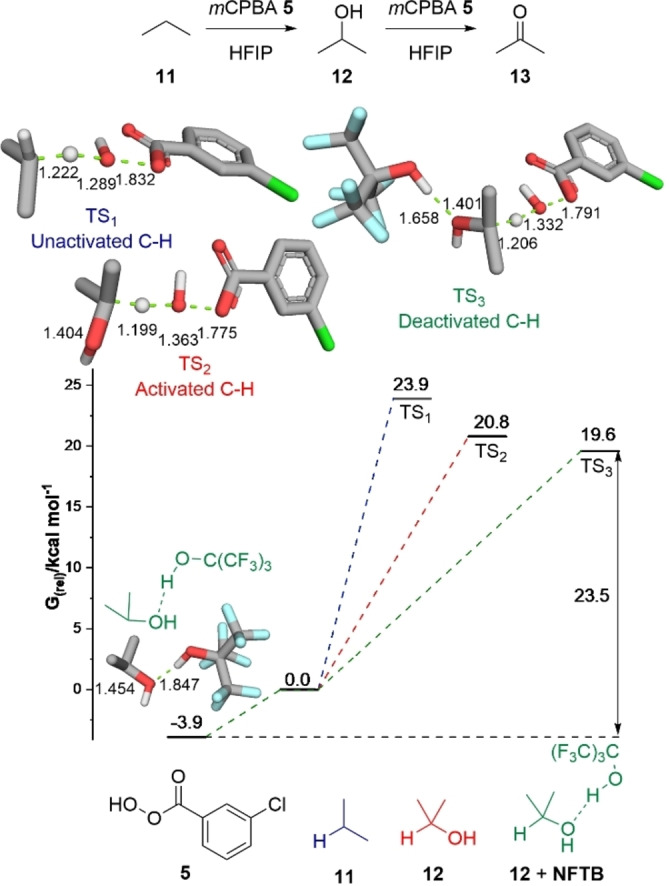
Calculated transition state energies for the oxidation of propane **11** and isopropanol **12** by *m*CPBA **5** in the presence and absence of NFTB. SMD(HFIP)‐B3LYP‐GD3 6‐311++G(d,p).

To obtain computational support for our observations we compared the oxidation of isopropanol **12** by *m*CPBA **5** in the presence and absence of a molecule of NFTB (Figure 3) using an implicit solvation model for HFIP.^[39]^ Addition of HFIP solvation reduces the barriers to oxidation for both propane **11** and isopropanol **12**, however, the reduction for isopropanol TS_2_ is more modest, resulting in a smaller gap of 3.1 kcal mol^−1^ between TS_1_ and TS_2_. This finding is consistent with deactivation of the C−H bond isopropanol in a HBD solvent. The calculations also revealed that NFTB can form a H‐bonded complex with isopropanol **12** that is 3.9 kcal mol^−1^ lower in energy than free NFTB and isopropanol. Calculation of the barrier to oxidation of this H‐bonded complex (TS_3_) predicted a slight reduction in the barrier relative to free isopropanol **12** (19.6 kcal mol^−1^) but an overall barrier of 23.5 kcal mol^−1^. Using an implicit solvent model the calculated C−O bond length increased in both TS_2_ and TS_3_ (1.404 Å and 1.401 Å respectively) relative to the gas phase calculation (1.382 Å), consistent with a reduction in the ability of the oxygen atom to stabilise a nascent positive charge. Whilst these predicted transition states are marginally lower in energy than that for the oxidation of the unactivated C−H bond in propane (23.9 kcal mol^−1^) it should be noted that in the oxidation of cyclohexane **4** (Scheme 3), 5 equivalents of the alkane are present within the reaction mixture and there are 12 oxidisable C−H bonds in the substrate, whereas cyclohexanol **6** is present at much lower concentrations and only contains a single oxidisable C−H bond, reinforcing the high selectivity observed.^[40]^ This provided a functional computational model to readily evaluate substrates within the oxidation process.

To obtain experimental evidence for a H‐bonding interaction between NFTB and the reaction components involved in the selective oxidation we undertook a series of DOSY NMR experiments to determine any change in diffusion coefficient (*D*) in the presence and absence of NFTB.^[41]^ The values obtained were compared to the diffusion coefficient of tetramethylsilane (*D*_TMS_), an inert hydrophobic compound that would have limited interaction with NFTB, allowing us to account for any change in solution viscosity. The diffusion coefficient of *m*CPBA **5** in CDCl_3_ remained constant upon the addition of 1, 2 or 3 equivalents of NFTB and the diffusion coefficient ratio compared to TMS (*D*/*D*_TMS_) also remained steady (0.82–0.83×10^−9^ m^2^ s^−1^) (Table 1). This suggested there was no strong interaction between the *m*CPBA **5** and the NFTB. As expected, this was also the case with cyclohexane **4** which showed similar diffusion coefficients in both the presence and absence of the fluorinated alcohol (2.37–2.39×10^−9^ m^2^ s^−1^). In stark contrast, the addition of NFTB to a CDCl_3_ solution of cyclohexanol brought about a significant decrease in the diffusion coefficient (*D*) of the alcohol, which reduced from 2.06×10^−9^ m^2^ s^−1^ (0 equivalents NFTB) to 1.77×10^‐9^ m^2^ s^−1^ (1 equivalent NFTB) and reduced further upon the addition of more NFTB (1.59×10^−9^ m^2^ s^−1^ 2 equivalents NFTB; 1.52×10^−9^ m^2^ s^−1^ 3 equivalents NFTB). This change is consistent with a strong H‐bond between the fluorinated alcohol and cyclohexanol **6**. Evidence that this reduction is not due to a change in viscosity of the solution comes from the diffusion coefficient ratio which also decreases upon the addition of NFTB to a mixture of cyclohexanol and TMS (*D*_**6**_/*D*_TMS_). Schneider reported that the addition of hydrogen bond acceptors such as tetrahydrofuran (THF), 1,4‐dioxane or ethyl acetate inhibited the peracid oxidation of alkanes, with THF having the greatest influence.^[37]^ Conducting a DOSY experiment with THF showed *D*_THF_=2.50×10^−9^ m^2^ s^−1^ and *D*_THF_/*D*_TMS_=1.11 (see Supporting Information). Upon the addition of *m*CPBA **5** D_THF_ reduced to 2.39×10^−9^ m^2^ s^−1^ showing an interaction between these two components. This provides evidence that there is a H‐bonding interaction between THF and the peracid which could reduce the rate of alkane oxidation. Interestingly, upon the addition of NFTB, *D*_THF_ reduced significantly (2.39→1.70×10^−9^ m^2^ s^−1^). This outcome indicates that NFTB can form a strong hydrogen bond with THF, disrupting the H‐bonding interaction between *m*CPBA **5** and THF, leading to the reduced diffusion coefficient observed.

**Table 1:** Diffusion coefficients determined through DOSY experiments.

Analyte mixture	Diffusion coefficient (*D*) (equiv. NFTB), 10^−9^ m^2^ s^−1^				Diffusion coefficient ratio (equiv. NFTB) (*D*/*D*_TMS_)			
	0	1	2	3	0	1	2	3
*m*CPBA TMS	1.85 2.24	1.85 2.24	1.85 2.24	1.84 2.25	0.83	0.83	0.83	0.82
Cyclohexane TMS	2.38 2.26	2.37 2.28	2.37 2.26	2.39 2.27	1.05	1.04	1.05	1.05
Cyclohexanol TMS	2.06 2.26	1.77 2.26	1.59 2.25	1.52 2.25	0.91	0.78	0.71	0.68
THF (1 equiv) *m*CPBA TMS	2.39 1.83 2.24	1.98 1.83 2.24	1.76 1.84 2.23	1.70 1.83 2.25	1.07 0.82	0.88 0.82	0.79 0.83	0.76 0.82

**Scheme 4:**
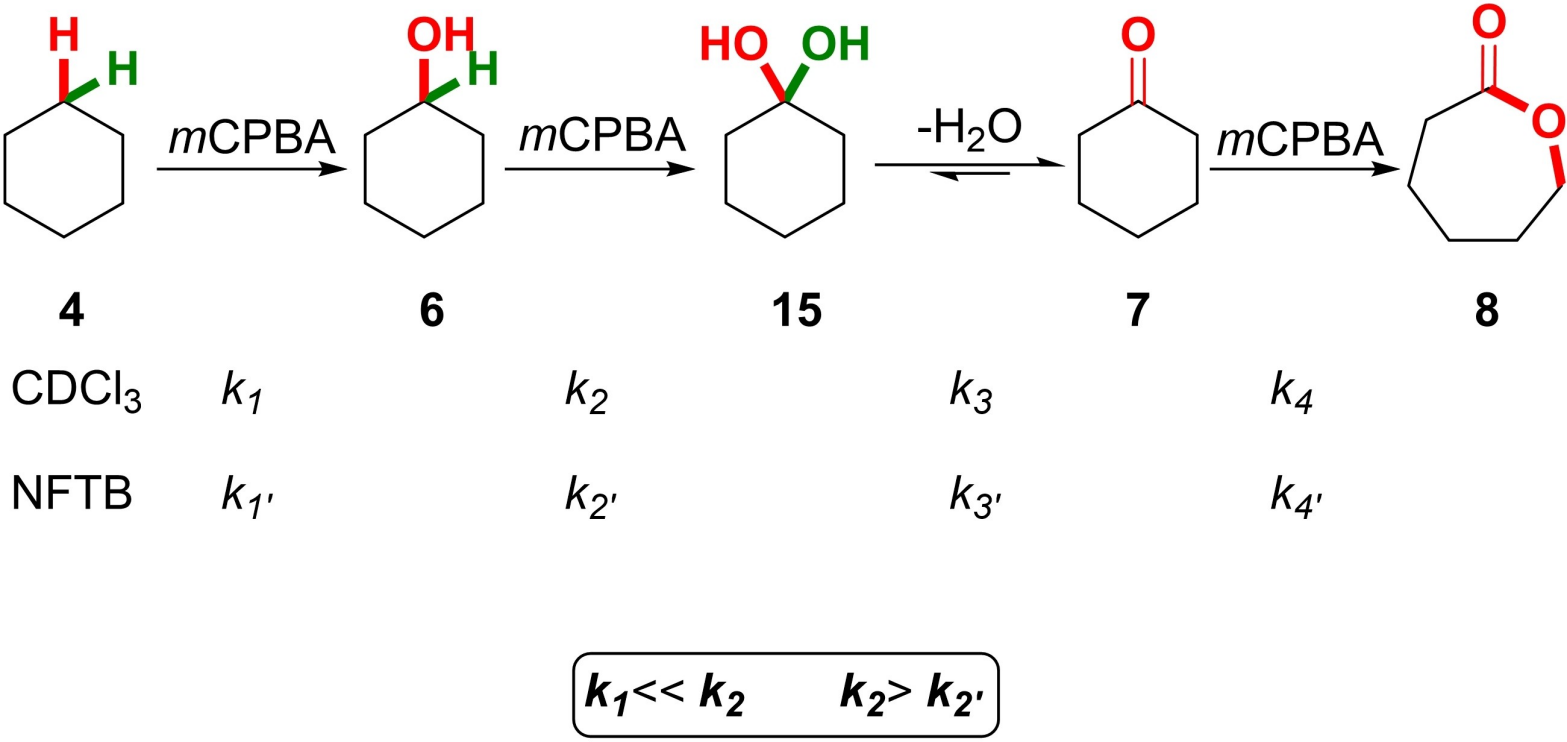
Relative rates of conversion in the oxidation of cyclohexane **4**, cyclohexanol **5** and cyclohexanone **6** with *m*CPBA **5**.

To gain support for the solvent effect hypothesis, the reaction was analysed using ^1^H NMR spectroscopy in a stepwise manner, observing qualitative conversion of the reactants over time in both CDCl_3_ and NFTB (Scheme 4). It was found that the rate of oxidation of cyclohexanol **6** with *m*CPBA **5** was greater in CDCl_3_ when compared to a reaction conducted in NFTB: *k_2_* >*k*_*2*′_. The rate of oxidation of cyclohexane **4** was determined quantitatively in NFTB whereas measuring the rate of oxidation of cyclohexane **4** in CDCl_3_ posed a significant challenge due to the large difference between barriers for Δ*G* ^≠^ of the first and subsequent oxidations. It is this change in relative rates *k_2_* and *k*_*2’*_ that accounts for the remarkable change in reaction selectivity observed when modifying the reaction medium. In addition, any solvent effect on the rapid oxidation of cyclohexanone **7** to caprolactone **8** (*k_4_* and *k*_*4*′_) was not important to the overall outcome of the process.

In line with Schneider's findings, the oxidation of cyclohexane **4** and cyclohexanol **6** were determined to be first order in *m*CPBA **5**.^[37]^ An Eyring plot for the oxidation of cyclohexanol **6** with *m*CPBA **5** in CDCl_3_ and NFTB was constructed, allowing quantification of Δ*G* ^≠^_298_ (Table 2) (See Supporting Information for full details). In chloroform, a good correlation of Δ*G* ^≠^ for the calculated value and those determined experimentally was observed whilst HFIP solvation resulted in a good estimation of the experimental difference between cyclohexane **4** and cyclohexanol **6** oxidations in NFTB (ΔΔ*G* ^≠^_298_ 0.6 kcal mol^−1^ vs. 1.4 kcal mol^−1^ from DFT calculations), providing confidence in the model developed. Comparison of the experimentally obtained values for this oxidation in CDCl_3_ and NFTB showed the difference between the transition state energies ΔΔ*G* ^≠^_298_=2.5 kcal mol^−1^. This difference in energy is mainly due to the enthalpic component Δ*H* ^≠^ being higher in NFTB (22.6 kcal mol^−1^) when compared to CDCl_3_ (16.3 kcal mol^−1^). This provides further experimental support for our proposal that destabilisation of the transition state for the conversion of cyclohexanol **6** to cyclohexanone **7** is the origin of the change in selectivity in NFTB.

**Table 2:** Experimental and calculated transition state energies for the oxidation of cyclohexane and cyclohexanol in CDCl_3_ and NFTB.

	Δ*G* ^≠^_298_/kcal mol^−1^	Δ*H* ^≠^/kcal mol^−1^	Δ*S* ^≠^/cal mol^−1^ K^−1^
Calc^[a]^ CyH CHCl_3_	26.3	15.3	−37
Calc^[a]^ CyOH CHCl_3_	22.4	11.1	−38
Calc^[a]^ CyH HFIP	21.8	11.7	−34
Calc^[a]^ CyOH HFIP	20.4	8.1	−41
Exp CyH NFTB	26.4	13.5	−43
Exp CyOH CDCl_3_	23.3	16.3	−24
Exp CyOH NFTB	25.8	22.6	−11

[a] SMD(solvent)‐B3LYP‐GD3 6‐311++G(d,p).

**Scheme 5:**
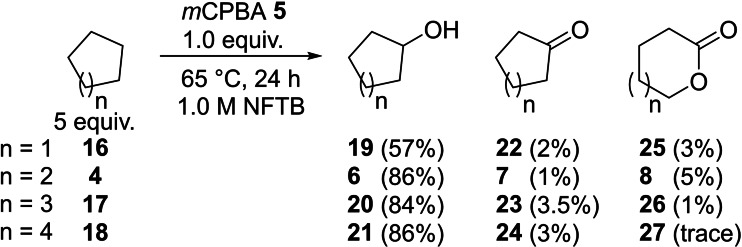
Oxidation of cycloalkanes with *m*CPBA **5** in NFTB.

Having established optimal conditions for the formation of cyclohexanol (86 % **6**, Scheme 3) we went on to examine if similar yields and selectivities were observed with alternative cycloalkane substrates (Scheme 5). Under the conditions used for cyclohexane (alkane 5 equiv., *m*CPBA **5** 1 equiv., 1.0 M in NFTB, 65 °C, 24 h), cyclopentane **16** (57 %), cycloheptane **17** (84 %), and cyclooctane **18** (86 %) consistently showed the outstanding selectivity and high yields observed for the oxidation of cyclohexane **4** suggesting this should be a general process for cyclic aliphatic substrates. It is expected that the lower yield obtained for the oxidation of cyclopentane **16** could be overcome through optimisation of the reaction conditions.

**Scheme 6:**
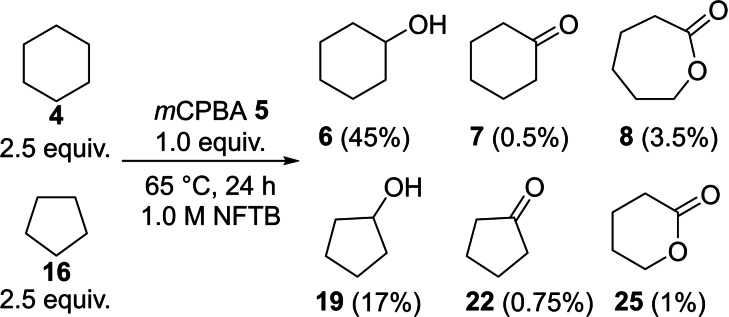
Competitive oxidation of cyclohexane **4** and cyclopentane **17** in NFTB by *m*CPBA **5**.

Conducting a competition experiment between equimolar amounts of cyclopentane **16** (2.5 equiv.) and cyclohexane **4** (2.5 equiv.) in the presence of *m*CPBA **5** (1.0 equiv.) in NFTB showed a clear preference for the oxidation of cyclohexane **4** (Scheme 6). From this experiment the difference between the transition state energies for the two transformations was determined as ΔΔ*G* ^≠^=0.65 kcal mol^−1^.

**Scheme 7:**
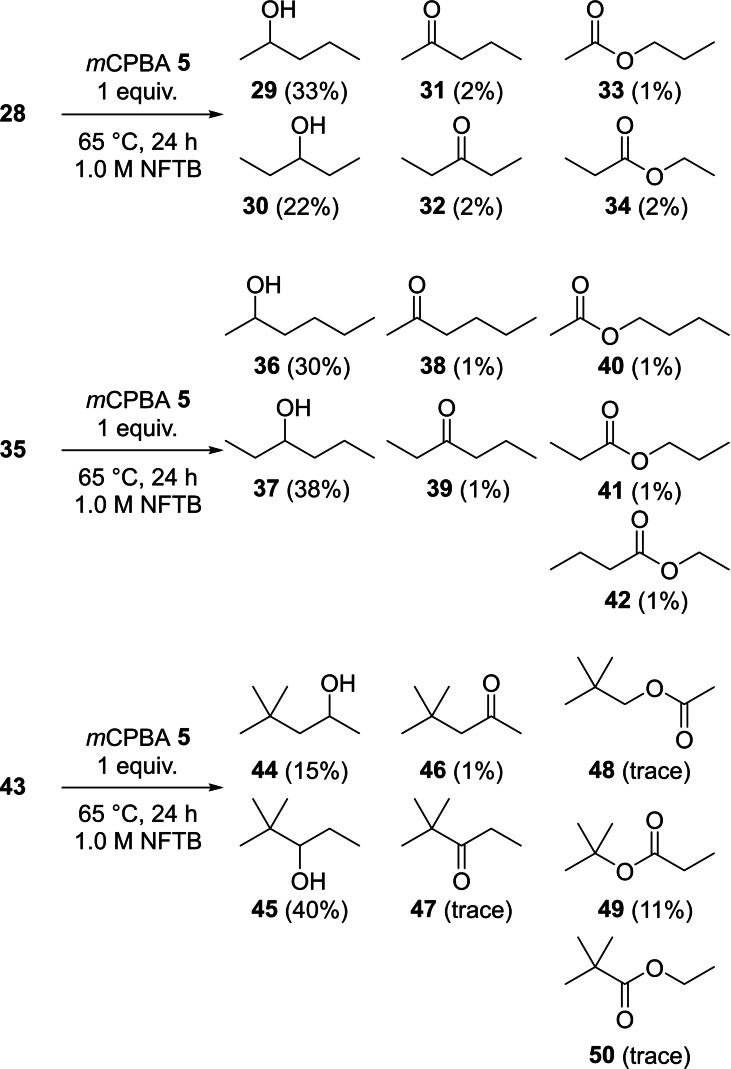
Selective *m*CPBA **5** oxidation of linear alkanes **28**, **35** and **43** in NFTB.

To explore the transformation further the oxidation of acyclic alkanes with *m*CPBA **5** was initially examined using *n*‐pentane **28** and *n*‐hexane **35** as substrates (Scheme 7). Using NFTB as the reaction solvent both of these substrates showed a high selectivity for oxidation to the secondary alcohol with only minor amounts of the overoxidation products **31–34** and **38–42** observed (≤2 %). In contrast to cyclic alkane substrates, where each C−H bond was in the same environment, different oxidisable secondary C−H bonds were present in both **28** and **35**. With *n*‐pentane **28**, preferential oxidation at C‐2 (35 %) was observed over C‐3 (21 %). This can be explained by considering the relative ratio of C−H bonds available for oxidation favouring reaction at C‐2 over C‐3, in combination with the additional stabilisation provided by hyperconjugation favoring reaction at C‐3 over C‐2. This was reinforced through the results obtained with *n*‐hexane **35** where a slight preference for oxidation at C‐3 (38 %) was observed overoxidation at C‐2 (32 %). Further support for the distinct effect of hyperconjugation influencing the selectivity in the C−H bond oxidation process came from the reaction of 2,2‐dimethylpentane **43**, which was 2.5‐fold more selective for the product **45** (40 %) over the isomer **44** (15 %). For the ester products with each of these three substrates, migration of the more substituted centre was observed as expected with the Baeyer‐Villiger oxidation.^[42]^

**Scheme 8:**
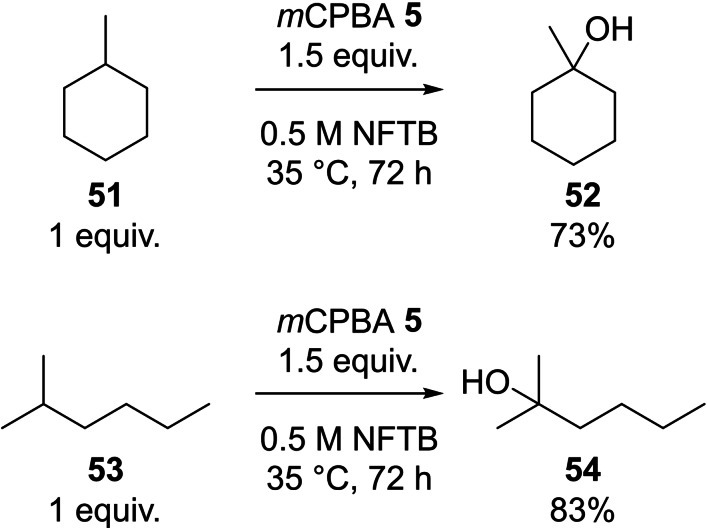
Peracid oxidation of substrates containing 3° centres.

Having established the selective *m*CPBA oxidation of secondary C−H bonds in NFTB we considered the reaction of tertiary C−H bonds. The reaction of methylcyclohexane **51** in NFTB under standard conditions (5 equiv. **51**, 1.0 M, 65 °C, 24 h) led to oxidation of both 3° and 2° centres within the substrate, however, optimisation identified conditions where the 3° centre could be oxidised selectively (1 equiv. **51**, 1.5 equiv. **5**, 0.5 M, 35 °C, 72 h), providing the product **52** in 73 %. Under identical conditions, 2‐methylhexane **53** was oxidised to the corresponding 3° alcohol **54** (83 %) (Scheme 8). To benchmark these results, we calculated the transition state barrier for the *m*CPBA oxidation of cyclohexane **4** (21.8 kcal mol^−1^) and methylcyclohexane **51** in HFIP (18.7 kcal mol^−1^) (see Supporting Information for full details) which support our experimental findings. Schneider reported extensive investigations on the selective oxidation of tertiary C−H bonds using *para*‐nitroperbenzoic acid (*p*NPBA) as the oxidant in chlorinated solvents.^[37]^ We believe our modified reaction conditions (NFTB, *m*CPBA **5**, 35 °C, 72 h) should mirror these experimental outcomes, significantly expanding the scope of this oxidation process.

**Scheme 9:**
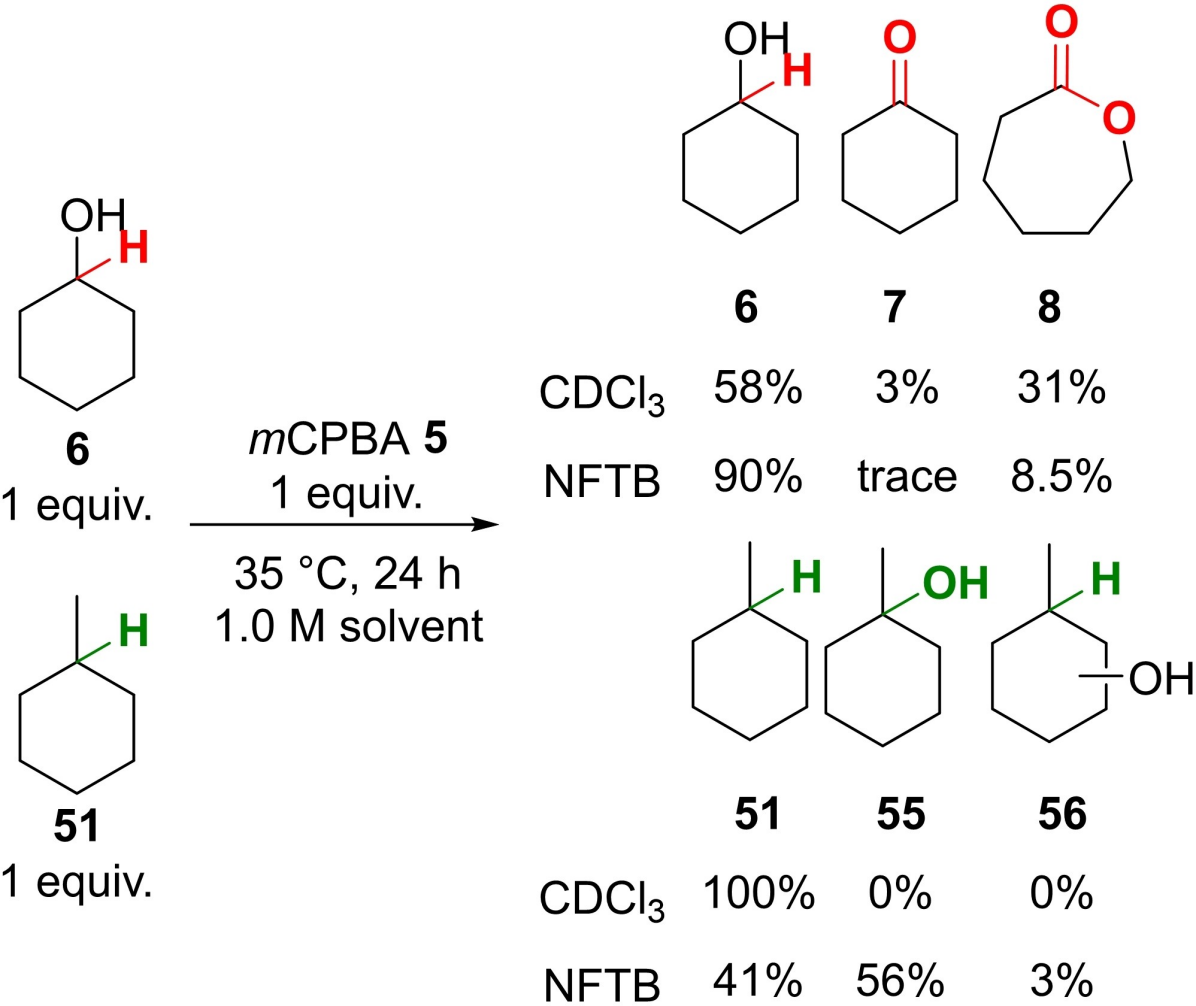
Competitive oxidation of cyclohexanol **6** and methylcyclohexane **51** in CDCl_3_ and NFTB.

Interestingly, the computational model predicted that the transition state barrier for the *m*CPBA oxidation of methylcyclohexane **51** (18.7 kcal mol^−1^) was lower than that calculated for the oxidation of cyclohexanol **6** in HFIP (20.8 kcal mol^−1^). In order to probe this experimentally we reacted methylcyclohexane **51** (1 equiv.) and cyclohexanol **6** (1 equiv.) with *m*CPBA **5** (1 equiv.) (35 °C, 24 h) in both CDCl_3_ and NFTB (Scheme 9). In CDCl_3_ the oxidation was completely selective for cyclohexanol **6** with all of the methycyclohexane **51** present in the mixture remaining unreacted after 24 h. In contrast, using NFTB as the reaction medium, selectivity for the oxidation of the tertiary centre was observed with 56 % of the methylcyclohexane being converted to the tertiary alcohol **52** after 24 h, whereas 90 % of the starting cyclohexanol **6** remained unreacted. This result was also replicated computationally, with a reversal in the order of energy barriers to oxidation for methylcyclohexane **51** (24.3 kcal mol^−1^) relative to cyclohexanol **6** (22.4 kcal mol^−1^) in chloroform (see Supporting Information for details). This provides further support for the accuracy of the model developed within this investigation and highlights the immense influence the solvent has on reaction outcome.

**Scheme 10:**
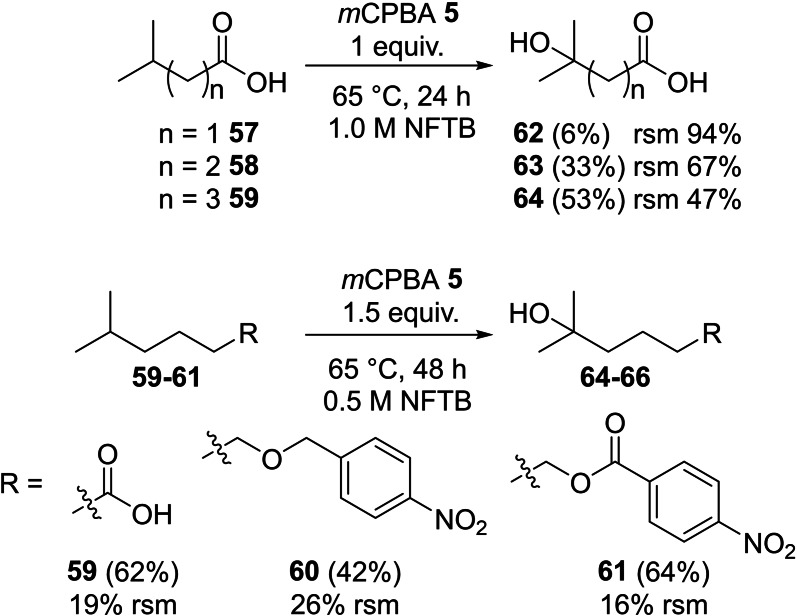
Selective oxidation of tertiary centres.

The influence of proximal functional groups on the outcome of the oxidation of tertiary centres was also examined (Scheme 10). Whilst the reaction of 3‐methylbutanoic acid **57** was unsuccessful under typical reaction conditions (65 °C, 1.0 M., 24 h; 94 % rsm), the reaction of more remote tertiary centres proceeded selectively with 4‐methyl pentanoic acid **58** (33 %) and 5‐methylhexanoic acid **59** (53 %) all showing remarkable selectivity for the process and leading to the corresponding tertiary alcohol **63–64**. The conversion in these transformations could be increased by extending the reaction time and the quantity of peracid present (**59**; 1.5 equiv. *m*CPBA **5**, 0.5 M, 65 °C, 48 h, 62 % yield) showing that optimisation of reaction conditions for individual substrates is possible. Along with substrates containing carboxylic acids the reaction also proved effective in the selective oxidation of ether (**60**; 42 %) and ester (**61**; 64 %) containing substrates suggesting that this selective oxidation process may also be extended to more complex and challenging substrates.

## Conclusion

In conclusion, we report a detailed investigation on the peracid oxidation of aliphatic sp^3^ C−H bonds revealing a critical solvent effect that dramatically influences product distribution and yield. The oxidation of cyclohexane **4** with *m*CPBA **5** in chlorinated solvents is low yielding and non‐selective, resulting in mixtures of cyclohexanol **6**, cyclohexanone **7**, and *ϵ*‐caprolactone **8** as the principal components of the crude reaction mixture. The reasons for this lack of selectivity in chlorinated solvents are two‐fold: First, the cyclohexanol product **6** has a lower energy barrier to oxidation than the starting cyclohexane **4**, leading to overoxidation. Second, the products generated **6–8** may H‐bond to unreacted *m*CPBA **5**, reducing its availability for the reaction, in a similar manner to the poor reactivity observed in HBA solvents such as THF.

It is possible to overcome the low reaction yields and poor selectivity of the process by changing the reaction medium. Switching solvent to a fluorinated alcohol such as NFTB or HFIP leads to a significantly higher yield of cyclohexanol **6** (86 %) and considerably improved reaction selectivity. The origins of this remarkable solvent effect were established through a combination of DFT calculations, kinetic measurement and DOSY NMR experiments. H‐Bonding of the cyclohexanol product **6** to the fluorinated alcohol solvent, reduces the ability of the oxygen lone pair of cyclohexanol to stabilise a developing positive charge on the *α*‐carbon atom in the oxidation to cyclohexanone, raising the transition state barrier of this overoxidation. In addition, the H‐bonding interaction of cyclohexanol **6** with *m*CPBA **5**, which promotes overoxidation (see above), is disfavoured due to the presence of the fluorinated alcohol solvent, resulting in improved selectivity. These findings are in line with previous reports of the dramatic effect fluorinated alcohols can have on reaction outcome and further highlight the influence of this reaction medium on synthetic transformations. Whilst the computational model enables the rapid evaluation of substrates within this oxidation, refinement has the potential to provide further insight.

Central to the successful outcome of this investigation was the development of a computational transition state model for the oxidation that provided clear insight into the subtle H‐bonding effects observed. Refinement of this model in conjunction with experimental observation could provide a simple and effective method for both the prediction and identification of sites susceptible to reaction with electrophilic oxidants. We believe this to have application in diverse areas of chemistry and our current investigations are focused on applying this model to predict metabolic sites within molecules of pharmaceutical interest.

Fluorinated alcohols have been noted to have a remarkable influence on the outcome of a number of synthetic transformations.[^15^, ^16^, ^17^, ^18^, ^19^, ^20^, ^21^, ^22^, ^23^, ^24^, ^25^, ^26^, ^27^, ^28^] It is possible that the subtle yet important effects identified within this study combined with the observations of others may provide insight into these processes.

## Experimental Section

Calculations were conducted using GAUSSIAN16 software package. Geometry optimisation followed by frequency calculations were done at a triple‐ζ level of theory using the D3 version of Grimme's dispersion with diffuse polarisation of the orbitals B3LYP‐GD3/6‐311++G(d,p). Optimised structures were confirmed as energy minima by absence of imaginary frequencies in the vibrational analysis. Transition states were confirmed as first order saddle points on the potential energy surface by the presence of only one imaginary frequency in the vibrational analysis. Calculated transition states were confirmed as true by following the intrinsic reaction coordinate (IRC).

Typical experimental procedure for oxidation of 2° sp^3^ C−H bonds in cyclic alkanes: In a 0.5–2.5 mL μwave vial 86 mg (0.5 mmol) of 100 % or 92 mg (0.5 mmol) of 90–93 % *m*CPBA **5** were suspended in 0.5 mL of nonafluoro‐*tert*‐butanol followed by addition of substrate (2.5 mmol, 5.0 equiv.). The reaction vial was sealed, thermally incubated, and stirred for the desired time. The reaction was cooled in air to ambient temperature, followed by addition of 1 mL of 21 mg/mL (0.125 mmol 0.25 equiv.) solution of 1,4‐dinitrobenzene in CDCl_3_. The reaction mixture was homogenised by addition of 4 mL of CDCl_3_ and analysed by ^1^H, ^13^C and ^19^F NMR spectroscopy. Reaction products were compared to literature values of authentic compounds.

Typical experimental procedure for oxidation of 2° sp^3^ C−H bonds in acyclic alkanes: In a 0.5–2.5 mL μwave vial 86 mg (0.5 mmol) of 100 % or 92 mg (0.5 mmol) of 93 % *m*CPBA **5** were suspended in 0.5 mL of nonafluoro‐*tert*‐butanol followed by addition of substrate (2.5 mmol, 5.0 equiv.). The reaction vial was sealed, thermally incubated, and stirred for the desired time. The reaction was cooled in air to ambient temperature, followed by addition of 1 mL of 21 mg/mL (0.125 mmol 0.25 equiv.) solution of 1,4‐dinitrobenzene in CDCl_3_. The reaction mixture was homogenised by addition of 4 mL of CDCl_3_ and analysed by ^1^H, ^13^C and ^19^F NMR spectroscopy. Reaction products were compared to literature values of authentic compounds.

Typical experimental procedure for oxidation of 3° sp^3^ C−H bonds in cyclic alkanes: In a 0.5–2.5 mL μwave vial 129 mg (0.75 mmol, 1.5 equiv.) of 100 % or 138 mg (0.75 mmol, 1.5 equiv.) of 93 % *m*CPBA **5** were suspended in 0.5 mL of nonafluoro‐*tert*‐butanol followed by addition of substrate (0.5 mmol, 1.0 equiv.). The reaction vial was sealed, thermally incubated, and stirred for the desired time. The reaction was cooled in air to ambient temperature, followed by addition of 1 mL of 21 mg/mL (0.125 mmol 0.25 equiv.) solution of 1,4‐dinitrobenzene in CDCl_3_. The reaction mixture was homogenised by addition of 4 mL of CDCl_3_ and analysed by ^1^H, ^13^C and ^19^F NMR spectroscopy. Reaction products were compared to literature values of authentic compounds.

Typical experimental procedure for oxidation of 3° sp^3^ C−H bonds in acyclic alkanes: In a 0.5–2.5 mL μwave vial 129 mg (0.75 mmol, 1.5 equiv.) of 100 % or 138 mg (0.75 mmol, 1.5 equiv.) of 93 % *m*CPBA **5** were suspended in 0.5 mL of nonafluoro‐*tert*‐butanol followed by addition of substrate (0.5 mmol, 1.0 equiv.). The reaction vial was sealed, thermally incubated, and stirred for the desired time. The reaction was cooled down, followed by addition of 1 mL of 21 mg/mL (0.125 mmol 0.25 equiv.) solution of 1,4‐dinitrobenzene in CDCl_3_. The reaction mixture was homogenised by addition of 4 mL of CDCl_3_ and analysed by ^1^H ^13^C and ^19^F NMR. Reaction products were compared towards literature reported compounds.

## Conflict of interest

The authors declare no conflict of interest.

1

## Supporting information

As a service to our authors and readers, this journal provides supporting information supplied by the authors. Such materials are peer reviewed and may be re‐organized for online delivery, but are not copy‐edited or typeset. Technical support issues arising from supporting information (other than missing files) should be addressed to the authors.

Supporting Information

## Data Availability

The data that support the findings of this study are available in the supplementary material of this article.
