# Supplementary material for: Peracid Oxidation of Unactivated sp3 C−H Bonds: An Important Solvent Effect
Source: Chemistry. 2023 Apr 24;29(31):e202204007. doi: 10.1002/chem.202204007 (PMC10946557; doi:10.1002/chem.202204007)

Chemistry–A European Journal

Supporting Information

Peracid Oxidation of Unactivated sp^3 C–H Bonds: An Important Solvent Effect

Sergej Maciuk, Susanna H. Wood, Vipulkumar K. Patel, Peter D. P. Shapland, and Nicholas C. O. Tomkinson*

Contents

1 Calculations. 4

1.1 Graphical summary of computational investigation. 7

1.2 Ground state calculations. 8

1.3 Transition state calculations. 20

2 General experimental details. 39

2.1 Procedure for purification of *m*CPBA 5 39

2.2 Oxidation of 2° sp3 C–H bonds. 40

2.2.1 Typical experimental procedure for oxidation of cyclic alkanes. 40

2.2.2 Typical experimental procedure for oxidation of acyclic alkanes. 40

2.3 Oxidation of 3° sp3 C–H bonds. 40

2.3.1 Typical experimental procedure for oxidation of cyclic alkanes. 40

2.3.2 Typical experimental procedure for oxidation of acyclic alkanes. 40

2.4 Typical experimental procedure for oxidation of functionalised molecules. 41

2.5 Compound characterisation. 41

3 Kinetic investigation of the transformation. 44

3.1 Determination of reactant orders. 45

3.2 Kinetic investigation of oxidation of cyclohexane 4 in NFTB. 48

3.3 Eyring plots for oxidation of cyclohexane 4 in NFTB. 53

3.4 Kinetic investigation of oxidation of cyclohexanol 6 in $CDCl_3$. 54

3.5 Eyring plots for oxidation of cyclohexanol 6 in $CDCl_3$. 59

3.6 Kinetic investigation of oxidation of cyclohexanol 6 in NFTB. 60

3.7 Eyring plots for oxidation of cyclohexanol 6 in NFTB. 65

4 Analyses of the crude reaction mixtures. 65

4.1 Competition oxidation experiments 93

4.2 Competition experiment between cyclohexane and cyclopentane. 103

5 DOSY experiments. 110

5.1 Experimental for DOSY NMR experiments 110

5.1.1 DOSY cyclohexane/TMS/0 eq NFTB. 111

5.1.2 DOSY cyclohexane/TMS/1 eq NFTB. 113

5.1.3 DOSY cyclohexane/TMS/2 eq NFTB. 115

5.1.4 DOSY cyclohexane/TMS/3 eq NFTB. 117

5.1.5 DOSY mCPBA/TMS/0 eq NFTB 119
5.1.6 DOSY mCPBA/TMS/1 eq NFTB 122
5.1.7 DOSY mCPBA/TMS/2 eq NFTB 125
5.1.8 DOSY mCPBA/TMS/3 eq NFTB 128
5.1.9 DOSY Cyclohexanol/TMS/0 eq NFTB 131
5.1.10 DOSY Cyclohexanol/TMS/1 eq NFTB 135
5.1.11 Cyclohexanol/TMS/2 eq NFTB 139
5.1.12 DOSY Cyclohexanol/TMS/3 eq NFTB 143
5.1.13 DOSY THF/mCPBA/TMS 149
5.1.14 DOSY mCPBA/THF/1 eq NFTB/TMS 153
5.1.15 DOSY *m*CPBA/THF/+2 eq NFTB/TMS 157
5.1.16 DOSY *m*CPBA/THF/+3 eq/NFTB/TMS 162
6 Copies of NMR spectra of novel products. 166

1 Calculations.

Calculations were conducted using GAUSSIAN16 software package. Geometry optimisation followed by frequency calculations were done at a triple-ζ level of theory using the D3 version of Grimme's dispersion with diffuse polarisation of the orbitals B3LYP-GD3/6-311++G(d,p). Optimised structures were confirmed as energy minima by absence of imaginary frequencies in the vibrational analysis. Transition states were confirmed as first order saddle points on the potential energy surface by the presence of only one imaginary frequency in the vibrational analysis. Calculated transition states were confirmed as true by following the intrinsic reaction coordinate (IRC). Where solvation was included in calculations, SMD solvation model was used with the internal parameter specified by the solvent keyword (e.g. chloroform), for HFIP the solvent was specified generic solvent and the solvent parameters read from the input file. Solvent parameters used for HFIP are as follows:

Eps=17.8

EpsInf=1.625626

HbondAcidity=1.96

HbondBasicity=0.00

SurfaceTensionAtInterface=23.23

CarbonAromaticity=0.00

ElectronegativeHalogenicity=0.6

Click on Figure to view animation of the oxidation of cyclohexane **4** with *m*CPBA **5**.

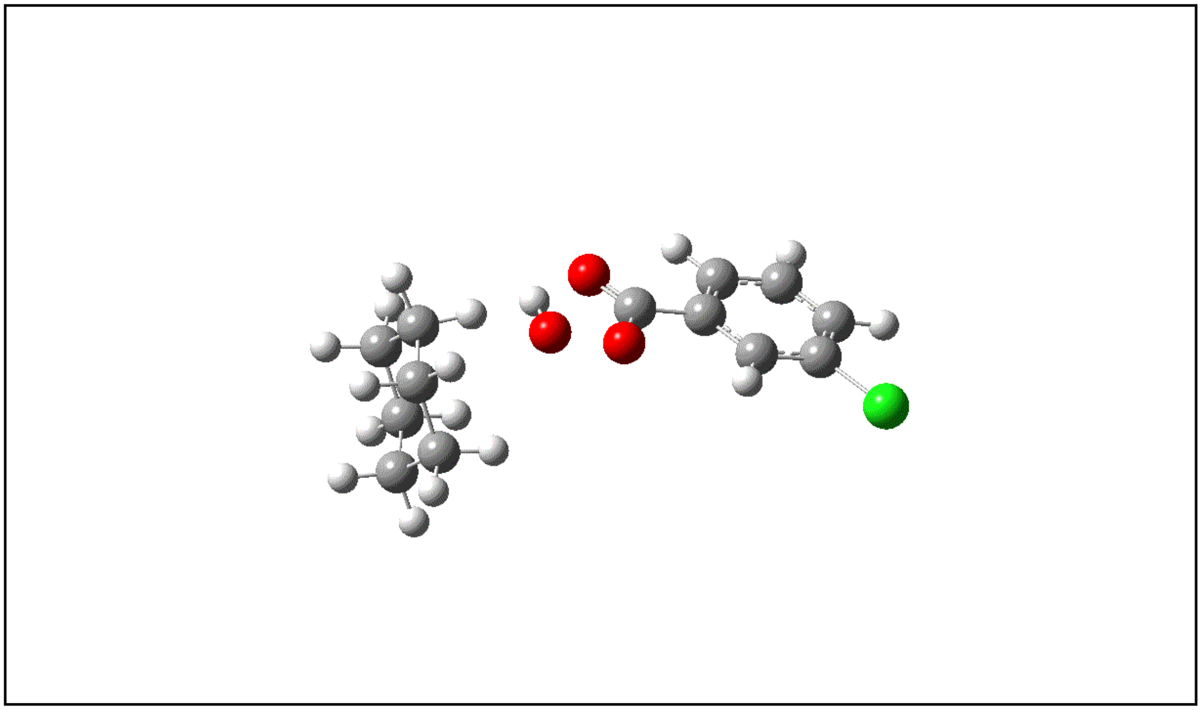

Figure S1. IRC calculation of oxidation of cyclohexane H_{ax} with *m*CPBA.

SMD(HFIP)-B3LYP-GD3/6-311++G(d,p) level of theory.

To view this as an animation you will need an updated version of your .pdf reader. If you are unable to visualise this please contact the corresponding author.

Click on Figure to view animation of the oxidation of propanol **12** with *m*CPBA **5**.

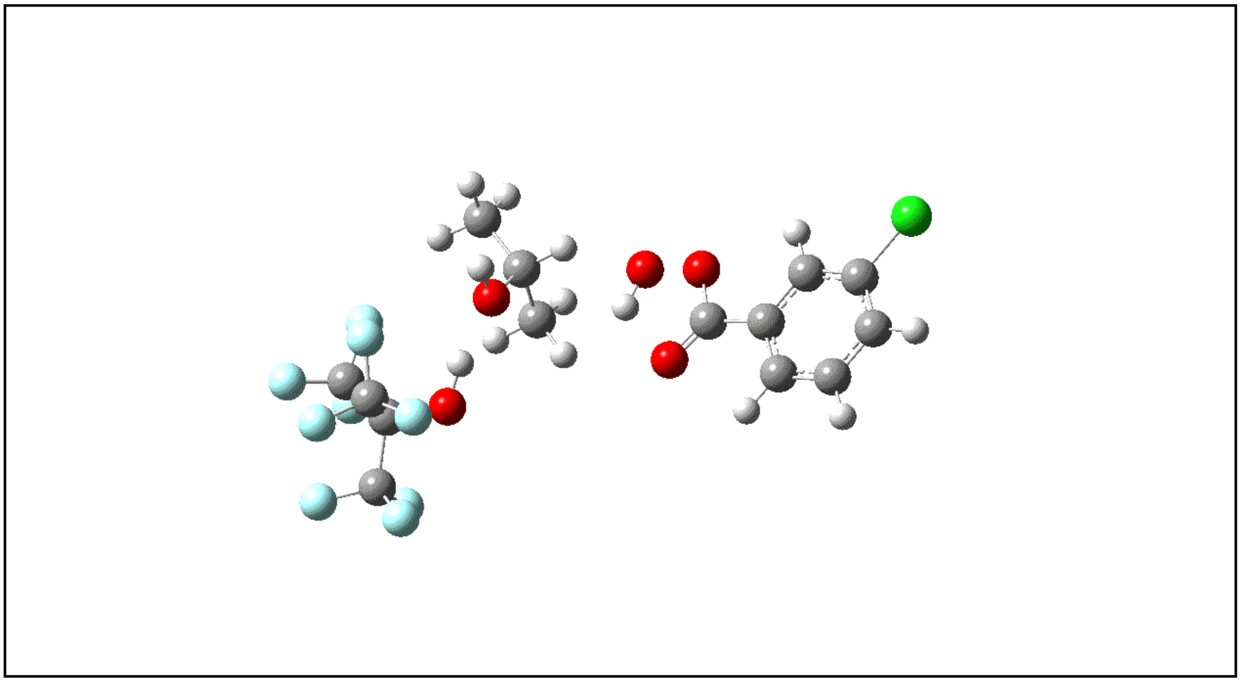

Figure S2. IRC calculation of oxidation of propanol *m*CPBA.

SMD(HFIP)-B3LYP-GD3/6-311++G(d,p) level of theory.

To view this as an animation you will need an updated version of your .pdf reader. If you are unable to visualise this please contact the corresponding author.

Animation shows *m*CPBA **5** accepts hydrogen atom from substrate alcohol **12**. The developing positive charge is stabilised by oxygen lone pair on the alcohol. The molecule of NFTB essentially remains static throughout the oxidation process.

1.1 Graphical summary of computational investigation.

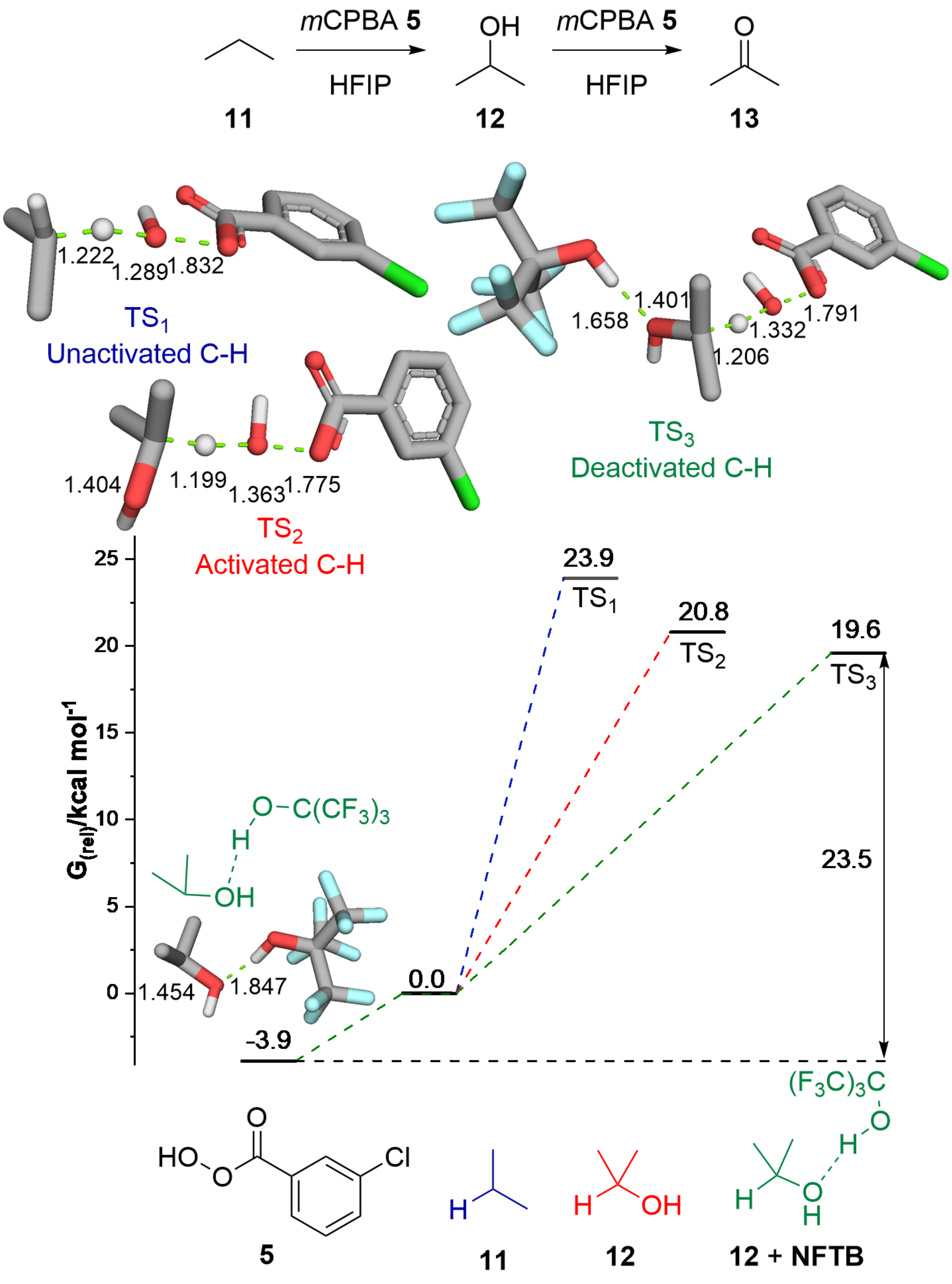

Figure S3. Summary of transition state energies of oxidation of alkanes *with* mCPBA. SMD(HFIP)-B3LYP-GD3/6-311++G(d,p) level of theory.

1.2 Ground state calculations.

Compound	*m*CPBA **5**
Functional/Basis set	B3LYP-GD3/6-311++G(d,p)
Solvation	none
Imaginary Frequency (cm^{-1})	none
E(RB3LYP) (A.U.)	-955.727665741
Corrections	Zero-point correction= 0.108544 (Hartree/Particle)<br>Thermal correction to Energy= 0.118073<br>Thermal correction to Enthalpy= 0.119017<br>Thermal correction to Gibbs Free Energy= 0.072485
Coordinates	0 1<br>C 1.11130 -0.00881 0.09360<br>O 1.21547 1.27116 -0.36902<br>O 1.99215 1.23521 -1.60577<br>H 2.36320 2.13056 -1.52819<br>O 1.60260 -1.01315 -0.39966<br>C 0.27151 -0.04203 1.32462<br>C -0.01956 -1.29492 1.88794<br>C -0.23063 1.11631 1.92971<br>C -0.80148 -1.39001 3.04073<br>H 0.36257 -2.20551 1.42680<br>C -1.01311 1.01222 3.08257<br>H -0.02286 2.10142 1.52078<br>C -1.29740 -0.23288 3.64007<br>H -1.02193 -2.36502 3.46967<br>H -1.90698 -0.30927 4.53680<br>Cl -1.62487 2.43760 3.82609

Compound	propane **11**
Functional/Basis set	B3LYP-GD3/6-311++G(d,p)
Solvation	none
Imaginary Frequency (cm^{-1})	none
E(RB3LYP) (A.U.)	-119.185459932
Corrections	Zero-point correction= 0.103027 (Hartree/Particle)<br>Thermal correction to Energy= 0.107522<br>Thermal correction to Enthalpy= 0.108466<br>Thermal correction to Gibbs Free Energy= 0.077403
Coordinates	0 1<br>C 1.05375 -0.00997 0.07213<br>H 0.69886 0.83231 -0.53038<br>H 0.68630 -0.93377 -0.38555<br>H 2.14770 -0.02324 0.03327<br>C 0.56108 0.09982 1.50517<br>H -0.53436 0.08250 1.51661<br>H 0.90659 -0.76841 2.07719

	C 1.05381 1.37322 2.17153<br>H 0.69894 2.25922 1.63537<br>H 0.68639 1.42923 3.20097<br>H 2.14776 1.40365 2.19903

Compound	propanol **12**
Functional/Basis set	B3LYP-GD3/6-311++G(d,p)
Solvation	none
Imaginary Frequency (cm^{-1})	none
E(RB3LYP) (A.U.)	-194.431089500
Corrections	Zero-point correction= 0.107633 (Hartree/Particle)<br>Thermal correction to Energy= 0.113053<br>Thermal correction to Enthalpy= 0.113997<br>Thermal correction to Gibbs Free Energy= 0.080273
Coordinates	0 1<br>C 1.01238 -0.05115 0.10079<br>H 0.64460 -1.06635 0.28470<br>H 0.61246 0.25617 -0.87180<br>H 0.60741 0.61031 0.87265<br>C 2.53331 -0.02926 0.09623<br>H 2.91650 -0.39106 1.05641<br>O 3.01412 -0.89709 -0.92527<br>H 2.67666 -1.78906 -0.73514<br>C 3.07172 1.36690 -0.17216<br>H 2.74562 1.73253 -1.15221<br>H 4.16693 1.35656 -0.18995<br>H 2.74215 2.07492 0.59424

Compound	*m*CPBA **5**
Functional/Basis set	B3LYP-GD3/6-311++G(d,p)
Solvation	SMD HFIP
Imaginary Frequency (cm^{-1})	none
E(RB3LYP) (A.U.)	-955.703840502
Corrections	Zero-point correction= 0.107619 (Hartree/Particle)<br>Thermal correction to Energy= 0.117365<br>Thermal correction to Enthalpy= 0.118309<br>Thermal correction to Gibbs Free Energy= 0.071265
Coordinates	0 1<br>C 0.98062 0.06071 0.15259<br>O 0.46274 1.32022 0.22627<br>O -0.98713 1.19448 0.35654<br>H -1.20045 2.09623 0.06105<br>O 0.36664 -0.99559 0.16792<br>C 2.46864 0.12963 0.04966<br>C 3.18267 -1.07896 0.00993<br>C 3.16759 1.34231 -0.01184

	C 4.57524 -1.07677 -0.09194<br>H 2.65272 -2.03015 0.05882<br>C 4.55984 1.33444 -0.11381<br>H 2.64612 2.29575 0.01803<br>C 5.26424 0.13337 -0.15569<br>H 5.11909 -2.01964 -0.12310<br>H 6.34912 0.13255 -0.23713<br>Cl 5.41498 2.82618 -0.19228

Compound	propane **11**
Functional/Basis set	B3LYP-GD3/6-311++G(d,p)
Solvation	SMD HFIP
Imaginary Frequency (cm^{-1})	none
E(RB3LYP) (A.U.)	-119.186105944
Corrections	Zero-point correction= 0.102701 (Hartree/Particle)<br>Thermal correction to Energy= 0.107186<br>Thermal correction to Enthalpy= 0.108131<br>Thermal correction to Gibbs Free Energy= 0.077088
Coordinates	0 1<br>C 0.97012 -0.02848 0.08352<br>H 0.61525 0.92124 -0.32939<br>H 0.60270 -0.83604 -0.55739<br>H 2.06407 -0.03333 0.04279<br>C 0.47738 -0.22104 1.50777<br>H -0.61806 -0.24035 1.51528<br>H 0.82285 -1.18977 1.88543<br>C 0.97009 0.88468 2.42591<br>H 0.61523 1.86329 2.08706<br>H 0.60264 0.72399 3.44427<br>H 2.06404 0.90869 2.45920

Compound	propanol **12**
Functional/Basis set	B3LYP-GD3/6-311++G(d,p)
Solvation	SMD HFIP
Imaginary Frequency (cm^{-1})	none
E(RB3LYP) (A.U.)	-194.432423943
Corrections	Zero-point correction= 0.107445 (Hartree/Particle)<br>Thermal correction to Energy= 0.112829<br>Thermal correction to Enthalpy= 0.113773<br>Thermal correction to Gibbs Free Energy= 0.080120
Coordinates	0 1<br>C 0.93800 -0.08740 0.05699<br>H 0.57010 -1.06038 -0.28616<br>H 0.53815 0.66137 -0.63566<br>H 0.53310 0.10488 1.05515<br>C 2.45894 -0.06634 0.06383

	H 2.84204 -0.85633 0.71868<br>O 2.93966 -0.31421 -1.25343<br>H 2.60215 -1.18319 -1.53013<br>C 2.99748 1.27936 0.52233<br>H 2.67146 2.08253 -0.14787<br>H 4.09268 1.27908 0.50173<br>H 2.66795 1.51476 1.53884

Compound	NFTB
Functional/Basis set	B3LYP-GD3/6-311++G(d,p)
Solvation	SMD HFIP
Imaginary Frequency (cm^{-1})	none
E(RB3LYP) (A.U.)	-1127.19983131
Corrections	Zero-point correction= 0.065028 (Hartree/Particle)<br>Thermal correction to Energy= 0.077609<br>Thermal correction to Enthalpy= 0.078553<br>Thermal correction to Gibbs Free Energy= 0.025877
Coordinates	0 1<br>C 1.00608 -0.16506 0.11609<br>C 0.39075 -1.28858 -0.65467<br>C -1.11672 -1.20462 -0.57456<br>F 1.11419 -0.46743 1.43187<br>F 0.34534 1.00433 0.04012<br>F 2.26762 0.10484 -0.29289<br>F -1.60106 0.05241 -0.56718<br>F -1.56977 -1.80049 0.55501<br>F -1.73596 -1.83515 -1.60328<br>O 0.87017 -2.59818 -0.16447<br>H 1.84569 -2.55819 -0.10628<br>C 0.80039 -1.09630 -2.08984<br>F 2.13393 -1.37795 -2.21854<br>F 0.59531 0.14234 -2.55837<br>F 0.19146 -1.96956 -2.94438

Compound	NFTB/propanol H-bond complex
Functional/Basis set	B3LYP-GD3/6-311++G(d,p)
Solvation	SMD HFIP
Imaginary Frequency (cm^{-1})	none
E(RB3LYP) (A.U.)	-1321.65651248
Corrections	Zero-point correction= 0.173569 (Hartree/Particle)<br>Thermal correction to Energy= 0.192862<br>Thermal correction to Enthalpy= 0.193807<br>Thermal correction to Gibbs Free Energy= 0.123988
Coordinates	0 1<br>C 1.04935 -0.02782 0.08240<br>H 1.75061 1.30441 -0.30713

	H 1.83690 -0.62100 1.28610<br>H -0.40143 0.26060 0.56162<br>C 0.92812 -1.28324 -1.48214<br>H -0.12877 -1.57170 -1.52583<br>O 1.65715 -2.44096 -1.12068<br>H 2.45505 -2.54410 -1.67420<br>C 1.25797 -0.88339 -2.92597<br>H 2.27627 -0.53660 -3.08518<br>H 0.55905 -0.15509 -3.34042<br>H 1.16784 -1.77712 -3.55927<br>C 1.04935 -0.02782 0.08240<br>C 0.15728 -1.55241 1.01642<br>C 3.01372 -0.17660 -0.19362<br>C -0.00633 1.17128 -1.09783<br>O 1.10643 1.17032 1.57613<br>H 0.45948 1.86404 1.32517<br>F -0.86855 -2.06622 0.30128<br>F 1.01488 -2.53887 1.35180<br>F -0.39077 -1.09292 2.18076<br>F -0.40887 2.24302 -0.35575<br>F -1.10685 0.55742 -1.57636<br>F 0.73417 1.64910 -2.12005<br>F 3.50665 -1.39043 0.13067<br>F 3.65758 0.74353 0.57790<br>F 3.35606 0.10073 -1.46943

Compound	Cyclohexane **4**
Functional/Basis set	B3LYP-GD3/6-311++G(d,p)
Solvation	SMD Chloroform
Imaginary Frequency (cm^{-1})	none
E(RB3LYP) (A.U.)	-235.964831861
Corrections	Zero-point correction= 0.169380 (Hartree/Particle)<br>Thermal correction to Energy= 0.175056<br>Thermal correction to Enthalpy= 0.176000<br>Thermal correction to Gibbs Free Energy= 0.140650
Coordinates	0 1<br>C 0.73792 1.26300 -0.22023<br>C 1.46530 -0.00045 0.23204<br>C 0.73594 -1.26292 -0.21967<br>C -0.72318 -1.26114 0.22832<br>C -1.44961 0.00174 -0.22669<br>C -0.72126 1.26367 0.22787<br>H 0.78109 -1.33814 -1.31315<br>H 2.48583 -0.00131 -0.16708<br>H 1.54970 -0.00026 1.32577<br>H 0.78307 1.33759 -1.31376<br>H 1.24758 2.14587 0.18155

	H -1.23312 -2.14464 -0.17187<br>H -0.76865 -1.33358 1.32199<br>H -1.53025 0.00162 -1.32074<br>H -2.47143 0.00261 0.16905<br>H -0.76645 1.33645 1.32155<br>H -1.22979 2.14784 -0.17254<br>H 1.24425 -2.14644 0.18257

Compound	*m*CPBA **5**
Functional/Basis set	B3LYP-GD3/6-311++G(d,p)
Solvation	SMD Chloroform
Imaginary Frequency (cm^{-1})	none
E(RB3LYP) (A.U.)	-955.739904919
Corrections	Zero-point correction= 0.108080 (Hartree/Particle)<br>Thermal correction to Energy= 0.117745<br>Thermal correction to Enthalpy= 0.118689<br>Thermal correction to Gibbs Free Energy= 0.071626
Coordinates	0 1<br>C 1.11299 0.06312 0.05161<br>O 2.17953 -0.55090 0.64881<br>O 2.81761 0.41068 1.54217<br>H 3.64004 0.50822 1.02948<br>O 0.75801 1.22019 0.18545<br>C 0.48809 -0.89539 -0.90185<br>C -0.28549 -0.37912 -1.95382<br>C 0.66853 -2.27893 -0.79097<br>C -0.84284 -1.23759 -2.90362<br>H -0.44557 0.69514 -2.04211<br>C 0.10803 -3.12857 -1.74287<br>H 1.24065 -2.70442 0.03124<br>C -0.63722 -2.61418 -2.80271<br>H -1.43040 -0.83415 -3.72432<br>H -1.06403 -3.27983 -3.54907<br>Cl 0.34749 -4.82994 -1.61466

Compound	Cyclohexanol **6** axial OH conformer
Functional/Basis set	B3LYP-GD3/6-311++G(d,p)
Solvation	SMD Chloroform
Imaginary Frequency (cm^{-1})	none
E(RB3LYP) (A.U.)	-311.213829075
Corrections	Zero-point correction= 0.173885 (Hartree/Particle)<br>Thermal correction to Energy= 0.180713<br>Thermal correction to Enthalpy= 0.181657<br>Thermal correction to Gibbs Free Energy= 0.143604
Coordinates	0 1<br>C 0.73729 1.25771 -0.22042

	C -0.72136 1.26545 0.23105<br>C -1.45539 0.00794 -0.22281<br>C -0.73234 -1.25843 0.23373<br>C 0.72609 -1.26752 -0.22217<br>C 1.46237 -0.00947 0.22715<br>H -1.55090 0.00783 -1.31650<br>H -0.76406 1.33771 1.32478<br>H -1.22641 2.15233 -0.16777<br>H 0.77973 1.33381 -1.31398<br>H 1.25190 2.13791 0.18109<br>H -0.77209 -1.32595 1.32717<br>H 0.77321 -1.35480 -1.31552<br>H 1.23074 -2.15945 0.16815<br>H 2.48096 -0.01365 -0.17682<br>H 1.55199 -0.01080 1.32042<br>H -2.48139 0.00913 0.16426<br>O -1.40192 -2.40386 -0.28184<br>H -1.37746 -2.35347 -1.25277

Compound	Cyclohexanol **6** equatorial OH conformer
Functional/Basis set	B3LYP-GD3/6-311++G(d,p)
Solvation	SMD Chloroform
Imaginary Frequency (cm^{-1})	none
E(RB3LYP) (A.U.)	-311.215190857
Corrections	Zero-point correction= 0.173822 (Hartree/Particle)<br>Thermal correction to Energy= 0.180689<br>Thermal correction to Enthalpy= 0.181633<br>Thermal correction to Gibbs Free Energy= 0.143481
Coordinates	0 1<br>C 0.75350 1.24755 -0.18216<br>C 1.48441 -0.03375 0.21935<br>C 0.74277 -1.28071 -0.25291<br>C -0.70875 -1.29315 0.21752<br>C -1.44600 -0.01691 -0.18679<br>C -0.71248 1.25118 0.25332<br>H 1.25643 -2.17575 0.11595<br>H 1.60297 -0.06831 1.30888<br>H 2.49477 -0.02558 -0.20560<br>H 1.27673 2.10403 0.25896<br>H 0.80400 1.35708 -1.27252<br>H -0.74000 -1.41144 1.30716<br>H -1.22402 -2.16233 -0.20698<br>H -2.45160 -0.03758 0.24894<br>H -1.56039 -0.00416 -1.27795<br>H -1.21288 2.12330 -0.18132<br>H 0.76991 -1.32656 -1.34857<br>O -0.80305 1.40320 1.66852

	H -0.36460 0.64693 2.09170

Compound	Cyclohexane **4**
Functional/Basis set	B3LYP-GD3/6-311++G(d,p)
Solvation	SMD HFIP
Imaginary Frequency (cm^{-1})	none
E(RB3LYP) (A.U.)	-235.961363969
Corrections	Zero-point correction= 0.169356 (Hartree/Particle)<br>Thermal correction to Energy= 0.175021<br>Thermal correction to Enthalpy= 0.175965<br>Thermal correction to Gibbs Free Energy= 0.140632
Coordinates	0 1<br>C 0.73792 1.26300 -0.22023<br>C 1.46530 -0.00045 0.23204<br>C 0.73594 -1.26292 -0.21967<br>C -0.72318 -1.26114 0.22832<br>C -1.44961 0.00174 -0.22669<br>C -0.72126 1.26367 0.22787<br>H 0.78109 -1.33814 -1.31315<br>H 2.48583 -0.00131 -0.16708<br>H 1.54970 -0.00026 1.32577<br>H 0.78307 1.33759 -1.31376<br>H 1.24758 2.14587 0.18155<br>H -1.23312 -2.14464 -0.17187<br>H -0.76865 -1.33358 1.32199<br>H -1.53025 0.00162 -1.32074<br>H -2.47143 0.00261 0.16905<br>H -0.76645 1.33645 1.32155<br>H -1.22979 2.14784 -0.17254<br>H 1.24425 -2.14644 0.18257

Compound	Cyclohexanol **6** axial OH conformer
Functional/Basis set	B3LYP-GD3/6-311++G(d,p)
Solvation	SMD HFIP
Imaginary Frequency (cm^{-1})	none
E(RB3LYP) (A.U.)	-311.207886334
Corrections	Zero-point correction= 0.174003 (Hartree/Particle)<br>Thermal correction to Energy= 0.180753<br>Thermal correction to Enthalpy= 0.181697<br>Thermal correction to Gibbs Free Energy= 0.143840
Coordinates	0 1<br>C 0.73749 1.25815 -0.22038<br>C 1.46247 -0.00910 0.22710<br>C 0.72572 -1.26692 -0.22191<br>C -0.73236 -1.25751 0.23526<br>C -1.45563 0.00891 -0.22074

	C -0.72096 1.26645 0.23186<br>H 0.77180 -1.35398 -1.31530<br>H 2.48090 -0.01358 -0.17728<br>H 1.55251 -0.01039 1.32033<br>H 0.77941 1.33410 -1.31396<br>H 1.25254 2.13830 0.18075<br>H -0.77115 -1.32487 1.32875<br>H -1.55241 0.00847 -1.31432<br>H -2.48118 0.01037 0.16748<br>H -0.76301 1.33939 1.32557<br>H -1.22598 2.15322 -0.16723<br>H 1.23044 -2.15908 0.16782<br>O -1.40255 -2.40298 -0.27965<br>H -1.37827 -2.35278 -1.25042

Compound	Cyclohexanol **6** equatorial OH conformer
Functional/Basis set	B3LYP-GD3/6-311++G(d,p)
Solvation	SMD HFIP
Imaginary Frequency (cm^{-1})	none
E(RB3LYP) (A.U.)	-311.208051994
Corrections	Zero-point correction= 0.173898 (Hartree/Particle)<br>Thermal correction to Energy= 0.180712<br>Thermal correction to Enthalpy= 0.181656<br>Thermal correction to Gibbs Free Energy= 0.143620
Coordinates	0 1<br>C 0.75202 1.25002 -0.18074<br>C 1.48379 -0.03141 0.21882<br>C 0.74203 -1.27856 -0.25326<br>C -0.70958 -1.29146 0.21668<br>C -1.44667 -0.01442 -0.18663<br>C -0.71333 1.25230 0.25708<br>H 1.25581 -2.17344 0.11592<br>H 1.60377 -0.06663 1.30813<br>H 2.49362 -0.02268 -0.20735<br>H 1.27539 2.10634 0.26047<br>H 0.80111 1.36066 -1.27108<br>H -0.74142 -1.41104 1.30614<br>H -1.22484 -2.16019 -0.20895<br>H -2.45269 -0.03610 0.24835<br>H -1.56020 -0.00023 -1.27776<br>H -1.21454 2.12565 -0.17453<br>H 0.76951 -1.32468 -1.34888<br>O -0.80171 1.39933 1.67287<br>H -0.36339 0.64168 2.09333

Compound	Methylcyclohexane **51** axial methyl conformer

Functional/Basis set	B3LYP-GD3/6-311++G(d,p)
Solvation	SMD HFIP
Imaginary Frequency (cm^{-1})	none
E(RB3LYP) (A.U.)	-275.288279773
Corrections	Zero-point correction= 0.197431 (Hartree/Particle)<br>Thermal correction to Energy= 0.204412<br>Thermal correction to Enthalpy= 0.205356<br>Thermal correction to Gibbs Free Energy= 0.167080
Coordinates	0 1<br>C 0.75260 1.23154 -0.17737<br>C 1.49899 -0.05068 0.18727<br>C 0.75974 -1.29450 -0.29728<br>C -0.69049 -1.32196 0.17602<br>C -1.43036 -0.03740 -0.19298<br>C -0.70969 1.22889 0.29134<br>H 0.78281 -1.32467 -1.39356<br>H 2.50159 -0.02696 -0.25517<br>H 1.63802 -0.10587 1.27317<br>H 0.77174 1.34946 -1.26884<br>H 1.28278 2.09693 0.23754<br>H -1.20434 -2.18013 -0.27212<br>H -0.72054 -1.47171 1.26153<br>H -1.52749 0.00962 -1.28554<br>H -2.45023 -0.07139 0.20792<br>H 1.27924 -2.19244 0.05605<br>C -0.81769 1.39474 1.81028<br>H -1.86754 1.42544 2.12034<br>H -0.33320 0.57954 2.35581<br>H -0.34975 2.33275 2.12779<br>H -1.21072 2.09536 -0.15973

Compound	Methylcyclohexane **51** equatorial methyl conformer
Functional/Basis set	B3LYP-GD3/6-311++G(d,p)
Solvation	SMD HFIP
Imaginary Frequency (cm^{-1})	none
E(RB3LYP) (A.U.)	-275.291154007
Corrections	Zero-point correction= 0.197123 (Hartree/Particle)<br>Thermal correction to Energy= 0.204167<br>Thermal correction to Enthalpy= 0.205111<br>Thermal correction to Gibbs Free Energy= 0.166662
Coordinates	0 1<br>C 0.70748 1.26337 -0.18389<br>C -0.77245 1.26533 0.19537<br>C -1.47994 0.00051 -0.28179<br>C -0.77258 -1.26501 0.19306<br>C 0.70726 -1.26254 -0.18654<br>C 1.44531 -0.00015 0.28239

	H -1.52219 0.00169 -1.37822<br>H -0.87944 1.35844 1.28238<br>H -1.25999 2.14363 -0.24303<br>H 0.78884 1.33474 -1.27642<br>H 1.19294 2.15865 0.22241<br>H -1.26037 -2.14258 -0.24675<br>H -0.87925 -1.36003 1.27994<br>H 0.78835 -1.33152 -1.27924<br>H 1.19268 -2.15877 0.21763<br>H 2.44041 0.00023 -0.18131<br>H -2.51553 0.00032 0.07628<br>C 1.66142 -0.00177 1.79843<br>H 0.72010 -0.00137 2.35563<br>H 2.23005 -0.88648 2.10443<br>H 2.23165 0.88138 2.10594

Compound	Methylcyclohexane **51** axial methyl conformer
Functional/Basis set	B3LYP-GD3/6-311++G(d,p)
Solvation	SMD Chloroform
Imaginary Frequency (cm^{-1})	none
E(RB3LYP) (A.U.)	-275.292026799
Corrections	Zero-point correction= 0.197481 (Hartree/Particle)<br>Thermal correction to Energy= 0.204469<br>Thermal correction to Enthalpy= 0.205413<br>Thermal correction to Gibbs Free Energy= 0.167128
Coordinates	0 1<br>C 0.75192 1.23418 -0.17578<br>C 1.49860 -0.04807 0.18862<br>C 0.75961 -1.29225 -0.29536<br>C -0.69115 -1.31951 0.17528<br>C -1.43018 -0.03470 -0.19461<br>C -0.71044 1.23104 0.29273<br>H 0.78463 -1.32334 -1.39142<br>H 2.50107 -0.02413 -0.25397<br>H 1.63795 -0.10294 1.27443<br>H 0.77106 1.35232 -1.26715<br>H 1.28198 2.09959 0.23928<br>H -1.20458 -2.17748 -0.27394<br>H -0.72332 -1.46946 1.26067<br>H -1.52492 0.01308 -1.28741<br>H -2.45095 -0.06868 0.20402<br>H 1.27888 -2.19015 0.05948<br>C -0.81847 1.39416 1.81159<br>H -1.86850 1.42334 2.12158<br>H -0.33315 0.57881 2.35595<br>H -0.35190 2.33216 2.13088<br>H -1.21116 2.09804 -0.15774

Compound	Methylcyclohexane **51** equatorial methyl conformer
Functional/Basis set	B3LYP-GD3/6-311++G(d,p)
Solvation	SMD Chloroform
Imaginary Frequency (cm^{-1})	none
E(RB3LYP) (A.U.)	-275.294847156
Corrections	Zero-point correction= 0.197149 (Hartree/Particle)<br>Thermal correction to Energy= 0.204210<br>Thermal correction to Enthalpy= 0.205154<br>Thermal correction to Gibbs Free Energy= 0.166674
Coordinates	0 1<br>C 0.70606 1.26353 -0.18399<br>C -0.77360 1.26610 0.19591<br>C -1.48186 0.00155 -0.28020<br>C -0.77456 -1.26431 0.19428<br>C 0.70511 -1.26200 -0.18586<br>C 1.44346 0.00010 0.28309<br>H -1.52619 0.00221 -1.37636<br>H -0.87987 1.36043 1.28267<br>H -1.26098 2.14438 -0.24310<br>H 0.78708 1.33373 -1.27653<br>H 1.19226 2.15898 0.22093<br>H -1.26278 -2.14169 -0.24548<br>H -0.88092 -1.35942 1.28110<br>H 0.78599 -1.33104 -1.27849<br>H 1.19052 -2.15837 0.21829<br>H 2.43872 -0.00007 -0.18032<br>H -2.51712 0.00181 0.07937<br>C 1.65916 -0.00078 1.79908<br>H 0.71748 -0.00018 2.35563<br>H 2.22727 -0.88550 2.10575<br>H 2.22917 0.88231 2.10648

1.3 Transition state calculations.

Compound	*m*CPBA + propane TS<br>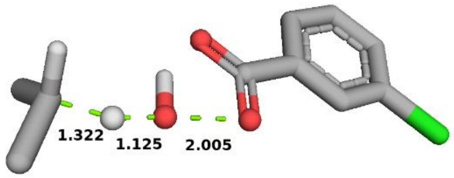
Functional/Basis set	B3LYP-GD3/6-311++G(d,p)
Solvation	none
Imaginary Frequency (cm^{-1})	-258.58
E(RB3LYP) (A.U.)	-1074.87523472
Corrections	Zero-point correction= 0.205461 (Hartree/Particle)<br>Thermal correction to Energy= 0.220882<br>Thermal correction to Enthalpy= 0.221826<br>Thermal correction to Gibbs Free Energy= 0.158875
Coordinates	0 1<br>C 0.92020 -0.04888 -0.03525<br>O 0.08125 -1.52281 0.11753<br>O -0.24575 1.15092 -0.30451<br>O 0.92020 -0.04888 -0.03525<br>H -0.42536 -0.12386 -0.42286<br>H 0.45567 -0.83773 1.02902<br>C 1.45191 3.49464 -0.76219<br>C 1.27078 2.97665 1.80857<br>C 0.80265 3.69203 0.58275<br>H 0.84603 3.97426 -1.53717<br>H 1.53320 2.45905 -1.06690<br>H 0.78268 2.00612 1.91558<br>H 2.35002 2.83071 1.83889<br>H -0.27244 3.80914 0.52636<br>C 2.85320 -0.09682 -0.10919<br>C 3.42215 -1.37904 -0.25404<br>C 3.67250 1.03654 -0.01656<br>C 4.80872 -1.52244 -0.31998<br>H 2.75843 -2.24329 -0.31263<br>C 5.05750 0.87574 -0.08429<br>H 3.25489 2.01383 0.12736<br>C 5.62971 -0.39164 -0.24227<br>H 5.24324 -2.50874 -0.44359<br>H 6.70830 -0.50455 -0.30230<br>Cl 6.08863 2.25507 0.02262<br>H 2.45048 3.93720 -0.78632<br>H 1.00387 3.56906 2.68754

Compound	*m*CPBA + propanol TS<br>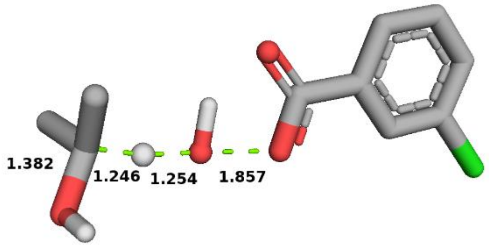
Functional/Basis set	B3LYP-GD3/6-311++G(d,p)
Solvation	none
Imaginary Frequency (cm^{-1})	-587.34
E(RB3LYP) (A.U.)	-1150.13274700
Corrections	Zero-point correction= 0.211840 (Hartree/Particle)<br>Thermal correction to Energy= 0.228003<br>Thermal correction to Enthalpy= 0.228948<br>Thermal correction to Gibbs Free Energy= 0.164430
Coordinates	0 1<br>C -0.63230 -2.41485 -1.63850<br>O -1.53870 -1.72586 -2.21804<br>O 0.35051 -2.97775 -2.23031<br>O 1.02426 0.08475 -0.04720<br>H 0.54460 -0.04231 -1.12049<br>H 1.02426 0.08475 -0.04720<br>C 3.19921 1.62360 -0.64601<br>C 3.22471 0.37736 1.54940<br>C 2.74208 0.33583 0.08057<br>H 2.91792 1.58420 -1.70404<br>H 4.29191 1.71531 -0.61717<br>H 2.95979 -0.55261 2.06825<br>H 4.31058 0.46027 1.59981<br>C -0.86641 -2.77522 -0.18034<br>C -0.23849 -3.89941 0.38139<br>C -1.75827 -2.03882 0.61936<br>C -0.56918 -4.34432 1.66241<br>H 0.50572 -4.42719 -0.21187<br>C -2.08283 -2.48134 1.90045<br>H -2.17419 -1.11620 0.21990<br>C -1.51489 -3.64908 2.41339<br>H -0.09306 -5.23408 2.05770<br>H -1.79465 -4.01089 3.39599<br>Cl -3.23104 -1.61892 2.85865<br>H 2.75638 2.51505 -0.19419<br>H 2.78291 1.22185 2.09242<br>O 3.45690 -0.76989 -0.55306<br>H 2.64841 -1.24661 -0.85892

Compound	*m*CPBA + propane TS<br>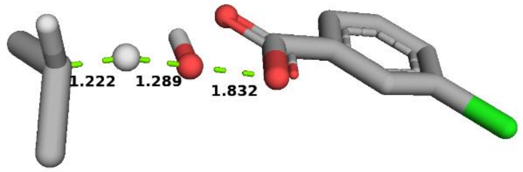
Functional/Basis set	B3LYP-GD3/6-311++G(d,p)
Solvation	SMD HFIP
Imaginary Frequency (cm^{-1})	-796.22
E(RB3LYP) (A.U.)	-1074.86333465
Corrections	Zero-point correction= 0.205666 (Hartree/Particle)<br>Thermal correction to Energy= 0.221010<br>Thermal correction to Enthalpy= 0.221955<br>Thermal correction to Gibbs Free Energy= 0.159901
Coordinates	0 1<br>C 0.93334 -0.06906 -0.02806<br>O -0.09752 0.51290 1.28430<br>O 0.16784 -1.38427 -0.93174<br>O 0.93334 -0.06906 -0.02806<br>H 0.24413 -0.99168 0.71910<br>H 0.93334 -0.06906 -0.02806<br>C 2.21258 1.17738 -2.16057<br>C 1.63407 2.66429 0.11848<br>C 1.23202 1.46244 -0.91261<br>H 1.67479 1.32514 -3.10091<br>H 2.49100 0.10991 -2.18900<br>H 1.66861 2.25497 1.15097<br>H 2.58041 3.16168 -0.04184<br>H 0.31063 1.81362 -1.36452<br>C 2.90539 -0.38465 0.34295<br>C 3.39480 -1.70558 0.38434<br>C 3.90684 0.63314 0.23670<br>C 4.78775 -1.97009 0.30694<br>H 2.65610 -2.51410 0.39663<br>C 5.27580 0.37027 0.12328<br>H 3.64053 1.65630 0.08011<br>C 5.71569 -0.94135 0.15885<br>H 5.12542 -2.98546 0.29446<br>H 6.77514 -1.14875 0.01772<br>Cl 6.37060 1.65231 -0.20574<br>H 3.15569 1.74282 -2.17557<br>H 0.85658 3.41055 0.14213

Compound	*m*CPBA + propanol TS<br>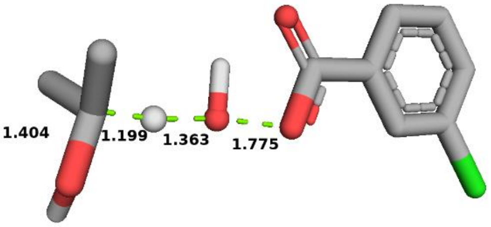
Functional/Basis set	B3LYP-GD3/6-311++G(d,p)
Solvation	SMD HFIP
Imaginary Frequency (cm^{-1})	-738.10
E(RB3LYP) (A.U.)	-1150.11649402
Corrections	Zero-point correction= 0.212316 (Hartree/Particle)<br>Thermal correction to Energy= 0.228720<br>Thermal correction to Enthalpy= 0.229664<br>Thermal correction to Gibbs Free Energy= 0.164721
Coordinates	0 1<br>C 0.97214 0.08680 -0.04110<br>O 1.12380 -1.33052 -1.09057<br>O 0.51397 -0.31929 1.61980<br>O 0.97214 0.08680 -0.04110<br>H 0.14286 -0.98716 0.15055<br>H 0.97214 0.08680 -0.04110<br>C 3.25298 1.72490 -0.62633<br>C 3.31657 0.44056 1.52025<br>C 2.75452 0.40485 0.06924<br>H 2.84915 1.80549 -1.63989<br>H 4.34493 1.71413 -0.73204<br>H 3.11127 -0.50597 2.03411<br>H 4.40889 0.55129 1.51531<br>C 0.03901 1.68958 -0.82748<br>C -0.77308 1.50464 -1.95994<br>C 0.25076 2.96068 -0.26337<br>C -1.35571 2.61744 -2.55798<br>H -0.91549 0.49020 -2.32296<br>C -0.28364 4.06120 -0.91669<br>H 0.86603 3.03846 0.62430<br>C -1.08978 3.89800 -2.05183<br>H -1.99694 2.48745 -3.42190<br>H -1.50708 4.76534 -2.55118<br>Cl 0.08060 5.65388 -0.36177<br>H 2.98180 2.61922 -0.05359<br>H 2.88553 1.26550 2.09847<br>O 3.52221 -0.65017 -0.61242<br>H 2.71491 -1.16988 -0.91066

Compound	*m*CPBA + propanol/NFTB H-bond complex TS 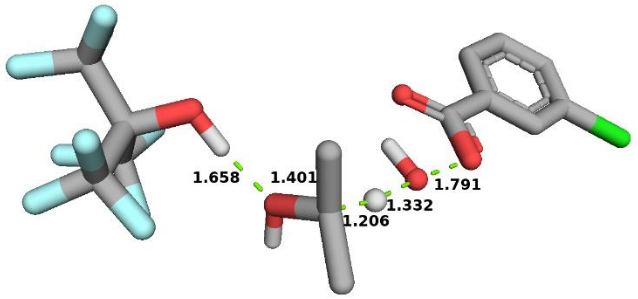
Functional/Basis set	B3LYP-GD3/6-311++G(d,p)
Solvation	SMD HFIP
Imaginary Frequency (cm^{-1})	-767.40
E(RB3LYP) (A.U.)	-2277.33599639
Corrections	Zero-point correction= 0.277370 (Hartree/Particle)<br>Thermal correction to Energy= 0.308052<br>Thermal correction to Enthalpy= 0.308996<br>Thermal correction to Gibbs Free Energy= 0.208334
Coordinates	0 1<br>C 1.00800 -0.07608 -0.06441<br>O 0.96930 1.74066 -0.33766<br>O 1.17371 0.27553 -1.86616<br>O 1.00800 -0.07608 -0.06441<br>H 1.70224 0.87223 -0.89555<br>H 1.00800 -0.07608 -0.06441<br>C 3.60356 -0.15010 -1.58448<br>C 3.25236 -1.92420 0.50504<br>C 2.50969 -1.04581 -0.65075<br>H 3.50660 -0.43455 -2.64861<br>H 4.64151 -0.29984 -1.28840<br>H 2.89599 -1.66487 1.50166<br>H 4.32986 -1.77859 0.48683<br>C -0.07820 -1.58276 -1.08255<br>C -0.90855 -1.26572 -2.22734<br>C 0.21273 -2.98683 -1.01699<br>C -1.34699 -2.19996 -3.18249<br>H -1.11616 -0.22494 -2.46647<br>C -0.21085 -3.91607 -1.98634<br>H 0.96626 -3.37361 -0.34452<br>C -0.97598 -3.52771 -3.07468<br>H -1.89852 -1.84328 -4.04488<br>H -1.22782 -4.22959 -3.85936<br>Cl 0.32535 -5.56036 -1.89035<br>H 3.42755 0.93177 -1.60482<br>H 3.06257 -2.99200 0.38607<br>O 2.47657 -2.18708 -1.59838<br>H 3.33505 -2.26586 -2.02484<br>C 1.00800 -0.07608 -0.06441

	C 2.57108 0.96589 0.90012<br>C 0.21954 1.16399 1.48725<br>C 0.35774 -1.30866 1.48887<br>O -0.78817 0.64714 -0.61052<br>H -0.26275 1.37248 -1.05720<br>F 2.45268 1.16888 2.24922<br>F 3.03303 2.12385 0.32344<br>F 3.76135 0.25064 0.90783<br>F -0.97133 -1.11339 1.77041<br>F 0.46505 -2.64992 1.32649<br>F 1.08775 -1.13936 2.63587<br>F 0.14073 0.61902 2.74834<br>F 0.81693 2.38513 1.75336<br>F -1.10830 1.54349 1.29988

Compound	*m*CPBA + cyclohexane TS (axial hydrogen)<br>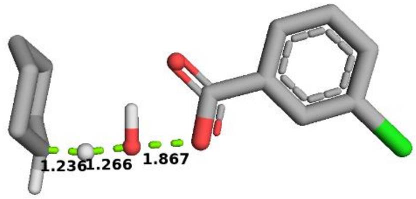
Functional/Basis set	B3LYP-GD3/6-311++G(d,p)
Solvation	SMD Chloroform
Imaginary Frequency (cm^{-1})	-701.29
E(RB3LYP) (A.U.)	-1191.67565993
Corrections	Zero-point correction= 0.272441 (Hartree/Particle)<br>Thermal correction to Energy= 0.288992<br>Thermal correction to Enthalpy= 0.289936<br>Thermal correction to Gibbs Free Energy= 0.225035
Coordinates	0 1<br>C -1.74706 0.79195 1.78880<br>O -2.40845 1.65607 1.11332<br>O -1.17400 1.02926 2.90604<br>O 1.05451 -0.08365 0.00557<br>H 0.59484 -0.59725 -0.95246<br>H 1.05451 -0.08365 0.00557<br>C 3.27150 -1.32099 1.16819<br>C 2.97693 -1.33120 2.66784<br>C 3.30628 -0.01677 3.36584<br>C 2.74427 1.19400 2.63127<br>C 3.18887 1.21714 1.17444<br>C 2.74203 -0.06600 0.41333<br>H 4.39411 0.08600 3.45076<br>H 1.92637 -1.57194 2.83455<br>H 3.54535 -2.14060 3.14202

	H 2.87308 -2.22835 0.70046<br>H 4.36121 -1.36444 1.03698<br>H 1.65025 1.18554 2.68742<br>H 3.07971 2.11061 3.12941<br>H 4.28412 1.30341 1.12666<br>H 2.78425 2.10489 0.67481<br>H 3.26271 -0.04688 -0.57651<br>H 2.90838 -0.03818 4.38754<br>C -1.84534 -0.65877 1.33734<br>C -0.87696 -1.61018 1.69871<br>C -2.88126 -1.07279 0.49241<br>C -0.89532 -2.91333 1.19005<br>H -0.01890 -1.25942 2.26632<br>C -2.93942 -2.38982 0.03729<br>H -3.61235 -0.33428 0.17937<br>C -1.94587 -3.31115 0.37094<br>H -0.05630 -3.57350 1.38640<br>H -1.96378 -4.31609 -0.03277<br>Cl -4.25757 -2.89108 -0.95695

Compound	*m*CPBA + cyclohexane TS (equatorial hydrogen)<br>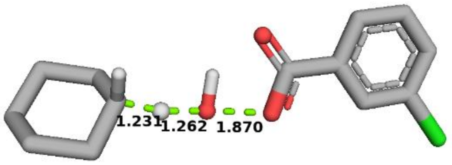
Functional/Basis set	B3LYP-GD3/6-311++G(d,p)
Solvation	SMD Chloroform
Imaginary Frequency (cm^{-1})	-664.21
E(RB3LYP) (A.U.)	-1191.67382667
Corrections	Zero-point correction= 0.272612 (Hartree/Particle)<br>Thermal correction to Energy= 0.289423<br>Thermal correction to Enthalpy= 0.290368<br>Thermal correction to Gibbs Free Energy= 0.223259
Coordinates	0 1<br>C 1.22883 0.90734 -2.73815<br>O 2.46931 1.09689 -2.56850<br>O 0.68932 -0.15629 -3.19358<br>O 0.42973 -0.91530 4.31527<br>H 0.53660 -0.76397 3.39712<br>C 1.27187 0.65785 0.90399<br>H 1.60167 1.19501 0.01496<br>H 1.75371 1.18522 1.73771<br>C -0.24204 0.80568 1.04271<br>H -0.50252 0.83390 2.10571<br>H -0.54536 1.78364 0.64919

	C 1.83201 -0.75941 0.82667<br>H 2.77618 -0.73264 0.26618<br>H 2.10392 -1.09179 1.83359<br>C 0.91888 -1.79888 0.19064<br>H 1.28789 -2.79973 0.44541<br>H 0.97676 -1.71858 -0.90037<br>C -0.53215 -1.67280 0.62608<br>C -1.06941 -0.27619 0.36308<br>H -1.10252 -0.09938 -0.71701<br>H -2.10482 -0.21084 0.71891<br>H -1.13935 -2.40392 0.07979<br>H -0.62932 -1.91778 1.68883<br>C 0.29977 2.06464 -2.43464<br>C 0.74066 3.18064 -1.69906<br>C -1.02469 2.05431 -2.90119<br>C -0.10120 4.26434 -1.45790<br>H 1.75213 3.17886 -1.29177<br>C -1.85271 3.15187 -2.68663<br>H -1.38277 1.17620 -3.43480<br>C -1.40141 4.25819 -1.97551<br>H 0.25181 5.10538 -0.87141<br>H -2.04792 5.11195 -1.81201<br>Cl -3.45062 3.15601 -3.33979

Compound	*m*CPBA + cyclohexanol TS (axial hydrogen)<br>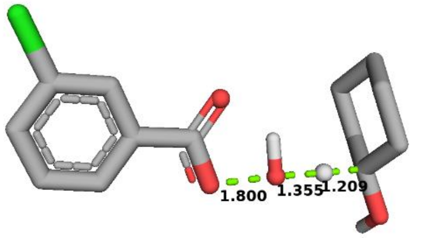
Functional/Basis set	B3LYP-GD3/6-311++G(d,p)
Solvation	SMD Chloroform
Imaginary Frequency (cm^{-1})	-651.53
E(RB3LYP) (A.U.)	-1266.93417843
Corrections	Zero-point correction= 0.278544 (Hartree/Particle)<br>Thermal correction to Energy= 0.296196<br>Thermal correction to Enthalpy= 0.297140<br>Thermal correction to Gibbs Free Energy= 0.229951
Coordinates	0 1<br>O 3.93510 0.91920 -0.18103<br>O 0.30677 -0.63597 -1.99601<br>C -0.26300 1.57395 1.50079<br>H 0.36196 1.41593 0.61376<br>C -0.01937 0.49298 2.54592<br>C -1.73487 1.59615 1.10999

	H -0.55351 0.74968 3.46971<br>H 1.03860 0.48261 2.82933<br>H -2.35354 1.81954 1.98851<br>H -1.92202 2.40975 0.39910<br>C 3.54387 -0.15578 0.38177<br>O 3.15133 -0.24059 1.59266<br>H 0.89018 -0.20969 -1.37433<br>O 0.08747 2.84265 2.06721<br>H 1.06563 2.82769 2.05168<br>C -2.15365 0.26499 0.50506<br>H -3.23071 0.27270 0.30176<br>H -1.66967 0.15514 -0.46744<br>C -1.81468 -0.93503 1.38772<br>C -0.44523 -0.88903 2.06790<br>H -0.45422 -1.57294 2.92631<br>H 0.31201 -1.27693 1.37803<br>H -2.58742 -1.02607 2.15940<br>H -1.87682 -1.85013 0.78448<br>C 3.73459 -1.45771 -0.36965<br>C 3.10573 -2.64211 0.05482<br>C 4.59915 -1.52643 -1.47169<br>C 3.39993 -3.86285 -0.55544<br>H 2.38195 -2.59126 0.86407<br>C 4.89912 -2.74567 -2.07947<br>H 5.03900 -0.60329 -1.84197<br>C 4.30956 -3.91823 -1.61036<br>H 5.59143 -2.77246 -2.91446<br>H 4.56336 -4.86706 -2.06482<br>Cl 2.67748 -5.31997 0.01169

Compound	*m*CPBA + cyclohexanol TS (equatorial hydrogen)<br>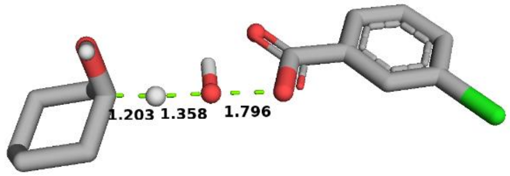
Functional/Basis set	B3LYP-GD3/6-311++G(d,p)
Solvation	SMD Chloroform
Imaginary Frequency (cm^{-1})	-658.59
E(RB3LYP) (A.U.)	-1266.93198393
Corrections	Zero-point correction= 0.278353 (Hartree/Particle)<br>Thermal correction to Energy= 0.296227<br>Thermal correction to Enthalpy= 0.297171<br>Thermal correction to Gibbs Free Energy= 0.228417
Coordinates	0 1<br>C 2.94873 -1.89859 0.89121

	O	3.49425	-2.50127	-0.09209
	O	3.36485	-0.81680	1.40712
	O	-1.18880	1.93368	3.14051
	H	-0.84620	1.16117	2.70966
	C	0.23188	2.04489	-0.82467
	H	0.70794	2.87222	-0.28598
	C	0.86319	0.72448	-0.39169
	H	0.81688	0.64905	0.69917
	H	1.92127	0.69489	-0.67706
	C	-1.27841	2.08411	-0.58783
	H	-1.71700	2.93256	-1.12634
	H	-1.46497	2.25572	0.47481
	C	-2.02078	0.81167	-1.00596
	H	-2.15524	0.81352	-2.09451
	H	-3.02516	0.82124	-0.56550
	C	-1.31234	-0.47384	-0.59791
	C	0.14433	-0.45738	-1.03449
	H	0.62593	-1.39751	-0.75909
	H	0.20717	-0.39094	-2.12710
	H	-1.37239	-0.59334	0.48945
	H	-1.82188	-1.33728	-1.03948
	O	0.48906	2.25654	-2.22264
	H	1.40159	1.94701	-2.36232
	C	1.71423	-2.55144	1.48872
	C	1.44875	-3.90393	1.21207
	C	0.78506	-1.83242	2.26498
	C	0.29199	-4.52644	1.68090
	H	2.15683	-4.46036	0.60388
	C	-0.38354	-2.45608	2.71260
	H	0.95166	-0.77601	2.47441
	C	-0.63068	-3.79704	2.42215
	H	0.11101	-5.56936	1.45127
	H	-1.54541	-4.26625	2.76575
	Cl	-1.56928	-1.59787	3.61615

Compound	*m*CPBA + cyclohexane TS (axial hydrogen)<br>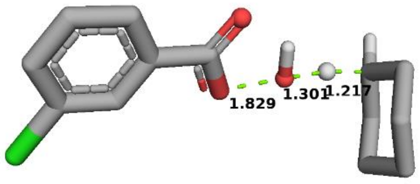
Functional/Basis set	B3LYP-GD3/6-311++G(d,p)
Solvation	SMD HFIP
Imaginary Frequency (cm^{-1})	-778.78
E(RB3LYP) (A.U.)	-1191.64210342
Corrections	Zero-point correction= 0.272176 (Hartree/Particle)

	Thermal correction to Energy= 0.288933<br>Thermal correction to Enthalpy= 0.289877<br>Thermal correction to Gibbs Free Energy= 0.223574
Coordinates	0 1<br>C 1.00294 0.02377 -0.09709<br>O -0.02115 1.46496 -0.10723<br>O 0.18026 -1.48261 0.33398<br>O 1.00294 0.02377 -0.09709<br>H 0.29510 -0.77893 -0.95746<br>H 1.00294 0.02377 -0.09709<br>C 2.69488 -1.07394 1.96962<br>C 2.07970 -1.16276 3.34005<br>C 2.42418 0.08801 4.12170<br>C 1.79358 1.31656 3.47276<br>C 2.30926 1.51165 2.06722<br>C 2.26225 0.22726 1.20516<br>H 2.05561 -0.00545 5.15295<br>H 0.99580 -1.28323 3.25046<br>H 2.46965 -2.05283 3.85291<br>H 3.79098 -1.06973 2.06548<br>H 2.47873 -1.97989 1.37763<br>H 2.03747 2.20682 4.05854<br>H 0.70378 1.22079 3.44462<br>H 3.37054 1.82640 2.14178<br>H 1.81642 2.34201 1.55648<br>H 3.21796 0.39355 0.68329<br>H 3.51073 0.20968 4.17892<br>C 2.56509 -0.04668 -1.39635<br>C 3.27597 -1.27212 -1.38492<br>C 3.22962 1.19342 -1.58872<br>C 4.67017 -1.23315 -1.32112<br>H 2.72464 -2.17557 -1.13315<br>C 4.62020 1.19746 -1.50156<br>H 2.64572 2.10251 -1.46844<br>C 5.33578 0.00001 -1.33650<br>H 5.21491 -2.14416 -1.09550<br>H 6.39946 0.03028 -1.11997<br>Cl 5.47108 2.69527 -1.36000

Compound	*m*CPBA + cyclohexane TS (equatorial hydrogen)<br>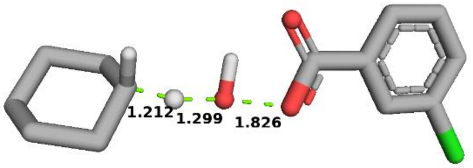
Functional/Basis set	B3LYP-GD3/6-311++G(d,p)

Solvation	SMD HFIP
Imaginary Frequency (cm^{-1})	-740.44
E(RB3LYP) (A.U.)	-1191.63958542
Corrections	Zero-point correction= 0.272047 (Hartree/Particle)<br>Thermal correction to Energy= 0.289084<br>Thermal correction to Enthalpy= 0.290029<br>Thermal correction to Gibbs Free Energy= 0.221746
Coordinates	0 1<br>C 1.09307 -0.02517 -0.02655<br>O 0.49172 -1.58643 -0.55795<br>O 0.40269 1.31697 -0.92258<br>O 1.09307 -0.02517 -0.02655<br>H 0.90340 -0.21494 -1.34917<br>C 0.43876 1.56305 2.32119<br>H 1.13454 1.95472 1.55992<br>H 0.45011 2.39629 3.03027<br>C -0.97073 1.41722 1.75404<br>H -1.66630 1.85939 2.46901<br>H -1.05992 2.06501 0.86879<br>C 1.05518 0.33306 3.00624<br>H 2.14358 0.35834 2.83462<br>H 0.93263 0.46265 4.07774<br>C 0.55236 -1.07003 2.64086<br>H 0.62254 -1.70402 3.53293<br>H 1.27033 -1.57153 1.98389<br>C -0.85493 -1.18635 2.07111<br>C -1.50376 0.02529 1.38509<br>H -1.48573 -0.10564 0.28945<br>H -2.57801 0.00666 1.57986<br>H -0.89367 -2.04536 1.37019<br>H -1.52652 -1.49040 2.88117<br>C 3.08596 0.02792 0.04710<br>C 3.80003 -1.19037 -0.03517<br>C 3.74792 1.26150 -0.11837<br>C 5.17399 -1.17830 -0.22553<br>H 3.21957 -2.10716 -0.02644<br>C 5.14199 1.25049 -0.29320<br>H 3.15735 2.16302 -0.16118<br>C 5.84760 0.04661 -0.35761<br>H 5.70816 -2.11798 -0.32501<br>H 6.92109 0.04569 -0.52879<br>Cl 5.99842 2.72838 -0.52972

Compound	*m*CPBA + cyclohexanol TS (axial hydrogen)

	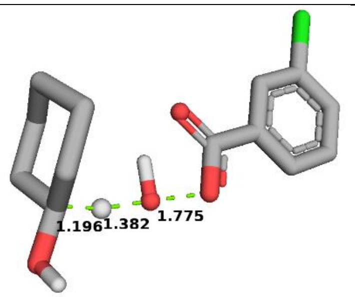
Functional/Basis set	B3LYP-GD3/6-311++G(d,p)
Solvation	SMD HFIP
Imaginary Frequency (cm^{-1})	-727.02
E(RB3LYP) (A.U.)	-1266.89714961
Corrections	Zero-point correction= 0.279841 (Hartree/Particle)<br>Thermal correction to Energy= 0.297259<br>Thermal correction to Enthalpy= 0.298203<br>Thermal correction to Gibbs Free Energy= 0.232673
Coordinates	0 1<br>O 2.38521 1.35821 2.64116<br>O 2.56768 0.49720 -3.62765<br>C 0.12162 0.99112 -0.19816<br>H 0.22870 1.23475 -1.26264<br>C 0.91654 -0.28231 0.11033<br>C -1.35921 0.85281 0.14322<br>H 1.02486 -0.39935 1.19520<br>H 1.93350 -0.18941 -0.28617<br>H -1.48021 0.86908 1.23536<br>H -1.90115 1.73384 -0.22316<br>C 3.48629 1.10801 2.04694<br>O 4.26363 0.13447 2.30586<br>H 2.60066 0.76275 -2.72523<br>O 0.58484 2.11206 0.55346<br>H 1.14581 1.77506 1.29393<br>C -1.98415 -0.41420 -0.41878<br>H -3.01826 -0.49657 -0.06776<br>H -2.01714 -0.35480 -1.51319<br>C -1.19399 -1.64620 0.00555<br>C 0.25821 -1.54018 -0.44838<br>H 0.81297 -2.42442 -0.11375<br>H 0.30291 -1.52928 -1.54408<br>H -1.23009 -1.74703 1.09796<br>H -1.65394 -2.54632 -0.41543<br>C 3.88568 2.06212 0.94657<br>C 3.39507 3.38155 0.93787<br>C 4.67687 1.64113 -0.13255<br>C 3.68973 4.24449 -0.11620<br>H 2.73096 3.70029 1.73723<br>C 4.97045 2.51046 -1.18863

	H 5.00751 0.60514 -0.18052<br>C 4.48238 3.81706 -1.17773<br>H 5.52579 2.14188 -2.04666<br>H 4.68437 4.47197 -2.01831<br>Cl 3.05462 5.84865 -0.11574

Compound	*m*CPBA + cyclohexanol TS (equatorial hydrogen)<br>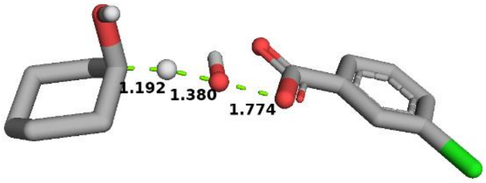
Functional/Basis set	B3LYP-GD3/6-311++G(d,p)
Solvation	SMD HFIP
Imaginary Frequency (cm^{-1})	-728.51
E(RB3LYP) (A.U.)	-1266.89349155
Corrections	Zero-point correction= 0.278958 (Hartree/Particle)<br>Thermal correction to Energy= 0.296742<br>Thermal correction to Enthalpy= 0.297687<br>Thermal correction to Gibbs Free Energy= 0.229339
Coordinates	0 1<br>C 1.03654 -0.08941 -0.02589<br>O 0.12300 -1.21476 -1.02744<br>O 2.69910 -0.44147 -0.49172<br>O 1.03654 -0.08941 -0.02589<br>H 1.44085 -0.72160 -1.14991<br>C 1.80157 2.00008 -0.25195<br>H 1.94442 3.08338 -0.15360<br>C 2.51089 1.00223 1.35549<br>H 2.80767 1.64692 2.19373<br>H 3.49470 0.56558 1.11175<br>C -0.10665 1.72659 -0.76680<br>H -0.09968 1.49524 -1.84036<br>H -0.63461 2.67748 -0.61279<br>C -1.22439 0.32607 0.10197<br>H -1.67597 -0.30498 -0.68259<br>H -2.15123 0.80027 0.46373<br>C -0.40325 -0.84920 1.50597<br>C 1.39051 -0.40005 2.20344<br>H 1.30353 -0.16563 3.26983<br>H 1.97442 -1.33489 2.10192<br>H -1.07380 -1.08623 2.34961<br>H -0.30417 -1.86375 1.07272<br>O 2.66294 1.78774 -1.40466<br>H 2.76296 0.79217 -1.30868<br>C 0.30463 1.37505 1.36971

	C -0.94799 1.44879 2.18168<br>C 0.79129 2.76104 1.70703<br>C -1.77033 2.59639 2.41253<br>H -1.52700 0.59642 2.50752<br>C 0.02071 3.93574 1.90082<br>H 1.83695 3.06028 1.67990<br>C -1.32237 3.84753 2.11082<br>H -2.79936 2.43401 2.71788<br>H -1.96846 4.71164 2.10968<br>Cl 0.76149 5.48790 1.75671

Compound	*m*CPBA + methylcyclohexane TS (equatorial hydrogen)<br>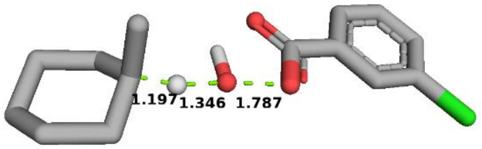
Functional/Basis set	B3LYP-GD3/6-311++G(d,p)
Solvation	SMD HFIP
Imaginary Frequency (cm^{-1})	-717.69
E(RB3LYP) (A.U.)	-1230.97666481
Corrections	Zero-point correction= 0.278958 (Hartree/Particle)<br>Thermal correction to Energy= 0.296742<br>Thermal correction to Enthalpy= 0.297687<br>Thermal correction to Gibbs Free Energy= 0.229339
Coordinates	0 1<br>C -0.15077 -0.17032 -3.17989<br>O 0.95416 -0.18844 -3.82598<br>O -1.00440 -1.12441 -3.17956<br>O 0.96075 0.09082 -0.01452<br>H 0.02185 0.54501 0.53711<br>H 0.96075 0.09082 -0.01452<br>C 3.14809 -1.16798 0.99356<br>C 2.55087 -1.19983 2.39863<br>C 2.88739 0.05985 3.19146<br>C 2.55351 1.35021 2.44701<br>C 3.13572 1.36493 1.03662<br>C 2.69266 0.10511 0.21242<br>H 2.34904 0.04143 4.14546<br>H 2.92267 -2.08161 2.93075<br>H 1.46111 -1.31472 2.33651<br>H 4.24317 -1.18671 1.06714<br>H 2.85289 -2.06926 0.43900<br>H 2.94127 2.20692 3.01064<br>H 1.46537 1.47822 2.40179<br>H 2.82986 2.28338 0.51847<br>H 4.23178 1.39596 1.10439

	H 3.95741 0.05261 3.42898<br>C -0.58799 1.15581 -2.57120<br>C -1.93775 1.39039 -2.26882<br>C 0.34288 2.16293 -2.24742<br>C -2.35641 2.59618 -1.70466<br>H -2.65272 0.59274 -2.45945<br>C -0.06718 3.34615 -1.63557<br>H 1.39978 1.94613 -2.37332<br>C -1.41829 3.57659 -1.38789<br>H -3.40566 2.74566 -1.48138<br>H -1.73738 4.49698 -0.91470<br>Cl 1.08655 4.53611 -1.15932<br>C 3.46413 0.13864 -1.14552<br>H 3.16013 -0.69217 -1.78954<br>H 4.54560 0.05459 -0.98164<br>H 3.29665 1.07734 -1.68221

Compound	*m*CPBA + methylcyclohexane TS (axial hydrogen)<br>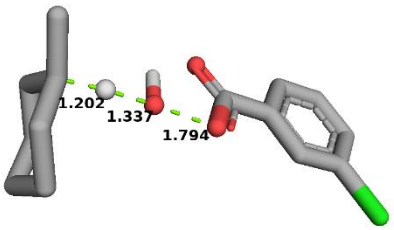
Functional/Basis set	B3LYP-GD3/6-311++G(d,p)
Solvation	SMD HFIP
Imaginary Frequency (cm^{-1})	-711.47
E(RB3LYP) (A.U.)	-1230.97242967
Corrections	Zero-point correction= 0.300781 (Hartree/Particle)<br>Thermal correction to Energy= 0.319139<br>Thermal correction to Enthalpy= 0.320083<br>Thermal correction to Gibbs Free Energy= 0.249115
Coordinates	0 1<br>C 1.04163 0.08928 -0.01719<br>O 0.15268 1.19984 -1.06551<br>O 2.61513 0.18116 -0.82175<br>O 1.04163 0.08928 -0.01719<br>H 1.25309 0.26563 -1.34050<br>C 1.59849 1.21035 1.71732<br>H 1.77652 1.56576 2.73243<br>C 2.64131 -0.47966 1.37995<br>H 2.79899 -0.88098 2.38829<br>H 3.65377 -0.18746 1.04985<br>C -0.35845 1.15460 1.32687<br>H -0.60683 2.12700 0.85963<br>H -0.83431 1.21982 2.30891

	C -1.13915 -0.26029 0.08487<br>H -1.55424 0.21730 -0.81221<br>H -2.12432 -0.61347 0.47178<br>C 0.02746 -1.77398 -0.53455<br>C 1.97228 -1.88073 0.04672<br>H 2.25170 -2.91219 0.30305<br>H 2.52904 -1.83175 -0.88745<br>H -0.49470 -2.73146 -0.42310<br>H 0.07762 -1.61854 -1.62162<br>C 0.40462 -1.10433 1.62799<br>C 0.95692 -2.40342 2.06699<br>C -0.92838 -1.24658 2.21999<br>C 0.26550 -3.65066 1.94105<br>H 2.02772 -2.57661 2.16668<br>C -1.69586 -2.45873 2.14396<br>H -1.57016 -0.40419 2.43928<br>C -1.09827 -3.65661 1.84007<br>H 0.83838 -4.55514 1.74844<br>H -1.65455 -4.53121 1.52885<br>Cl -3.42838 -2.40528 2.20729<br>C 2.15581 2.48576 0.70705<br>H 3.21093 2.73569 0.84290<br>H 1.53012 3.38259 0.81158<br>H 2.09501 2.31426 -0.35740

Compound	*m*CPBA + methylcyclohexane TS (equatorial hydrogen)<br>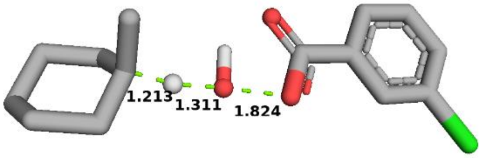
Functional/Basis set	B3LYP-GD3/6-311++G(d,p)
Solvation	SMD Chloroform
Imaginary Frequency (cm^{-1})	-668.74
E(RB3LYP) (A.U.)	-1231.01035700
Corrections	Zero-point correction= 0.300863 (Hartree/Particle)<br>Thermal correction to Energy= 0.318889<br>Thermal correction to Enthalpy= 0.319833<br>Thermal correction to Gibbs Free Energy= 0.252574
Coordinates	0 1<br>C 0.96706 0.09377 -0.01110<br>O 0.59798 -1.33925 -0.98294<br>O 0.58322 -0.14871 1.70143<br>O 0.96706 0.09377 -0.01110<br>H 0.53726 -1.09210 0.53600<br>H 0.96706 0.09377 -0.01110

	C	3.38262	-1.24506	0.70794
	C	3.16213	-1.28402	2.22243
	C	3.45918	0.02406	2.95971
	C	3.19526	1.32735	2.20126
	C	3.50547	1.24160	0.71212
	C	2.77982	0.03911	-0.00355
	H	2.89058	0.04278	3.89909
	H	2.14297	-1.61559	2.44026
	H	3.78775	-2.07592	2.65311
	H	2.97499	-2.15377	0.25204
	H	4.46615	-1.27702	0.52873
	H	2.15928	1.64827	2.35156
	H	3.80470	2.12258	2.64810
	H	4.59299	1.12416	0.60159
	H	3.27241	2.18585	0.21148
	H	4.51522	0.01406	3.25266
	C	0.06207	1.79235	-0.61942
	C	0.50284	3.03632	-0.13226
	C	-1.19806	1.66007	-1.23636
	C	-0.28339	4.16934	-0.32732
	H	1.40655	3.07758	0.45678
	C	-1.98534	2.79465	-1.38545
	H	-1.54014	0.65660	-1.47361
	C	-1.52780	4.04796	-0.95231
	H	0.04960	5.12341	0.06494
	H	-2.15936	4.92382	-1.05112
	Cl	-3.57721	2.67243	-2.04224
	C	3.32809	0.03281	-1.49049
	H	4.42252	0.01215	-1.51398
	H	2.96772	-0.84641	-2.03467
	H	2.99567	0.93134	-2.02182

Compound	*m*CPBA + methylcyclohexane TS (axial hydrogen)<br>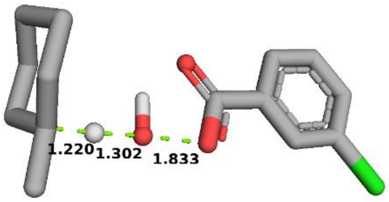
Functional/Basis set	B3LYP-GD3/6-311++G(d,p)
Solvation	SMD Chloroform
Imaginary Frequency (cm^{-1})	-660.96
E(RB3LYP) (A.U.)	-1231.00708560
Corrections	Zero-point correction= 0.301072 (Hartree/Particle)<br>Thermal correction to Energy= 0.319282<br>Thermal correction to Enthalpy= 0.320226<br>Thermal correction to Gibbs Free Energy= 0.250567

Coordinates	0 1<br>C 1.00081 -0.09941 -0.01082<br>O 0.35395 1.18166 -1.02146<br>O 0.34632 -1.67242 -0.43346<br>O 1.00081 -0.09941 -0.01082<br>H 0.81073 -0.34624 -1.32411<br>C 0.39419 1.81271 2.01330<br>H 0.97162 1.70555 1.11407<br>C -1.08192 1.61659 1.62037<br>H -1.67746 2.08429 2.42662<br>H -1.29744 2.21525 0.72838<br>C 0.90971 0.70691 2.98083<br>H 2.01428 0.68698 2.90656<br>H 0.68268 1.01267 3.99771<br>C 0.41903 -0.72555 2.78725<br>H 0.45404 -1.26904 3.73418<br>H 1.16163 -1.30630 2.20247<br>C -0.97753 -0.94915 2.18450<br>C -1.61954 0.19827 1.35908<br>H -1.58897 -0.04101 0.29022<br>H -2.70364 0.17707 1.54020<br>H -0.96281 -1.85830 1.54233<br>H -1.67205 -1.23764 2.97257<br>C 3.00129 -0.12121 0.06315<br>C 3.64902 -1.36774 -0.04248<br>C 3.71439 1.08313 -0.05392<br>C 5.02957 -1.40979 -0.25492<br>H 3.02459 -2.25811 -0.06360<br>C 5.09130 1.01583 -0.24487<br>H 3.15974 2.01296 -0.06550<br>C 5.74592 -0.21850 -0.34339<br>H 5.51494 -2.36963 -0.39254<br>H 6.81922 -0.25015 -0.52962<br>Cl 6.01065 2.46715 -0.41669<br>C 0.87761 3.24788 2.16275<br>H 1.89886 3.34837 1.76136<br>H 0.88374 3.59659 3.19822<br>H 0.26887 3.91480 1.53910

2 General experimental details.

Nuclear magnetic resonance (NMR) spectra were recorded on a Bruker Avance III 400 (^{1}H 400 MHz and ^{13}C 101 MHz), Bruker Avance 400 (^{1}H 400 MHz and ^{13}C 101 MHz), Bruker Avance III HD NanoBay (^{1}H 400 MHz and ^{13}C 101 MHz) or Bruker Avance DRX 500 (^{1}H 500 MHz and ^{13}C 125 MHz) spectrometer. NMR spectra were recorded at 25 °C. Chemical shifts are given in parts per million (ppm) referenced to residual protium or carbon of the solvents. Data for ^{1}H NMR spectra are reported as follows: chemical shift, multiplicity, coupling constant, and integration. Coupling constants (*J* values) are reported in Hertz (Hz) and multiplicities are expressed according to the usual conventions. Low-resolution mass spectra (LRMS) were determined on an Agilent 6130 single quadrupole with an APCI/electrospray dual source or ThermoQuest Finnigan LCQ DUO electrospray. GC-MS was performed using an Agilent 7890A GC system, equipped with a 30 m DB5MS column connected to a 5975C inert XL CI MSD with Triple-Axis Detector. Infrared spectra were determined on neat samples and were recorded in the range 4000–600 cm^{-1} on a Shimadzu IRAffinity-1 equipped with an ATR (Attenuated Total Reflectance) accessory. Measured relative intensities are abbreviated as follows: s = strong, med = medium, wk = weak, br = broad. Melting points were determined on a Stuart SMP11 and are uncorrected. Flash chromatography was carried out using head pressure by means of compressed air and Merck Kieselgel 60 H silica. The solvents used for chromatography were not purified. When required, organic solvents were removed under reduced pressure. Evaporation was affected at about 20 mmHg using Büchi rotary evaporators and water bath, followed to evaporation to dryness under high vacuum (4 mbar). Analytical thin-layer chromatography (TLC) was carried out using 0.2 mm commercial silica gel plates (silica gel 60, F254, EMD chemical), and visualised under UV light (at 254 nm) or by staining with a solution of 2% aqueous potassium permanganate followed by gentle heating. Single-crystal X-ray diffraction data were measured on Oxford Diffraction Xcalibur E and Gemini S instruments. The structures were refined to convergence on F2 and against all independent reflections by full-matrix least-squares, using the SHELXL-97 program. Selected parameters are given in Appendix. All glassware used in anhydrous reactions was either oven-dried (100 °C) or flame dried under vacuum and cooled under a stream of nitrogen or argon prior to use.

2.1 Procedure for purification of *m*CPBA 5

In a 250 mL separating funnel commercial *m*CPBA **5** (10.0 g, 68%) was dissolved in diethyl ether (150 mL). The organic phase was washed with a solution of sodium phosphate buffer pH = 7.4 0.1 M (5 × 100 mL). The organic phase was separated, dried over magnesium sulphate, filtered and concentrated *in vacuo* to afford *m*CPBA **5** as white solid (5.98 g, 88%, 91–94% active oxygen).

In a conical flask commercial *m*CPBA **5** (10.0 g, 68%) was dissolved in dichloromethane (100 mL), passed through a hydrophobic frit, the solution was cooled to 0 °C and concentrated while stirring under a stream of nitrogen. After reducing volume by half, the precipitate was filtered off and dried under air to afford *m*CPBA **5** as white solid (5.03 g, 74%, >99% active oxygen).

2.2 Oxidation of 2° sp3 C–H bonds.

2.2.1 Typical experimental procedure for oxidation of cyclic alkanes.

In a 0.5-2.5 mL μwave vial 86 mg (0.5 mmol) of 100% or 92 mg (0.5 mmol) of 90-93% *m*CPBA **5** were suspended in 0.5 mL of nonafluoro-*tert*-butanol followed by addition of substrate (2.5 mmol, 5.0 eq). The reaction vial was sealed, thermally incubated and stirred for the desired time. The reaction was cooled in air to ambient temperature, followed by addition of 1 mL of 21 mg/mL (0.125 mmol 0.25 eq) solution of 1,4-dinitrobenzene in $CDCl_3$. The reaction mixture was homogenised by addition of 4 mL of $CDCl_3$ and analysed by ^{1}H, ^{13}C and ^{19}F NMR spectroscopy. Reaction products were compared to literature values of authentic compounds.

2.2.2 Typical experimental procedure for oxidation of acyclic alkanes.

In a 0.5-2.5 mL μwave vial 86 mg (0.5 mmol) of 100% or 92 mg (0.5 mmol) of 93% *m*CPBA **5** were suspended in 0.5 mL of nonafluoro-*tert*-butanol followed by addition of substrate (2.5 mmol, 5.0 eq). The reaction vial was sealed, thermally incubated and stirred for the desired time. The reaction was cooled in air to ambient temperature, followed by addition of 1 mL of 21 mg/mL (0.125 mmol 0.25 eq) solution of 1,4-dinitrobenzene in $CDCl_3$. The reaction mixture was homogenised by addition of 4 mL of $CDCl_3$ and analysed by ^{1}H, ^{13}C and ^{19}F NMR spectroscopy. Reaction products were compared to literature values of authentic compounds.

2.3 Oxidation of 3° sp3 C–H bonds.

2.3.1 Typical experimental procedure for oxidation of cyclic alkanes.

In a 0.5-2.5 mL μwave vial 129 mg (0.75 mmol, 1.5 eq) of 100% or 138 mg (0.75 mmol, 1.5 eq) of 93% *m*CPBA **5** were suspended in 0.5 mL of nonafluoro-*tert*-butanol followed by addition of substrate (0.5 mmol, 1.0 eq). The reaction vial was sealed, thermally incubated and stirred for the desired time. The reaction was cooled in air to ambient temperature, followed by addition of 1 mL of 21 mg/mL (0.125 mmol 0.25 eq) solution of 1,4-dinitrobenzene in $CDCl_3$. The reaction mixture was homogenised by addition of 4 mL of $CDCl_3$ and analysed by ^{1}H, ^{13}C and ^{19}F NMR spectroscopy. Reaction products were compared to literature values of authentic compounds.

2.3.2 Typical experimental procedure for oxidation of acyclic alkanes.

In a 0.5-2.5 mL μwave vial 129 mg (0.75 mmol, 1.5 eq) of 100% or 138 mg (0.75 mmol, 1.5 eq) of 93% *m*CPBA **5** were suspended in 0.5 mL of nonafluoro-*tert*-butanol followed by addition of substrate (0.5 mmol, 1.0 eq). The reaction vial was sealed, thermally incubated and stirred for the desired time. The reaction was cooled down, followed by addition of 1 mL of 21 mg/mL (0.125 mmol 0.25 eq) solution of 1,4-dinitrobenzene in $CDCl_3$. The reaction mixture was homogenised by addition of 4 mL of $CDCl_3$ and analysed by ^{1}H ^{13}C and ^{19}F NMR. Reaction products were compared towards literature reported compounds.

2.4 Typical experimental procedure for oxidation of functionalised molecules.

In a 0.5-2.5 mL µwave vial 129 mg (0.75 mmol, 1.5 eq) of 100% or 138 mg (0.75 mmol, 1.5 eq) of 93% *m*CPBA **5** were suspended in 0.5 mL of nonafluoro-*tert*-butanol followed by addition of substrate (0.5 mmol, 1.0 eq). The reaction vial was sealed, thermally incubated and stirred for the desired time. The reaction was cooled to ambient temperature, followed by the work-up and isolation described for individual compounds.

2.5 Compound characterisation.

1-(((5-Methylhexyl)oxy)methyl)-4-nitrobenzene 62.

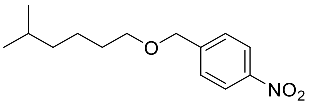

62

In a 20 mL microwave vial 5-methylhexano-1-ol (1.00 g, 8.6 mmol) was dissolved in DCM (17 mL), followed by addition of 1-(bromomethyl)-4-nitrobenzene (2.23 g 10.4 mmol, 1.2 eq) and silver(I) oxide (2.19 g, 9.5 mmol, 1.1 eq). The reaction vial was sealed and stirred for 18 hours at 65 °C. The reaction was filtered, concentrated *in vacuo* and purified by flash column chromatography to obtain the *title compound* **62** as a pale-yellow oil (632 mg, 29%). **IR (neat)/cm^{-1}** 2951 (med), 2933 (med), 2864 (med) 1604 (med) 1519 (s), 1342 (s) 1101 (s); **^{1}H NMR (400 MHz, $CDCl_3$)**: δ (ppm) 8.26 – 8.16 (m, 2H), 7.53 – 7.47 (m, 2H), 4.60 (s, 2H), 3.52 (t, *J* = 6.6 Hz, 2H), 1.70 – 1.57 (m, 2H), 1.57 – 1.49 (m, 1H), 1.44 – 1.33 (m, 2H), 1.23 – 1.16 (m, 2H), 0.88 (d, *J* = 6.7 Hz, 6H).**^{13}C NMR (101 MHz, $CDCl_3$)**: δ (ppm) 147.5, 146.6, 127.8, 123.8, 71.8, 71.4, 38.9, 30.1, 28.1, 24.1, 22.7; **LRMS (GC-MS-EI)** *m/z* calc. for $C_{14}H_{21}NO_3$ 251.2, found 252.2 $[M+H]^+$.

5-Methylhexyl 4-nitrobenzoate 63.

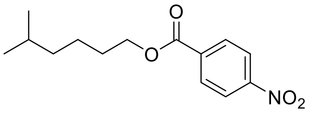

63

In a 100 mL round bottom flask 5-methylhexan-1-ol (1.16 g 10.0 mmol) was dissolved in anhydrous DCM (100 mL) followed by the addition of triethylamine (2.79 mL, 20.0 mmol, 2.0 eq). 4-Nitrobenzoyl chloride (2.41 g, 13.0 mmol, 1.3 eq) was added in 3 portions to the reaction mixture. The reaction mixture was stirred for 2 hours. The reaction mixture was washed with saturated sodium bicarbonate solution 3 x 20 mL followed by passing through a hydrophobic frit. The crude reaction mixture was purified by flash column chromatography to afford the *title compound* **63** as a pale-yellow oil (962 mg 36.3%). **IR (neat)/cm^{-1}** 2953 (med) 2935 (med) 1722 (s) 1608 (med) 1525 (s) 1269 (s); **^{1}H NMR (400 MHz, $CDCl_3$)**: δ (ppm) 8.31–8.26 (m, 2H), 8.24–8.17 (m, 2H), 4.37 (t, *J* = 6.7 Hz, 2H), 1.83–1.73 (m, 2H), 1.63–1.51 (m, 1H), 1.50–1.39 (m, 2H), 1.30–1.21 (m, 2H), 0.89 (d, *J* = 6.6 Hz, 6H); **^{13}C NMR (101 MHz, $CDCl_3$)**: δ (ppm) 164.9, 150.7, 136.1, 130.8, 123.7, 66.3, 38.6, 29.0, 28.0, 23.9, 22.7.; **LRMS (GC-MS-EI)** *m/z* calc. for $C_{14}H_{19}NO_4$ 265.1, found 265.0 $[M]^+$.

5-Hydroxy-5-methylhexanoic acid 66

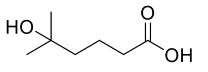

66

In a 0.5-2 mL microwave vial 5-methylhexanoic acid (0.130 g, 1.0 mmol) was dissolved in nonafluoro-*tert*-butanol (2 mL), followed by addition of *m*CPBA **5** (0.259 g, 1.5 mmol, 1.5 eq). The reaction vial was sealed and stirred for 48 hours at 65 °C. The crude reaction mixture was purified by flash column chromatography to afford the *title compound* **66** as a clear oil (89 mg, 62% with 7 mol% *m*CBA). **IR (neat)/cm^{-1}** 3454 (br wk), 2976 (med), 1718 (s), 1278 (s) 1211 (s) 1105 (s); **^{1}H NMR (400 MHz, CDCl$_3$)**: δ (ppm) 2.48 (t, *J* = 6.9 Hz, 2H), 1.93 – 1.84 (m, 2H), 1.77 – 1.72 (m, 2H), 1.40 (s, 6H); **^{13}C NMR (101 MHz, CDCl$_3$)**: δ (ppm) 171.4, 82.3, 34.1, 29.2, 28.9, 17.0; **LRMS (GC-MS-EI)** *m/z* calc. for $C_7H_{14}O_3$ 146.1, found 128.1 $[M-H_2O]^+$.

2-Methyl-6-((4-nitrobenzyl)oxy)hexan-2-ol 67

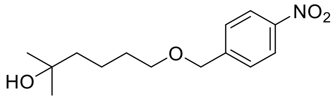

67

In a 0.5-2 mL microwave vial 1-(((5-methylhexyl)oxy)methyl)-4-nitrobenzene (0.251 g, 1.0 mmol) was dissolved in nonafluoro-*tert*-butanol (2 mL), followed by addition of *m*CPBA (0.259 g, 1.5 mmol, 1.5 eq). The reaction vial was sealed and stirred for 48 hours at 65 °C. The reaction mixture was cooled down, dissolved in DCM (20 mL), concentrated *in vacuo* and dissolved in diethyl ether (100 mL). The organic phase was washed with saturated solution of sodium bicarbonate (3 x 30 mL) passed through a phase separating filter paper and concentrated *in vacuo*. The crude product was purified by flash column chromatography to afford the *title compound* **67** as a pale-yellow oil (110 mg, 42%). **IR (neat)/cm^{-1}** 3402 (br wk), 2964 (med), 2939 (med), 2864 (med), 1604 (med), 1517 (s) 1342 (s); **^{1}H NMR (400 MHz, CDCl$_3$)**: 8.23–8.18 (m, 2H), 7.53–7.48 (m, 2H), 4.60 (s, 2H), 3.54 (t, *J* = 6.5 Hz, 2H), 1.71 – 1.62 (m, 2H), 1.56 – 1.39 (m, 4H), 1.22 (s, 6H); **^{13}C NMR (101 MHz, CDCl$_3$)**: δ (ppm) 147.5, 146.5, 127.8, 123.8, 71.8, 71.2, 71.1, 43.8, 30.3, 29.4, 21.2; **LRMS (GC-MS-EI)** *m/z* calc. for $C_{14}H_{21}NO_4$ 267.1, found 267.1 $[M]^+$.

5-Hydroxy-5-methylhexyl 4-nitrobenzoate 68.

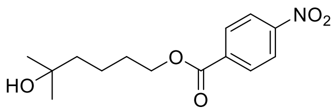

68

In a 0.5-2 mL microwave vial 5-methylhexyl 4-nitrobenzoate (0.265 g, 1.0 mmol) was dissolved in nonafluoro-*tert*-butanol (2 mL), followed by addition of *m*CPBA (0.259 g, 1.5 mmol, 1.5 eq). The reaction vial was sealed and stirred for 48 hours at 65 °C. The reaction mixture was cooled to ambient temperature, dissolved in DCM (20 mL), concentrated *in vacuo* and re-dissolved in diethyl ether (100 mL). The organic phase was washed with saturated solution of sodium bicarbonate (3 x 30 mL) passed through a phase separating filter paper and concentrated *in vacuo*. The crude product was purified by flash column chromatography to afford the *title compound* **68** as a pale-yellow solid (180 mg, 64%). **M.p.** = 62–64 °C; **IR (neat)/cm^{-1}** 3564 (med) 2970 (med), 956 (med) 2935 (med) 1708 (s) 1521 (s) 1276 (s); **^{1}H NMR (400**

MHz, $CDCl_3$): δ (ppm) 8.32 – 8.25 (m, 2H), 8.24 – 8.18 (m, 2H), 4.40 (t, *J* = 6.7 Hz, 2H), 1.87 – 1.78 (m, 2H), 1.58 – 1.50 (m, 4H), 1.24 (s, 6H); **^{13}C NMR (101 MHz, $CDCl_3$)**: δ (ppm) 164.9, 150.7, 136.0, 130.8, 123.7, 71.0, 66.0, 43.5, 29.5, 29.3, 20.9; **LRMS (GC-MS-EI)** *m/z* calc. for $C_{14}H_{19}NO_5$ 281.1, found 281.1 $[M]^+$.

3 Kinetic investigation of the transformation.

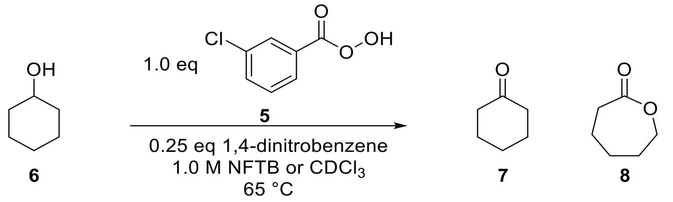

Figure S4. Oxidation of cyclohexanol **6** with *m*CPBA **5** in NFTB or $CDCl_3$.

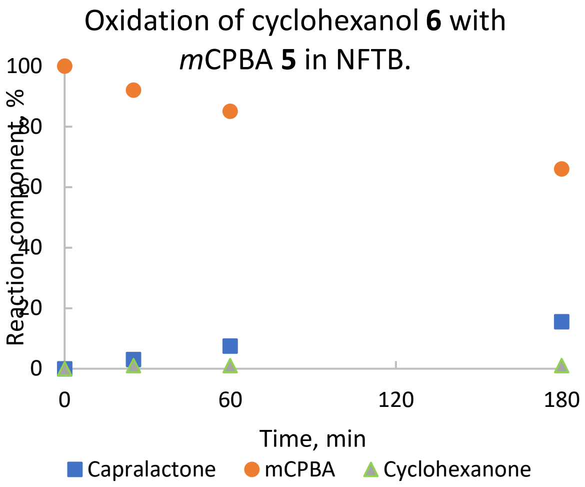

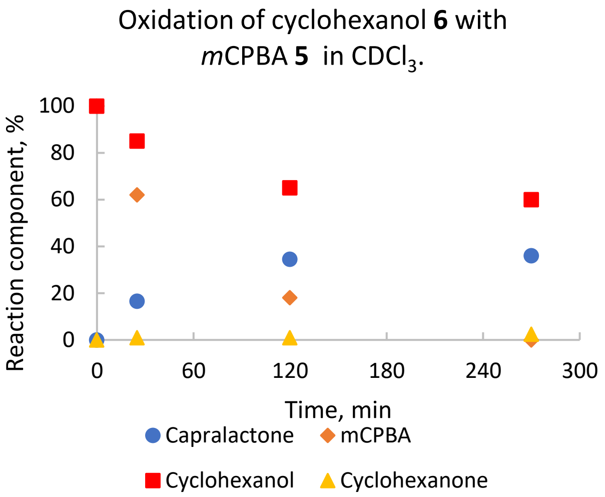

Oxidation of cyclohexanone **7** to caprolactone **8** was rapid (30 minutes) in both NFTB and $CDCl_3$ indicating an insignificant impact of the solvent on the reaction rate of the final step of the oxidation sequence. Monitoring reaction progress provided an explanation as why no meaningful accumulation of cyclohexanone **7** was observed in the reaction mixture in both NFTB and $CDCl_3$ (Figure S2).

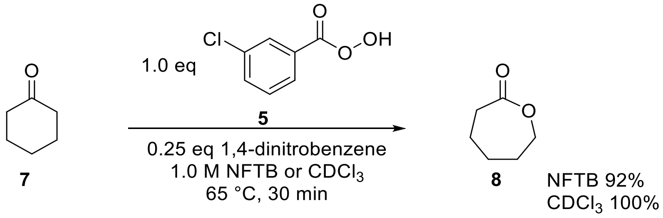

Figure S5. Oxidation of cyclohexanone **7** with mCPBA **5** in NFTB or $CDCl_3$.

3.1 Determination of reactant orders.

In an NMR tube fitted with a Youngs tap, 0.70 mL 0.357 M (43.14 mg, 1.0 eq) solution of 100% *meta*-chloroperbenzoic acid **5** in NFTB was placed in an NMR instrument and the temperature allowed to equilibrate. 0.30 mL of 0.208 M (10.5 mg, 0.25 eq) solution of 1,4-dinitrobenzene in 4.16 M (105.2 mg, 5.0 eq) solution of cyclohexane in NFTB were added to the reaction mixture. The reaction was conducted at 60 °C The time of addition was recorded and a ^{1}H NMR spectra was obtained every 5 minutes. The reaction exhibited as a pseudo first order with respect to *m*CPBA (see ln[*m*CPBA] *versus* time).

Oxidation of cyclohexane **4** with mCPBA in NFTB.

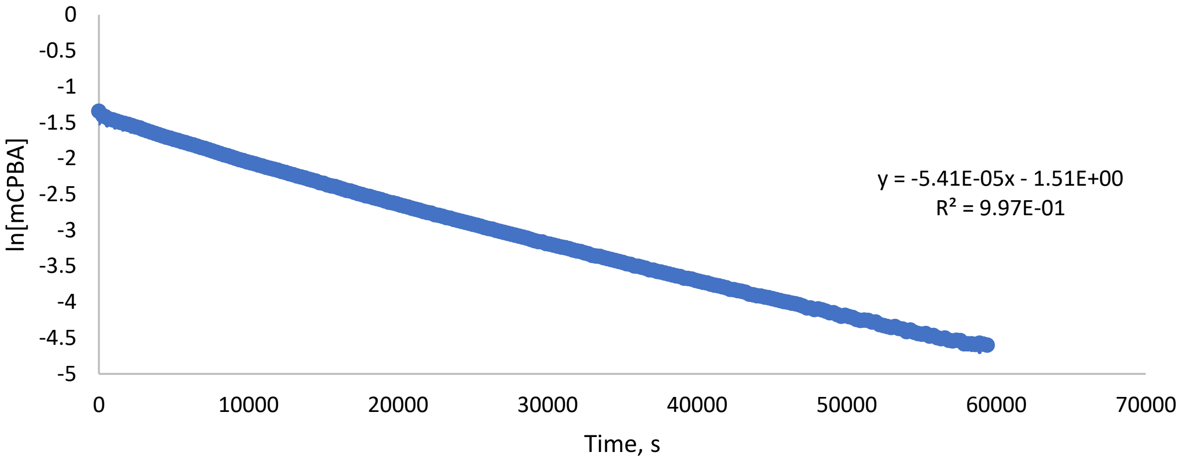

In an NMR tube fitted with a Youngs tap, 0.40 mL 0.0625 M (4.31 mg, 1.0 eq) solution of 100% *meta*-chloroperbenzoic acid **5** in NFTB was placed in an NMR instrument and the temperature allowed to equilibrate. 0.60 mL of 0.0104 M (1.06 mg, 0.25 eq) solution of 1,4-dinitrobenzene in 0.832 M (42.0 mg, 20.0 eq) solution of cyclohexane in NFTB were added to the reaction mixture. The time of addition was recorded and a ^{1}H NMR spectra was obtained every 3 minutes until consumption of 10% of the cyclohexanol was observed. The reactions were conducted at 50 °C. Observed rate constants were obtained using initial reaction rates by plotting [CyOH] *versus* time. The observed rate constant was twice the amount than observed at 50 °C in section 3.2 indicating 1st order in cyclohexane.

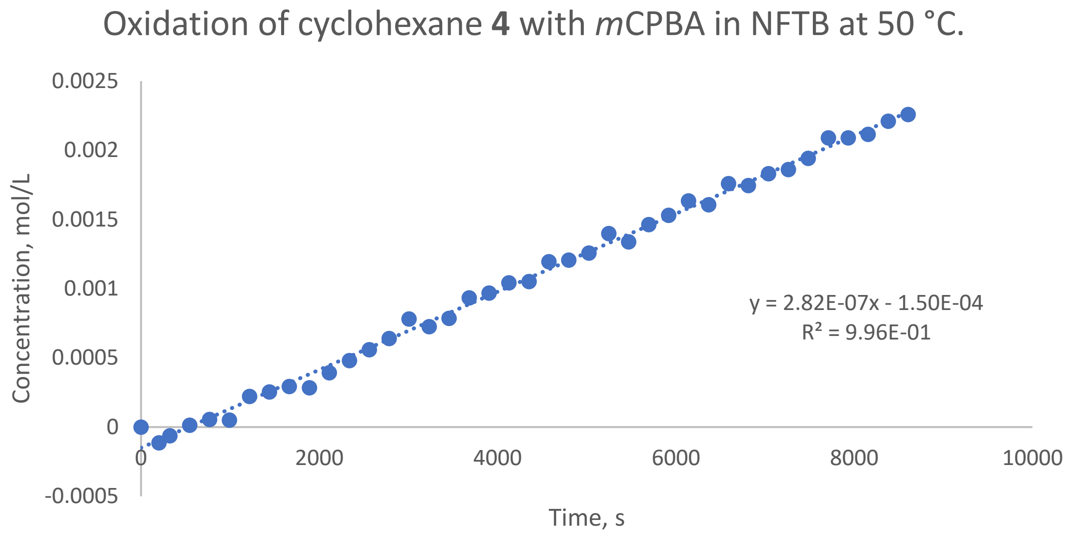

In an NMR tube fitted with a Youngs tap, 0.80 mL 0.3125 M (43.1 mg, 10.0 eq) solution of 100% *meta*-chloroperbenzoic acid in $CDCl_3$ or NFTB was placed in an NMR instrument and the temperature allowed to equilibrate. 0.20 mL of 0.03125 M (1.06 mg, 0.25 eq) solution of 1,4-dinitrobenzene in 0.125 M (2.50 mg, 1.0 eq) solution of cyclohexanol in $CDCl_3$ or NFTB were added to the reaction mixture. The time of addition was recorded and a ^{1}H NMR spectra was obtained every 3 minutes The reactions were conducted at 60 °C. The reactions exhibited as a pseudo first order with respect to cyclohexanol (see ln[CyOH] *versus* time).

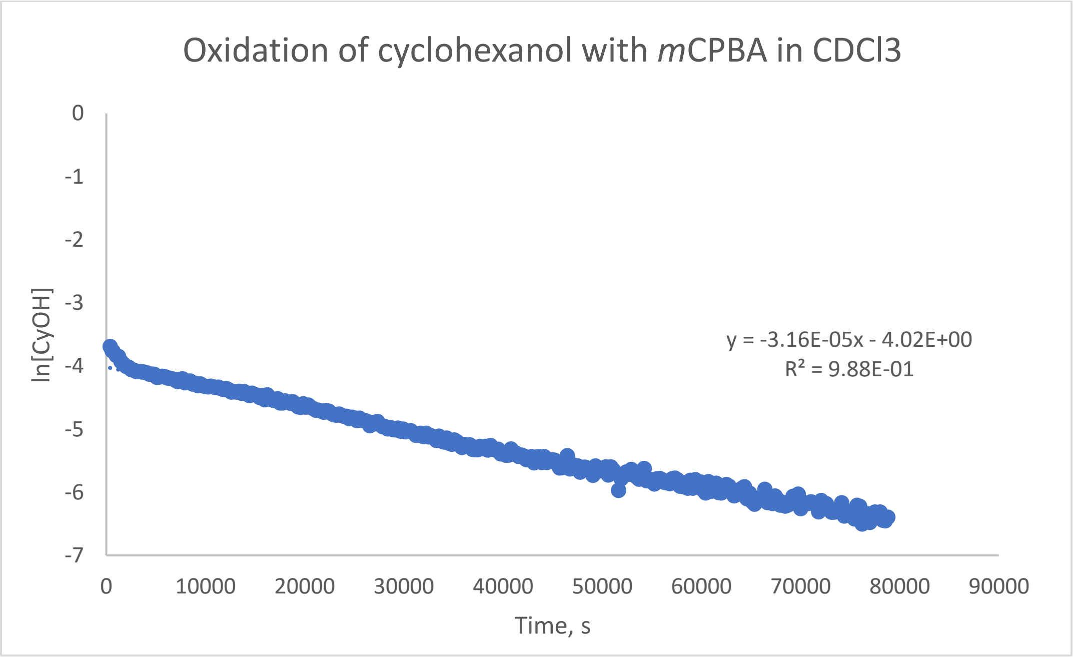

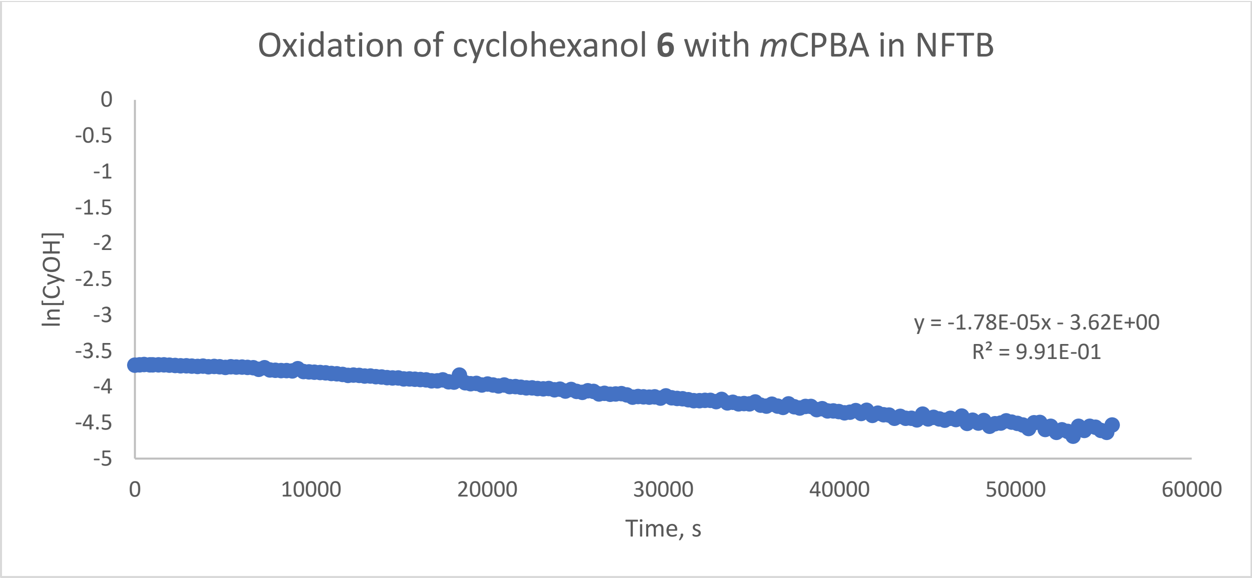
Oxidation of cyclohexanol 6 with mCPBA in NFTB
ln[CyOH]
0
-0.5
-1
-1.5
-2
-2.5
-3
-3.5
-4
-4.5
-5
0
10000
20000
30000
40000
50000
60000
Time, s
y = -1.78E-05x - 3.62E+00
R² = 9.91E-01

3.2 Kinetic investigation of oxidation of cyclohexane 4 in NFTB.

In an NMR tube fitted with a Youngs tap, 0.40 mL 0.0625 M (4.31 mg, 1.0 eq) solution of 100% *meta*-chloroperbenzoic acid **5** in NFTB was placed in an NMR instrument and the temperature allowed to equilibrate. 0.60 mL of 0.0104 M (1.06 mg, 0.25 eq) solution of 1,4-dinitrobenzene in 0.416 M (21.0 mg, 10.0 eq) solution of cyclohexane in NFTB were added to the reaction mixture. The time of addition was recorded and a ^{1}H NMR spectra was obtained every 3 to 10 minutes until consumption of 10% of the cyclohexanol was observed. The reactions were conducted in duplicate at 30, 40, 50 and 60 °C. Observed rate constants were obtained using initial reaction rates by plotting [CyOH] *versus* time. The observed rate constant was divided by $12[CyH]_0[m\text{CPBA}]_0$.

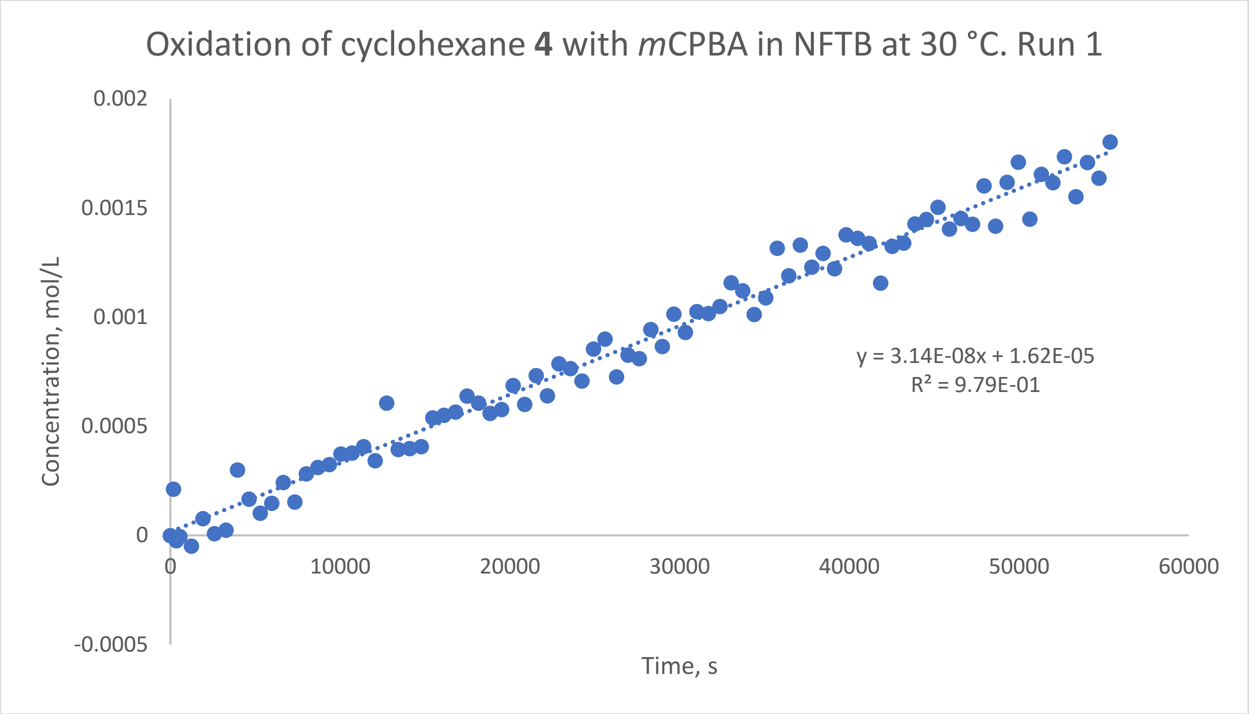
Oxidation of cyclohexane **4** with *m*CPBA in NFTB at 30 °C. Run 1
Concentration, mol/L
0.002
0.0015
0.001
0.0005
0
-0.0005
0
10000
20000
30000
40000
50000
60000
Time, s
y = 3.14E-08x + 1.62E-05
R² = 9.79E-01

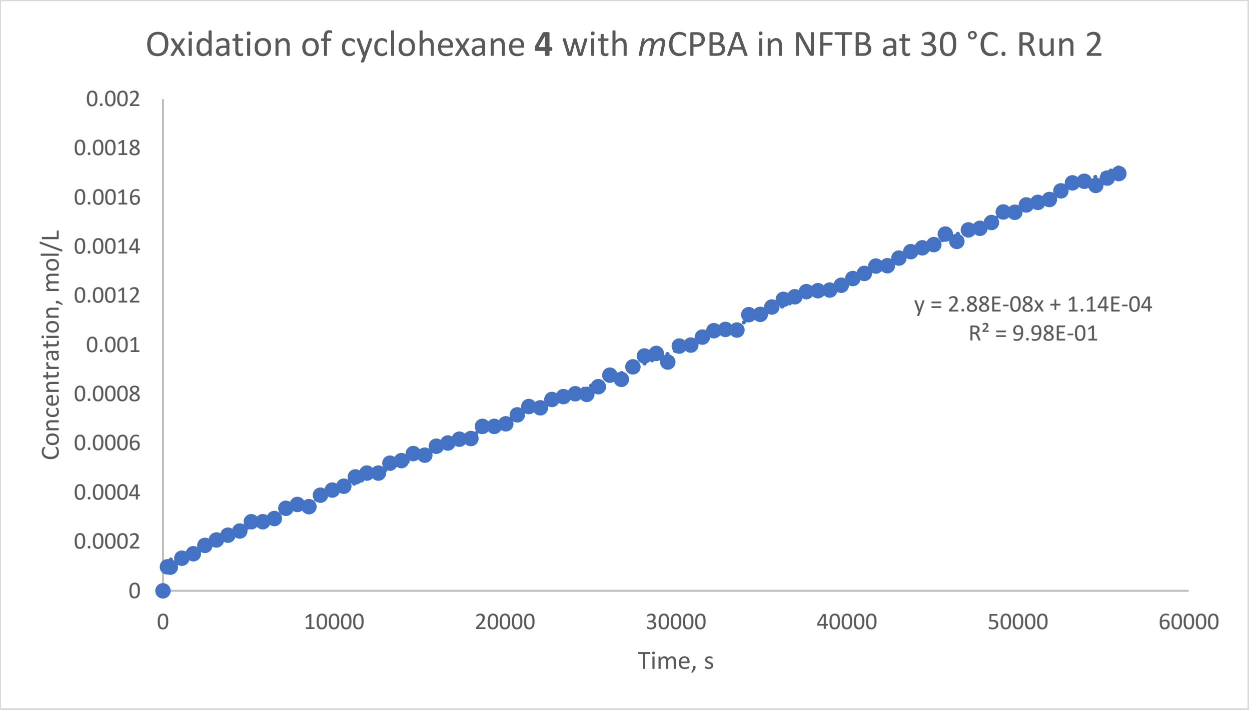
Oxidation of cyclohexane **4** with *m*CPBA in NFTB at 30 °C. Run 2
Concentration, mol/L
0.002
0.0018
0.0016
0.0014
0.0012
0.001
0.0008
0.0006
0.0004
0.0002
0
0
10000
20000
30000
40000
50000
60000
Time, s
y = 2.88E-08x + 1.14E-04
R² = 9.98E-01

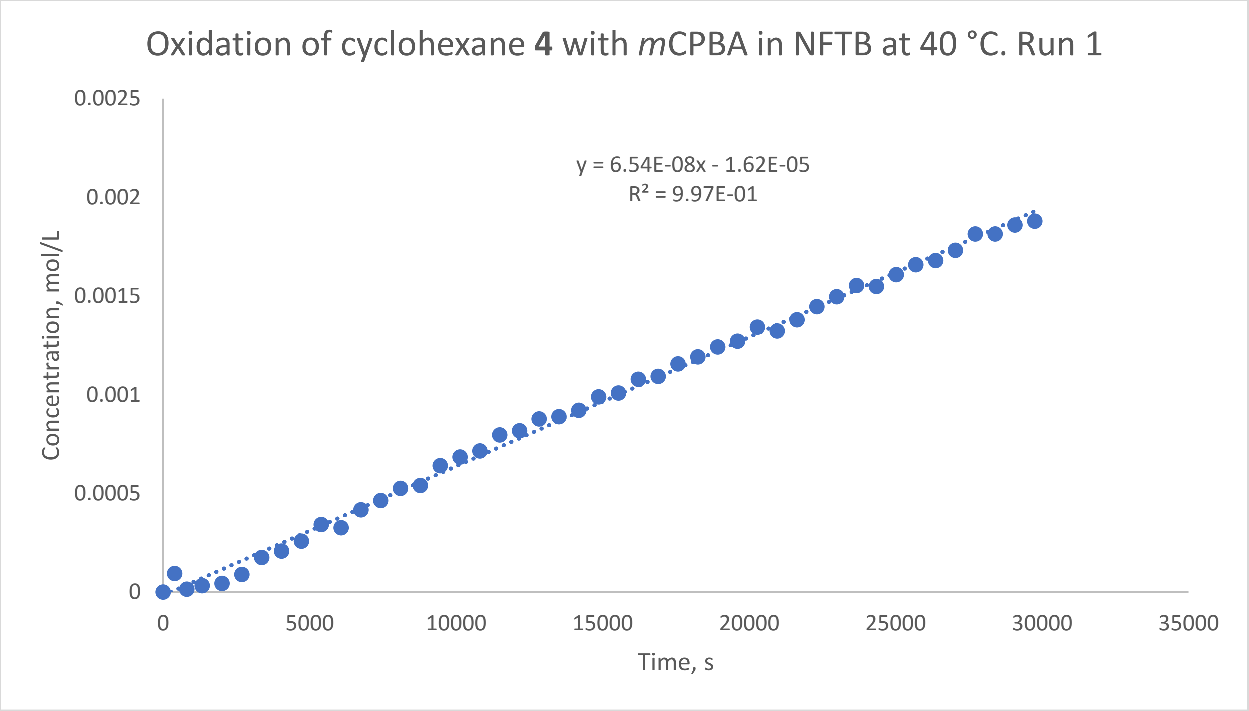
Oxidation of cyclohexane 4 with mCPBA in NFTB at 40 °C. Run 1
y = 6.54E-08x - 1.62E-05
R² = 9.97E-01
Concentration, mol/L
Time, s
0
0.0005
0.001
0.0015
0.002
0.0025
0
5000
10000
15000
20000
25000
30000
35000

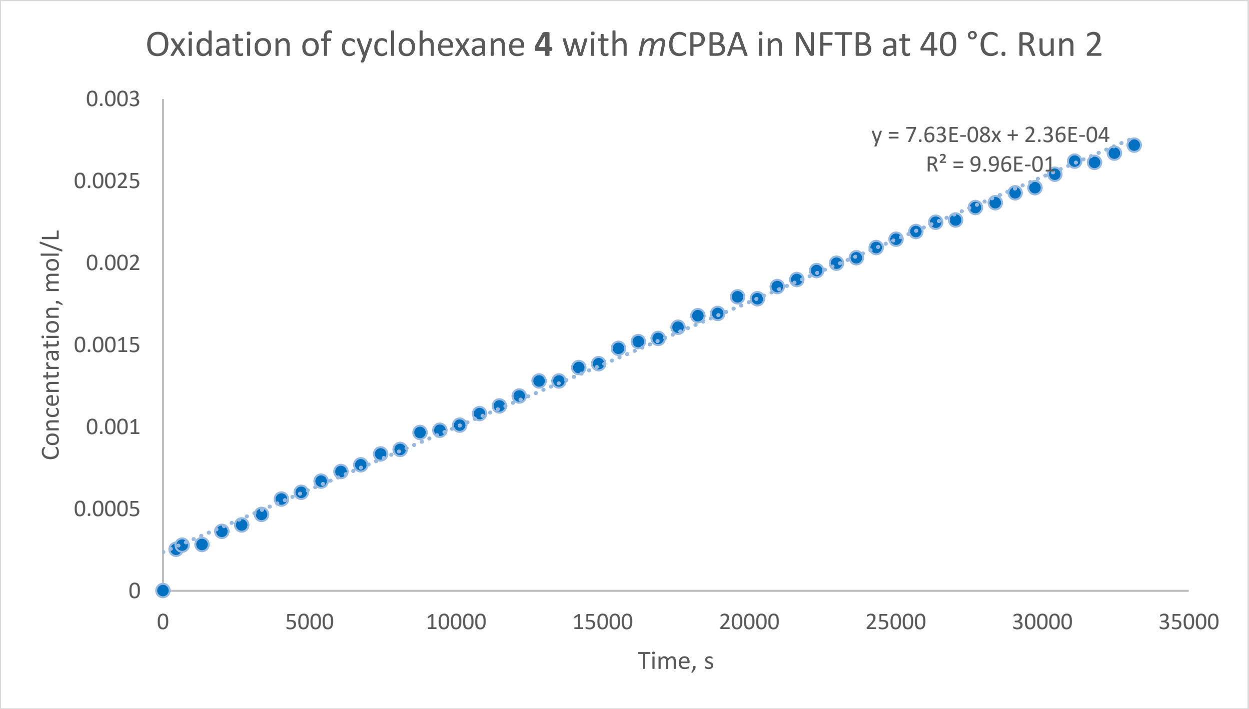
Oxidation of cyclohexane 4 with mCPBA in NFTB at 40 °C. Run 2
y = 7.63E-08x + 2.36E-04
R² = 9.96E-01
Concentration, mol/L
Time, s
0
0.0005
0.001
0.0015
0.002
0.0025
0.003
0
5000
10000
15000
20000
25000
30000
35000

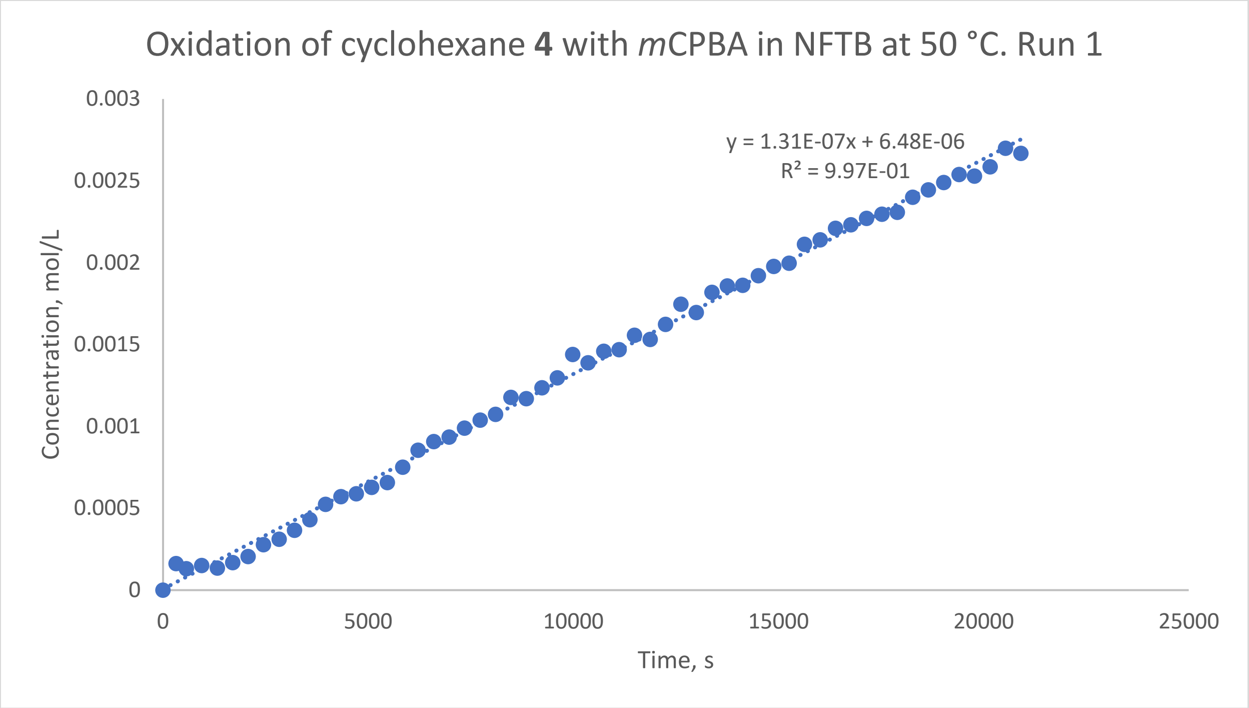

Oxidation of cyclohexane **4** with *m*CPBA in NFTB at 50 °C. Run 1
y = 1.31E-07x + 6.48E-06
R² = 9.97E-01
Concentration, mol/L
Time, s

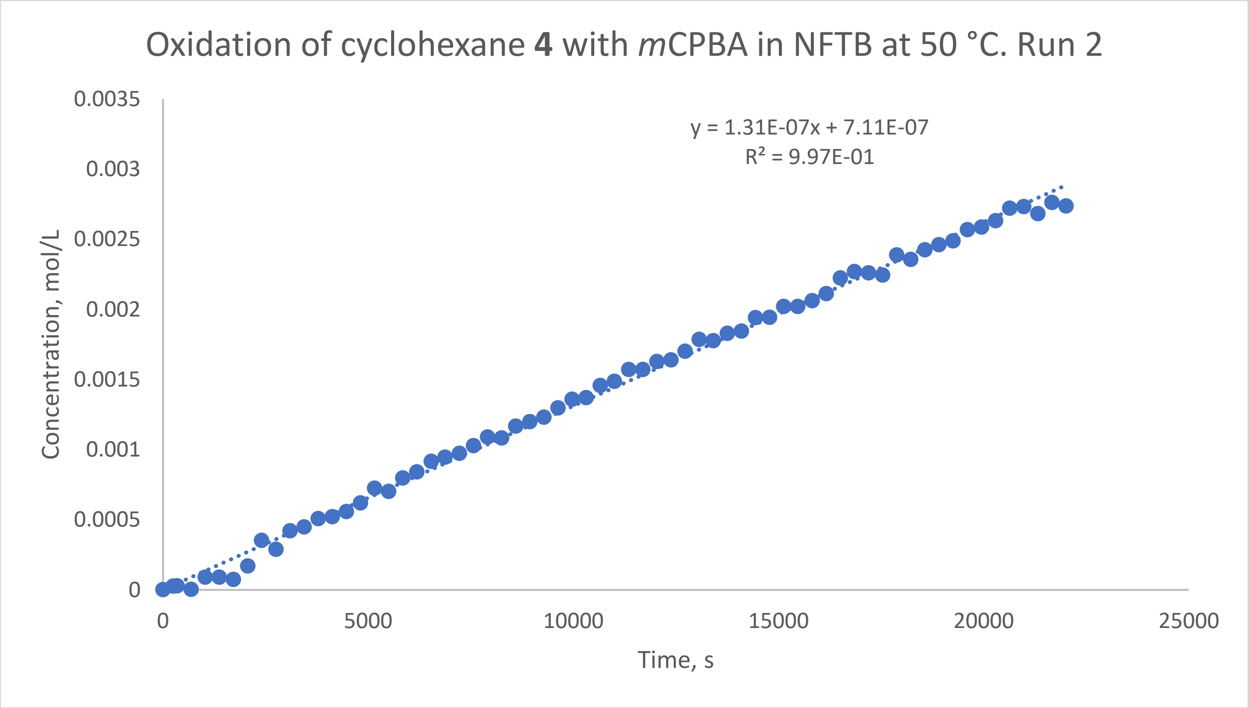

Oxidation of cyclohexane **4** with *m*CPBA in NFTB at 50 °C. Run 2
y = 1.31E-07x + 7.11E-07
R² = 9.97E-01
Concentration, mol/L
Time, s

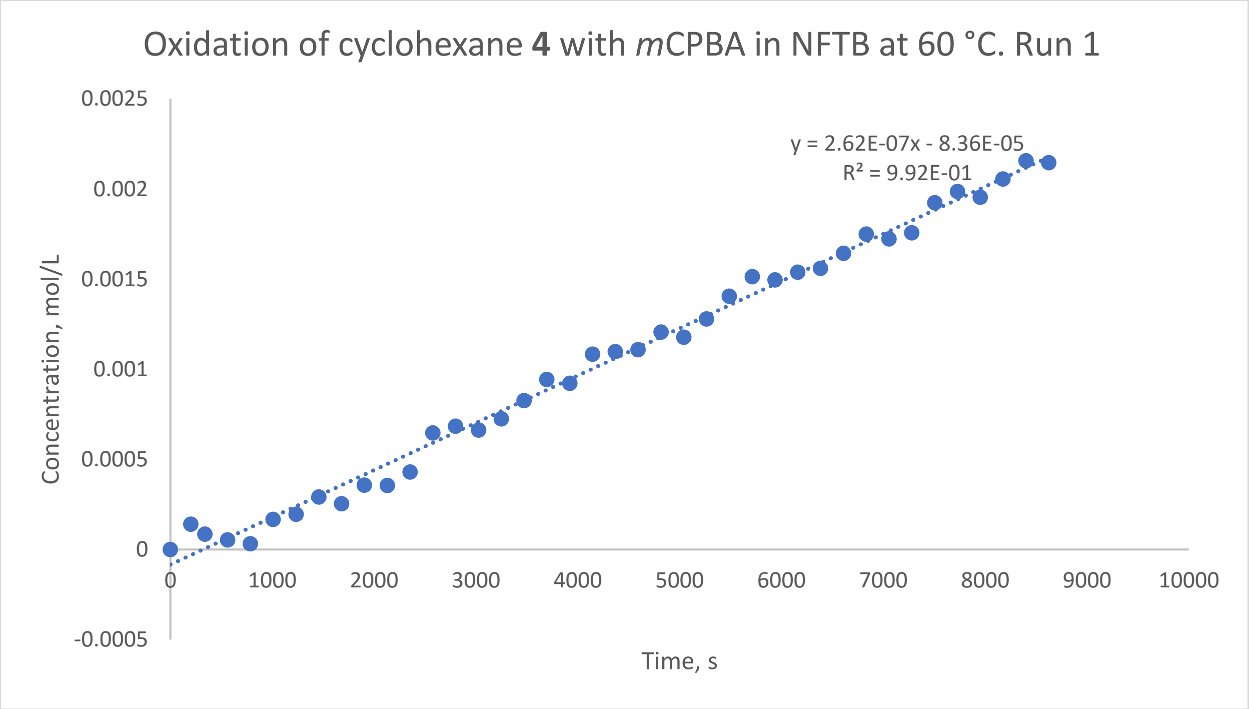

Oxidation of cyclohexane 4 with mCPBA in NFTB at 60 °C. Run 1
y = 2.62E-07x - 8.36E-05
R² = 9.92E-01
Concentration, mol/L
Time, s
0.0025
0.002
0.0015
0.001
0.0005
0
-0.0005
0
1000
2000
3000
4000
5000
6000
7000
8000
9000
10000

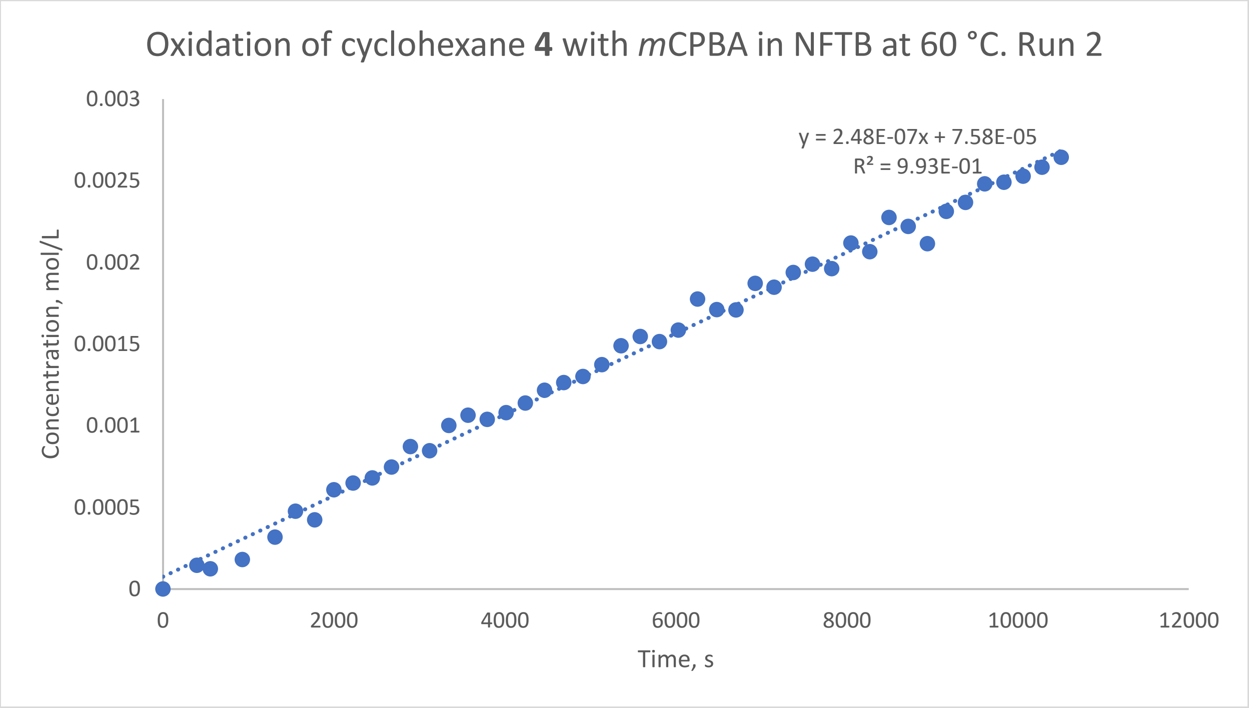

Oxidation of cyclohexane 4 with mCPBA in NFTB at 60 °C. Run 2
y = 2.48E-07x + 7.58E-05
R² = 9.93E-01
Concentration, mol/L
Time, s
0.003
0.0025
0.002
0.0015
0.001
0.0005
0
0
2000
4000
6000
8000
10000
12000

3.3 Eyring plots for oxidation of cyclohexane 4 in NFTB.

Temperature, °C	Rate constant *k*, $M^{-1}s^{-1}$	R^2	Average rate constant *k*, $M^{-1}s^{-1}$
30	4.19E-07	0.98	4.01E-07
	3.84E-07	0.99	
40	8.72E-07	0.97	9.45E-07
	1.02E-06	0.99	
50	1.75E-06	0.99	1.75E-06
	1.75E-06	0.99	
60	3.49E-06	0.99	3.40E-06
	3.31E-06	0.98	

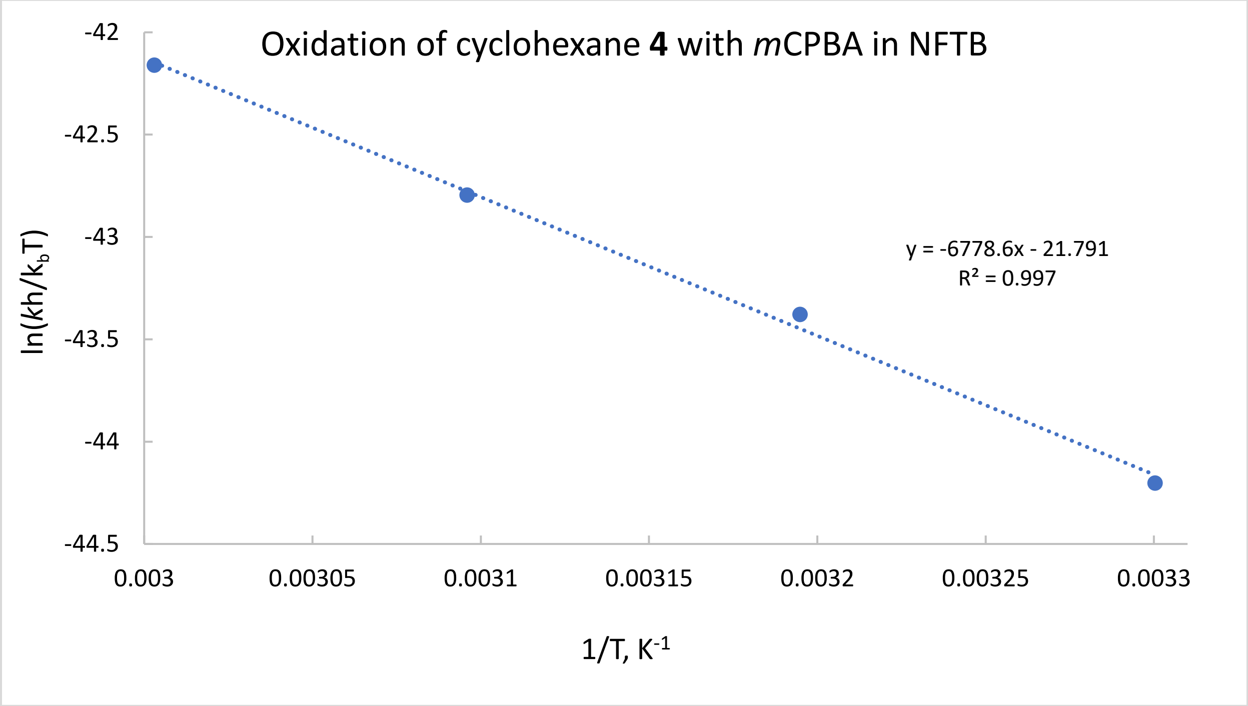

3.4 Kinetic investigation of oxidation of cyclohexanol 6 in $CDCl_3$.

In an NMR tube fitted with a Youngs tap, 0.80 mL 0.3125 M (46.40 mg, 10.0 eq) solution of 92% *meta*-chloroperbenzoic acid in $CDCl_3$ was placed in an NMR instrument and the temperature allowed to equilibrate. 0.20 mL of 0.03125 M (1.06 mg, 0.25 eq) solution of 1,4-dinitrobenzene in 0.125 M (2.50 mg, 1.0 eq) solution of cyclohexanol in $CDCl_3$ were added to the reaction mixture. The time of addition was recorded and a ^{1}H NMR spectra was obtained every 3 to 10 minutes until consumption of 10% of the cyclohexanol was observed. The reactions were conducted in duplicate at 30, 40, 50 and 60 °C. Observed rate constants were obtained using initial reaction rates by plotting [CyOH] *versus* time. The observed rate constants were divided by $[m\text{CPBA}]_0[\text{CyOH}]_0$.

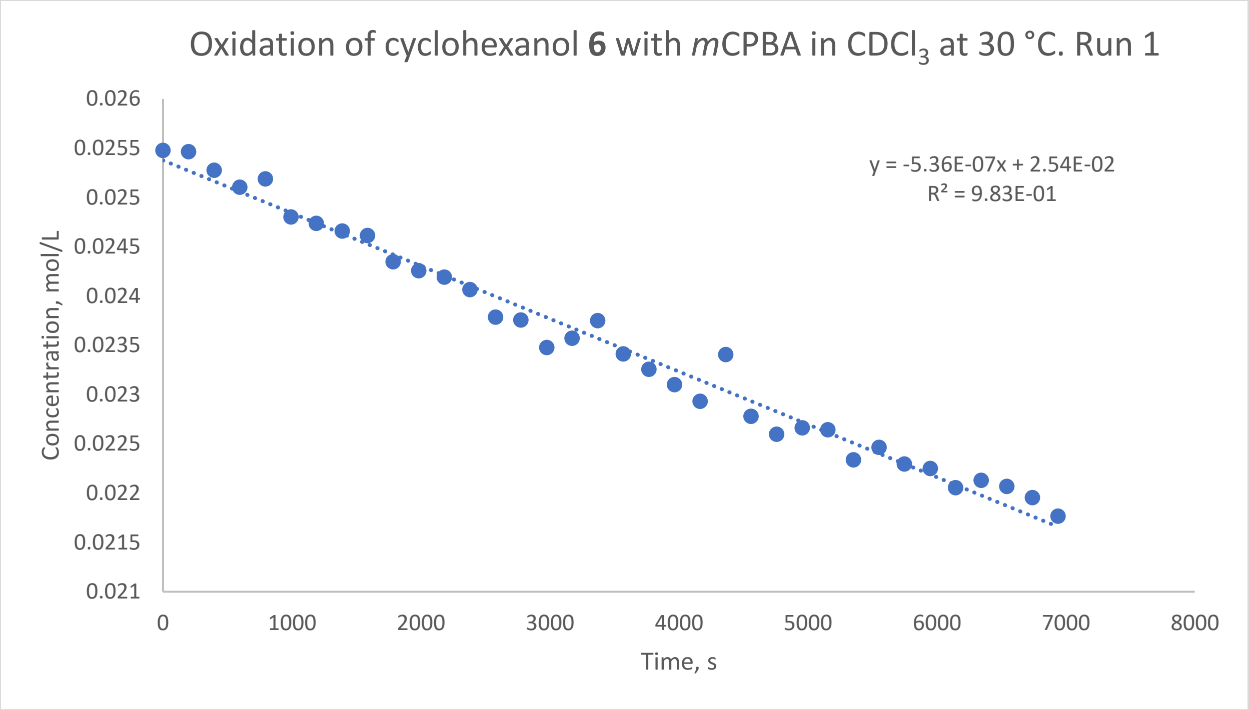
Oxidation of cyclohexanol 6 with mCPBA in CDCl3 at 30 °C. Run 1
y = -5.36E-07x + 2.54E-02
R² = 9.83E-01
Concentration, mol/L
Time, s
0.026
0.0255
0.025
0.0245
0.024
0.0235
0.023
0.0225
0.022
0.0215
0.021
0
1000
2000
3000
4000
5000
6000
7000
8000

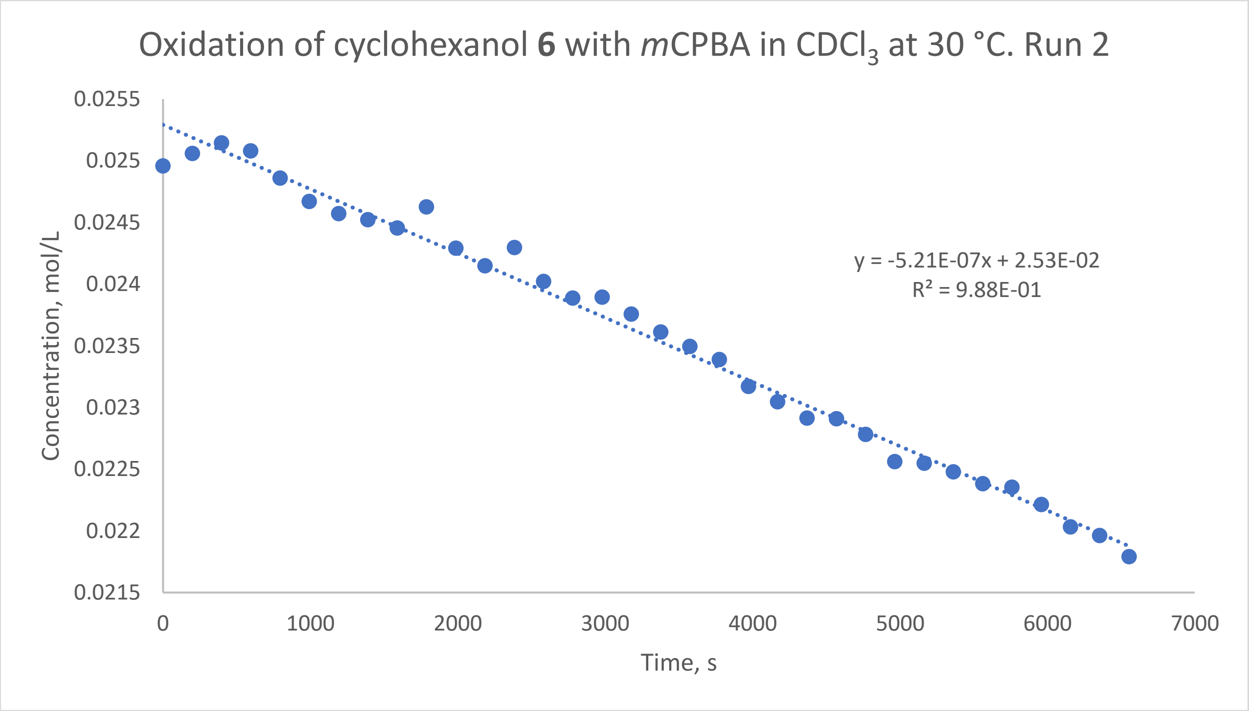
Oxidation of cyclohexanol 6 with mCPBA in CDCl3 at 30 °C. Run 2
y = -5.21E-07x + 2.53E-02
R² = 9.88E-01
Concentration, mol/L
Time, s
0.0255
0.025
0.0245
0.024
0.0235
0.023
0.0225
0.022
0.0215
0
1000
2000
3000
4000
5000
6000
7000

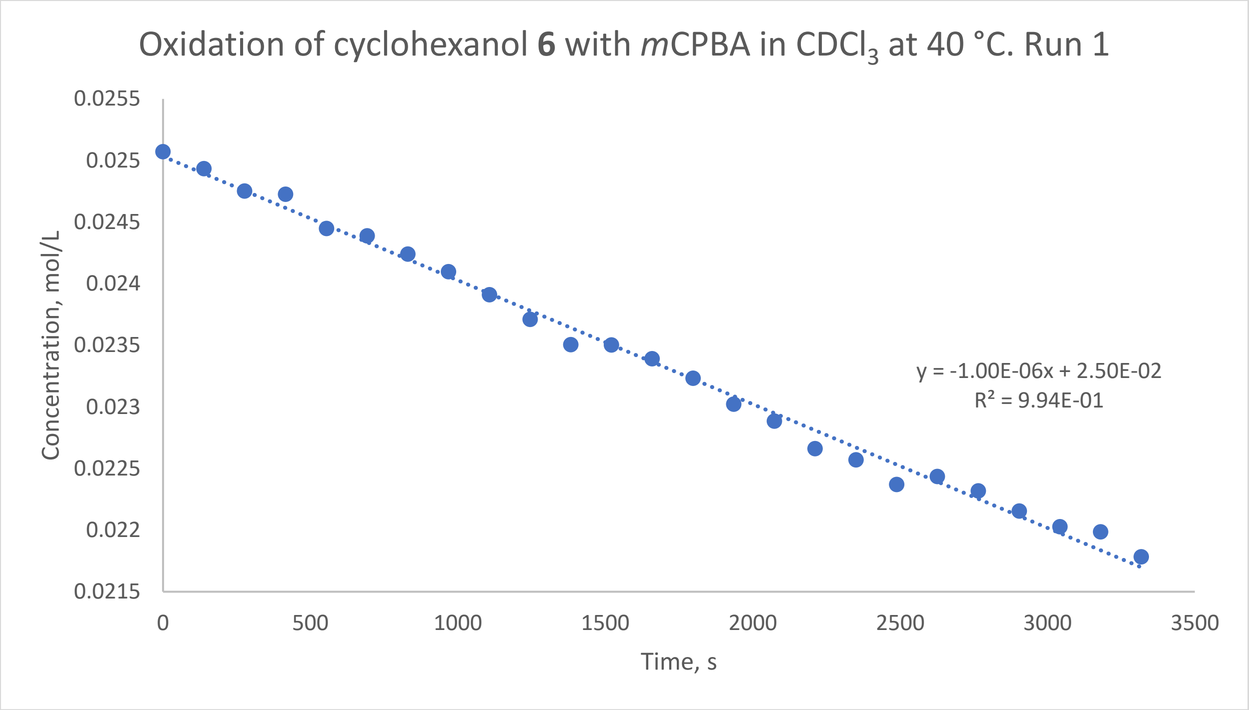

Oxidation of cyclohexanol **6** with *m*CPBA in $CDCl_3$ at 40 °C. Run 1
Concentration, mol/L
0.0255
0.025
0.0245
0.024
0.0235
0.023
0.0225
0.022
0.0215
0
500
1000
1500
2000
2500
3000
3500
Time, s
y = -1.00E-06x + 2.50E-02
R^2 = 9.94E-01

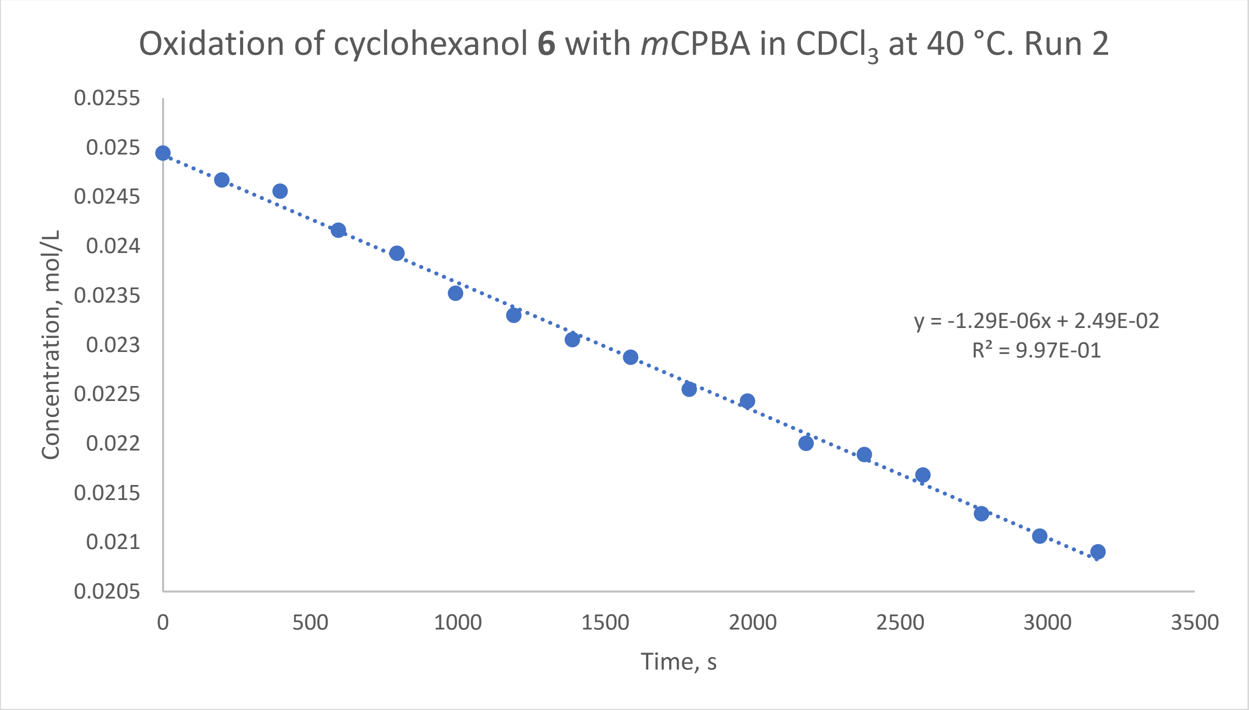

Oxidation of cyclohexanol **6** with *m*CPBA in $CDCl_3$ at 40 °C. Run 2
Concentration, mol/L
0.0255
0.025
0.0245
0.024
0.0235
0.023
0.0225
0.022
0.0215
0.021
0.0205
0
500
1000
1500
2000
2500
3000
3500
Time, s
y = -1.29E-06x + 2.49E-02
R^2 = 9.97E-01

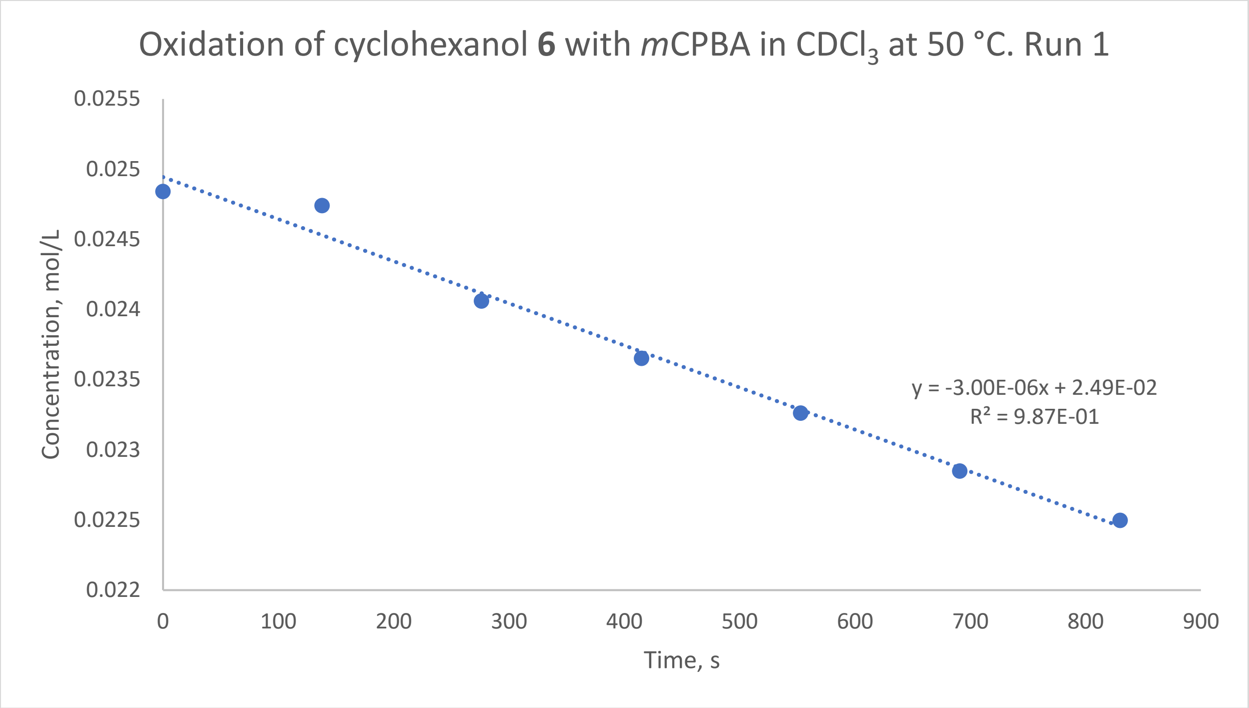

Oxidation of cyclohexanol 6 with mCPBA in CDCl3 at 50 °C. Run 1
Concentration, mol/L
Time, s
0.0255
0.025
0.0245
0.024
0.0235
0.023
0.0225
0.022
0
100
200
300
400
500
600
700
800
900
y = -3.00E-06x + 2.49E-02
R² = 9.87E-01

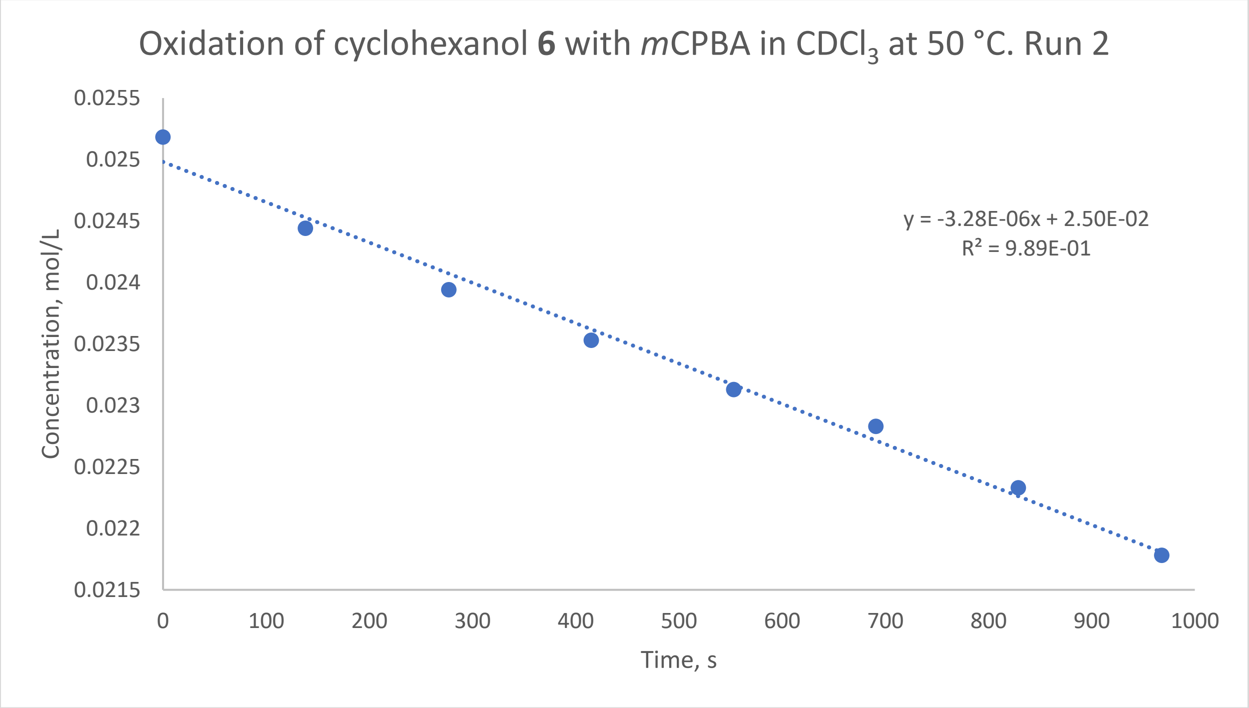

Oxidation of cyclohexanol 6 with mCPBA in CDCl3 at 50 °C. Run 2
Concentration, mol/L
Time, s
0.0255
0.025
0.0245
0.024
0.0235
0.023
0.0225
0.022
0.0215
0
100
200
300
400
500
600
700
800
900
1000
y = -3.28E-06x + 2.50E-02
R² = 9.89E-01

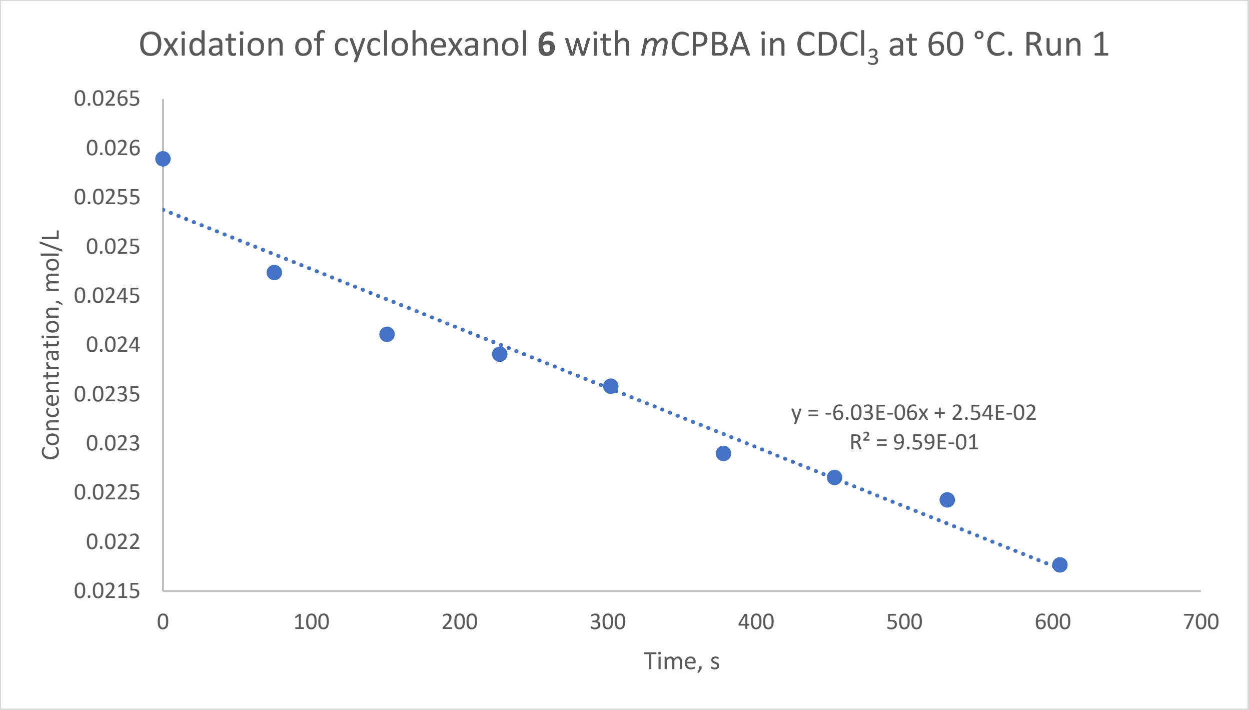
Oxidation of cyclohexanol 6 with mCPBA in CDCl3 at 60 °C. Run 1
y = -6.03E-06x + 2.54E-02
R² = 9.59E-01
Concentration, mol/L
Time, s
0.0265
0.026
0.0255
0.025
0.0245
0.024
0.0235
0.023
0.0225
0.022
0.0215
0
100
200
300
400
500
600
700

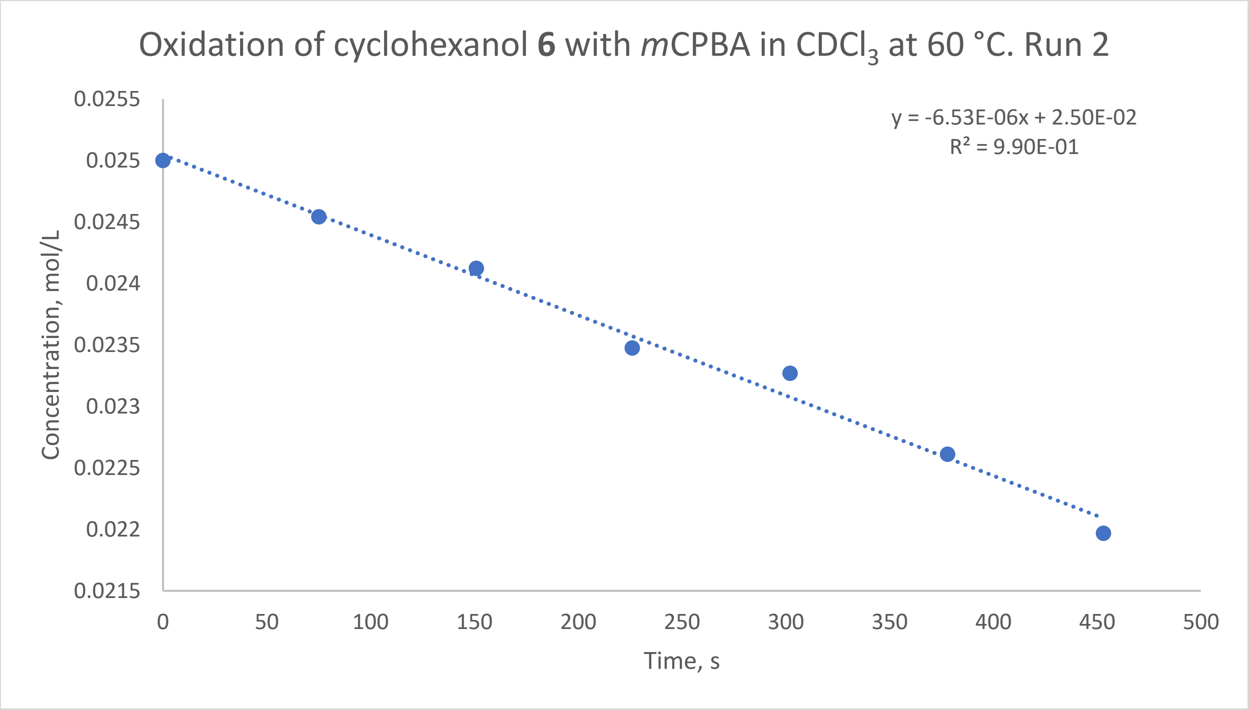
Oxidation of cyclohexanol 6 with mCPBA in CDCl3 at 60 °C. Run 2
y = -6.53E-06x + 2.50E-02
R² = 9.90E-01
Concentration, mol/L
Time, s
0.0255
0.025
0.0245
0.024
0.0235
0.023
0.0225
0.022
0.0215
0
50
100
150
200
250
300
350
400
450
500

3.5 Eyring plots for oxidation of cyclohexanol 6 in $CDCl_3$.

Temperature, °C	Rate constant *k*, $M^{-1}s^{-1}$	R^2	Average rate constant *k*, $M^{-1}s^{-1}$
30	8.58E-05	0.98	8.46E-05
	8.34E-05	0.99	
40	1.60E-04	0.99	1.83E-04
	2.06E-04	0.99	
50	4.80E-04	0.99	5.02E-04
	5.25E-04	0.99	
60	9.65E-04	0.99	1.00E-03
	1.04E-03	0.99	

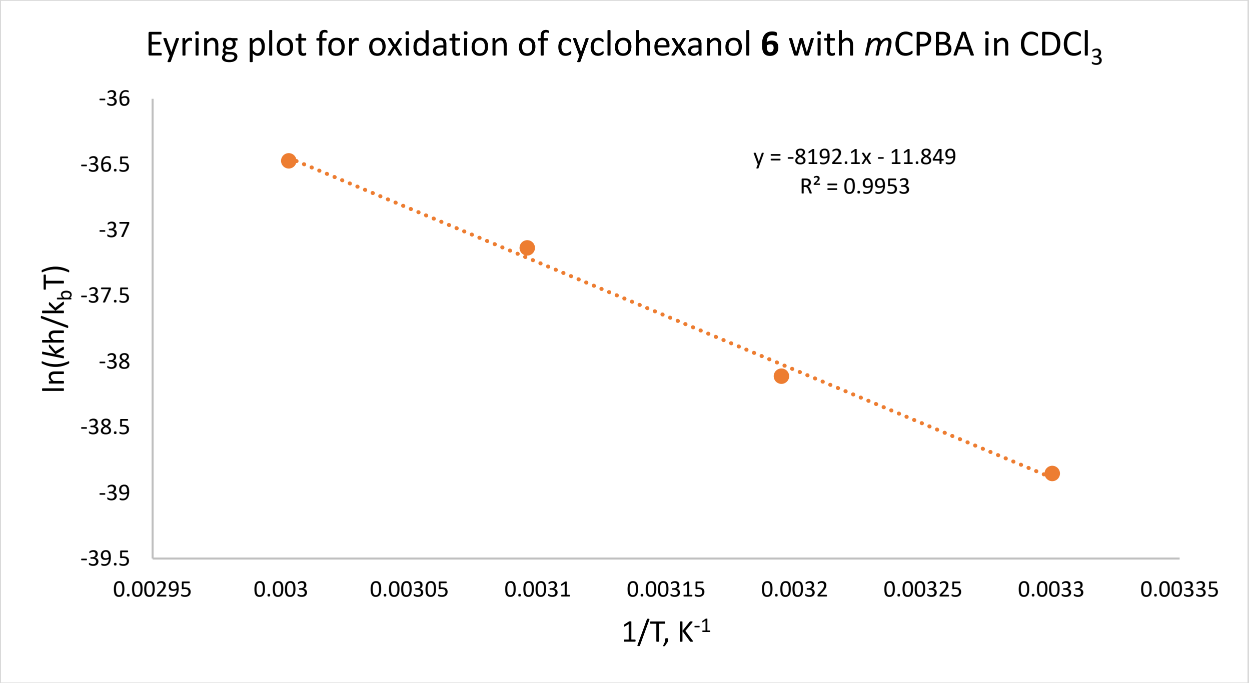

3.6 Kinetic investigation of oxidation of cyclohexanol 6 in NFTB.

In an NMR tube fitted with a Youngs tap, 0.80 mL 0.3125 M (46.40 mg, 10.0 eq) solution of 92% *meta*-chloroperbenzoic acid **5** in NFTB was placed in an NMR instrument and the temperature allowed to equilibrate. 0.20 mL of 0.03125 M (1.06 mg, 0.25 eq) solution of 1,4-dinitrobenzene in 0.125 M (2.50 mg, 1.0 eq) solution of cyclohexanol in NFTB were added to the reaction mixture. The time of addition was recorded and a ^{1}H NMR spectra was obtained every 3 to 10 minutes until consumption of 10% of the cyclohexanol was observed. The reactions were conducted in duplicate at 30, 40, 50 and 60 °C. As NFTB overlaps with characteristic cyclohexanol peaks the rate of oxidation of cyclohexanol was observed by formation of the product of overoxidation caprolactone **8**. Observed rate constants were obtained using initial reaction rates by plotting [Caprolactone]+[Cyclohexanone] *versus* time. The observed rate constants were divided by $[m\text{CPBA}]_0[\text{CyOH}]_0$.

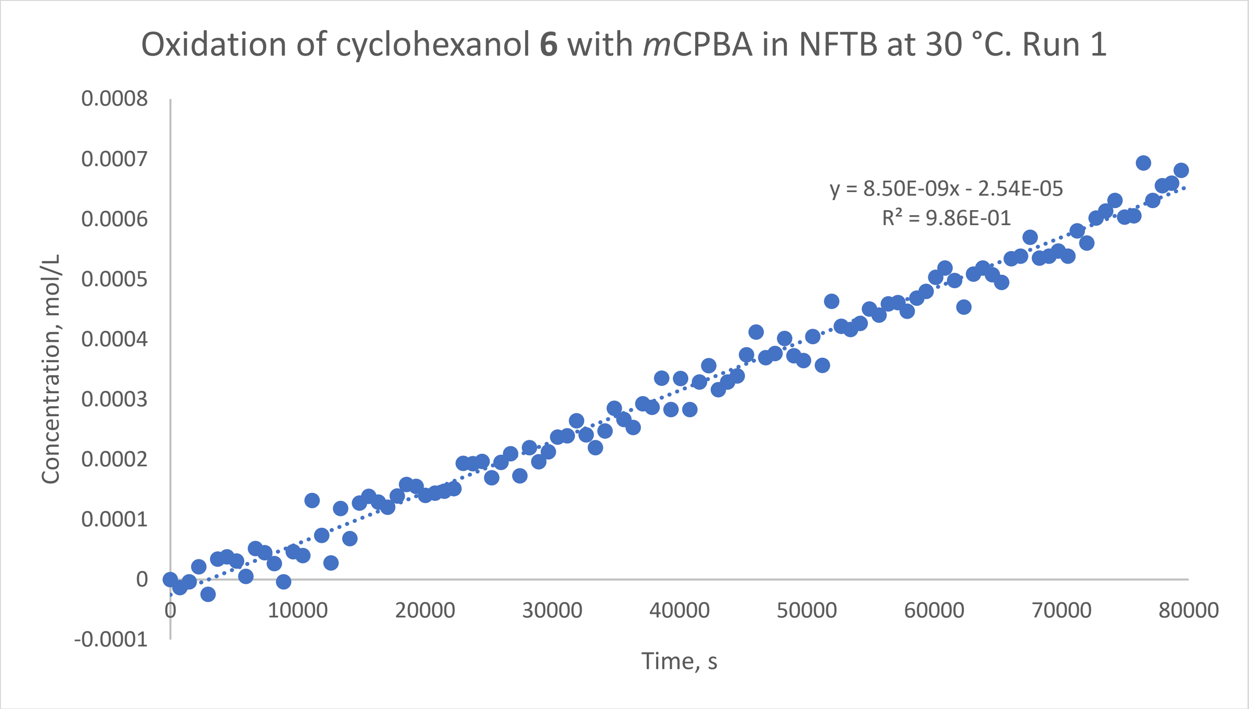
Oxidation of cyclohexanol 6 with mCPBA in NFTB at 30 °C. Run 1
y = 8.50E-09x - 2.54E-05
R² = 9.86E-01
Concentration, mol/L
Time, s
0.0008
0.0007
0.0006
0.0005
0.0004
0.0003
0.0002
0.0001
0
-0.0001
0
10000
20000
30000
40000
50000
60000
70000
80000

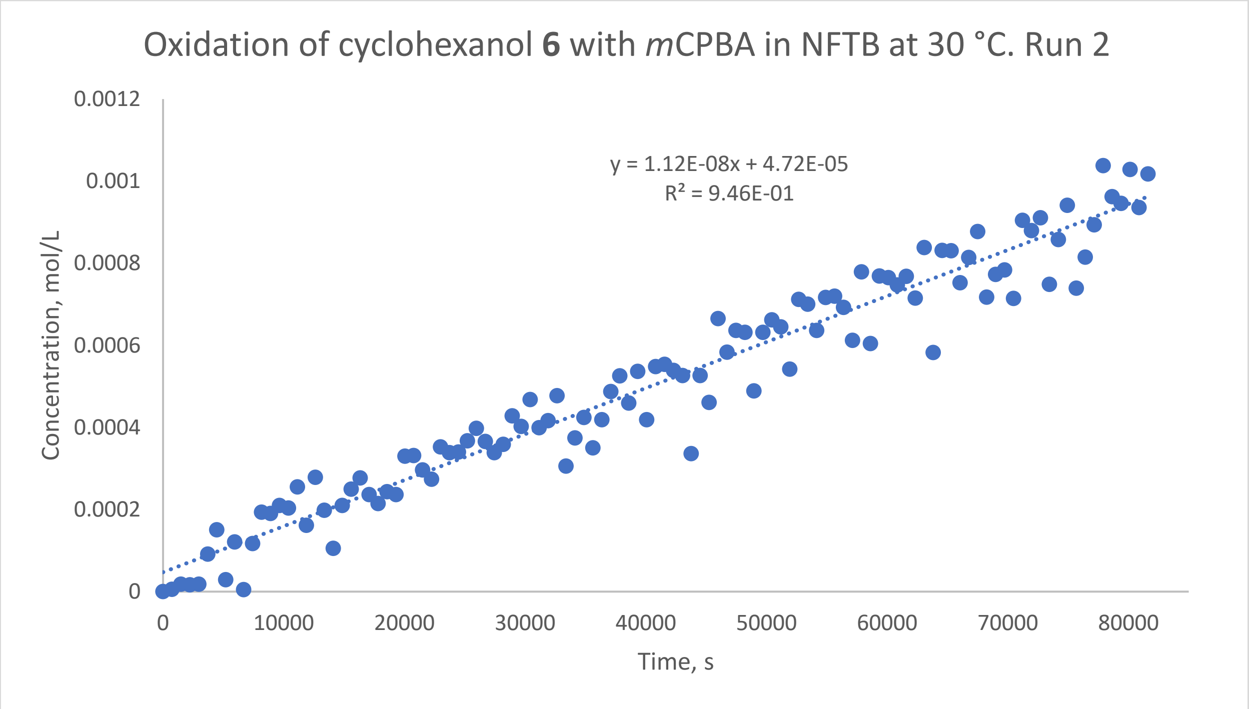
Oxidation of cyclohexanol 6 with mCPBA in NFTB at 30 °C. Run 2
y = 1.12E-08x + 4.72E-05
R² = 9.46E-01
Concentration, mol/L
Time, s
0.0012
0.001
0.0008
0.0006
0.0004
0.0002
0
0
10000
20000
30000
40000
50000
60000
70000
80000

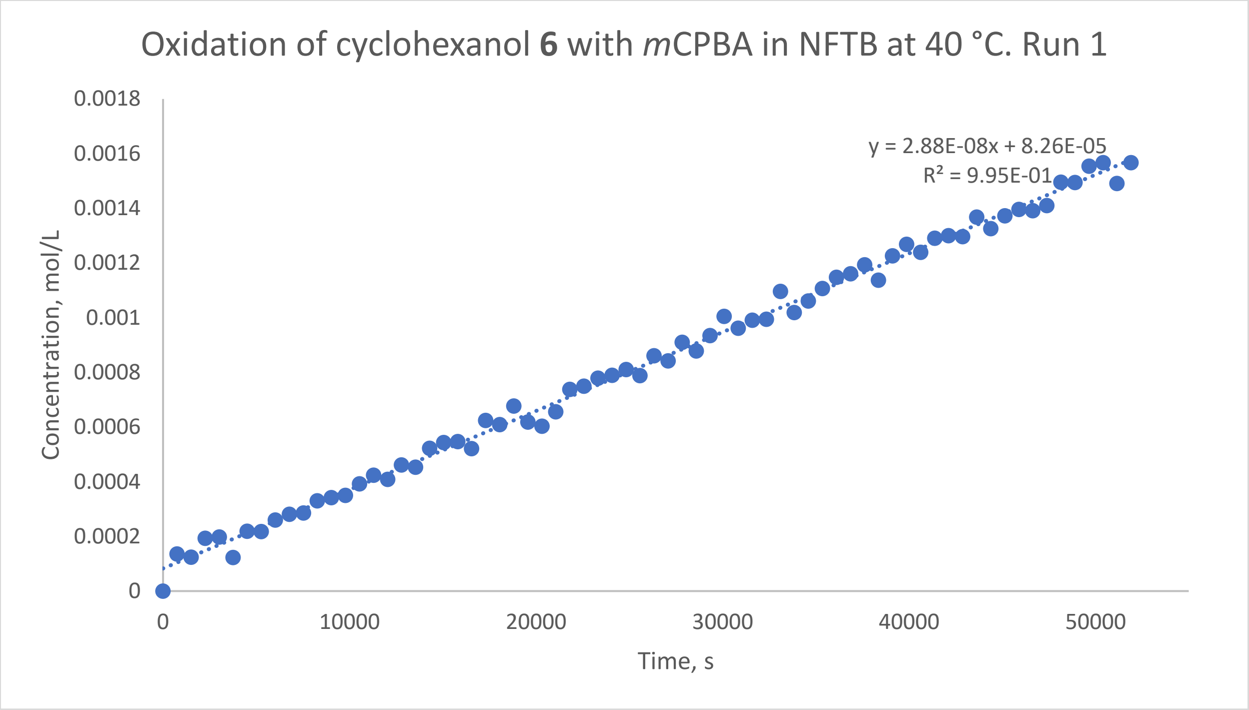

Oxidation of cyclohexanol **6** with *m*CPBA in NFTB at 40 °C. Run 1
y = 2.88E-08x + 8.26E-05
R² = 9.95E-01
Concentration, mol/L
0.0018
0.0016
0.0014
0.0012
0.001
0.0008
0.0006
0.0004
0.0002
0
0
10000
20000
30000
40000
50000
Time, s

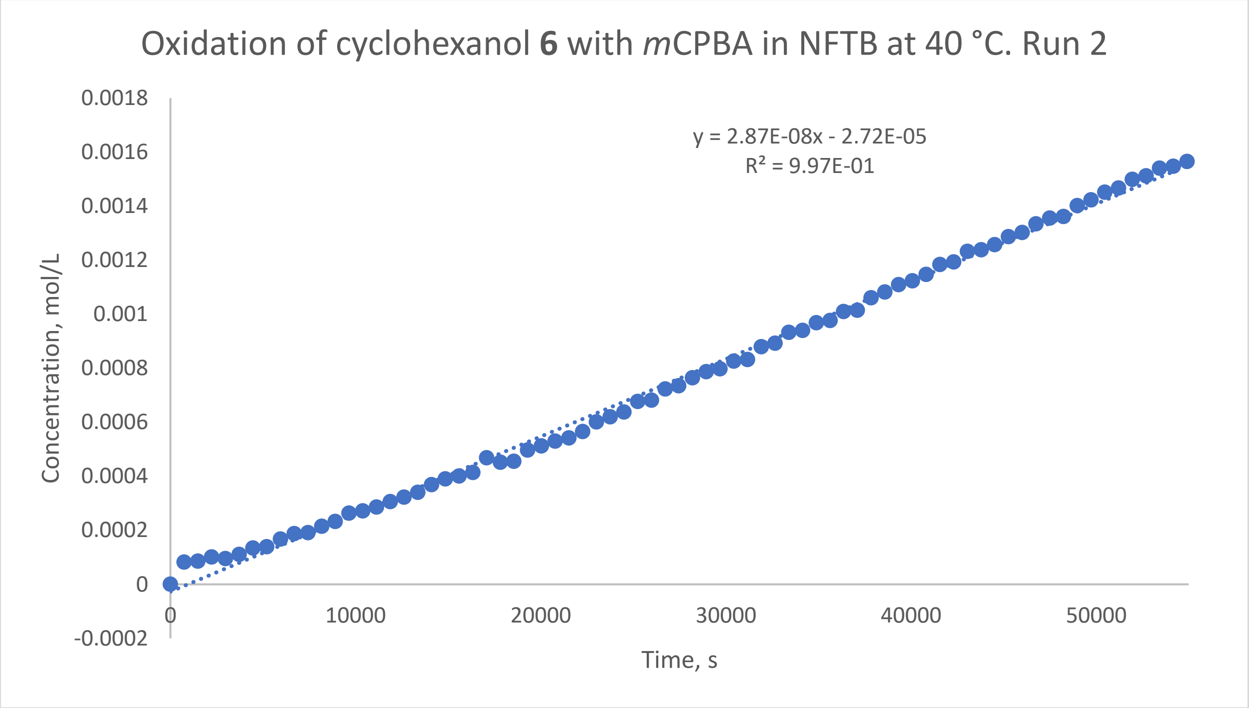

Oxidation of cyclohexanol **6** with *m*CPBA in NFTB at 40 °C. Run 2
y = 2.87E-08x - 2.72E-05
R² = 9.97E-01
Concentration, mol/L
0.0018
0.0016
0.0014
0.0012
0.001
0.0008
0.0006
0.0004
0.0002
0
-0.0002
0
10000
20000
30000
40000
50000
Time, s

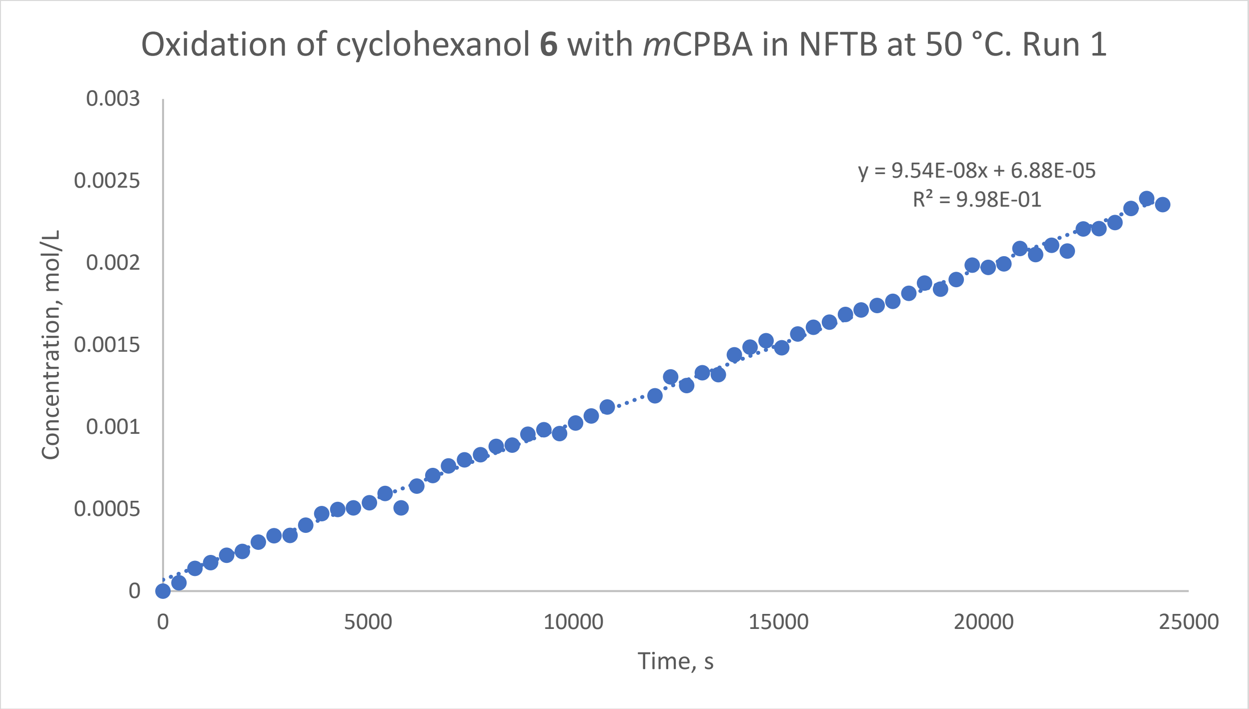

Oxidation of cyclohexanol **6** with *m*CPBA in NFTB at 50 °C. Run 1
y = 9.54E-08x + 6.88E-05
R² = 9.98E-01
Concentration, mol/L
0.003
0.0025
0.002
0.0015
0.001
0.0005
0
0
5000
10000
15000
20000
25000
Time, s

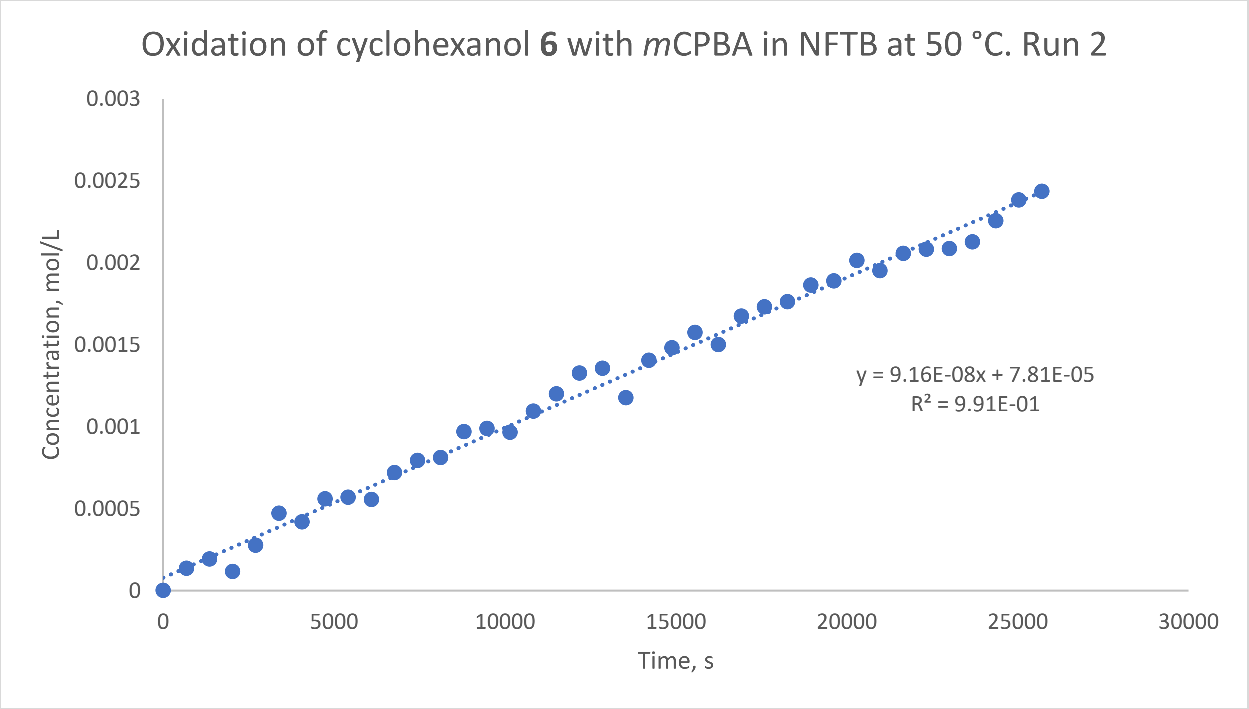

Oxidation of cyclohexanol **6** with *m*CPBA in NFTB at 50 °C. Run 2
y = 9.16E-08x + 7.81E-05
R² = 9.91E-01
Concentration, mol/L
0.003
0.0025
0.002
0.0015
0.001
0.0005
0
0
5000
10000
15000
20000
25000
30000
Time, s

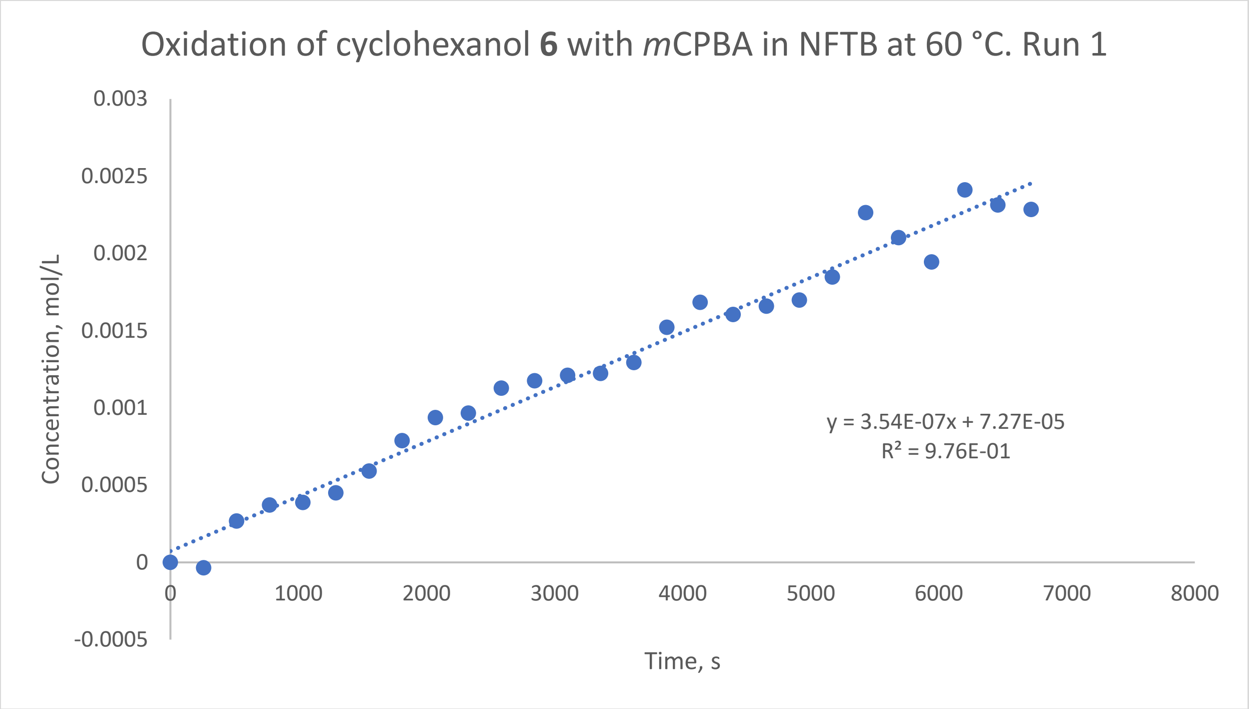

Oxidation of cyclohexanol 6 with mCPBA in NFTB at 60 °C. Run 1
Concentration, mol/L
Time, s
y = 3.54E-07x + 7.27E-05
R² = 9.76E-01
0.003
0.0025
0.002
0.0015
0.001
0.0005
0
-0.0005
0
1000
2000
3000
4000
5000
6000
7000
8000

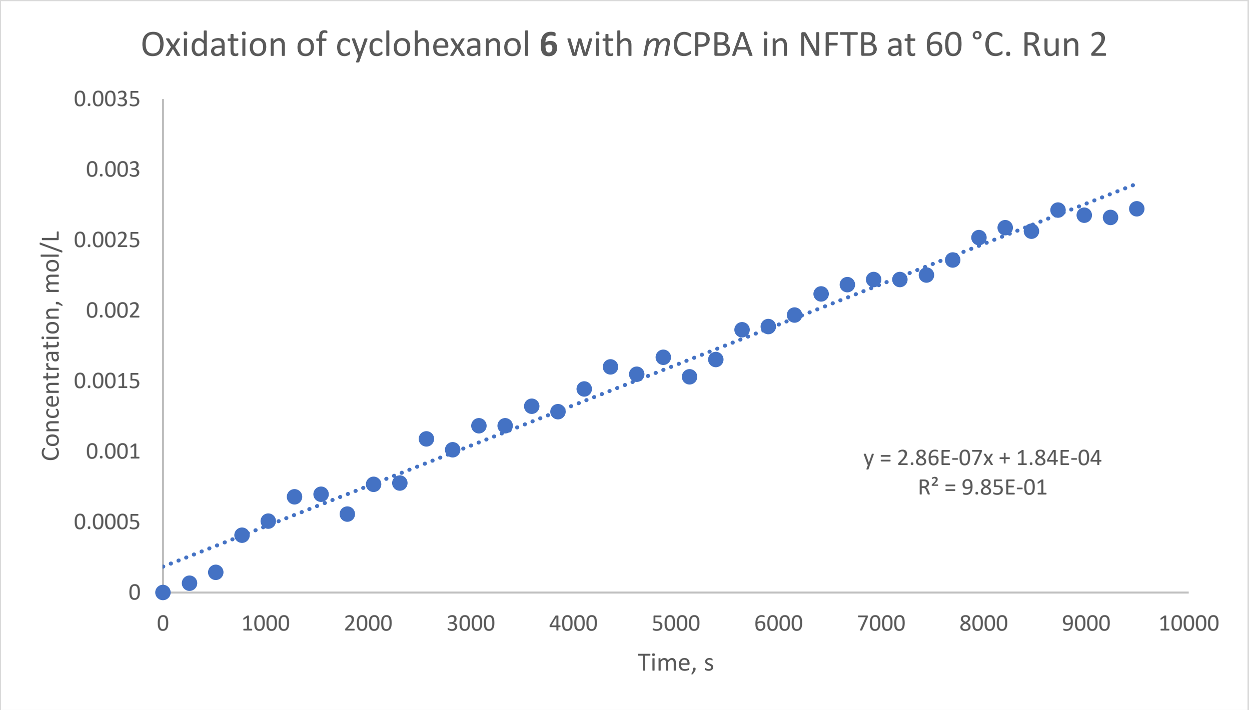

Oxidation of cyclohexanol 6 with mCPBA in NFTB at 60 °C. Run 2
Concentration, mol/L
Time, s
y = 2.86E-07x + 1.84E-04
R² = 9.85E-01
0.0035
0.003
0.0025
0.002
0.0015
0.001
0.0005
0
0
1000
2000
3000
4000
5000
6000
7000
8000
9000
10000

3.7 Eyring plots for oxidation of cyclohexanol 6 in NFTB.

Temperature, °C	Rate constant *k*, $M^{-1}s^{-1}$	R^2	Average rate constant *k*, $M^{-1}s^{-1}$
30	1.36E-06	0.99	-1.58E-06
	1.79E-06	0.95	
40	4.61E-06	0.99	4.60E-06
	4.59E-06	0.99	
50	1.52E-05	0.99	1.54E-05
	1.56E-05	0.98	
60	5.66E-05	0.98	5.12E-05
	4.58E-05	0.99	

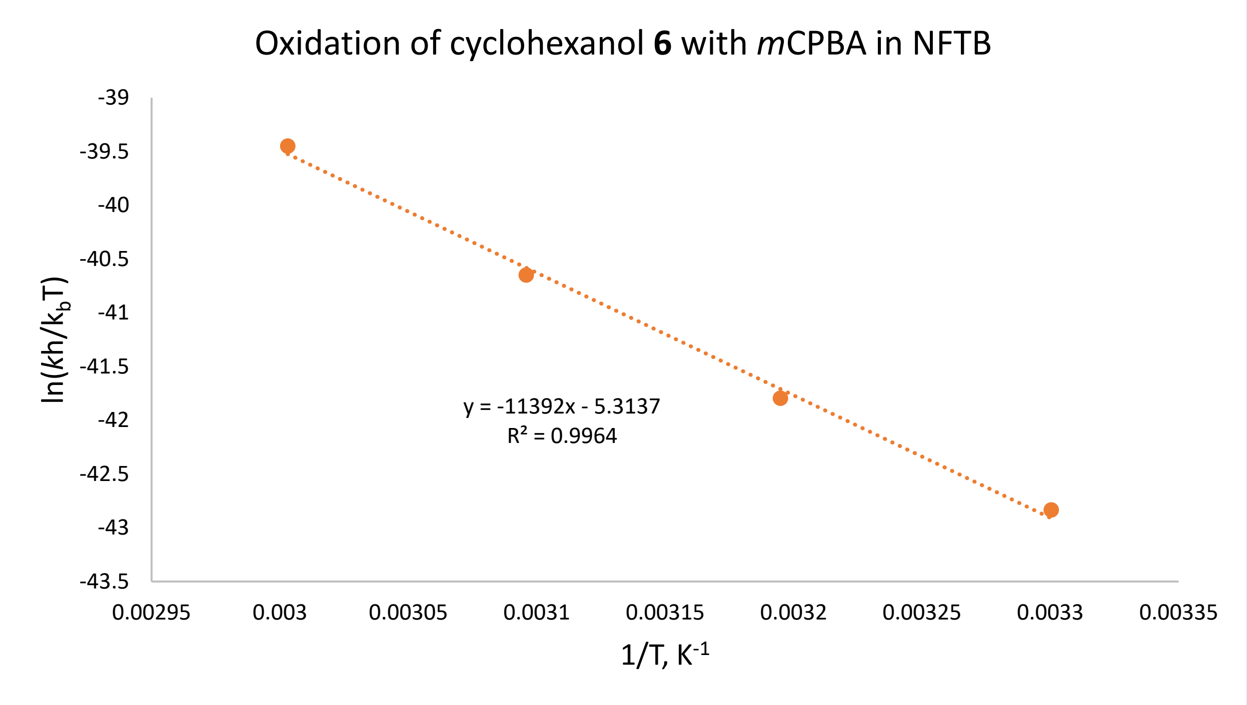

4 Analyses of the crude reaction mixtures.

Reactions were performed in sealed microwave vials (0.5-2.5 mL). Upon reaction completion 0.25 eq of 1,4-dinitrobenzene (solution at 21.0 mg/mL in $CDCl_3$) was added to the reaction mixture, following homogenisation by adding 4.0 mL of $CDCl_3$, an aliquot of the reaction mixture was analysed by ^{1}H, ^{13}C and ^{19}F NMR spectroscopy.

^{1}H NMR of the crude reaction mixture of oxidation of cyclopentane 17 with *m*CPBA 5 in NFTB.

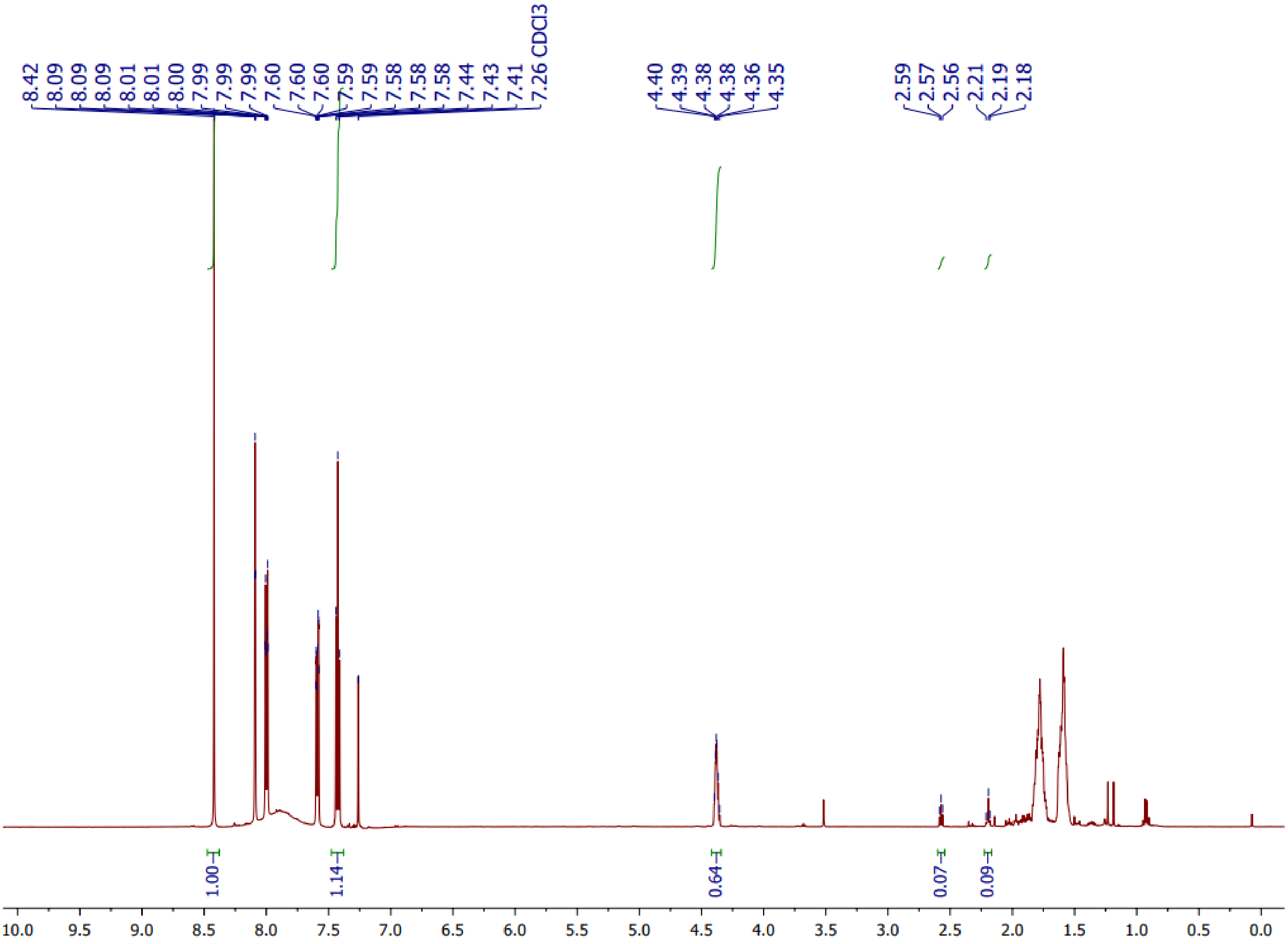

^{1}H NMR of the crude reaction mixture of oxidation of cyclopentane 17 with *m*CPBA 5 in NFTB.

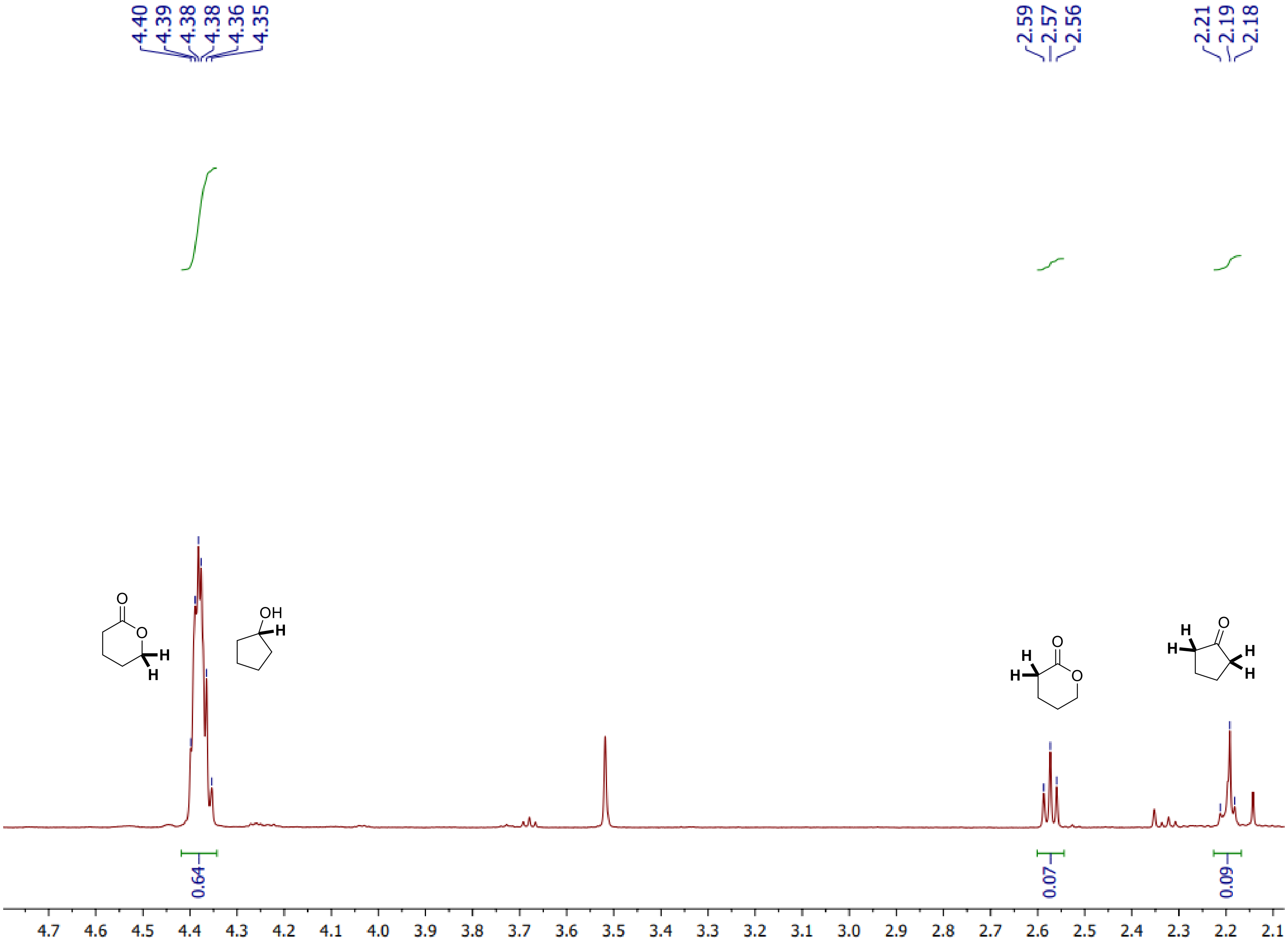

Compound Number	Structure	δ ppm	Integration	Number protons	% yield
**20**	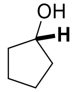	4.38	0.64-0.07=0.57	1	57
**23**	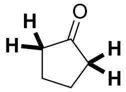	2.19	0.09	4	2
**26**	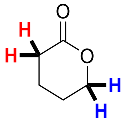	2.57<br>4.36	0.07<br>Overlapping	2	3

^{1}H NMR of the crude reaction mixture of oxidation of cyclohexane 4 with *m*CPBA 5 in NFTB.

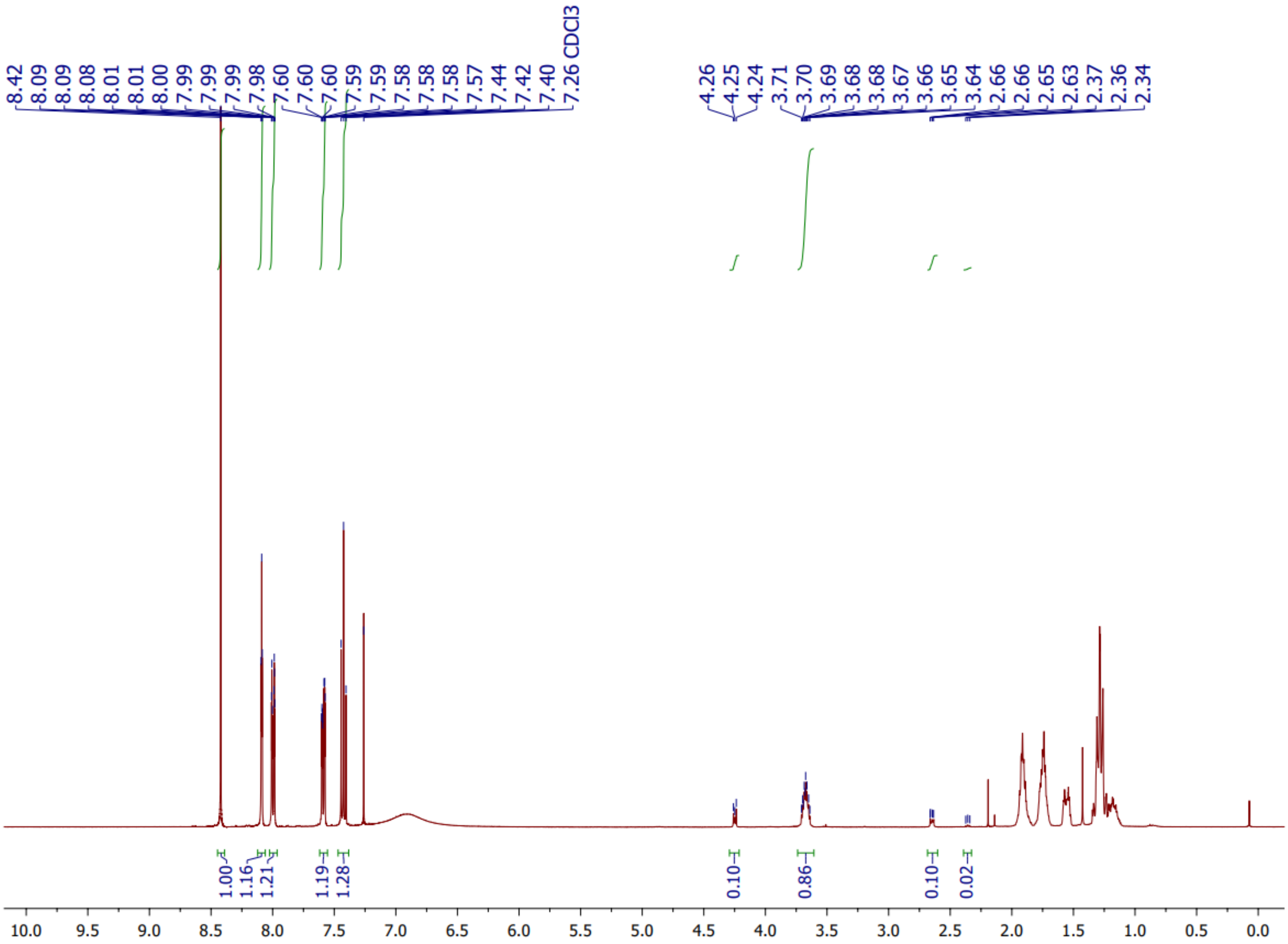

^{1}H NMR of the crude reaction mixture of oxidation of cyclohexane 4 with *m*CPBA 5 in NFTB.

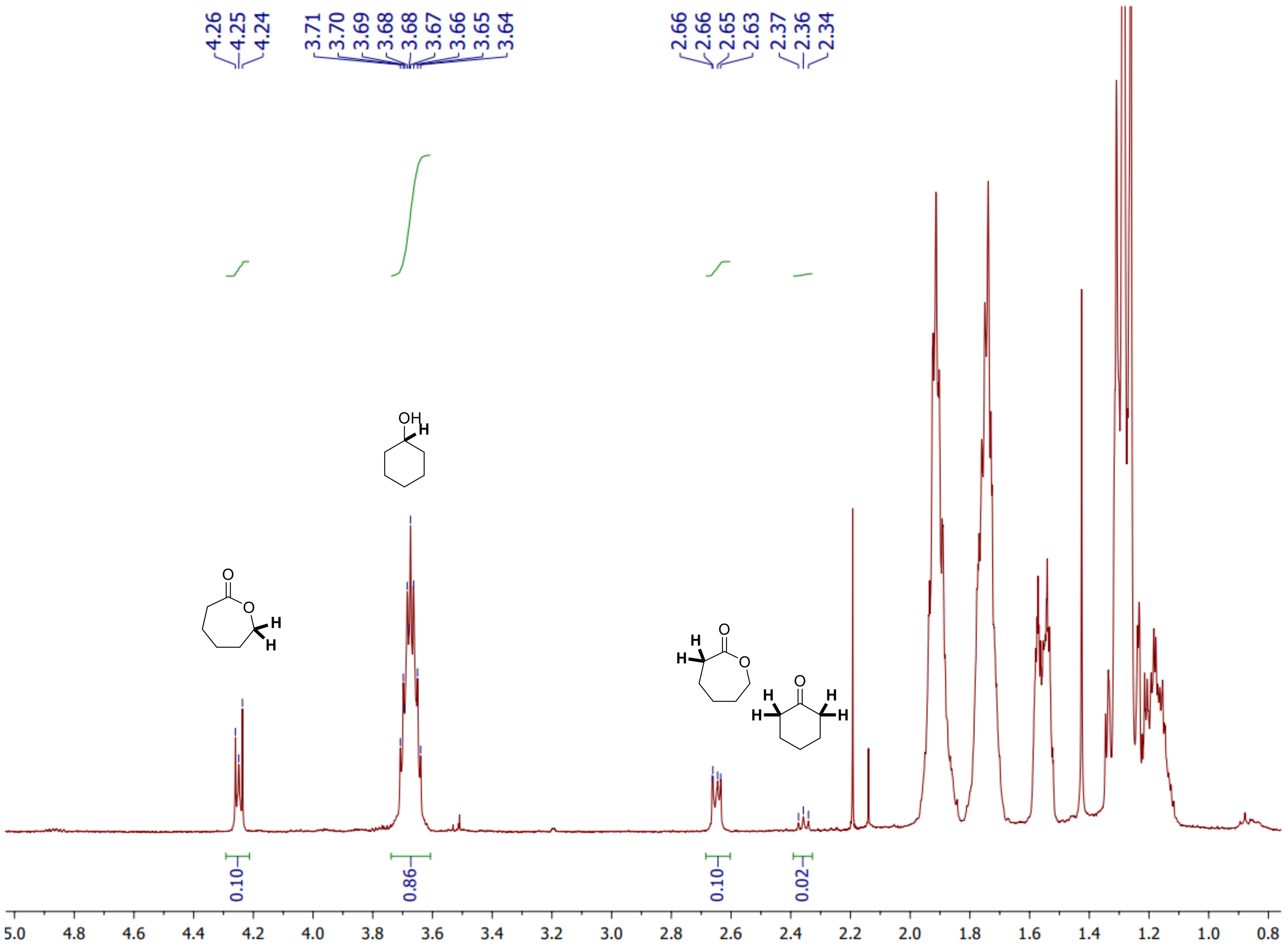

Compound Number	Structure	δ ppm	Integration	Number protons	% yield
**6**	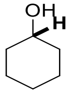	3.68	0.86	1	86
**7**	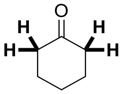	2.36	0.02	4	0.5
**8**	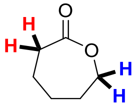	2.57<br>4.36	0.10<br>0.10	2	5

^{1}H NMR of the crude reaction mixture of oxidation of cycloheptane 18 with *m*CPBA 5 in NFTB.

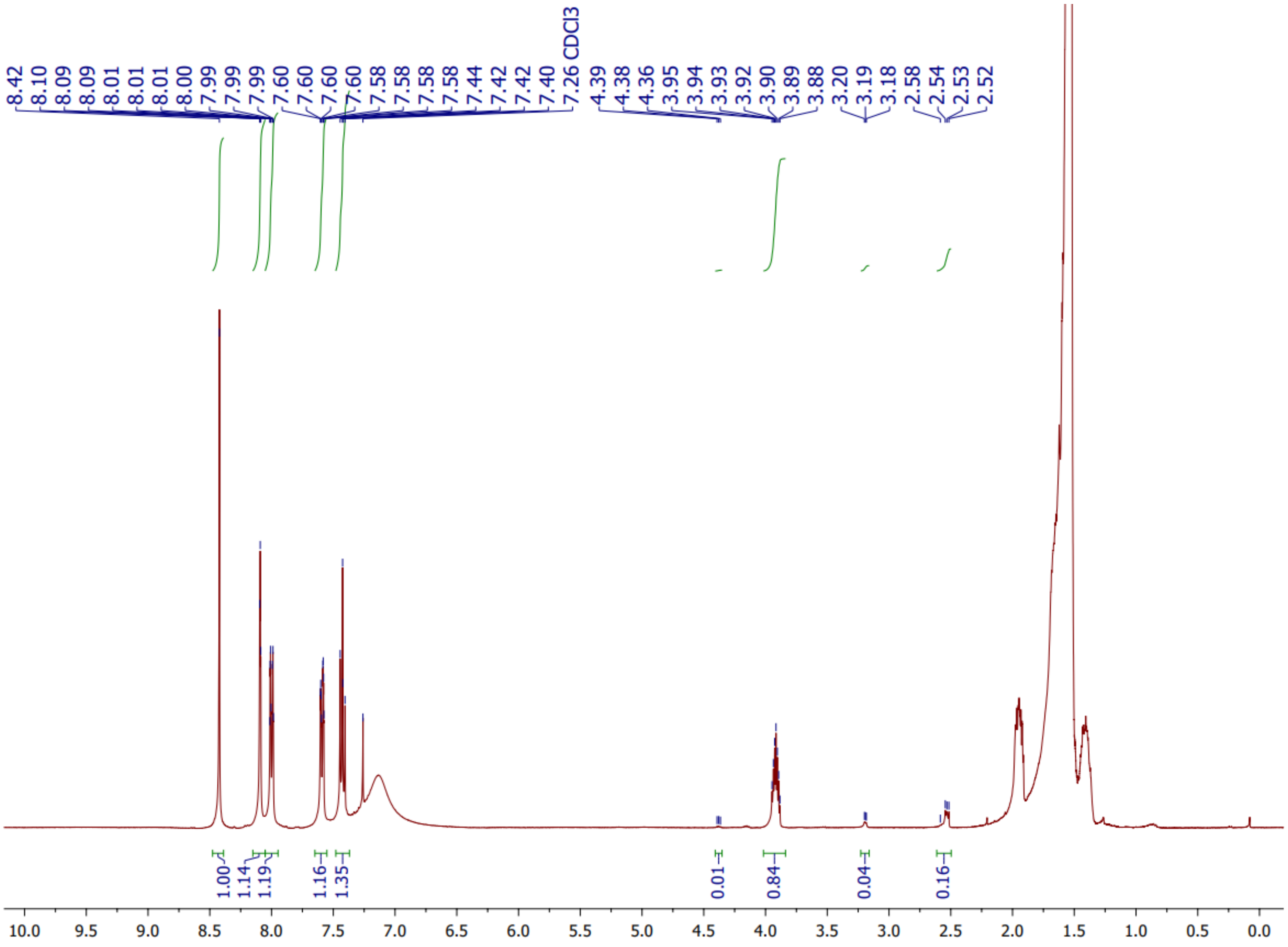

^{1}H NMR of the crude reaction mixture of oxidation of cycloheptane 18 with *m*CPBA 5 in NFTB.

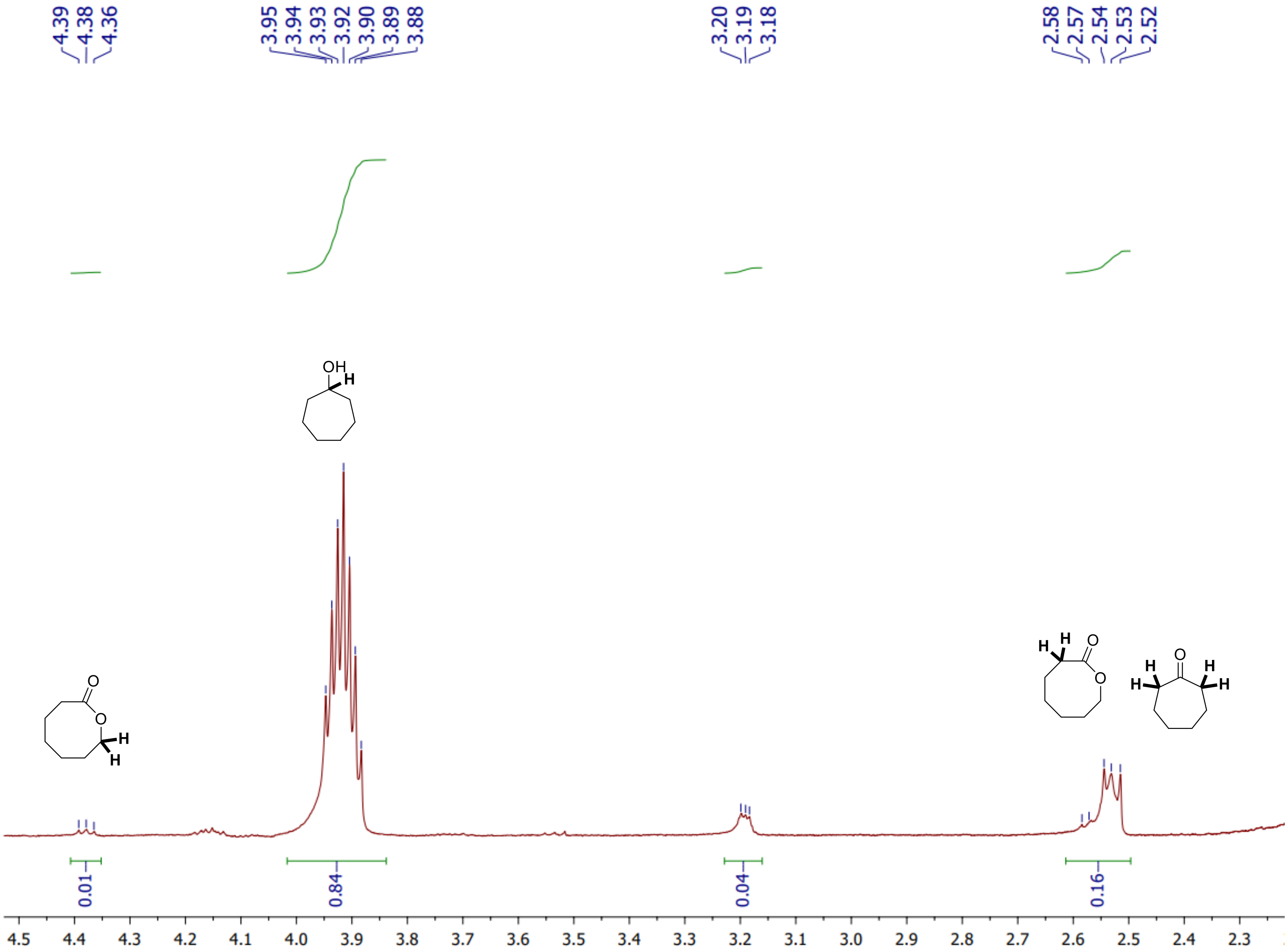

Compound Number	Structure	δ ppm	Integration	Number protons	% yield
**21**	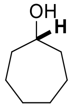	3.92	0.84	1	84
**24**	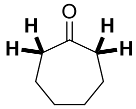	2.53	0.16-0.01	4	4
**27**	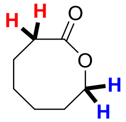	2.57<br>4.38	0.01<br>Overlapping	2	1

^{1}H NMR of the crude reaction mixture of oxidation of cyclooctane 19 with *m*CPBA 5 in NFTB.

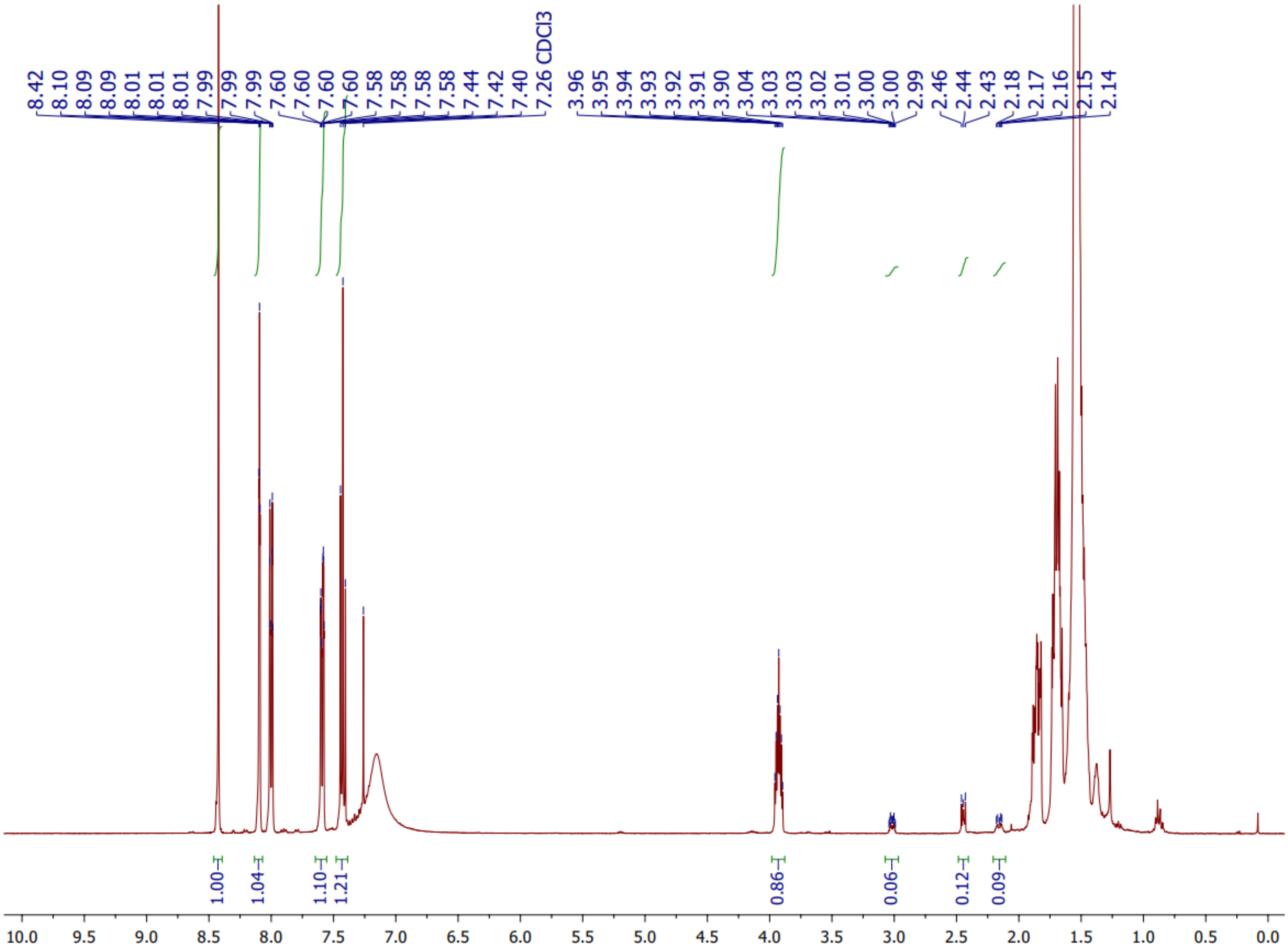

^{1}H NMR of the crude reaction mixture of oxidation of cyclooctane 19 with *m*CPBA 5 in NFTB.

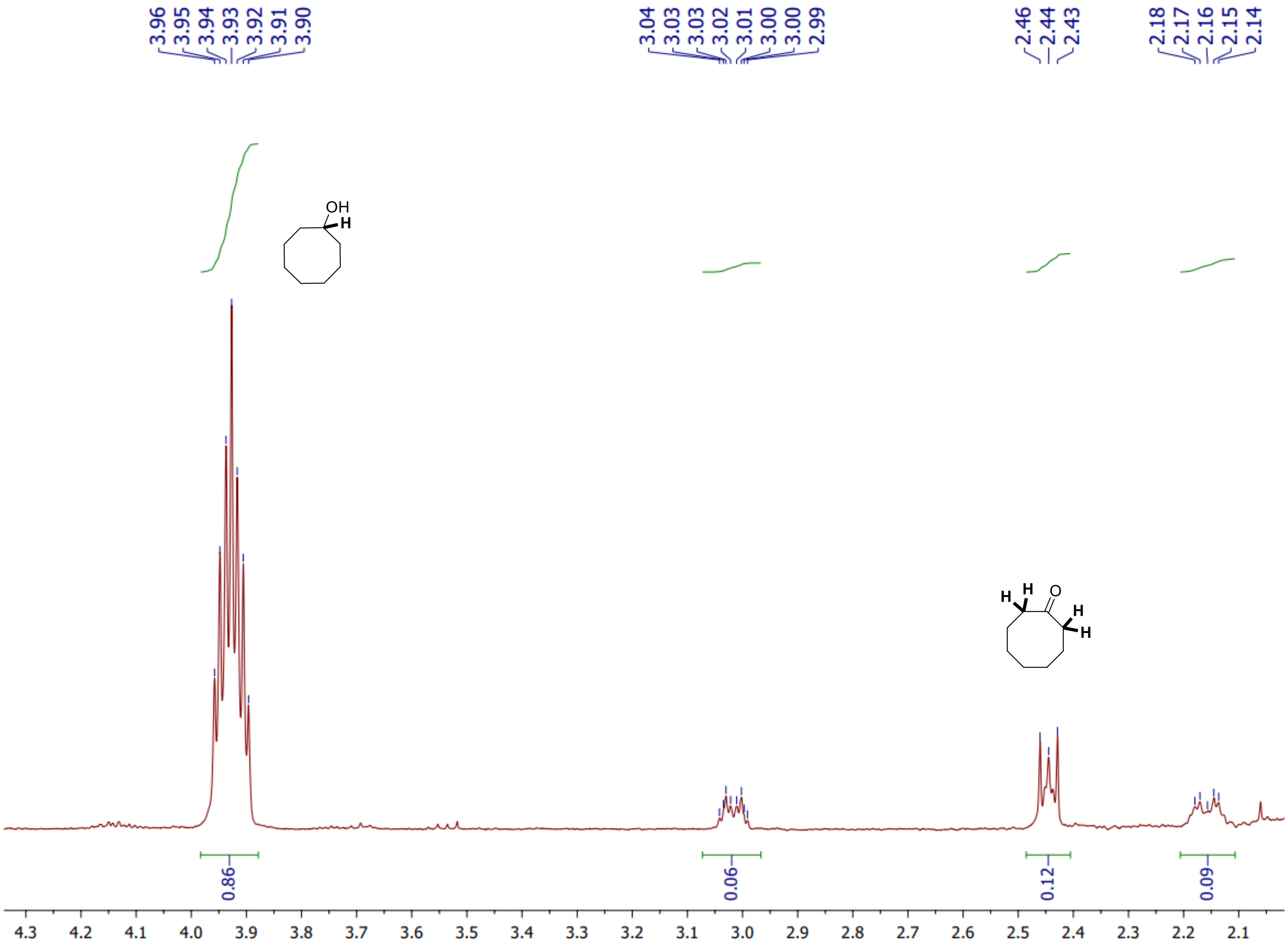

Compound Number	Structure	δ ppm	Integration	Number protons	% yield
**22**	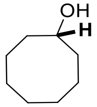	3.93	0.84	1	84
**25**	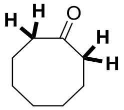	2.44	0.12	4	3
**28**	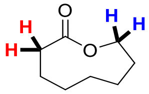	2.32<br>4.14	Presumed trace	2	1

^{1}H NMR of the crude reaction mixture of oxidation of *n*-pentane 29 with *m*CPBA 5 in NFTB.

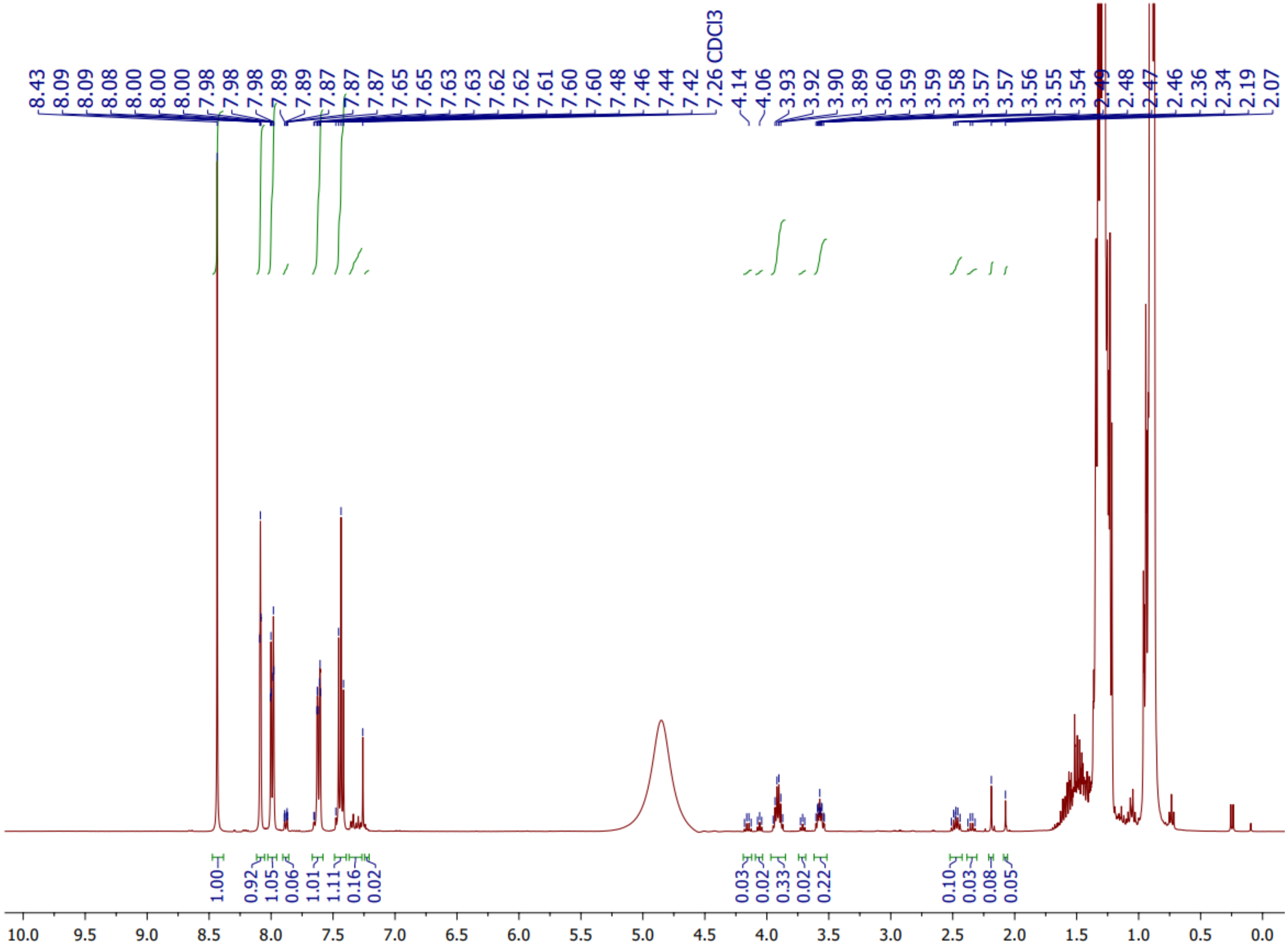

^{1}H NMR of the crude reaction mixture of oxidation of *n*-pentane 29 with *m*CPBA 5 in NFTB.

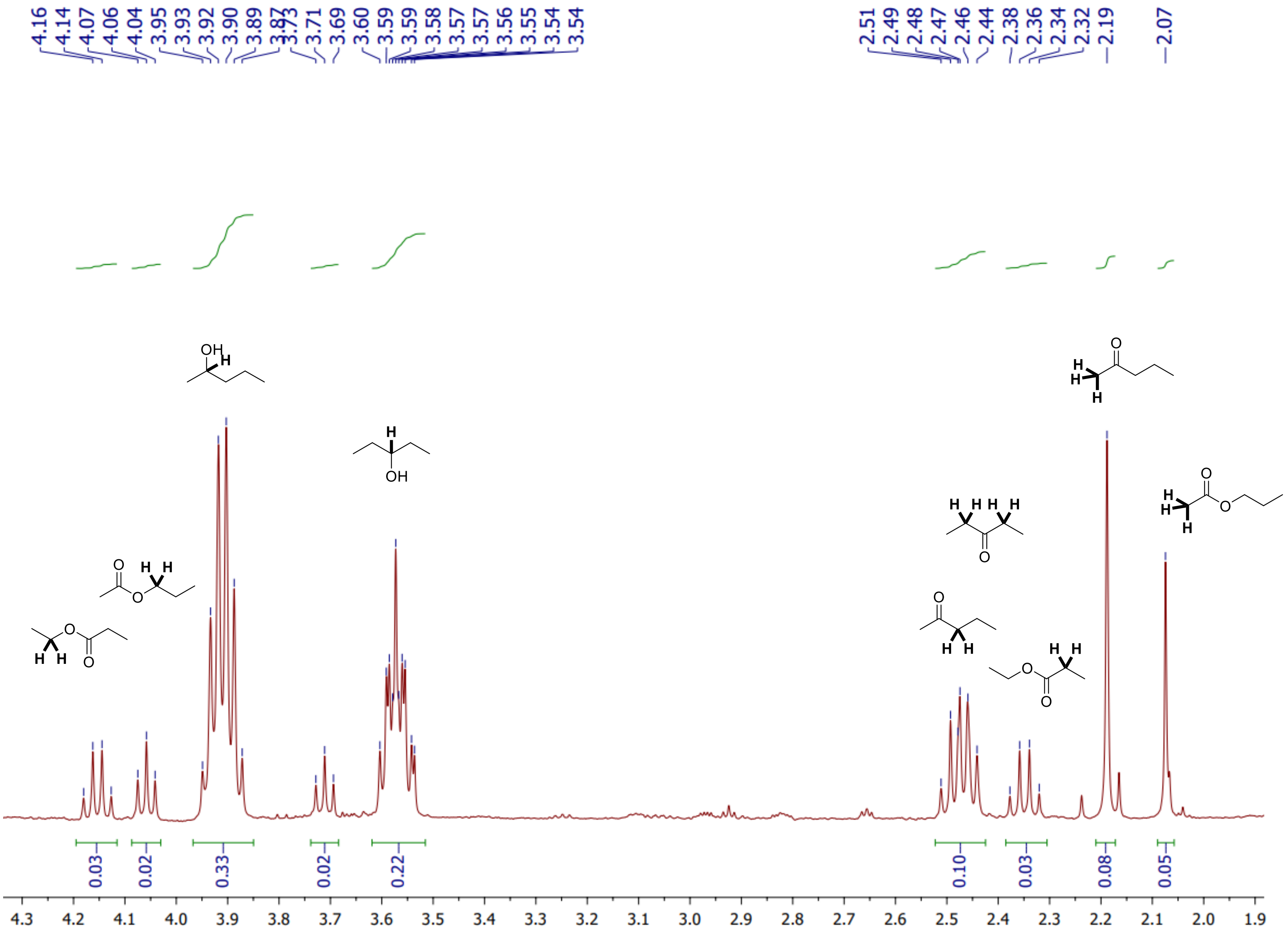

Compound Number	Structure	δ ppm	Integration	Number protons	% yield
**30**	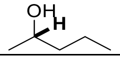	3.91	0.33	1	33
**32**	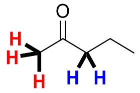	2.19<br>2.47	0.08<br>0.10-(0.08/3*2)	3<br>2	2<br>2
**34**	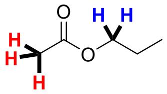	2.07<br>4.06	0.05<br>0.03	3<br>2	2<br>2
**31**	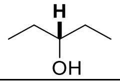	3.57	0.22	1	22
**33**	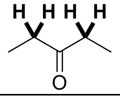	2.47	0.10-(0.08/3*2)	4	1
**35**	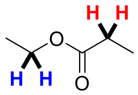	2.35<br>4.13	0.03<br>0.03	2<br>2	2<br>2

^{1}H NMR of the crude reaction mixture of oxidation of *n*-hexane 36 with *m*CPBA 5 in NFTB.

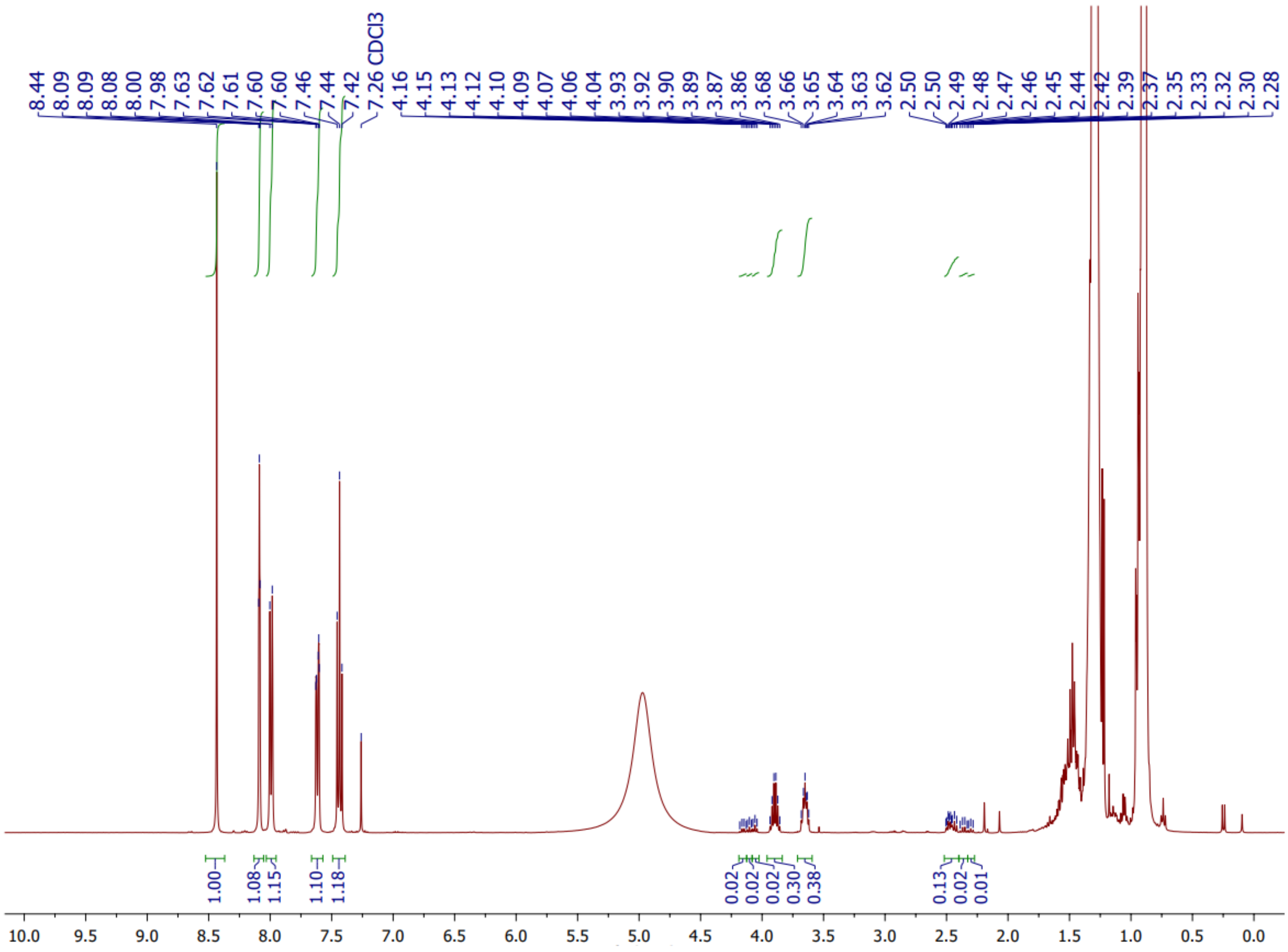

^{1}H NMR of the crude reaction mixture of oxidation of *n*-hexane 36 with *m*CPBA 5 in NFTB.

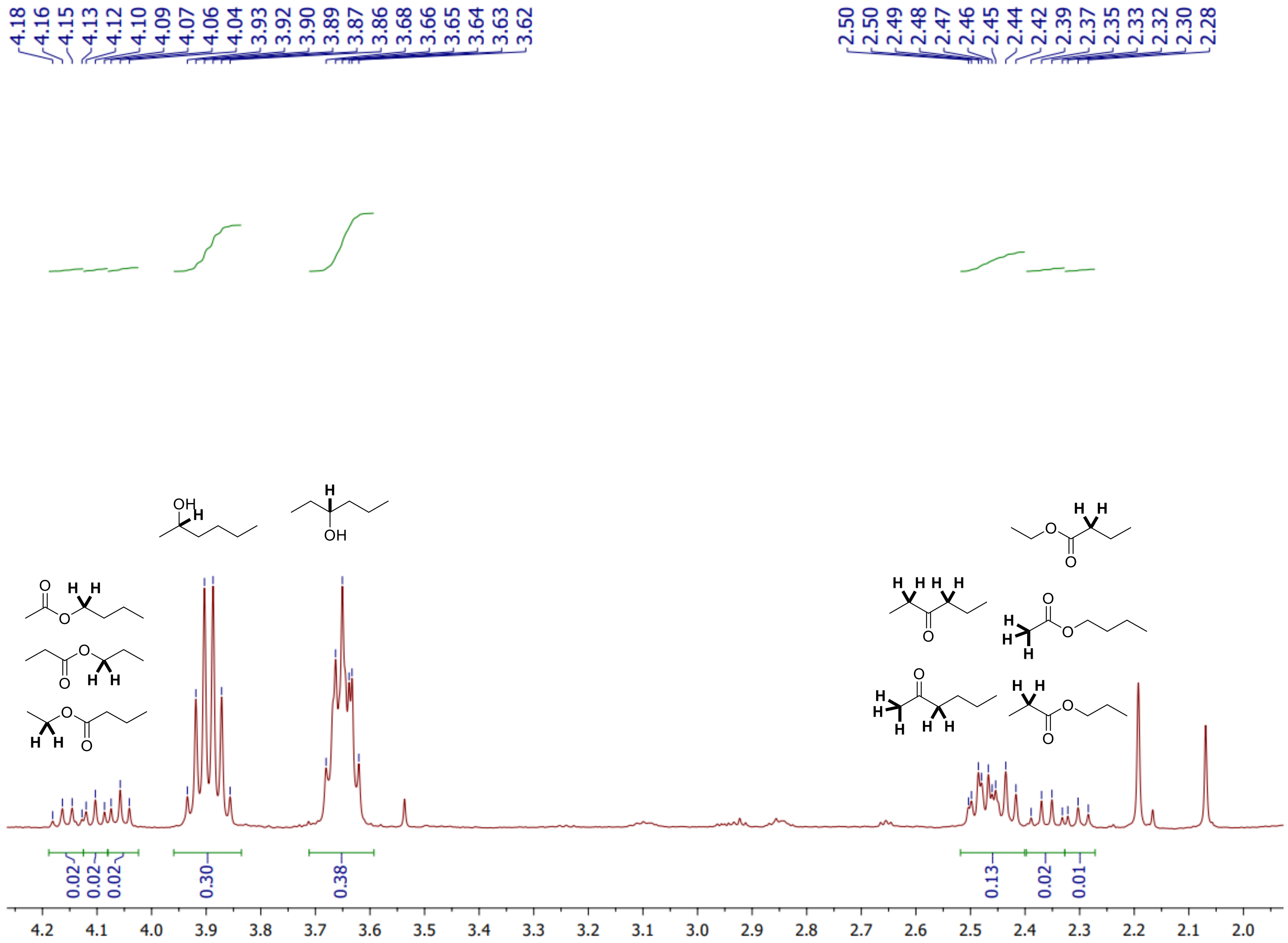

Compound Number	Structure	δ ppm	Integration	Number protons	% yield
**37**	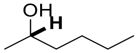	3.90	0.30	1	30
**39**	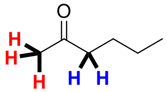	2.42-2.50	0.13	2	1
**41**	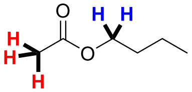	4.06	0.02	3<br>2	1<br>2
**38**	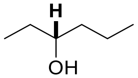	3.66	0.38	1	38
**40**	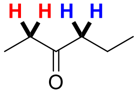	2.42-2.50	0.13	2<br>2	1
**42**	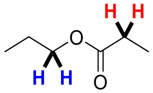	2.36<br>4.10	0.02<br>0.02	2<br>2	1
**43**	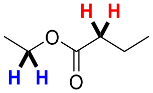	2.30<br>4.15	0.01<br>0.02	2<br>2	1

Yield for 39 and 40 was averaged out using this calculation: (0.13/6)/2

^{1}H NMR of the crude reaction mixture of oxidation of 2,2-dimethylpentane 44 with *m*CPBA 5 in NFTB.

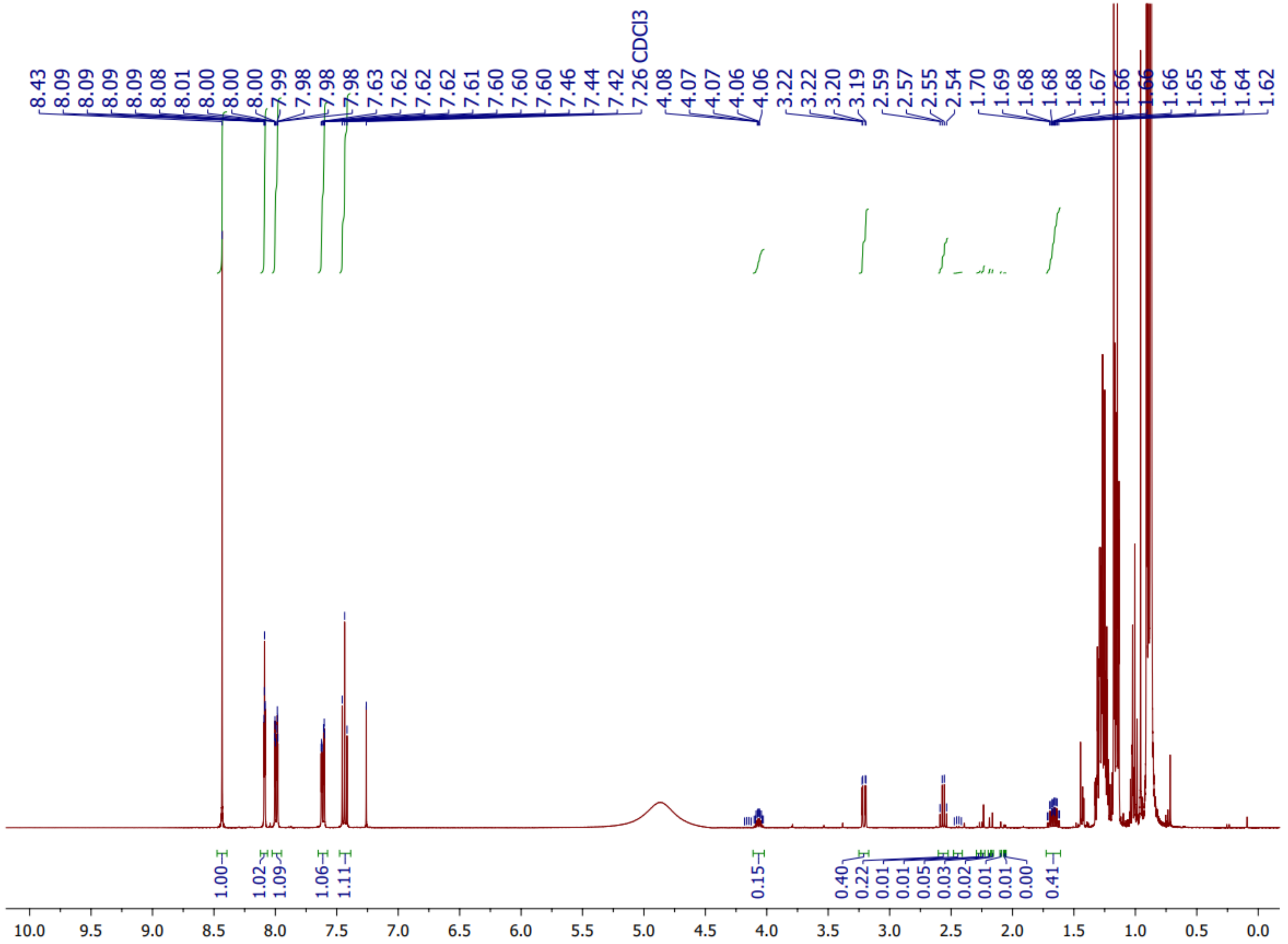

^{1}H NMR of the crude reaction mixture of oxidation of 2,2-dimethylpentane 44 with *m*CPBA 5 with NFTB.

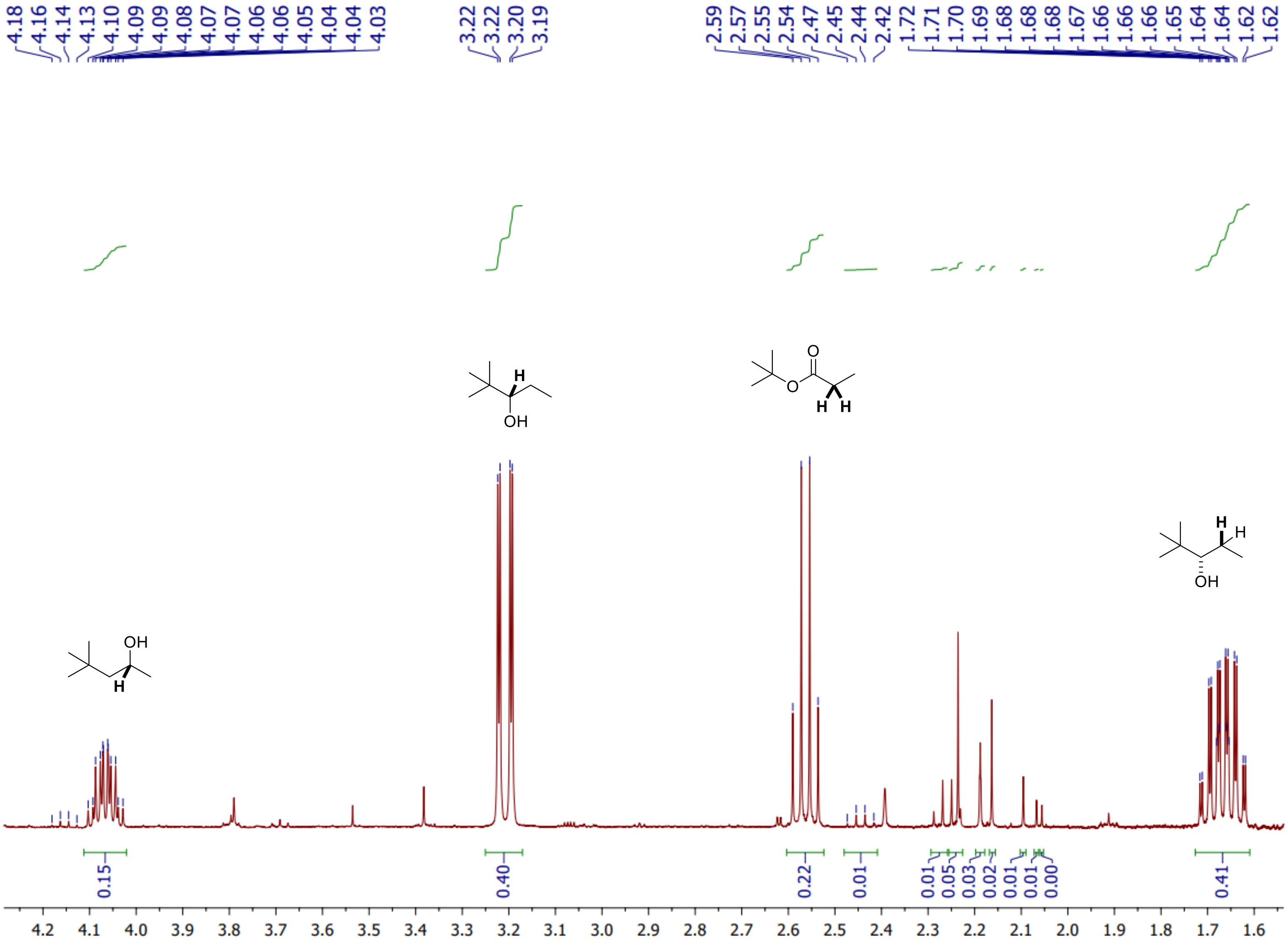

Compound Number	Structure	δ ppm	Integration	Number protons	% yield
**45**	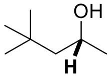	4.07	0.15	1	15
**47**	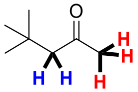	2.26<br>2.19	0.02<br>0.05-0.01=0.04	3<br>2	1
**46**	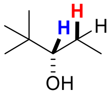	3.21<br>1.68	0.40<br>0.41	1<br>1	40
**51**	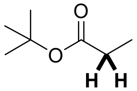	2.56	0.22	2	11
**52**	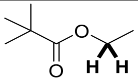	2.44	0.01	2	trace

^{1}H NMR of the crude reaction mixture of oxidation of methylcyclohexane 53 with *m*CPBA 5 in NFTB.

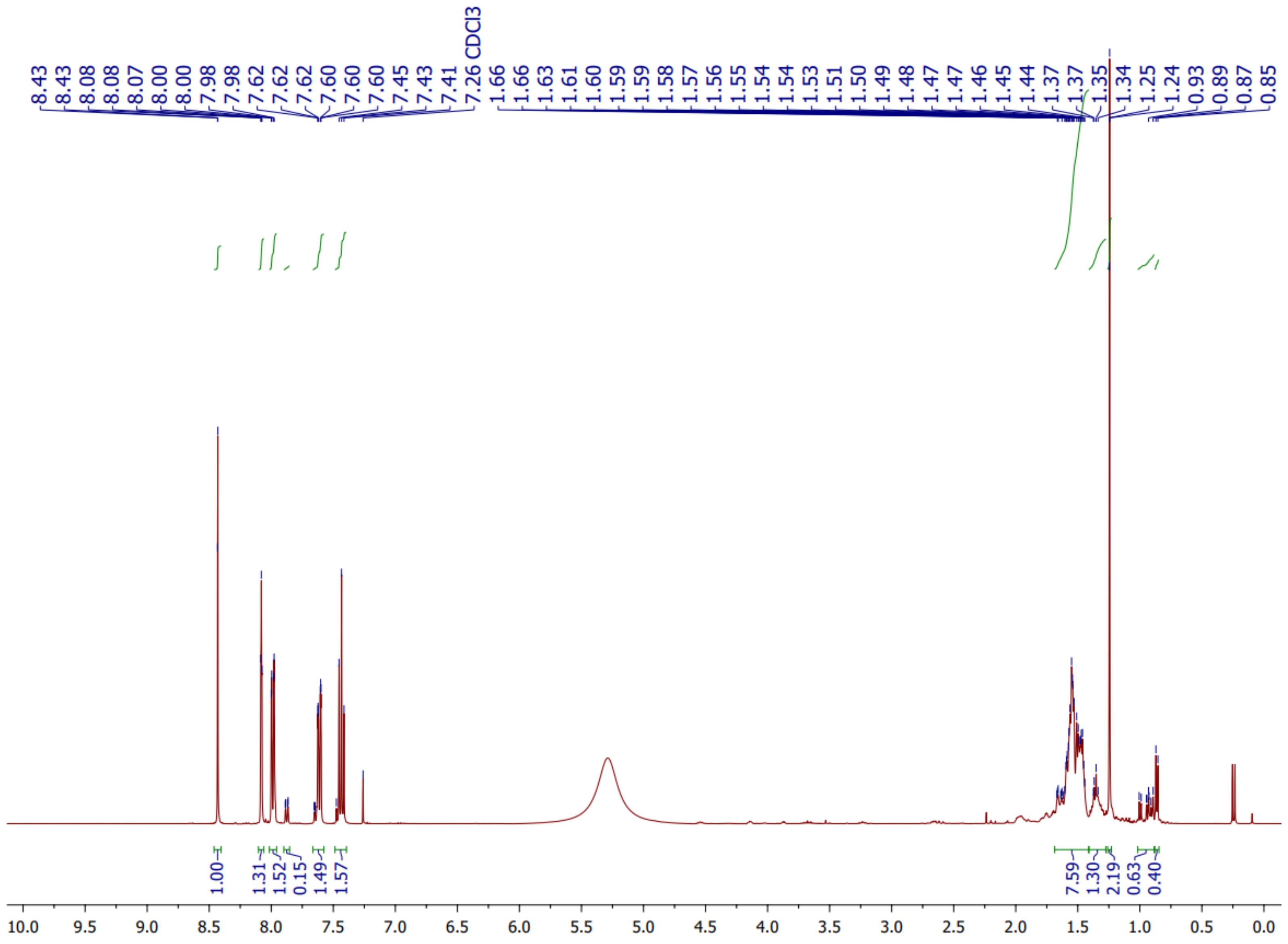

^{1}H NMR of the crude reaction mixture of oxidation of methylcyclohexane 53 with *m*CPBA 5 in NFTB.

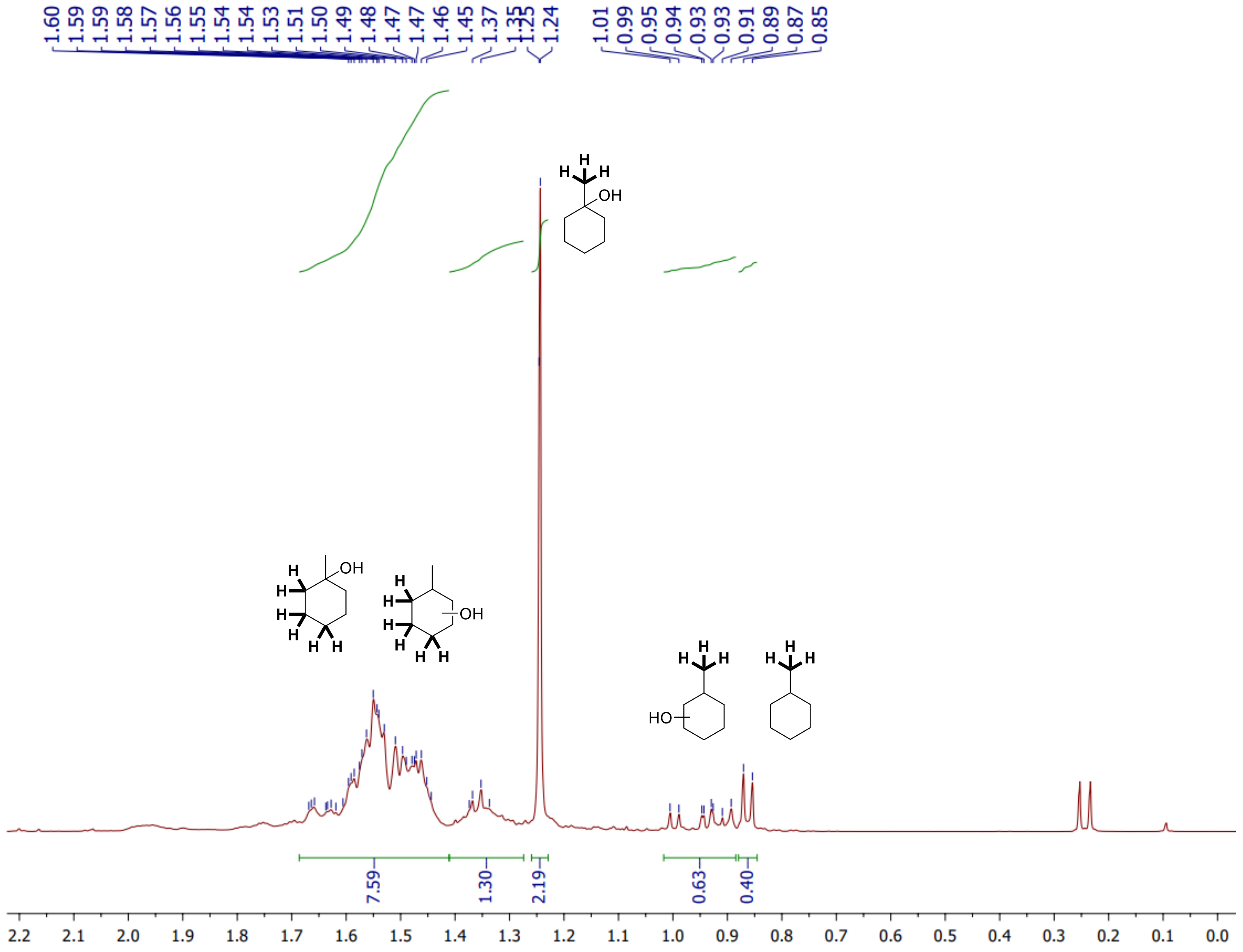

Compound Number	Structure	δ ppm	Integration	Number protons	% yield
**54**	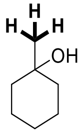	1.25	2.19	3	73

^{1}H NMR of the crude reaction mixture of oxidation of 2-methylhexane 55 with *m*CPBA 5 in NFTB.

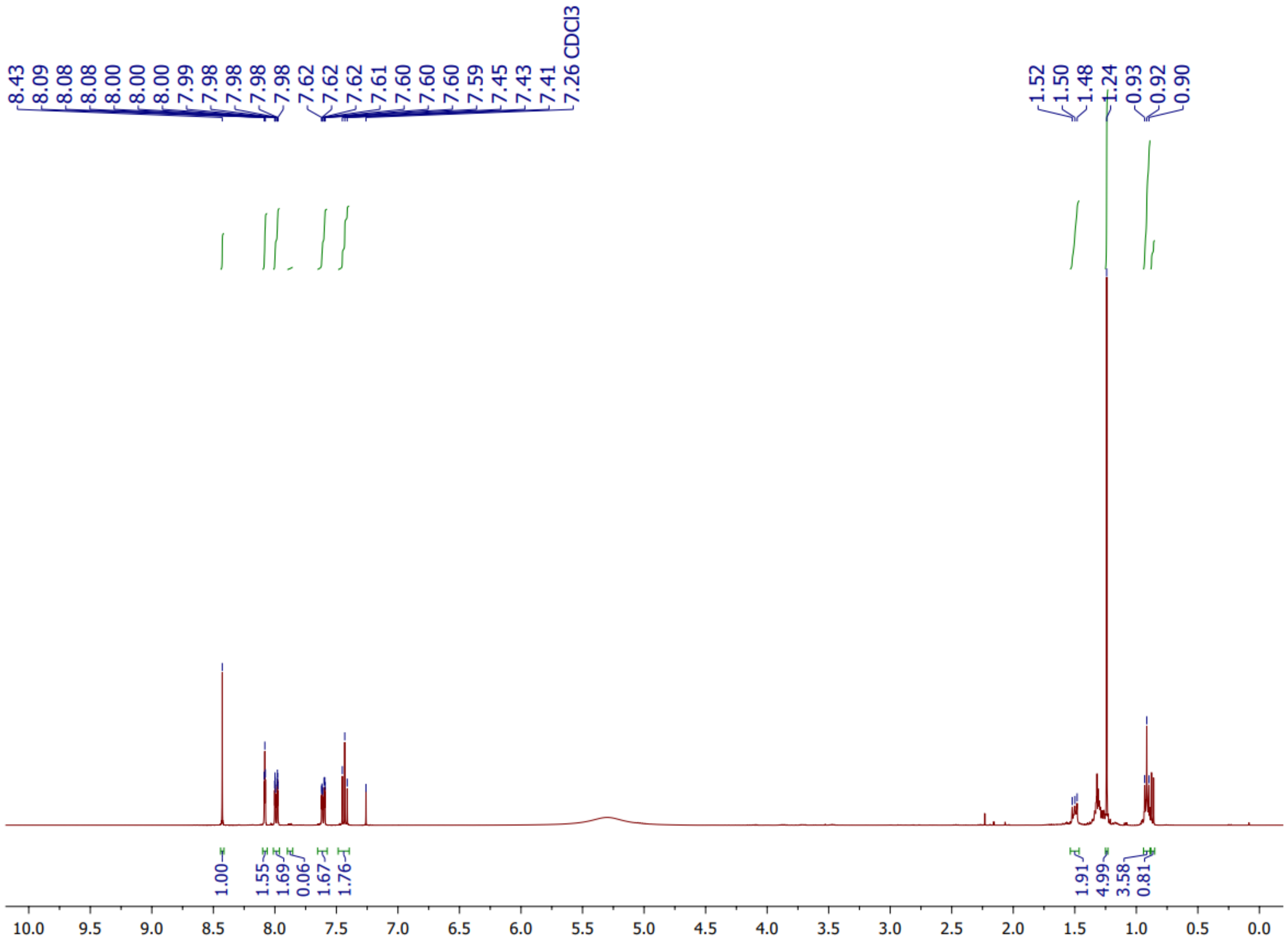

^{1}H NMR of the crude reaction mixture of oxidation of 2-methylhexane 55 with *m*CPBA 5 in NFTB.

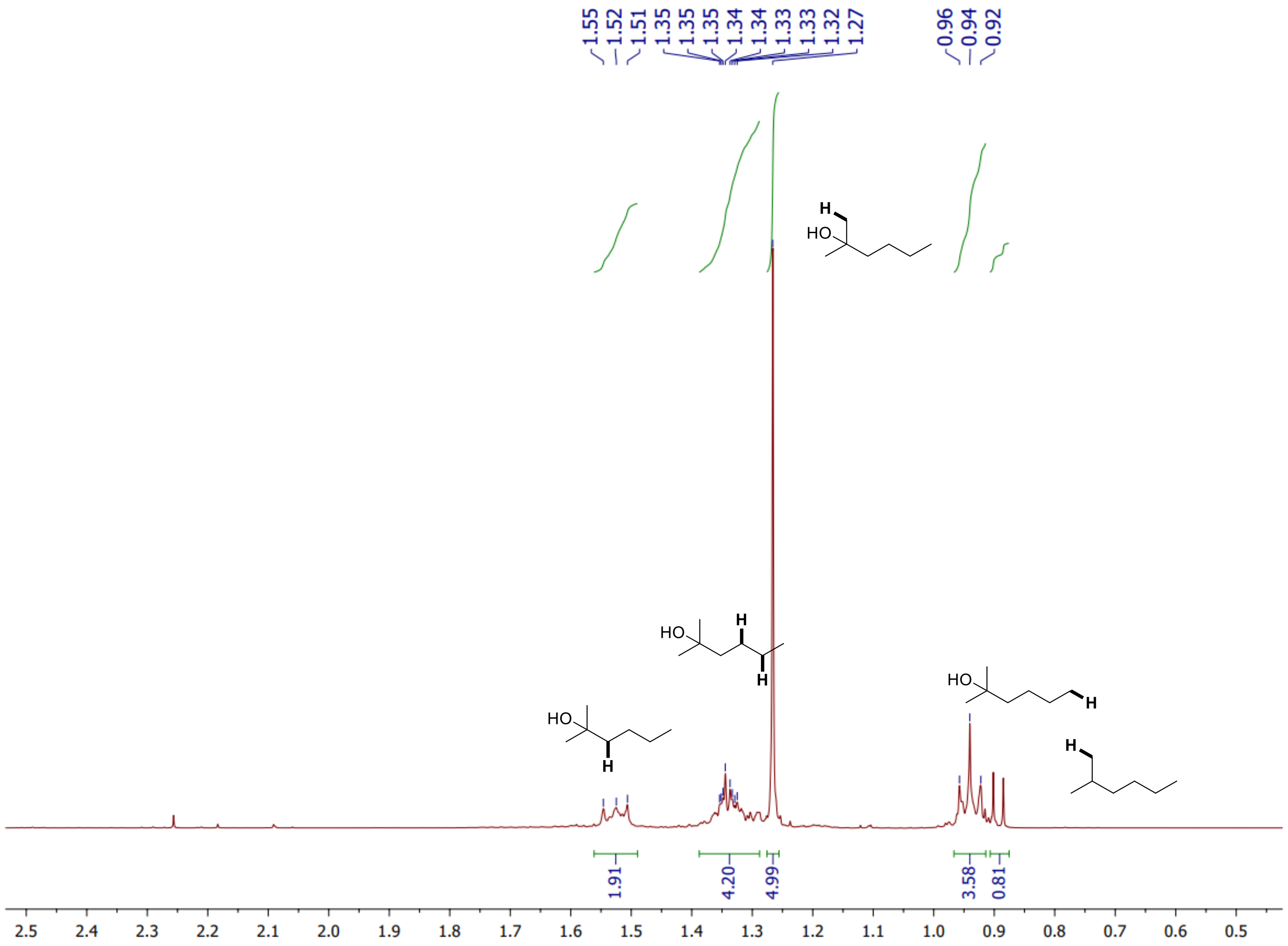

Compound Number	Structure	δ ppm	Integration	Number protons	% yield
**56**	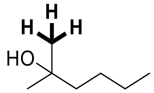	1.27	4.99	6	83

4.1 Competition oxidation experiments

^{1}H NMR of the crude reaction mixture of oxidation of cyclohexane 4 and cyclopentane 17 with *m*CPBA 5 in NFTB.

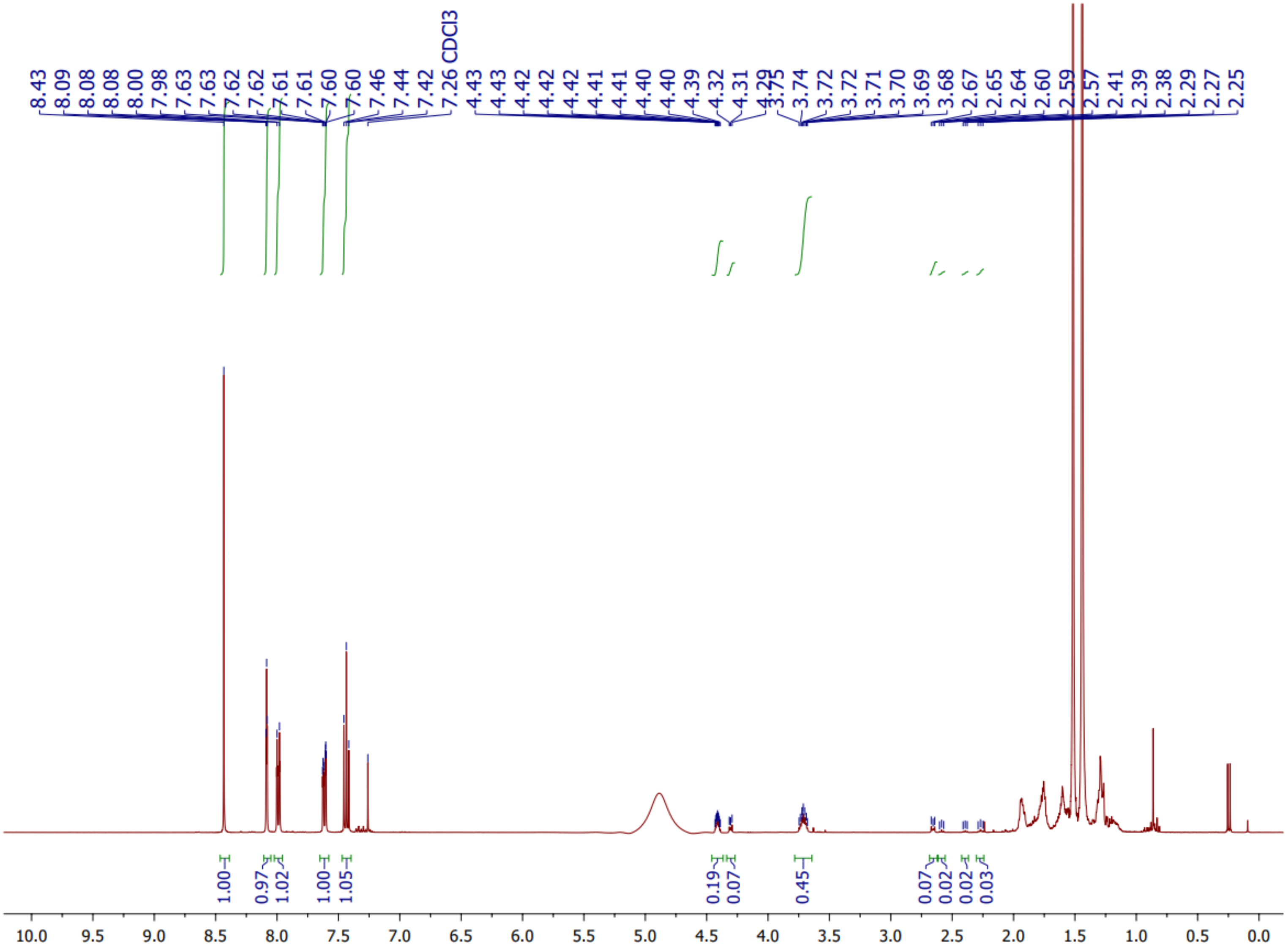

^{1}H NMR of the crude reaction mixture of oxidation of cyclohexane 4 and cyclopentane 17 with *m*CPBA 5 in NFTB.

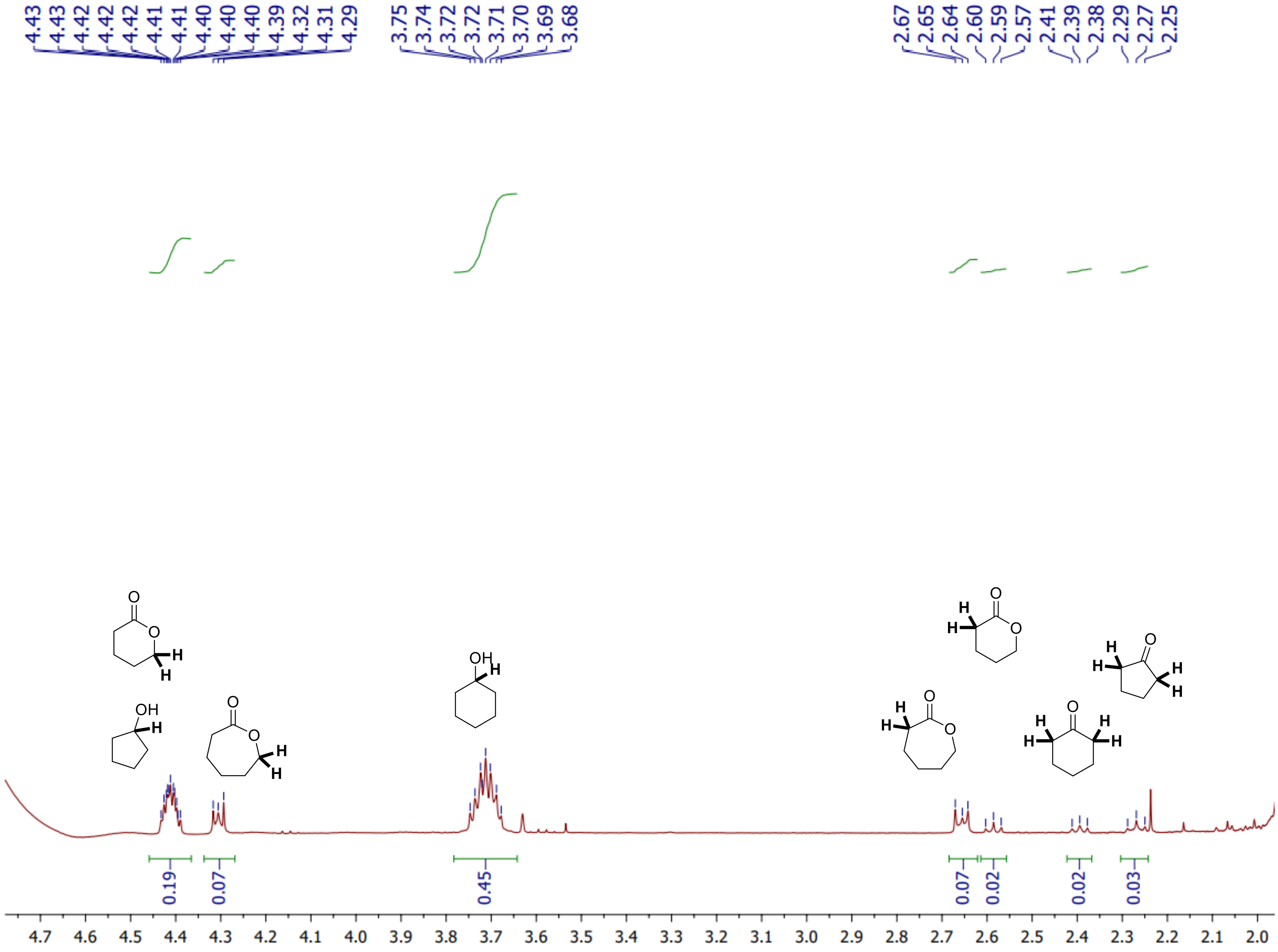

Compound Number	Structure	δ ppm	Integration	Number protons	% yield
**20**	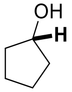	4.38	0.19-0.02=0.17	1	17
**23**	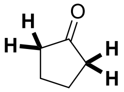	2.27	0.03	4	1
**26**	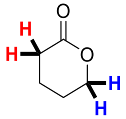	2.57<br>4.36	0.02<br>Overlapping	2	1
**6**	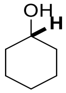	3.72	0.45	1	45
**7**	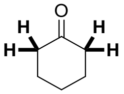	2.39	0.02	4	0.5
**8**	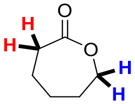	2.65<br>4.31	0.07<br>0.07	2	3

^{1}H NMR of the crude reaction mixture of oxidation of cyclohexanol 6 and methylcyclohexane 53 with *m*CPBA 5 in NFTB.

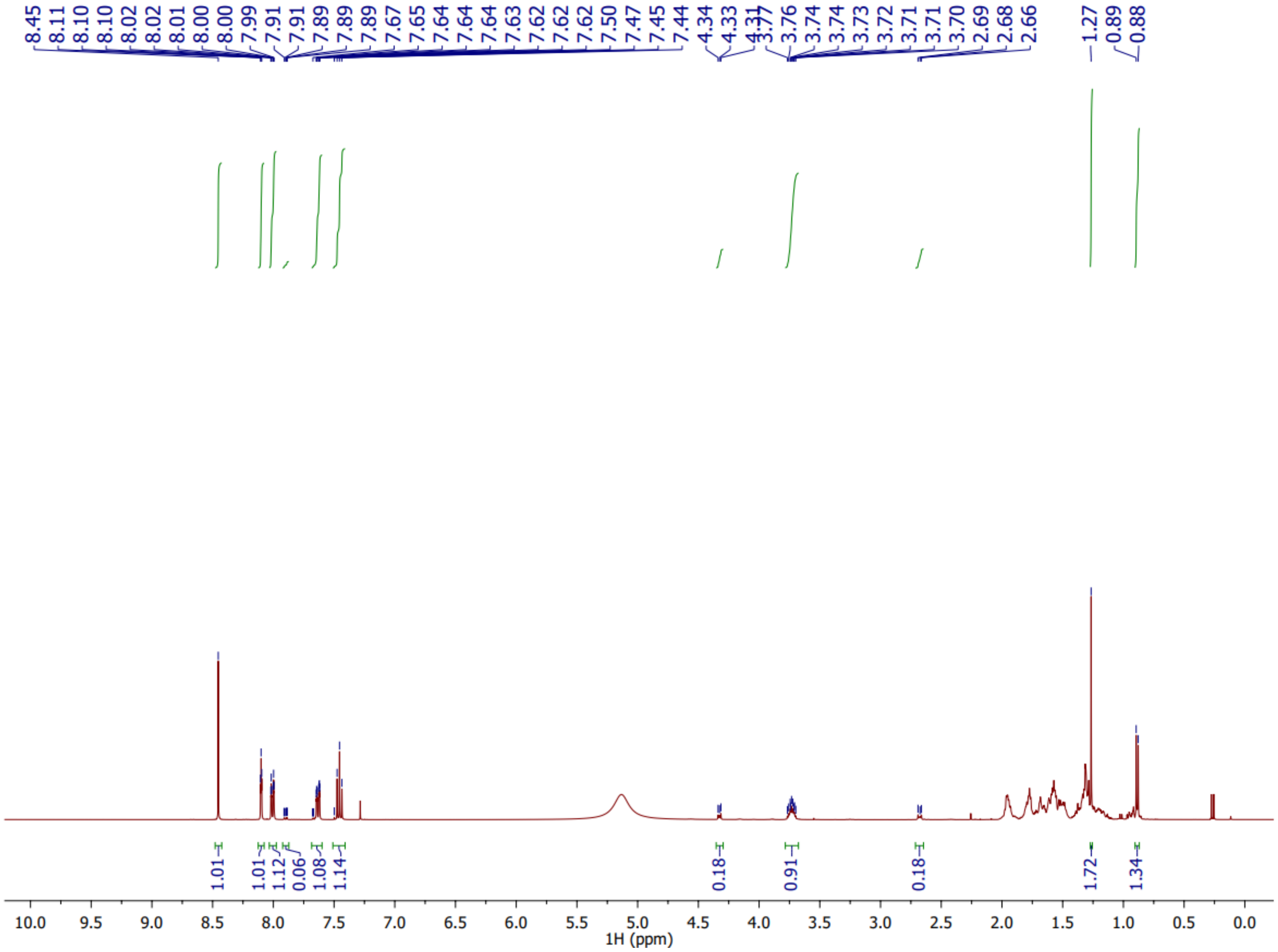

^{1}H NMR of the crude reaction mixture of oxidation of cyclohexanol 6 and methylcyclohexane 53 with *m*CPBA 5 in NFTB.

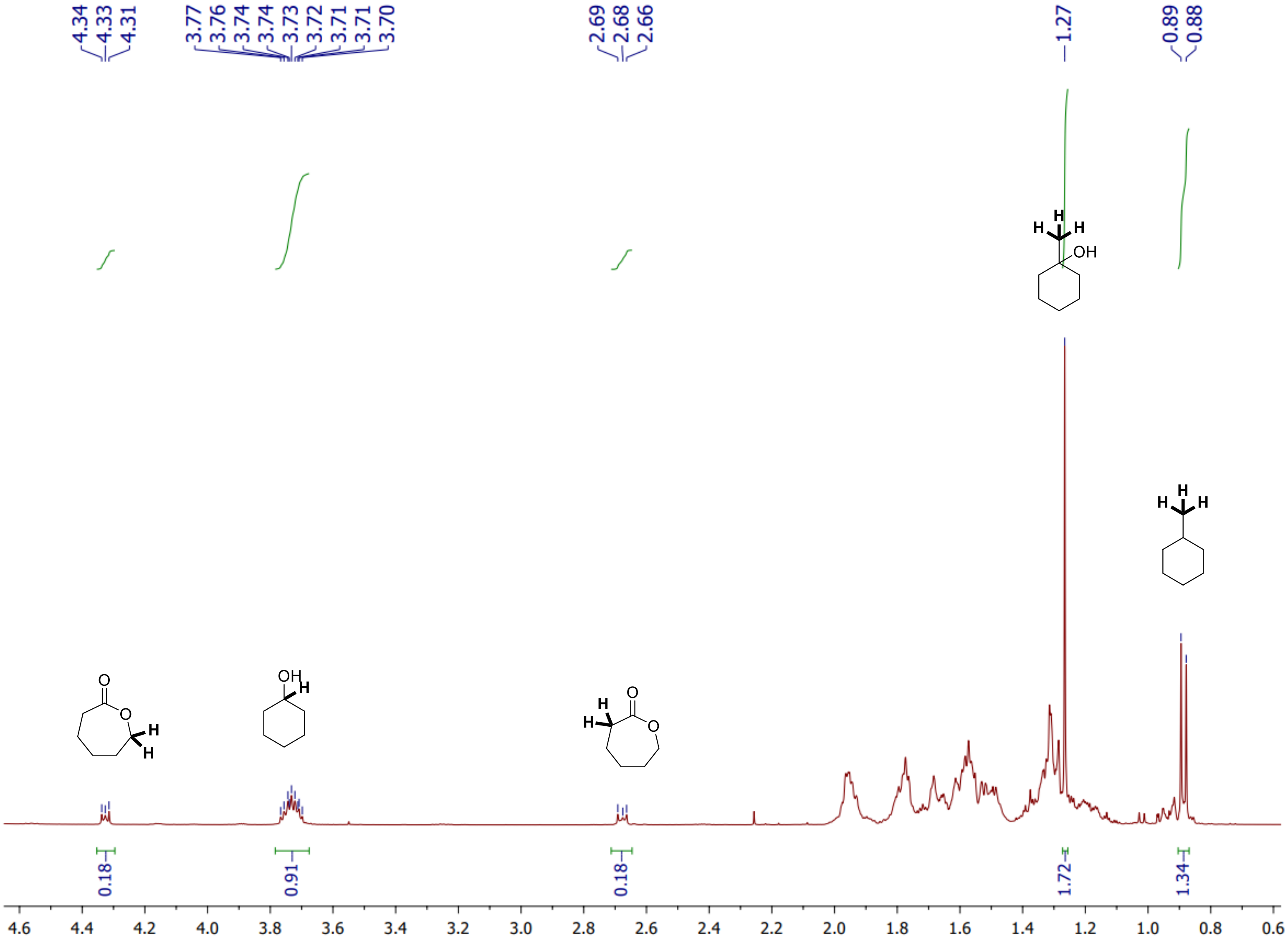

Compound Number	Structure	δ ppm	Integration	Number protons	% yield
**54**	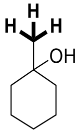	1.27	1.72	3	57
**6**	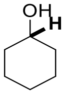	3.73	0.91	1	91
**7**	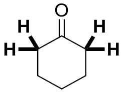	2.39	trace	4	trace
**8**	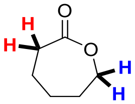	2.68<br>4.33	0.09<br>0.09	2	5

^{1}H NMR of the crude reaction mixture of oxidation of cyclohexanol 6 and methylcyclohexane 53 with *m*CPBA 5 in $CDCl_3$.

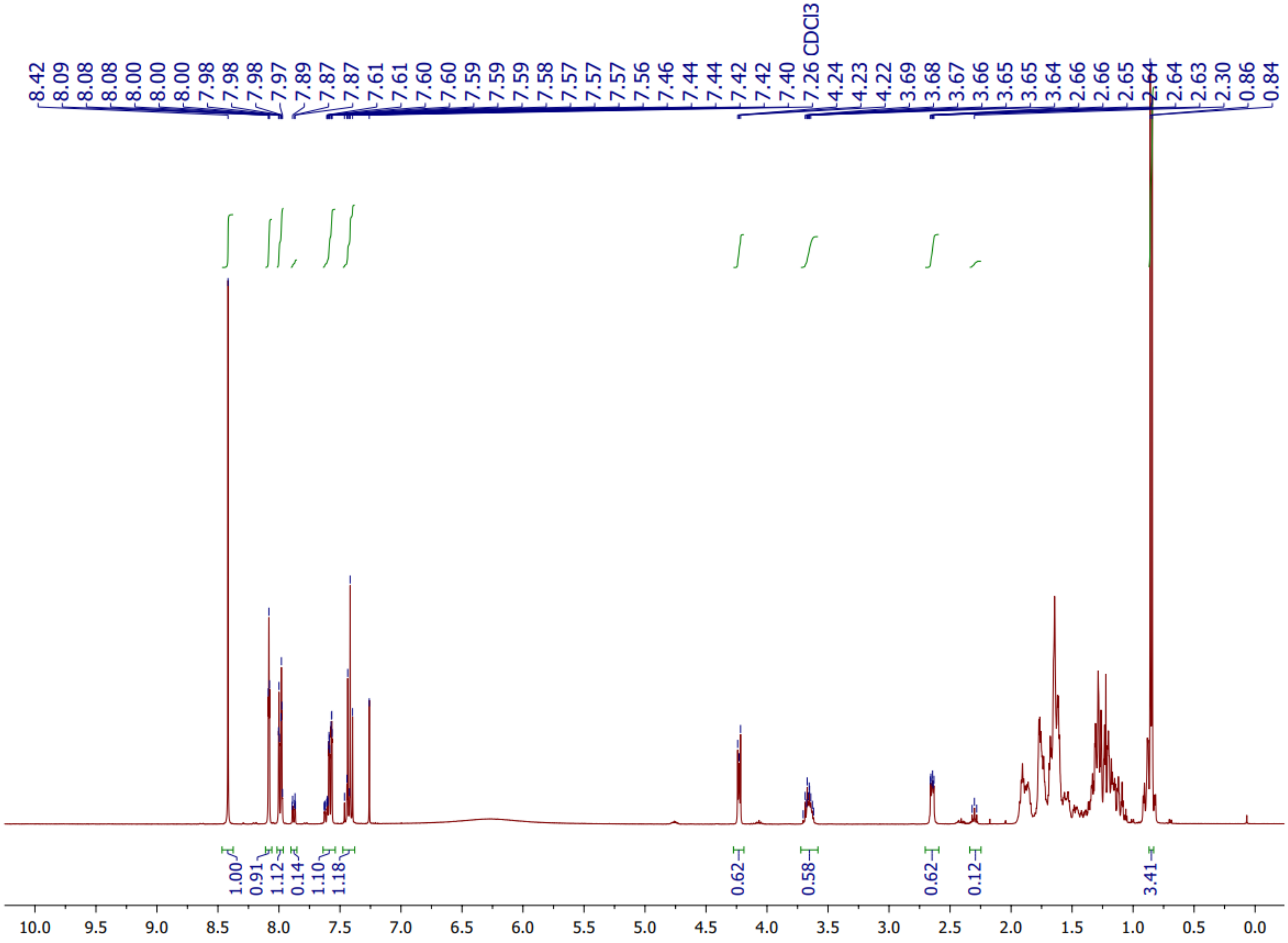

^{1}H NMR of the crude reaction mixture of oxidation of cyclohexanol 6 and methylcyclohexane 53 with *m*CPBA 5 in $CDCl_3$.

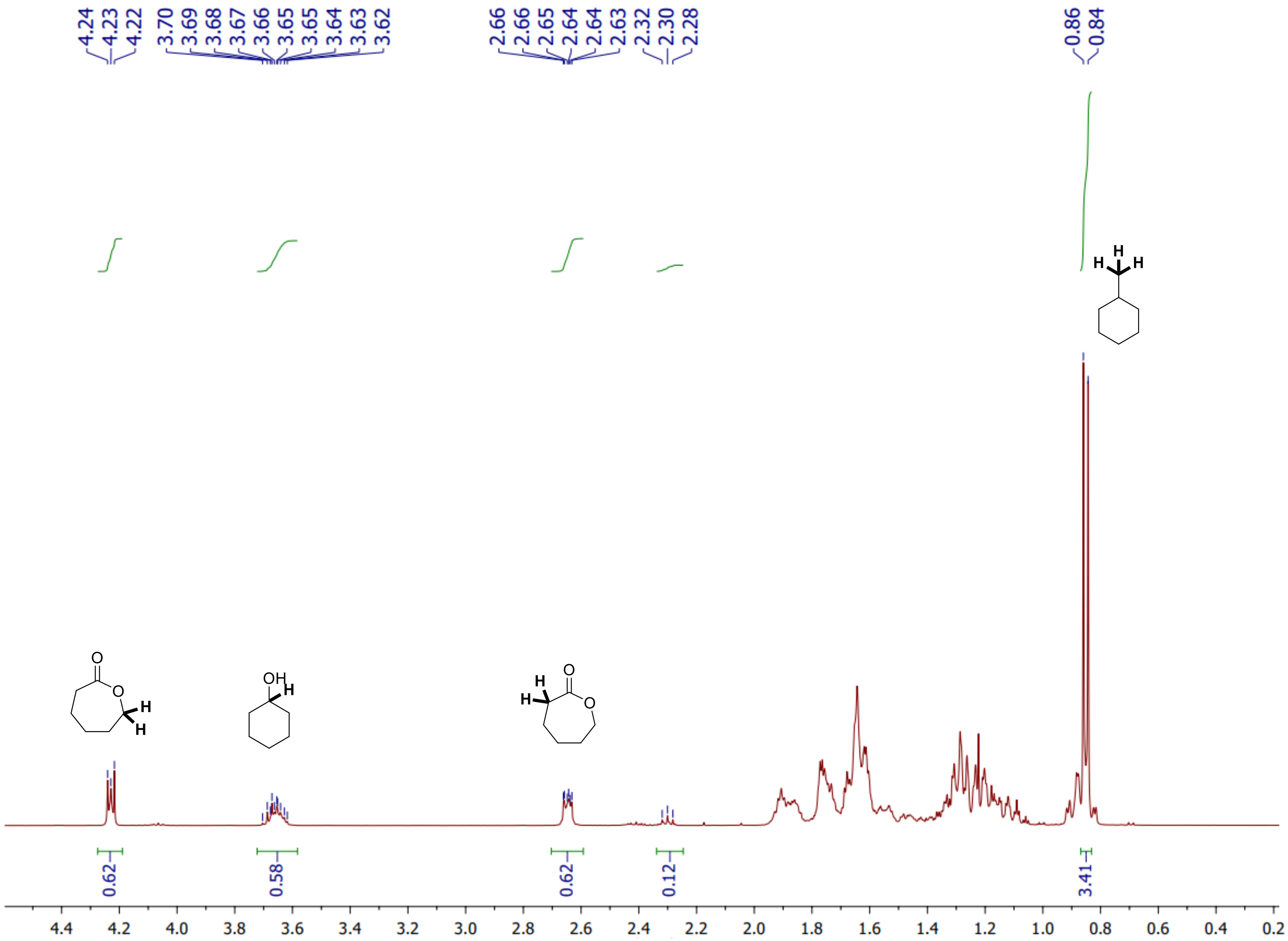

Compound Number	Structure	δ ppm	Integration	Number protons	% yield
**54**	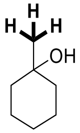	1.27	trace	3	trace
**6**	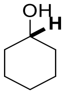	3.66	0.58	1	58
**7**	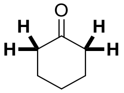	2.30	0.12	4	3
**8**	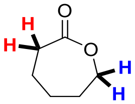	2.65<br>4.23	0.62<br>0.62	2	31

4.2 Competition experiment between cyclohexane and cyclopentane.

$$\frac{[CyOH]}{[CpOH]} = \frac{k_{CyOH}}{k_{CpOH}}$$

$$\frac{\frac{n(CyOH)}{L}}{\frac{n(CpOH)}{L}} = \frac{\frac{k_B T}{h} e^{-\frac{\Delta G^{\ddagger}_{CyOH}}{RT}}}{\frac{k_B T}{h} e^{-\frac{\Delta G^{\ddagger}_{CpOH}}{RT}}}$$

$$\frac{n(CyOH)}{n(CpOH)} = e^{\frac{-\Delta G^{\ddagger}_{CyOH} + \Delta G^{\ddagger}_{CpOH}}{RT}}$$

$$RT\left(\ln\left(n(CyOH)\right) - \ln\left(n(CyOH)\right)\right) = -\Delta G^{\ddagger}_{CyOH} + \Delta G^{\ddagger}_{CpOH}$$

The total yield of oxidation of cyclohexane was calculated as such:

45% cyclohexanol + 0.5% cyclohexanone + 3.5% caprolactone. n(CyOH) = 0.5 mmol * (45%+0.5%+3.5%) = 0.245 mmol

The total yield of oxidation of cyclopentane was calculated as such:

17% cyclopentanol + 0.75% cyclopentanone + 1% valerolactone. n(CpOH) = 0.5 mmol * (17% + 0.75% + 1%) = 0.094 mmol

^{1}H NMR of the crude reaction mixture of oxidation of 3-methylbutanoic acid 59 with *m*CPBA 5.

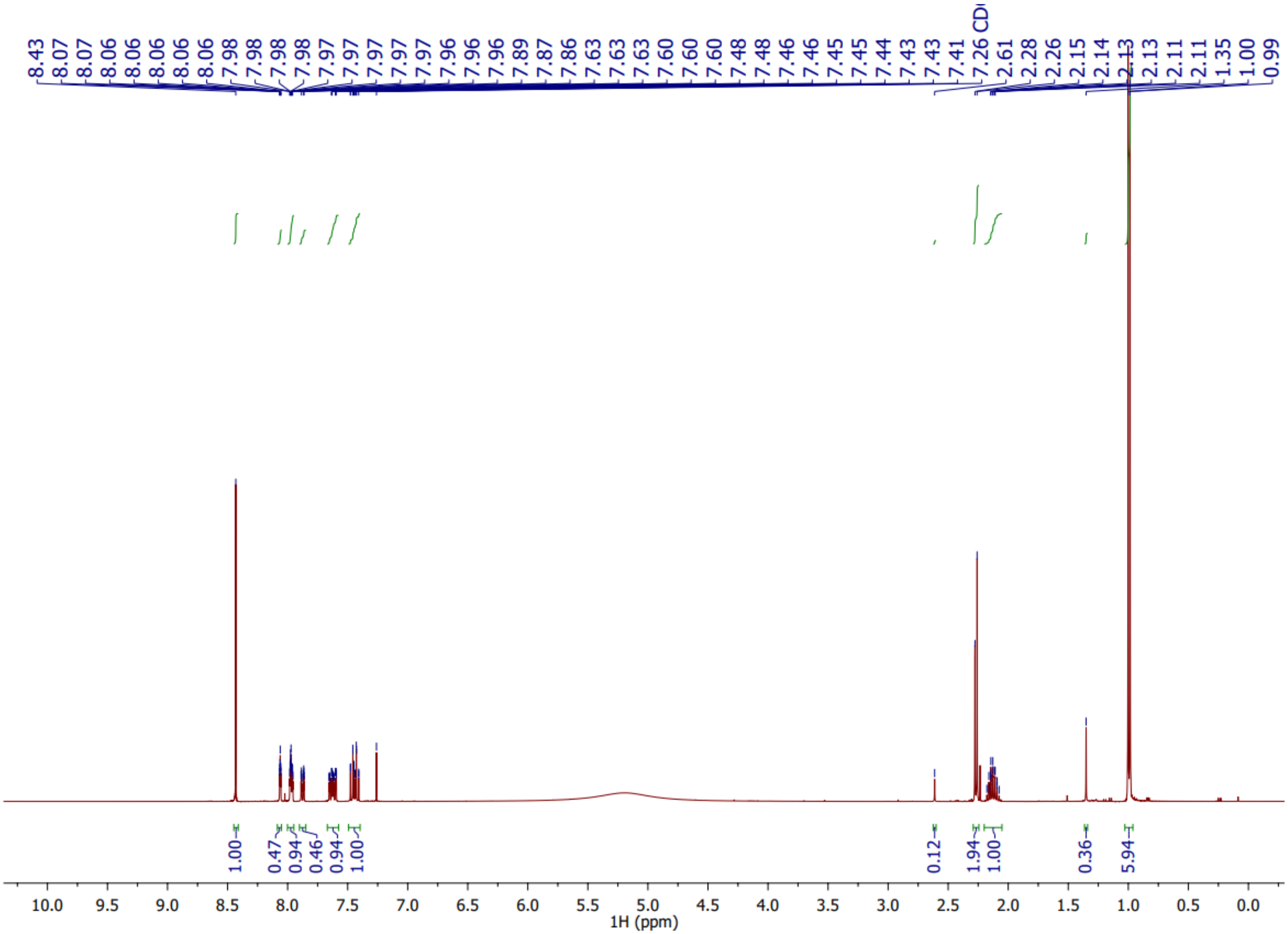

^{1}H NMR of the crude reaction mixture of oxidation of 3-methylbutanoic acid 59 with *m*CPBA 5.

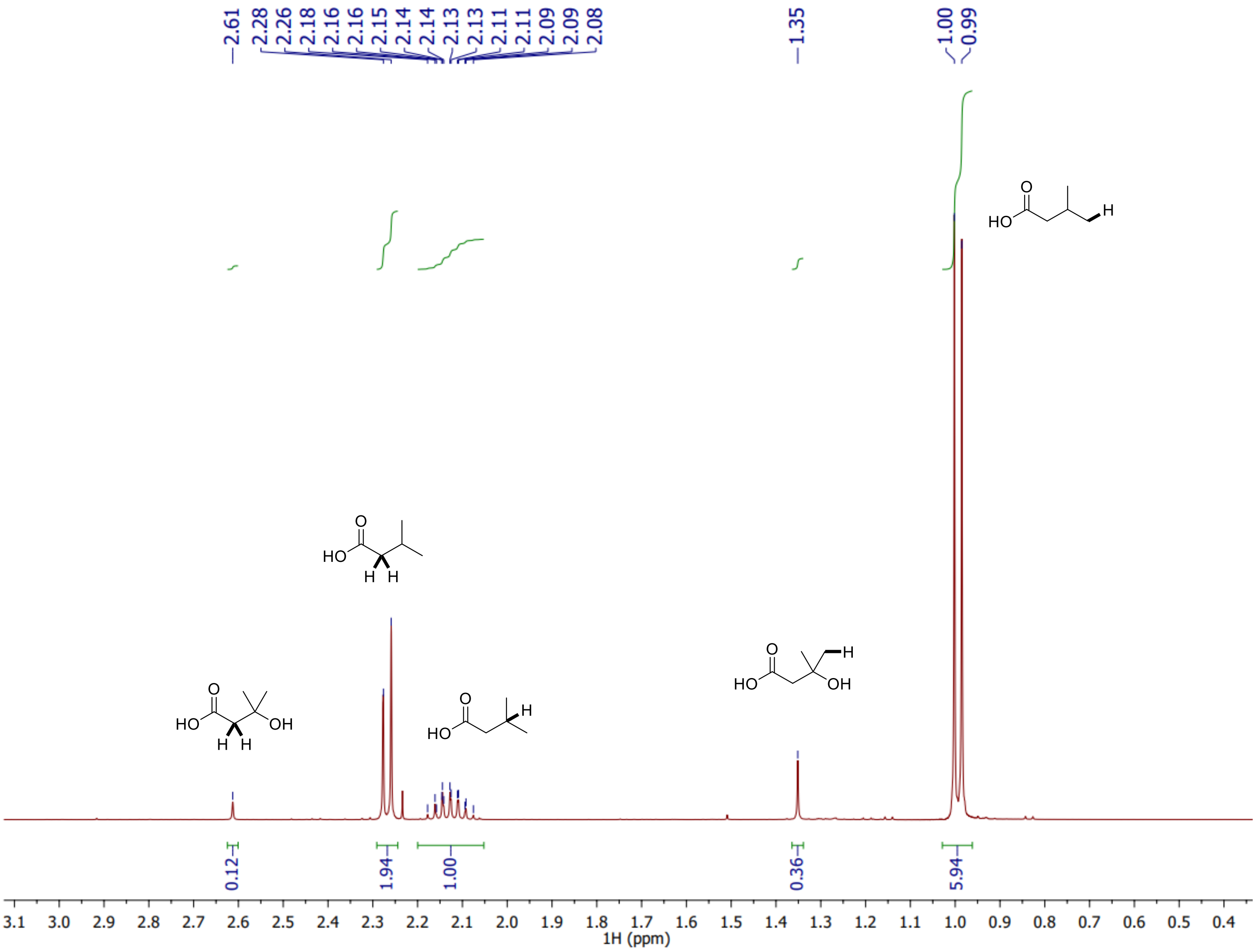

^{1}H NMR of the crude reaction mixture of oxidation of 4-methylpentanoic acid 60 with *m*CPBA 5.

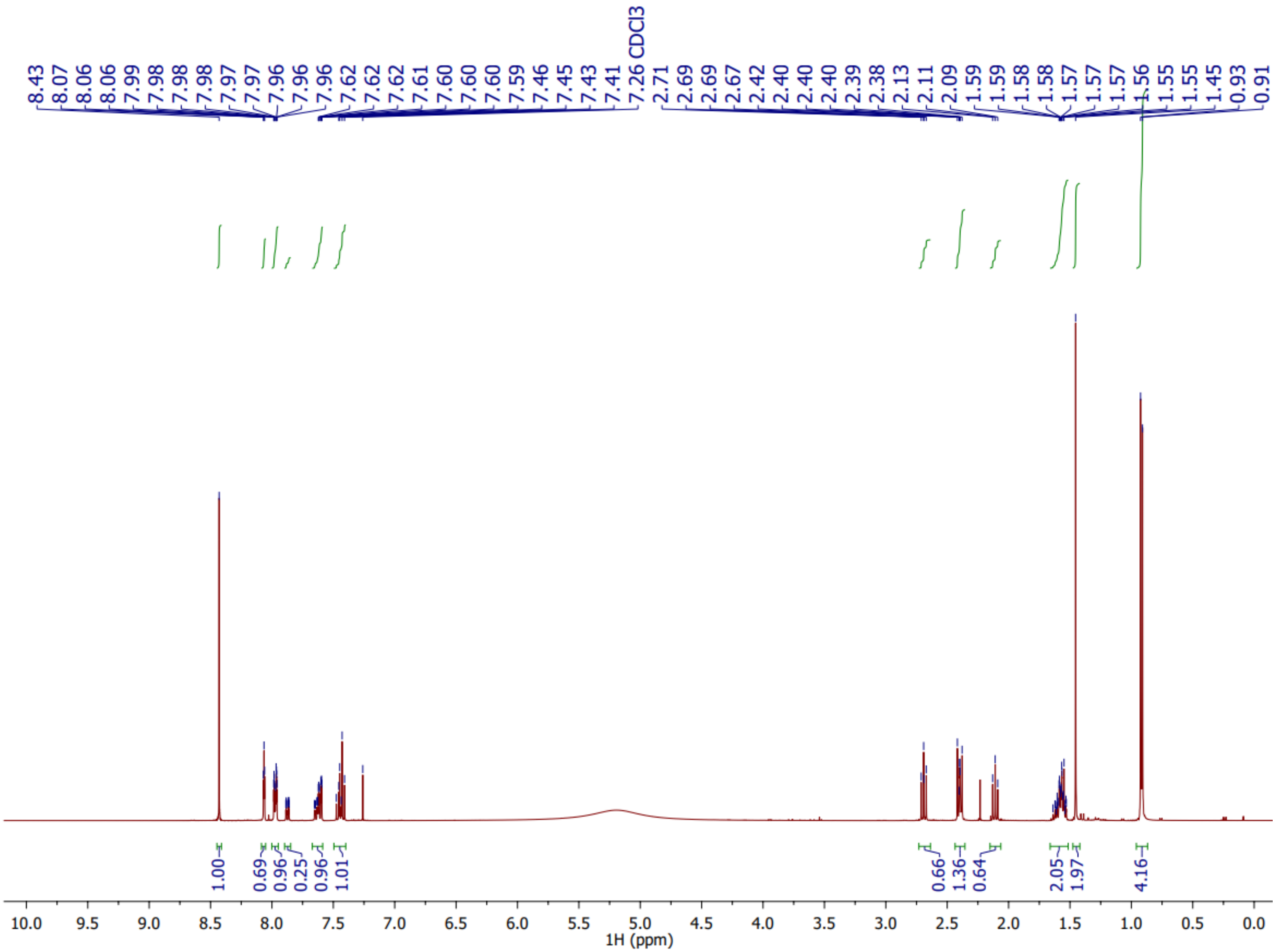

^{1}H NMR of the crude reaction mixture of oxidation of 4-methylpentanoic acid 60 with *m*CPBA 5.

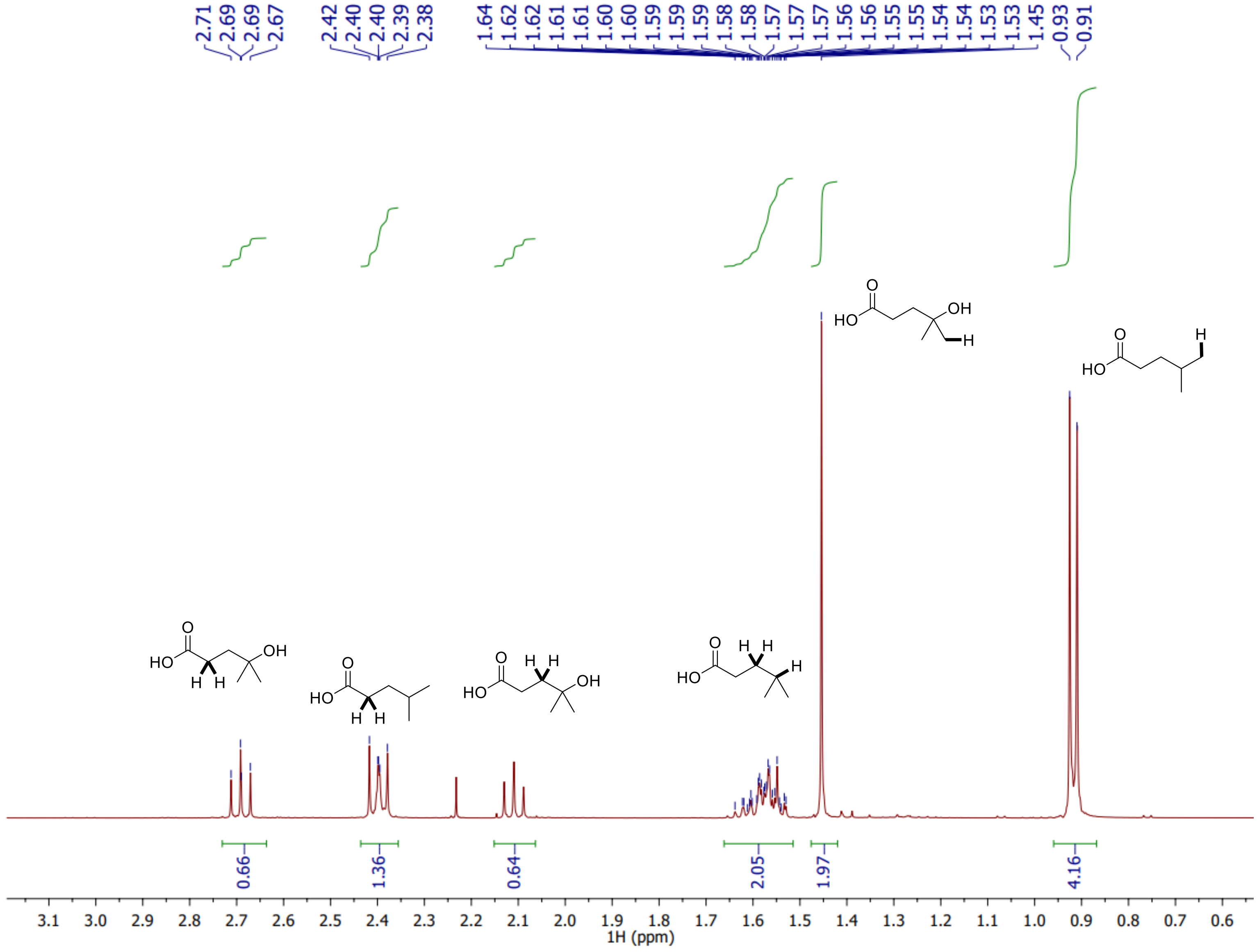

^{1}H NMR of the crude reaction mixture of oxidation of 5-methylhexanoic acid 61 with *m*CPBA 5.

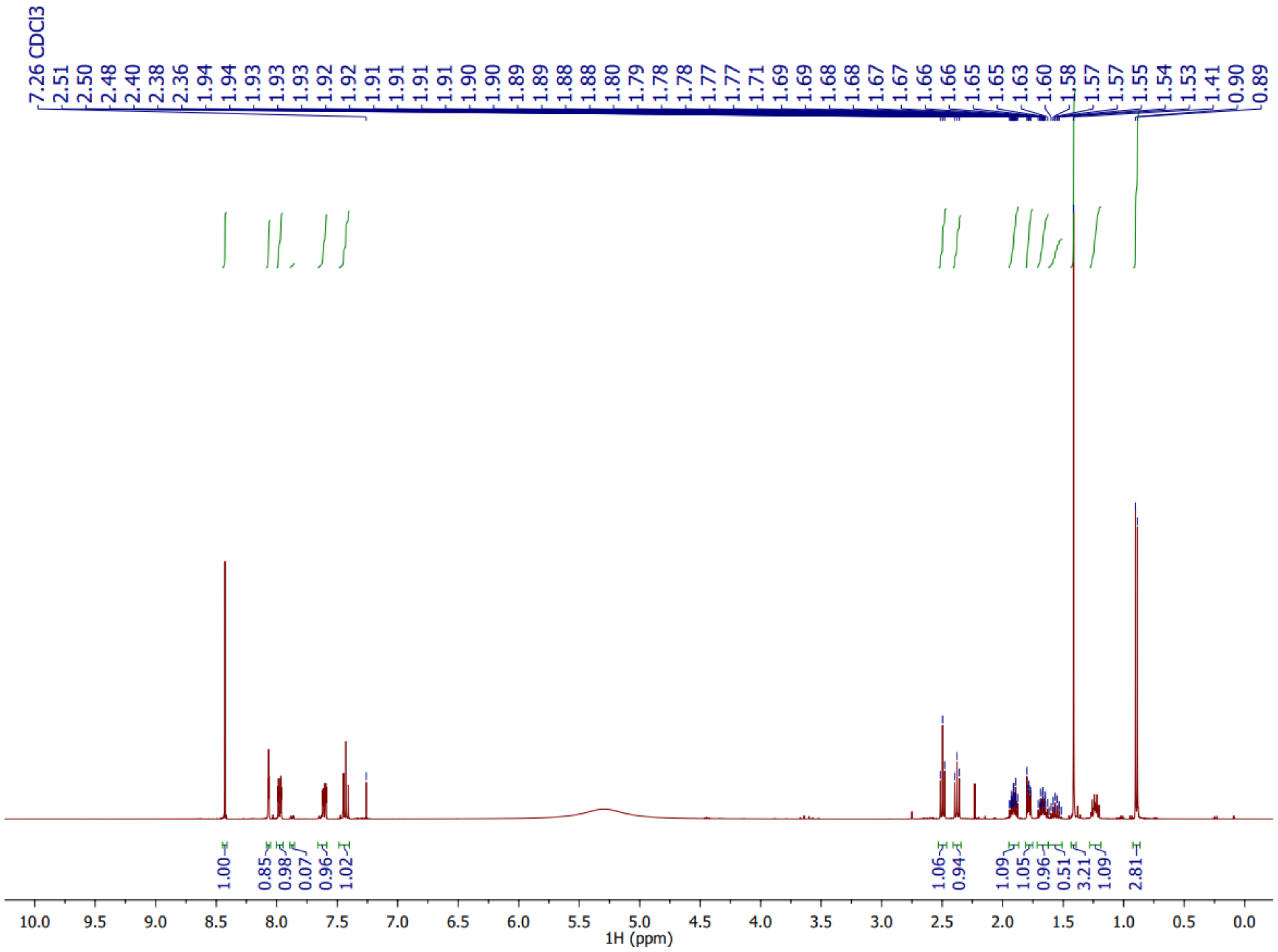

^{1}H NMR of the crude reaction mixture of oxidation of 5-methylhexanoic acid 61 with *m*CPBA 5.

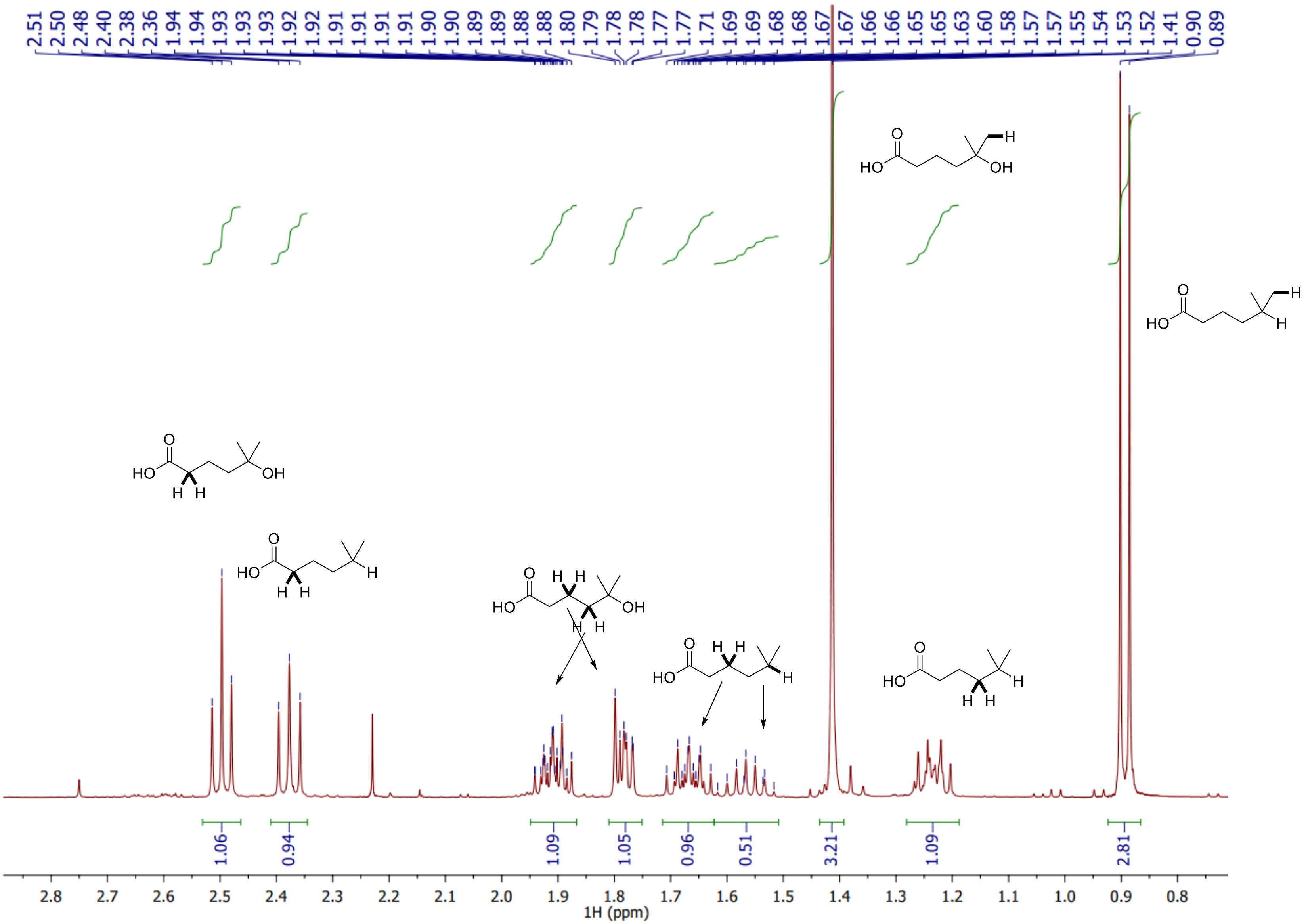

5 DOSY experiments.

5.1 Experimental for DOSY NMR experiments

In a 3 mL volumetric flask 0.15 mmol of analyte of interest and 0, 1, 2 and 3 eq of NFTB was dissolved in $CDCl_3$ with 0.5% tetramethylsilane solution to obtain final volume of 3mL. An aliquot of the obtained solution was analysed by method described further.

All of the NMR experiments were recorded with a Bruker Avance III HD NanoBay (9.4 T) equipped with a pulsed gradient unit capable of producing magnetic field pulse gradients in the z-direction of 56 G cm^{-1}. The probe temperature was set to 300 K. The gradient strength was calibrated using cyclohexanol. All DOSY experiments were performed using the bipolar pulse longitudinal eddy current delay (BPPLED) pulse sequence. The analysed samples were locked on $CDCl_3$ signal. The duration of the magnetic field pulse gradients (δ = 0.5 ms) and the diffusion times (Δ = 200 ms) were optimised for cyclohexanol and used throughout this investigation, the eddy current delay (T_e) was set to 5 ms. Optimisation of δ and Δ for *m*CPBA **5** did not yield different results. A series of 16 spectra on 16 K data points were collected for each of the experiments. The pulse gradients (g) were incremented from 5 to 95% of the maximum gradient strength in a linear ramp. The temperature was set and controlled at 300 K with an air flow of 400 L h^{-1}. After Fourier transformation and baseline correction, the diffusion dimension was processed with the Bruker xwinnmr software package (version 4.0.8).

5.1.1 DOSY cyclohexane/TMS/0 eq NFTB.

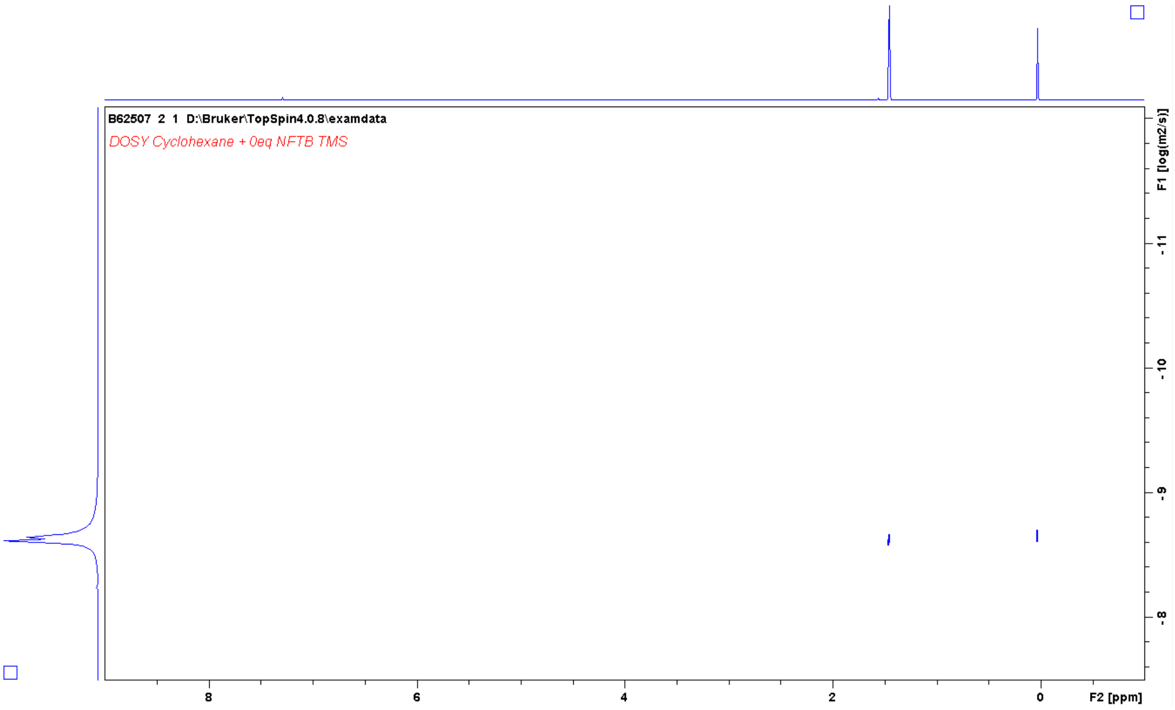

D cyclohexane

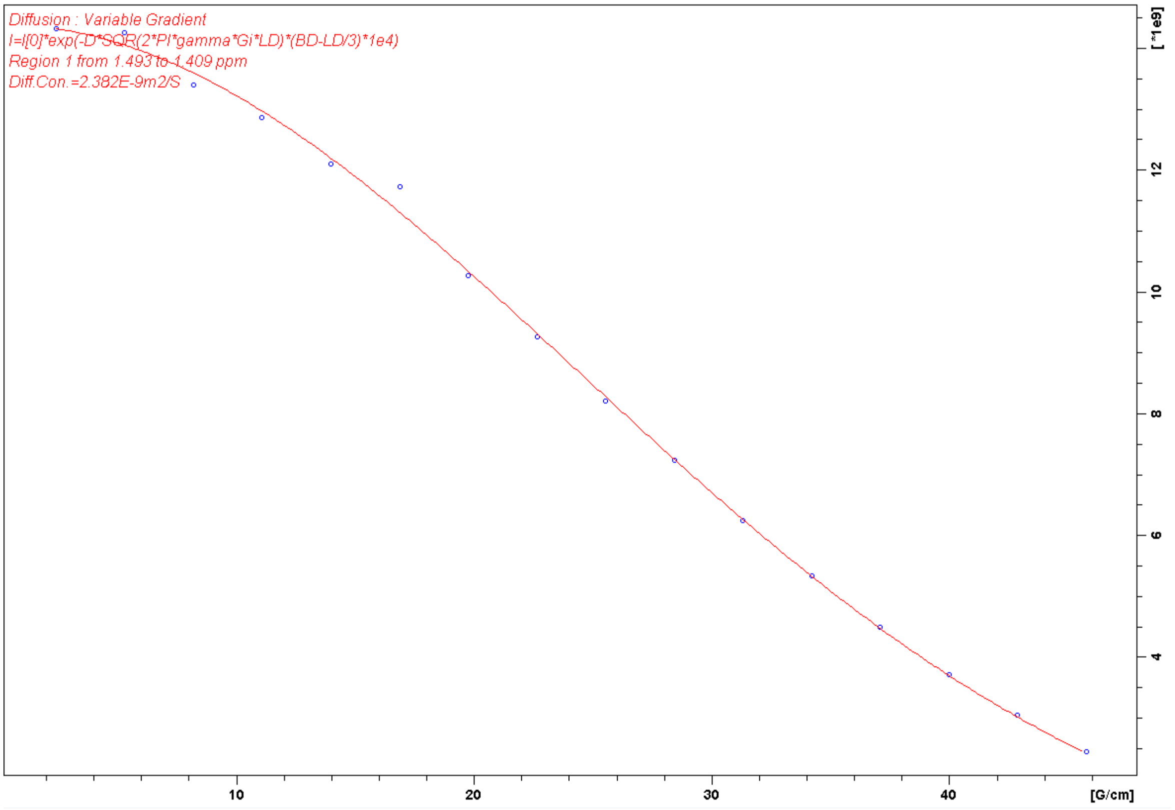

D TMS

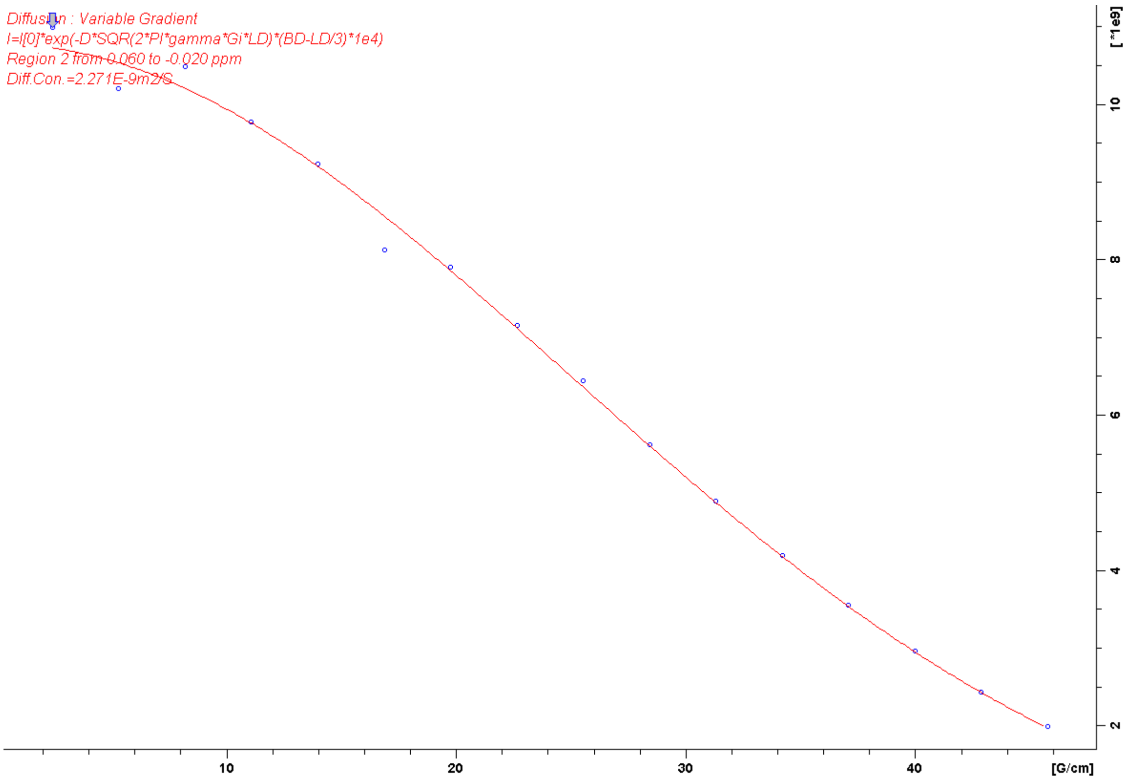

Diffusion : Variable Gradient
I=I[0]*exp(-D*SQR(2*PI*gamma*Gi*LD)*(BD-LD/3)*1e4)
Region 2 from 0.060 to -0.020 ppm
Diff.Con.=2.271E-9m2/S
[*1e9]
10
8
6
4
2
10
20
30
40
[G/cm]

5.1.2 DOSY cyclohexane/TMS/1 eq NFTB.

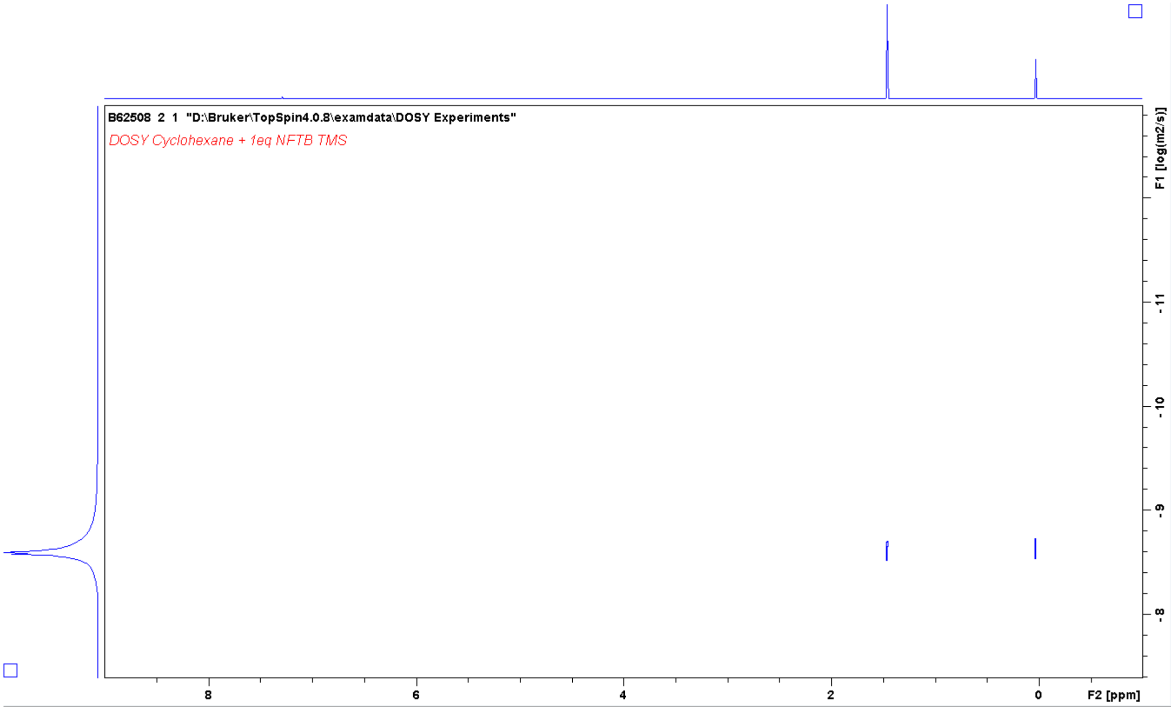

D cyclohexane

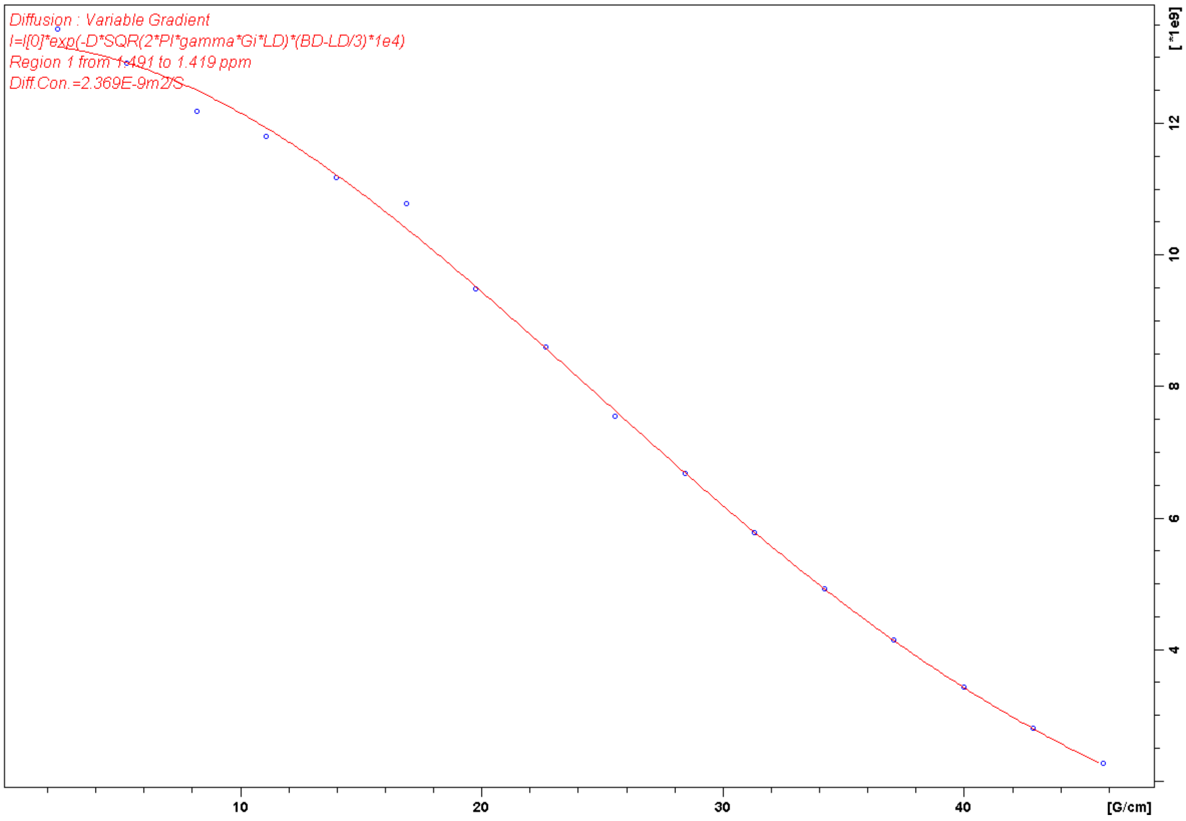

D TMS

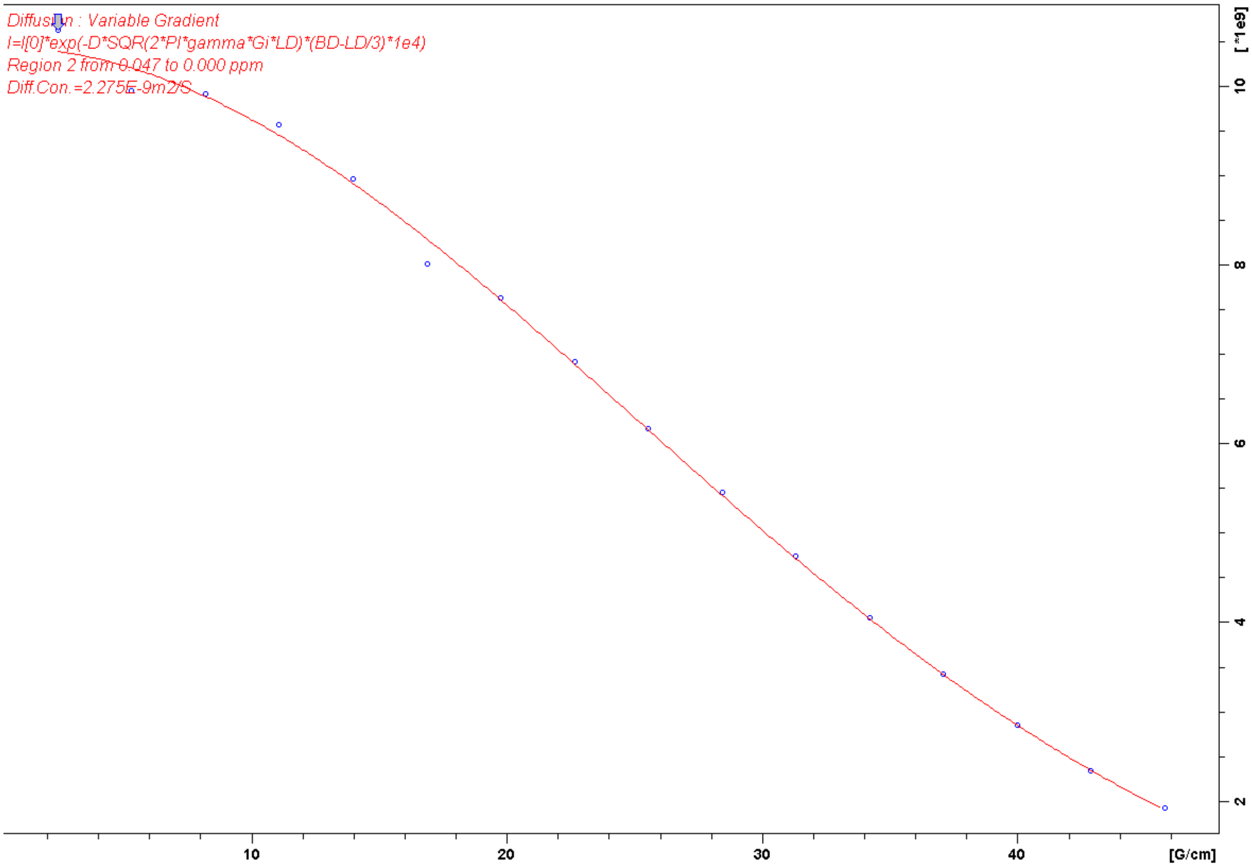
Diffusion : Variable Gradient
I=I[0]*exp(-D*SQR(2*PI*gamma*Gi*LD)*(BD-LD/3)*1e4)
Region 2 from 0.047 to 0.000 ppm
Diff.Con.=2.275E-9m2/S
[*1e9]
10
8
6
4
2
10
20
30
40
[G/cm]

5.1.3 DOSY cyclohexane/TMS/2 eq NFTB.

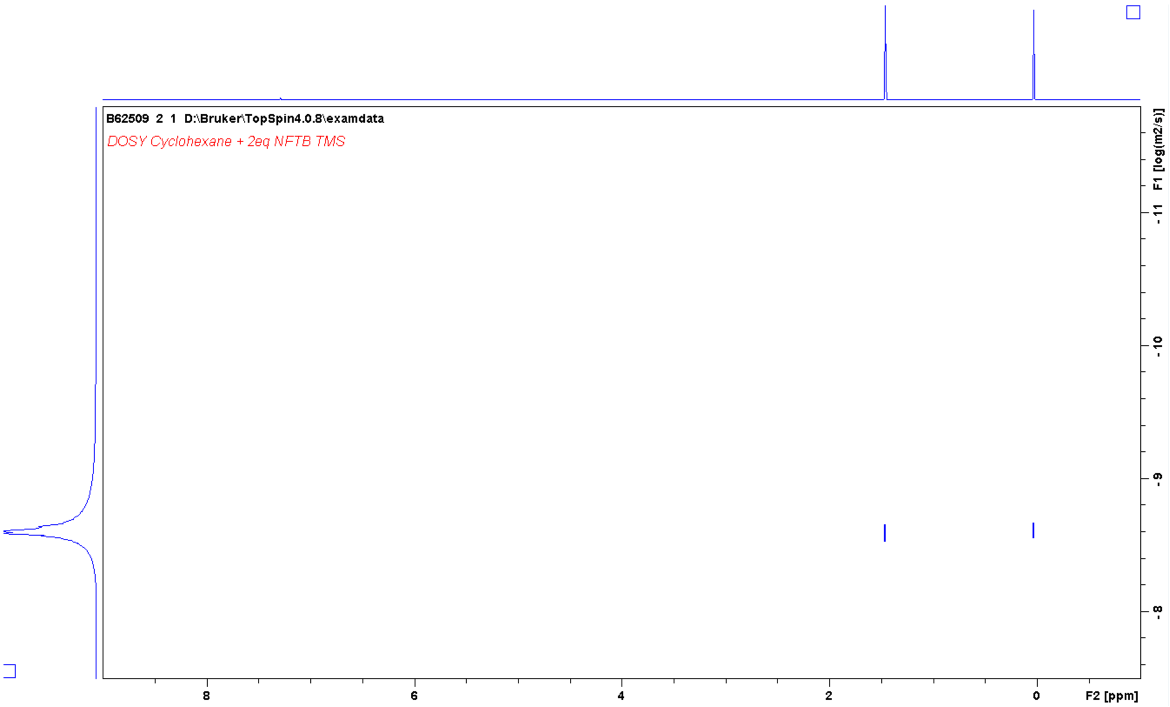

D cyclohexane

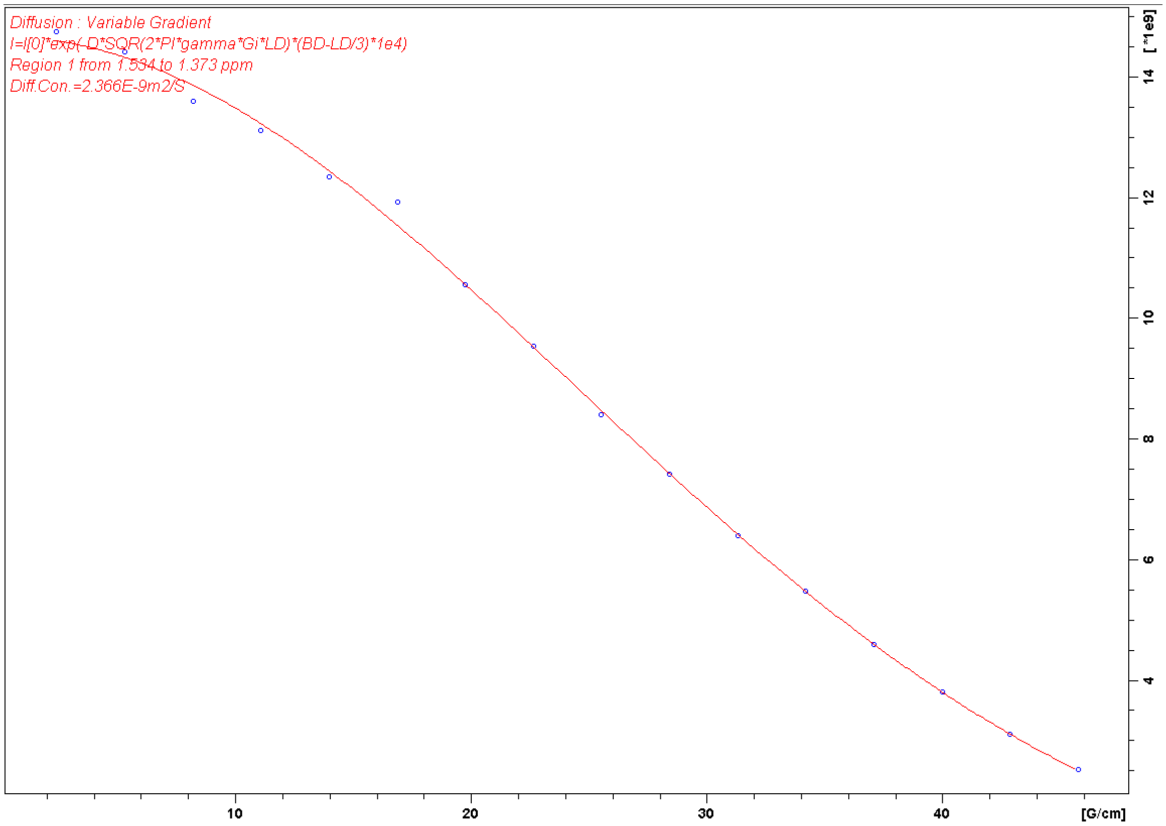

D TMS

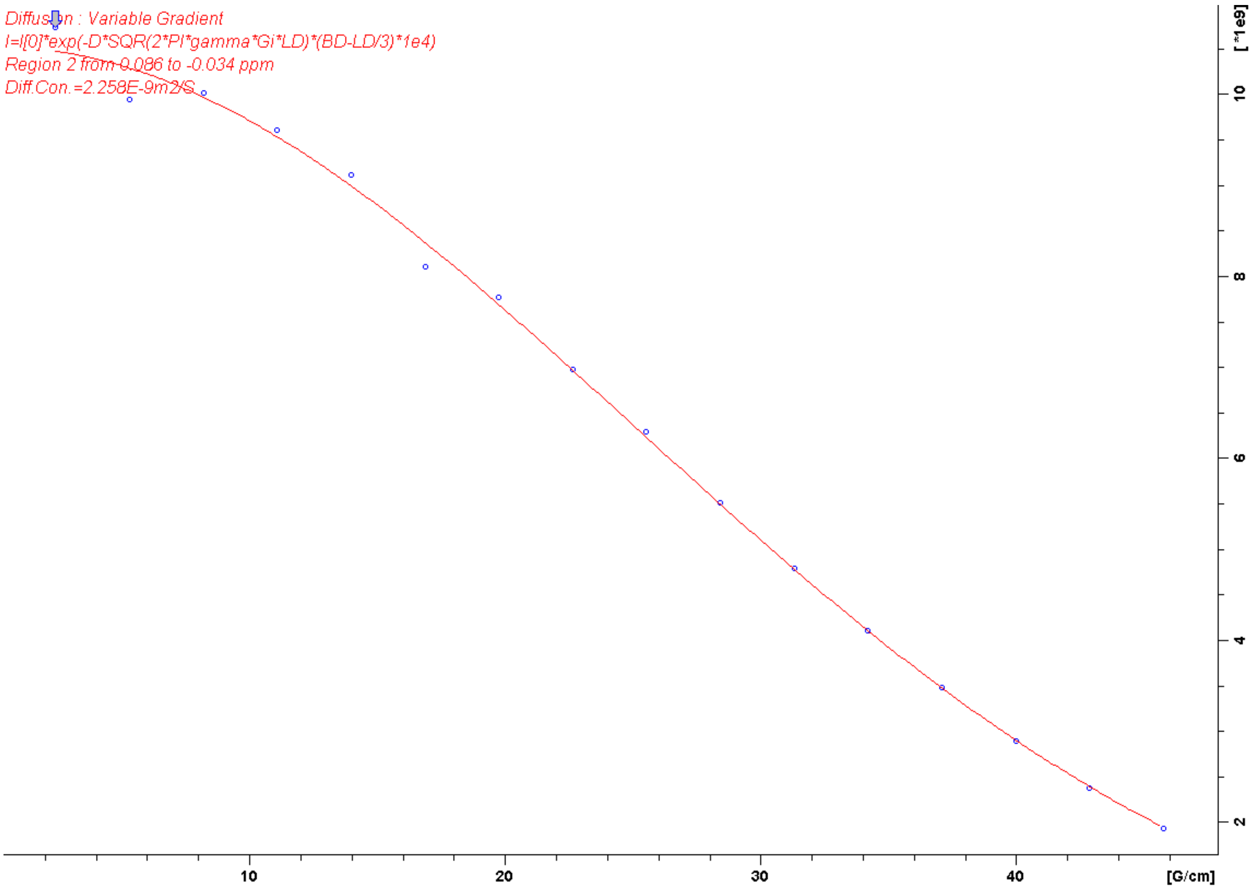

Diffusion : Variable Gradient
I=I[0]*exp(-D*SQR(2*PI*gamma*Gi*LD)*(BD-LD/3)*1e4)
Region 2 from 0.086 to -0.034 ppm
Diff.Con.=2.258E-9m2/S
[*1e9]
10
8
6
4
2
10
20
30
40
[G/cm]

5.1.4 DOSY cyclohexane/TMS/3 eq NFTB.

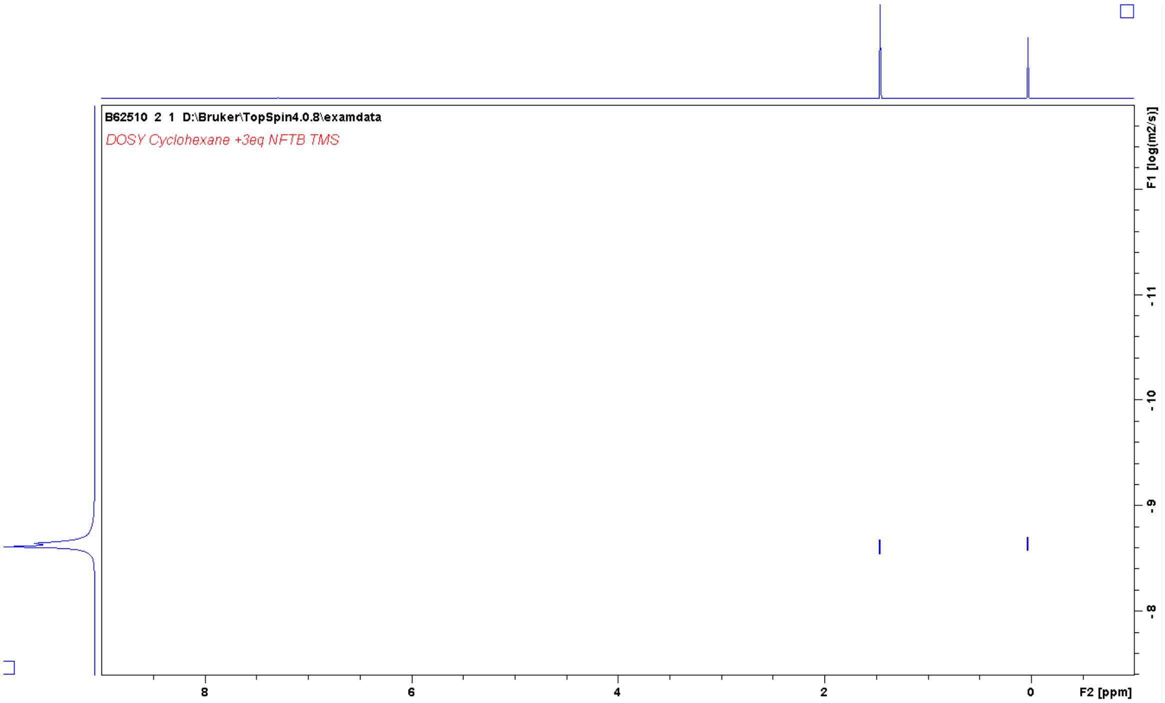

D cyclohexane

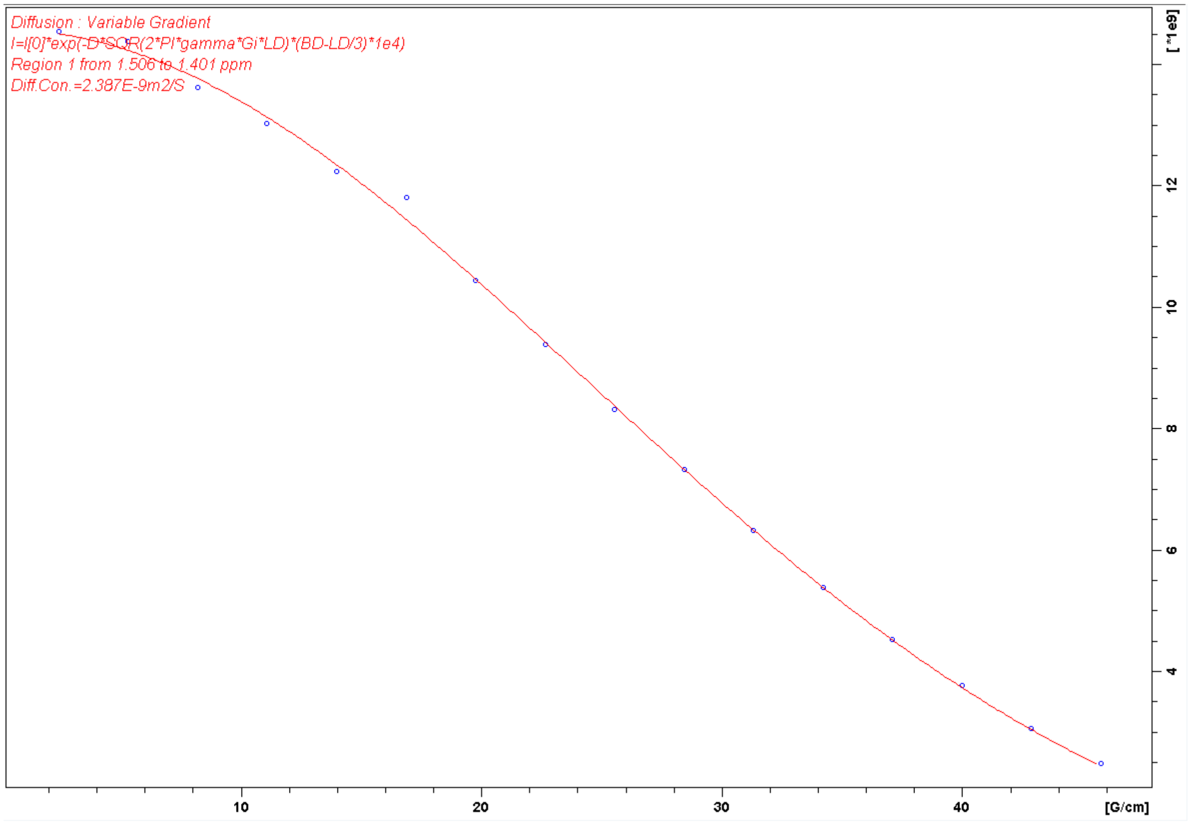

D TMS

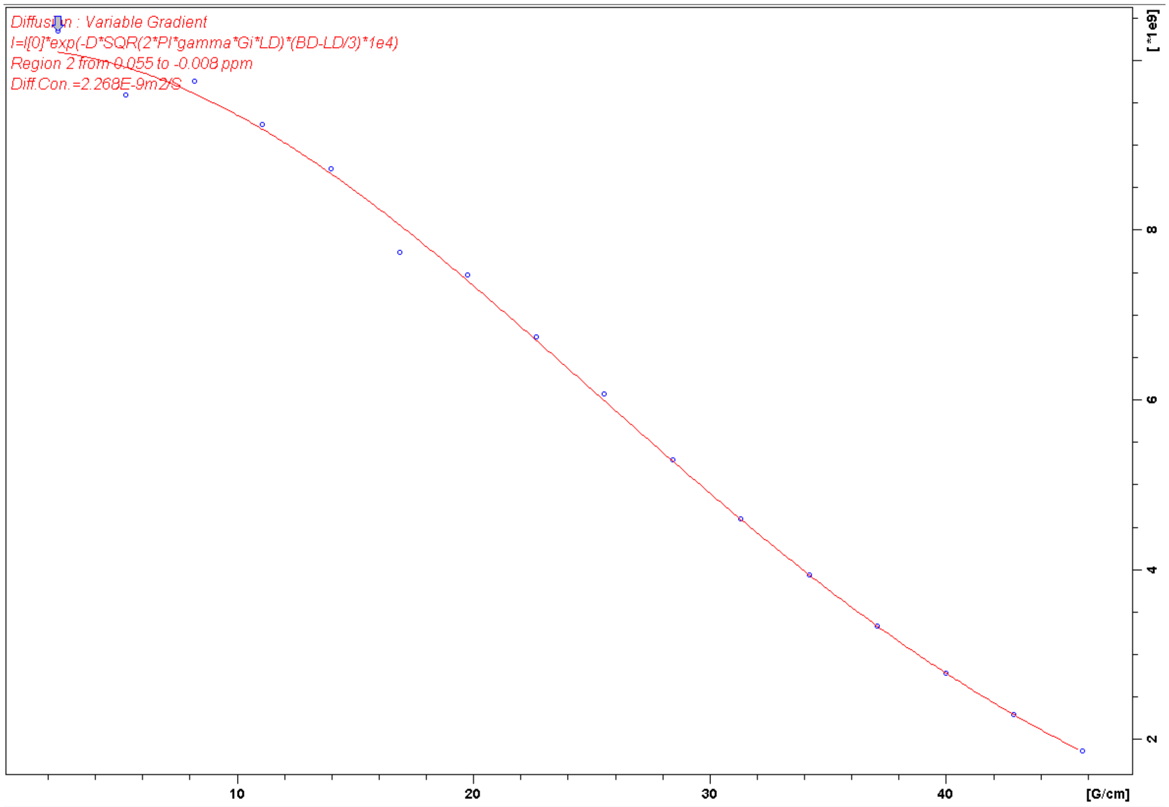
Diffusion : Variable Gradient
I=I[0]*exp(-D*SQR(2*PI*gamma*Gi*LD)*(BD-LD/3)*1e4)
Region 2 from 0.055 to -0.008 ppm
Diff.Con.=2.268E-9m2/S
[*1e9]
8
6
4
2
10
20
30
40
[G/cm]

5.1.5 DOSY mCPBA/TMS/0 eq NFTB

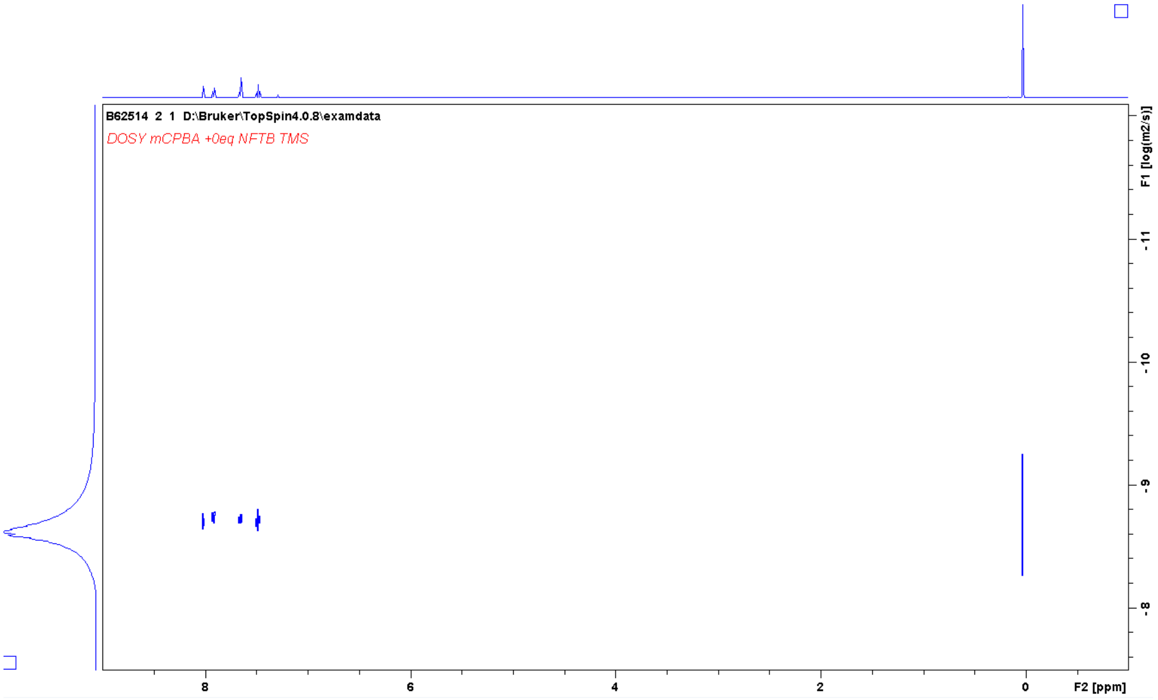

D *m*CPBA

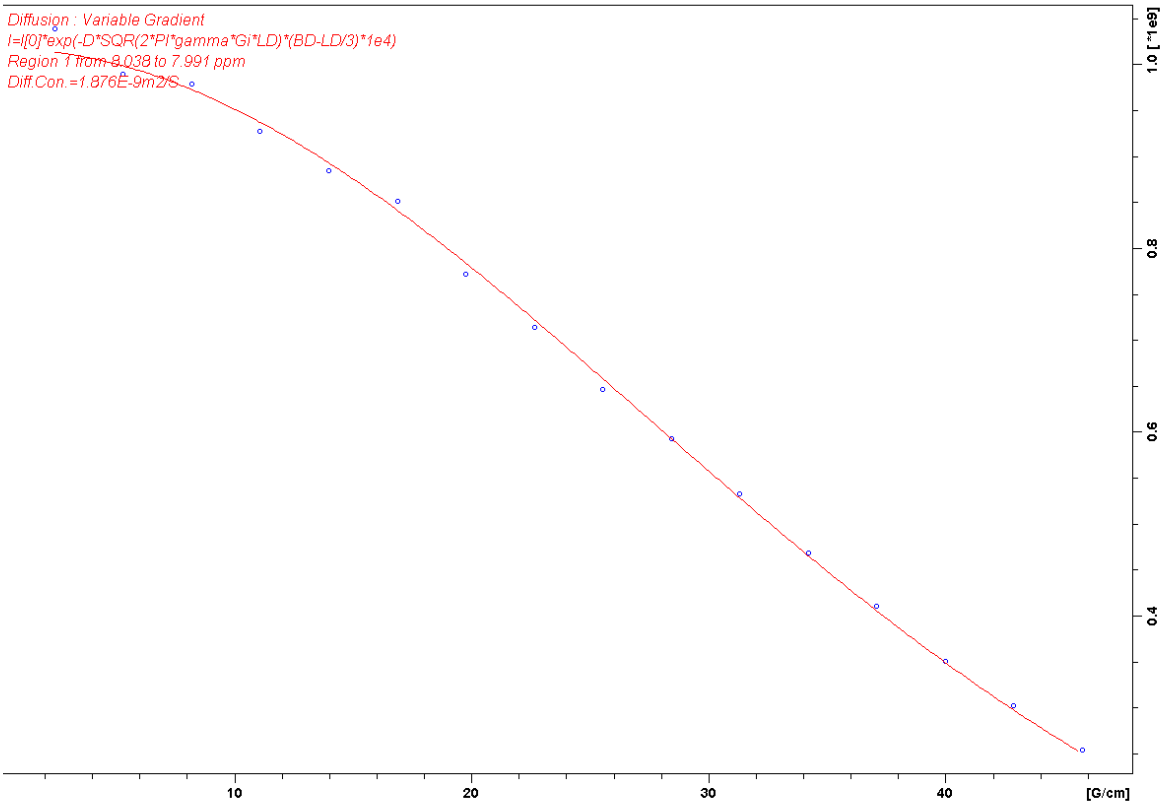

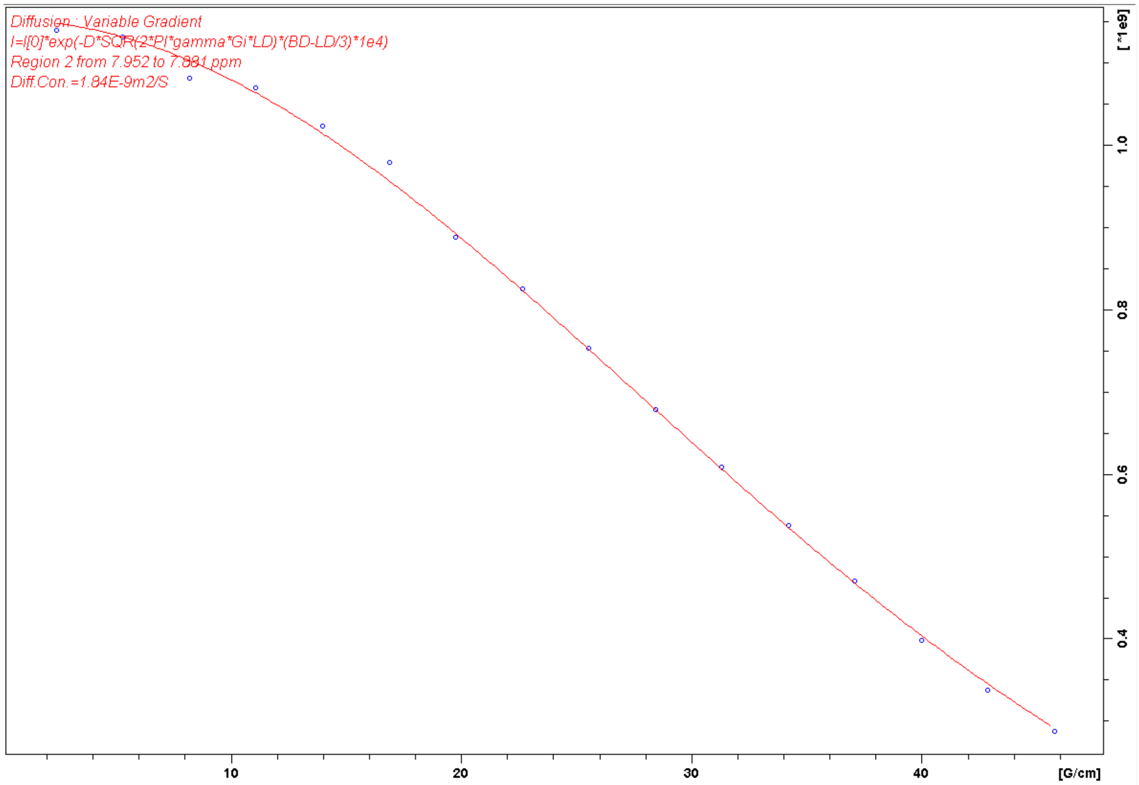
Diffusion : Variable Gradient
I=I[0]*exp(-D*SQR(2*PI*gamma*Gi*LD)*(BD-LD/3)*1e4)
Region 2 from 7.952 to 7.881 ppm
Diff.Con.=1.84E-9m2/S
[*1e9]
1.0
0.8
0.6
0.4
10
20
30
40
[G/cm]

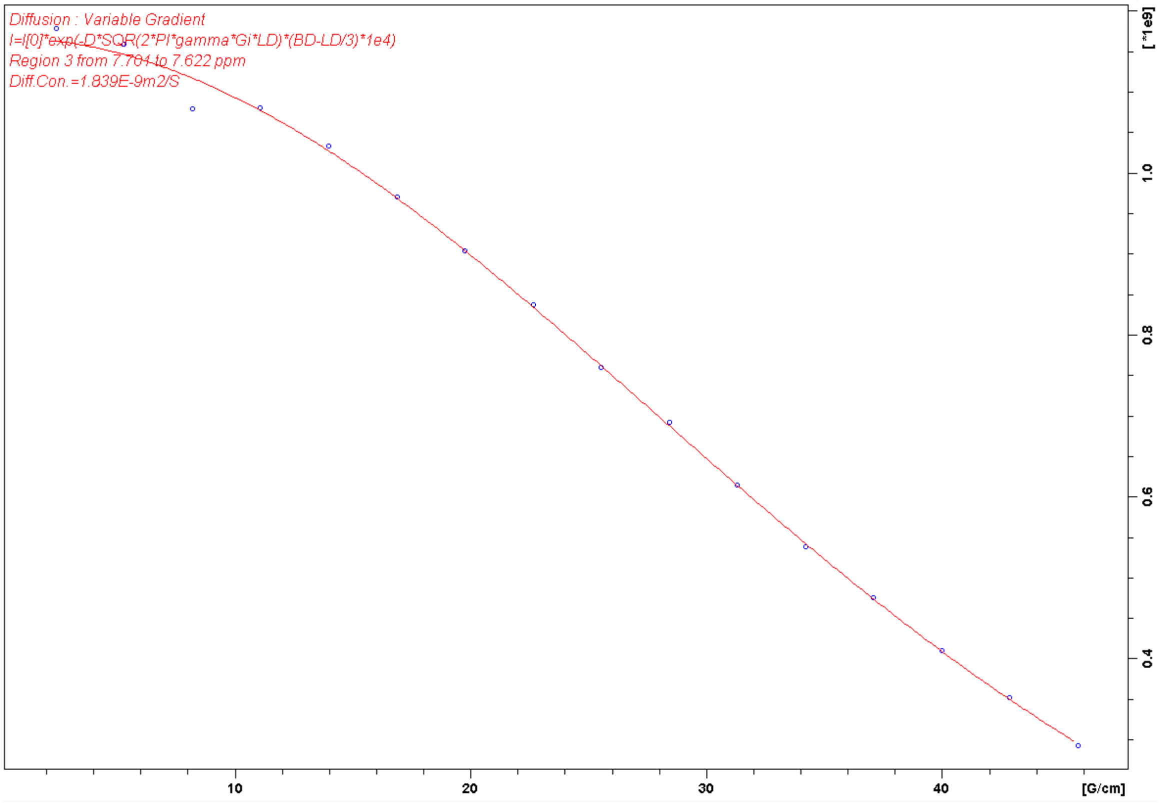
Diffusion : Variable Gradient
I=I[0]*exp(-D*SQR(2*PI*gamma*Gi*LD)*(BD-LD/3)*1e4)
Region 3 from 7.701 to 7.622 ppm
Diff.Con.=1.839E-9m2/S
[*1e9]
1.0
0.8
0.6
0.4
10
20
30
40
[G/cm]

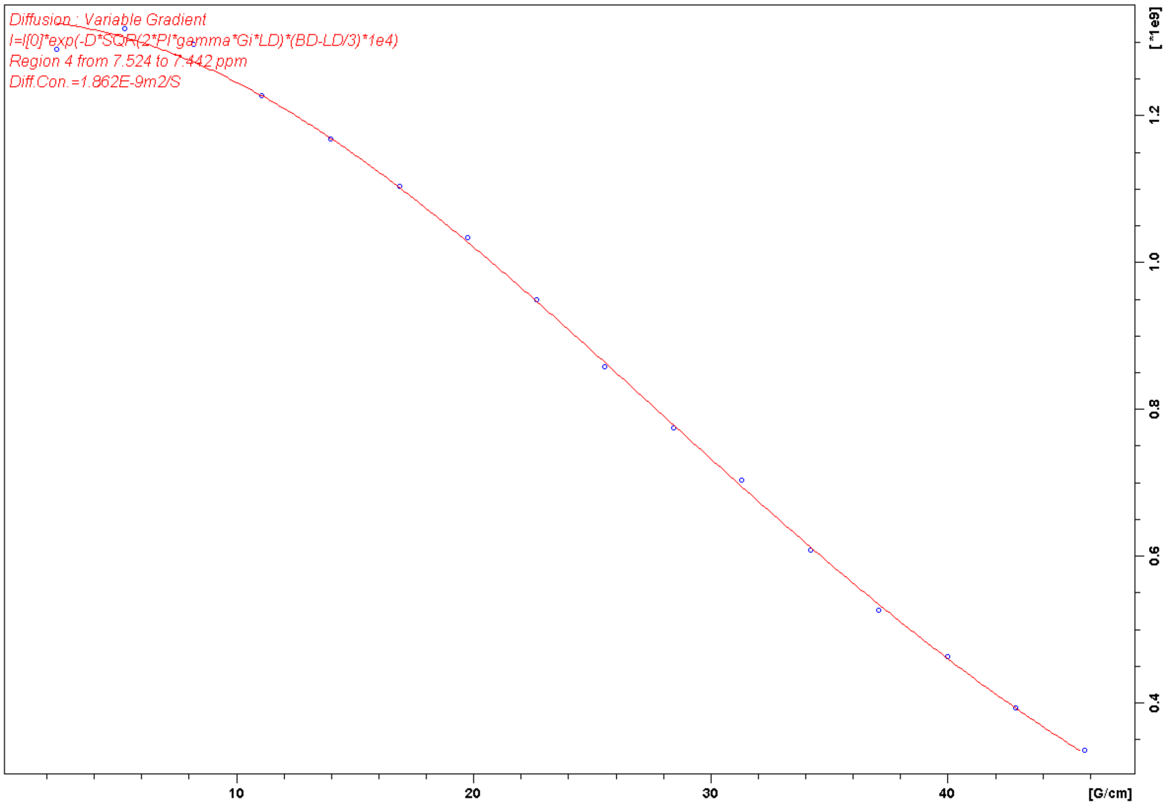
Diffusion : Variable Gradient
I=I[0]*exp(-D*SQR(2*PI*gamma*Gi*LD)*(BD-LD/3)*1e4)
Region 4 from 7.524 to 7.442 ppm
Diff.Con.=1.862E-9m2/S
[*1e9]
1.2
1.0
0.8
0.6
0.4
10
20
30
40
[G/cm]

D TMS

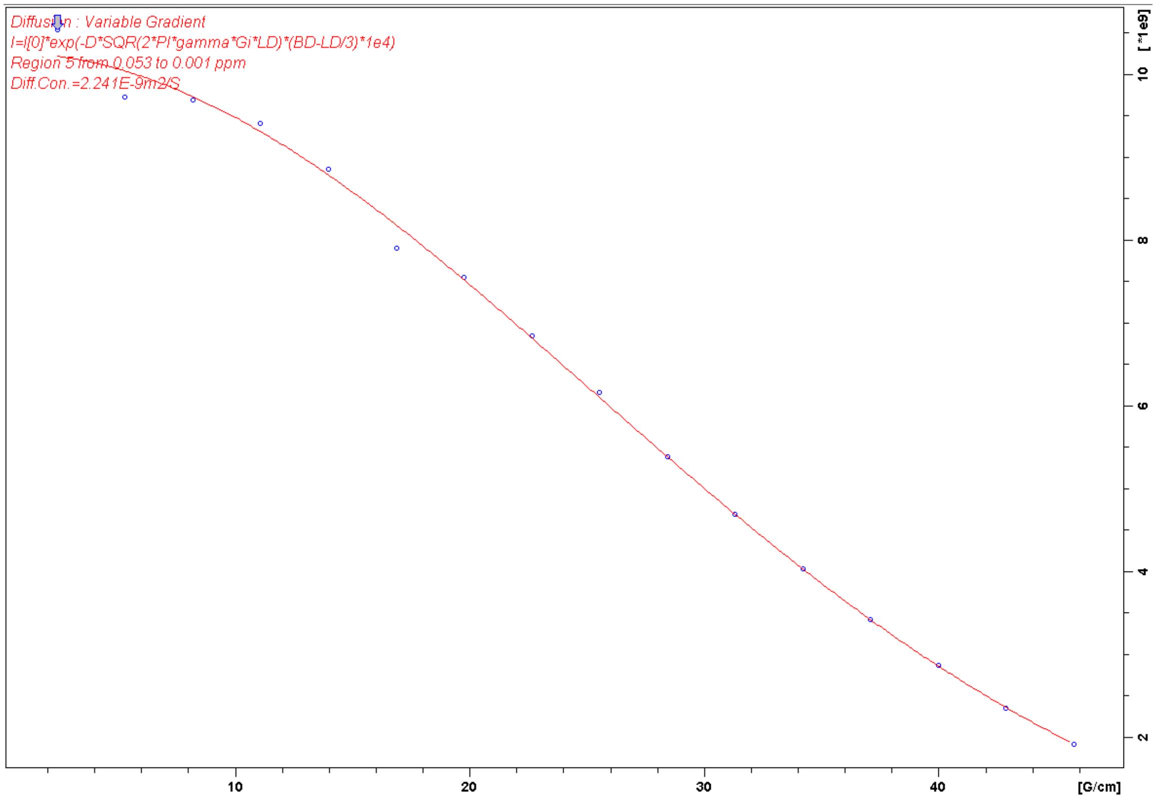
Diffusion : Variable Gradient
I=I[0]*exp(-D*SQR(2*PI*gamma*Gi*LD)*(BD-LD/3)*1e4)
Region 5 from 0.053 to 0.001 ppm
Diff.Con.=2.241E-9m2/S
[*1e9]
10
8
6
4
2
10
20
30
40
[G/cm]

5.1.6 DOSY mCPBA/TMS/1 eq NFTB

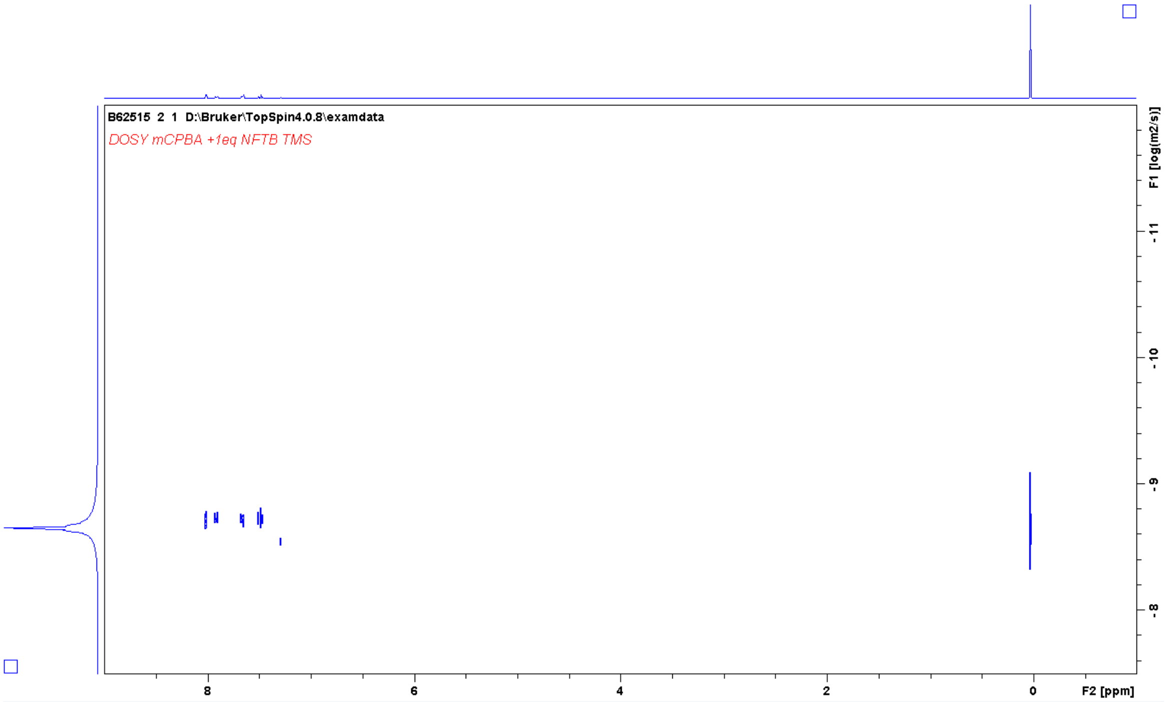

D *m*CPBA

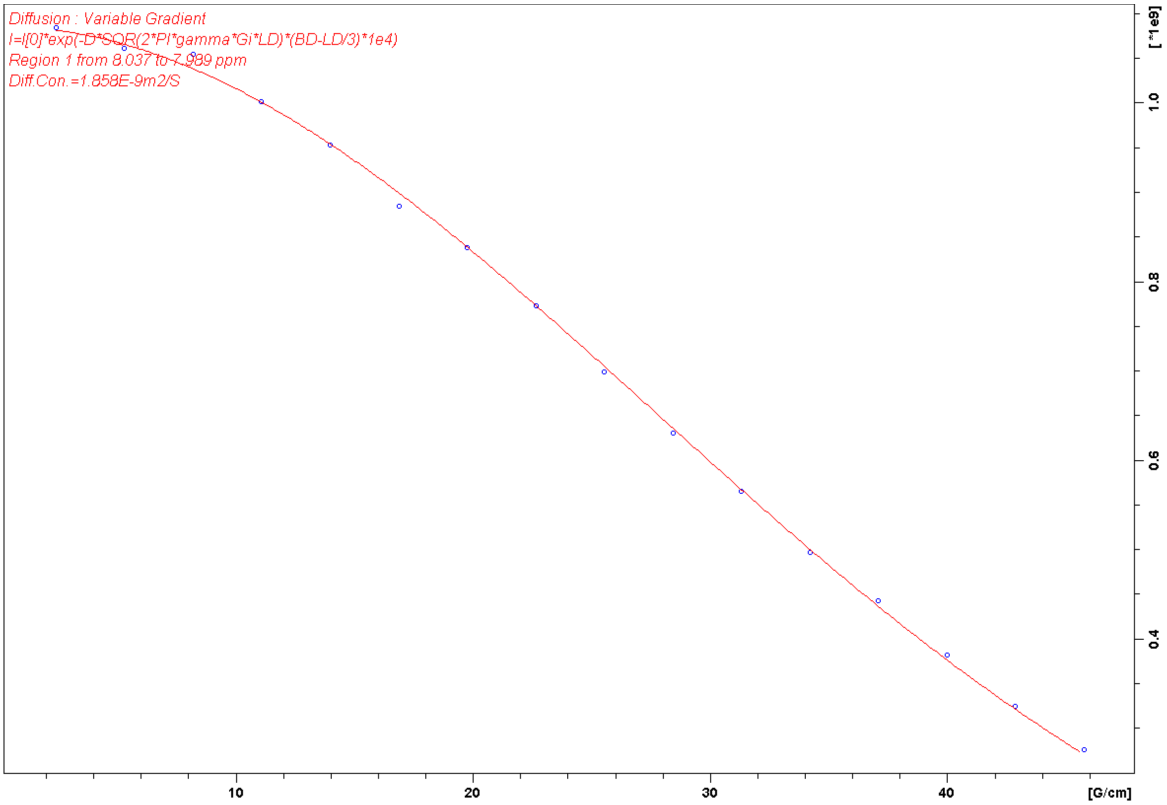

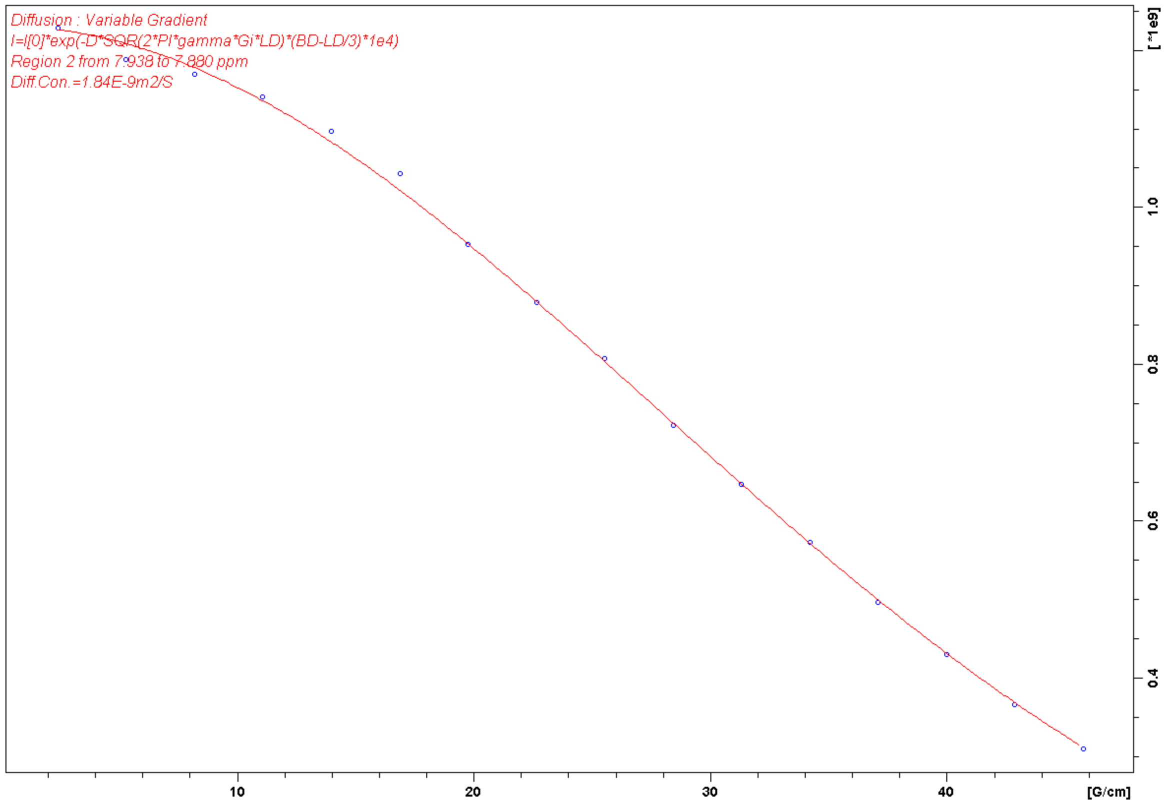
Diffusion : Variable Gradient
I=I[0]*exp(-D*SQR(2*PI*gamma*Gi*LD)*(BD-LD/3)*1e4)
Region 2 from 7.938 to 7.880 ppm
Diff.Con.=1.84E-9m2/S
[*1e9]
1.0
0.8
0.6
0.4
10
20
30
40
[G/cm]

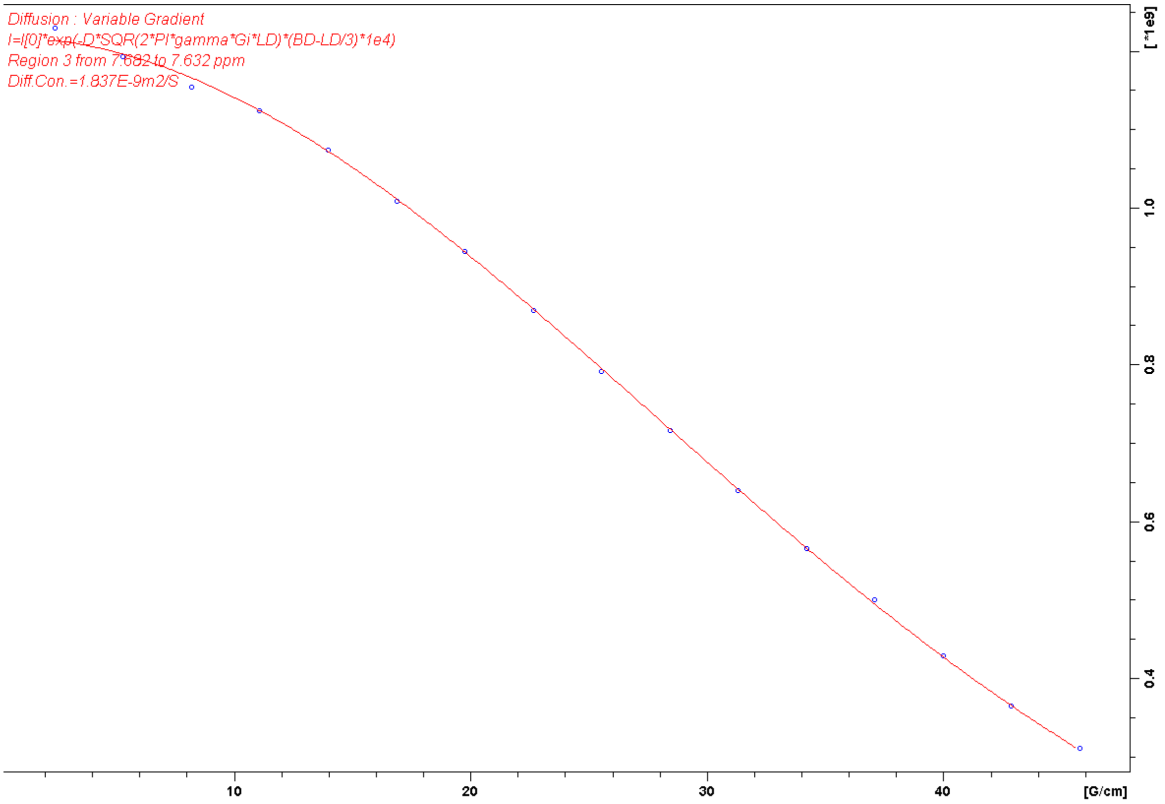
Diffusion : Variable Gradient
I=I[0]*exp(-D*SQR(2*PI*gamma*Gi*LD)*(BD-LD/3)*1e4)
Region 3 from 7.682 to 7.632 ppm
Diff.Con.=1.837E-9m2/S
[*1e9]
1.0
0.8
0.6
0.4
10
20
30
40
[G/cm]

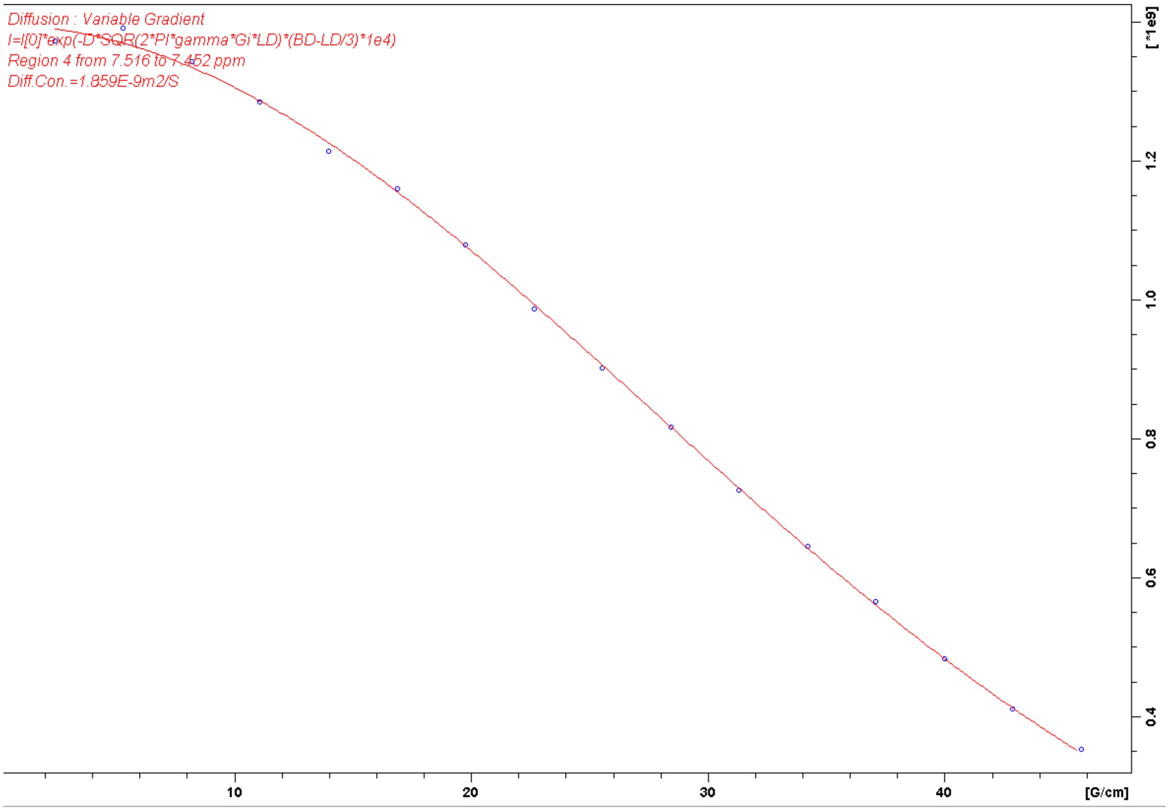
Diffusion : Variable Gradient
I=I[0]*exp(-D*SQR(2*PI*gamma*Gi*LD)*(BD-LD/3)*1e4)
Region 4 from 7.516 to 7.452 ppm
Diff.Con.=1.859E-9m2/S
[*1e9]
[G/cm]

D TMS

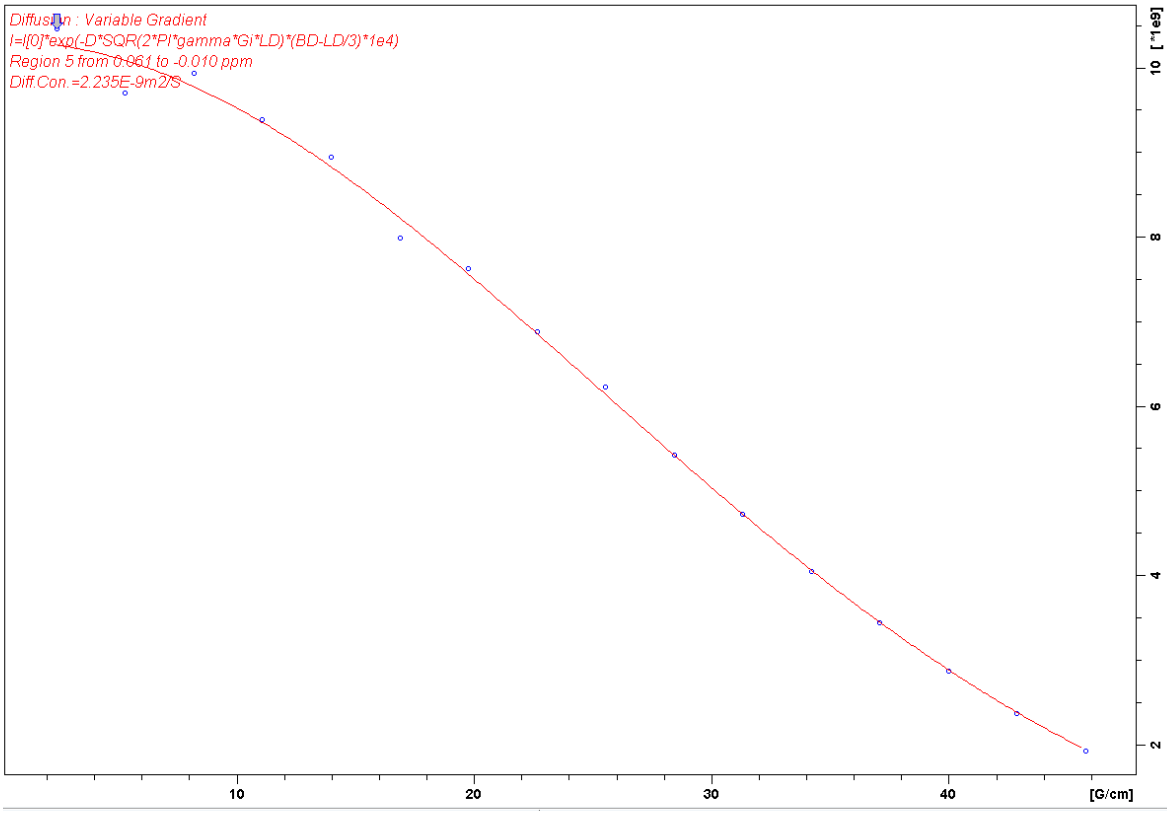
Diffusion : Variable Gradient
I=I[0]*exp(-D*SQR(2*PI*gamma*Gi*LD)*(BD-LD/3)*1e4)
Region 5 from 0.061 to -0.010 ppm
Diff.Con.=2.235E-9m2/S
[*1e9]
[G/cm]

5.1.7 DOSY mCPBA/TMS/2 eq NFTB

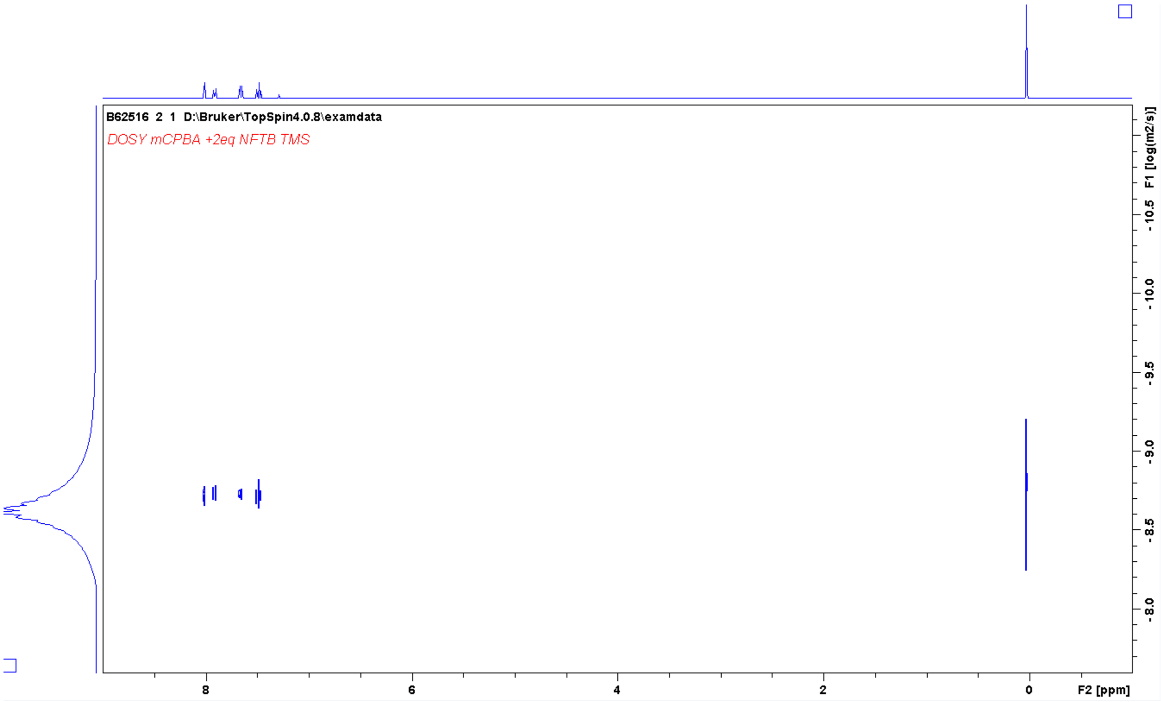

D *m*CPBA

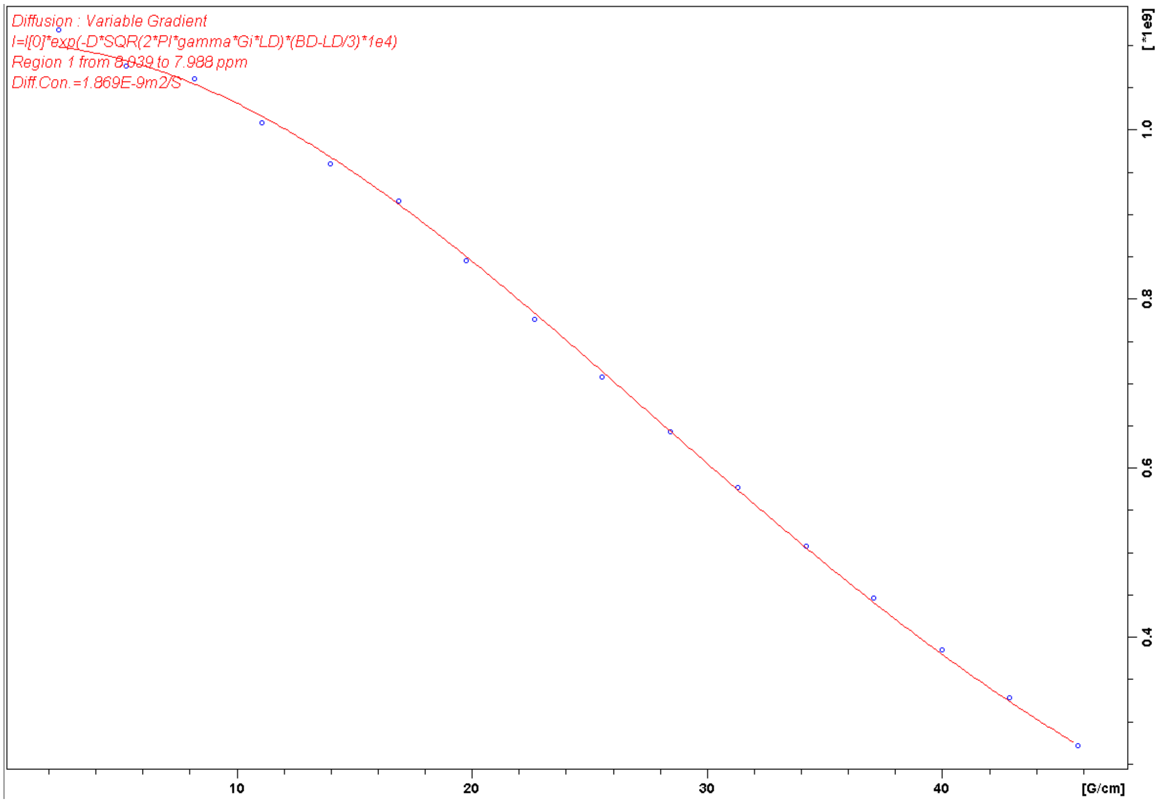

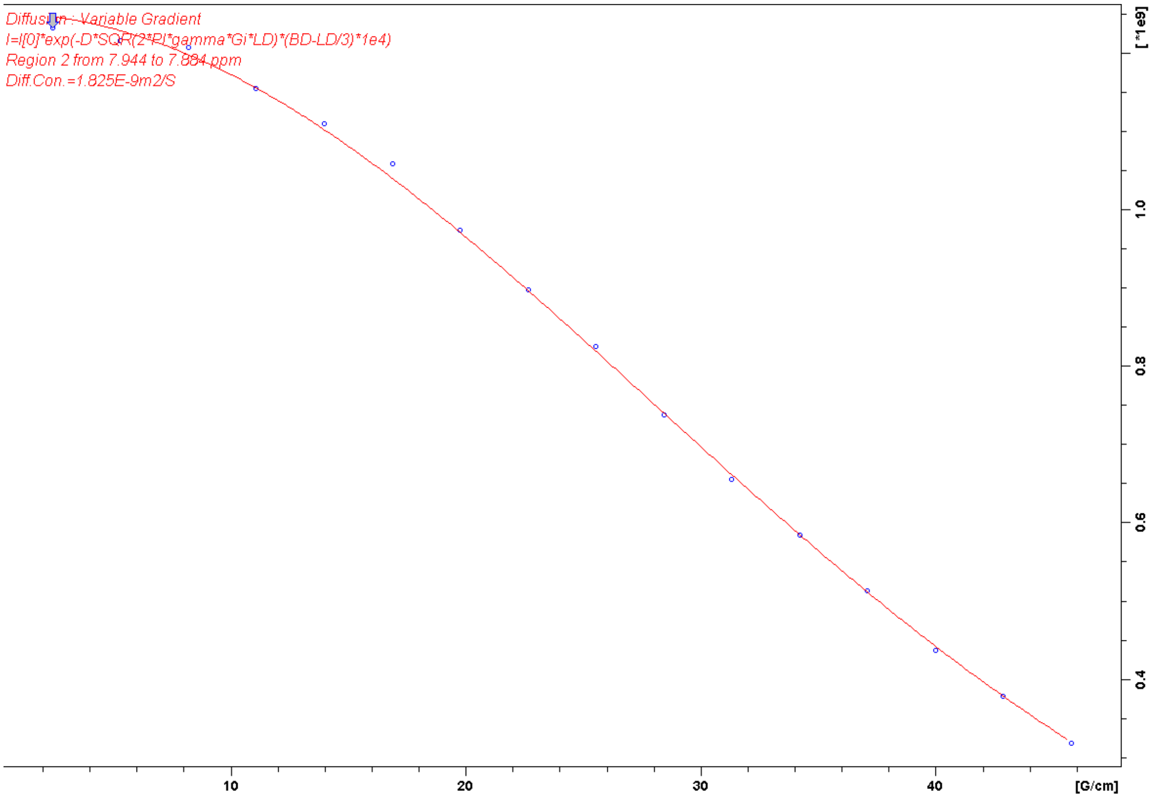
Diffusion : Variable Gradient
I=I[0]*exp(-D*SQR(2*PI*gamma*Gi*LD)*(BD-LD/3)*1e4)
Region 2 from 7.944 to 7.884 ppm
Diff.Con.=1.825E-9m2/S
[*1e9]
1.0
0.8
0.6
0.4
10
20
30
40
[G/cm]

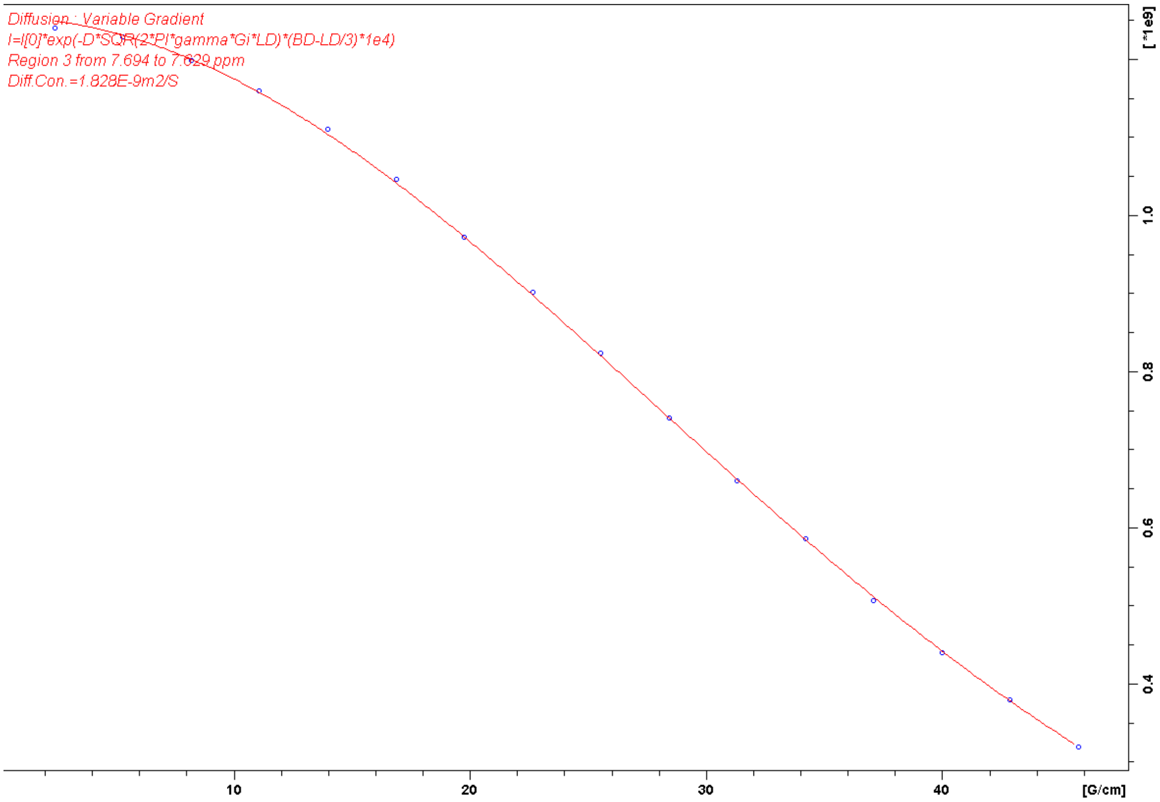
Diffusion : Variable Gradient
I=I[0]*exp(-D*SQR(2*PI*gamma*Gi*LD)*(BD-LD/3)*1e4)
Region 3 from 7.694 to 7.629 ppm
Diff.Con.=1.828E-9m2/S
[*1e9]
1.0
0.8
0.6
0.4
10
20
30
40
[G/cm]

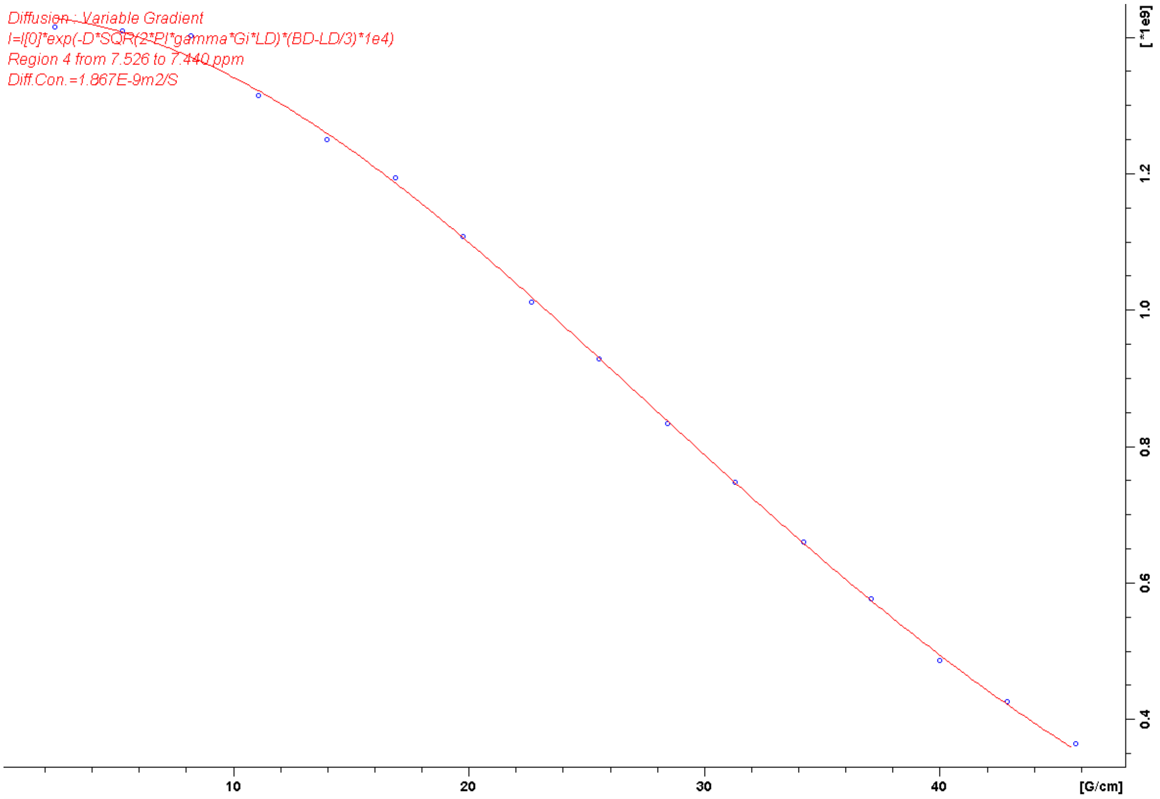
Diffusion : Variable Gradient
I=I[0]*exp(-D*SQR(2*PI*gamma*Gi*LD)*(BD-LD/3)*1e4)
Region 4 from 7.526 to 7.440 ppm
Diff.Con.=1.867E-9m2/S
[*1e9]
1.2
1.0
0.8
0.6
0.4
10
20
30
40
[G/cm]

D TMS

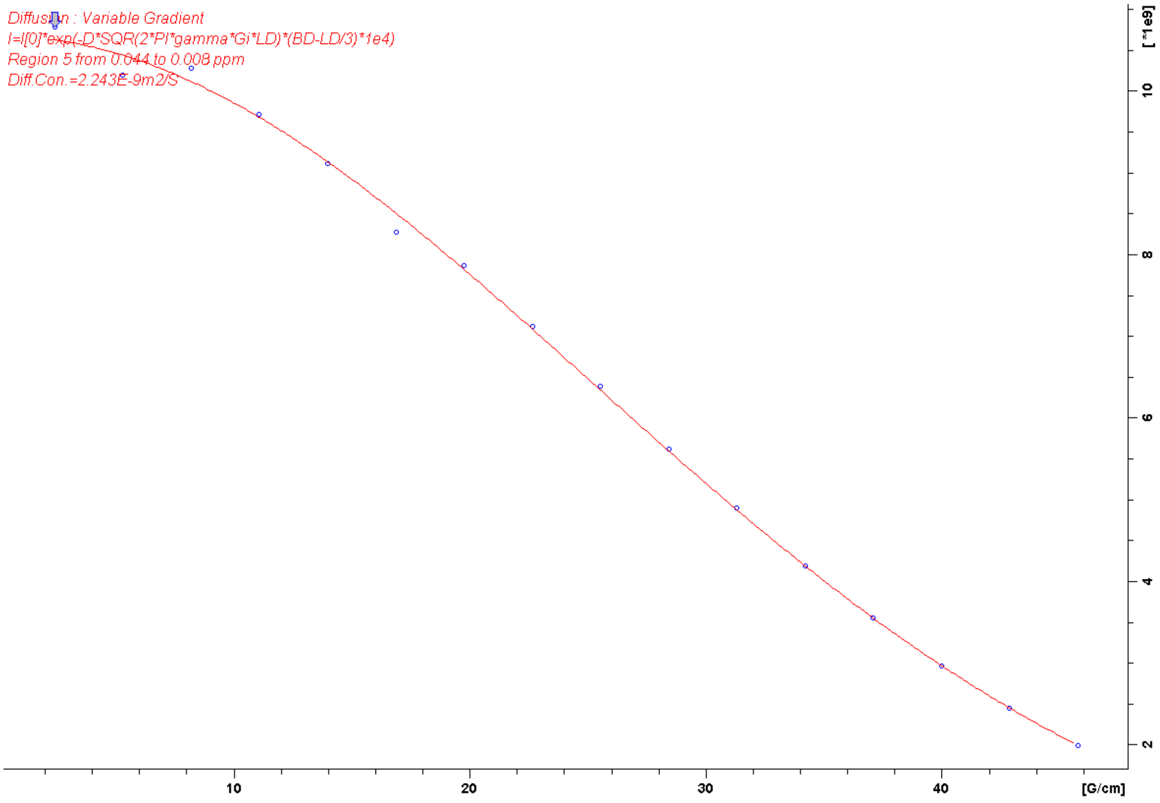
Diffusion : Variable Gradient
I=I[0]*exp(-D*SQR(2*PI*gamma*Gi*LD)*(BD-LD/3)*1e4)
Region 5 from 0.044 to 0.008 ppm
Diff.Con.=2.243E-9m2/S
[*1e9]
10
8
6
4
2
10
20
30
40
[G/cm]

5.1.8 DOSY mCPBA/TMS/3 eq NFTB

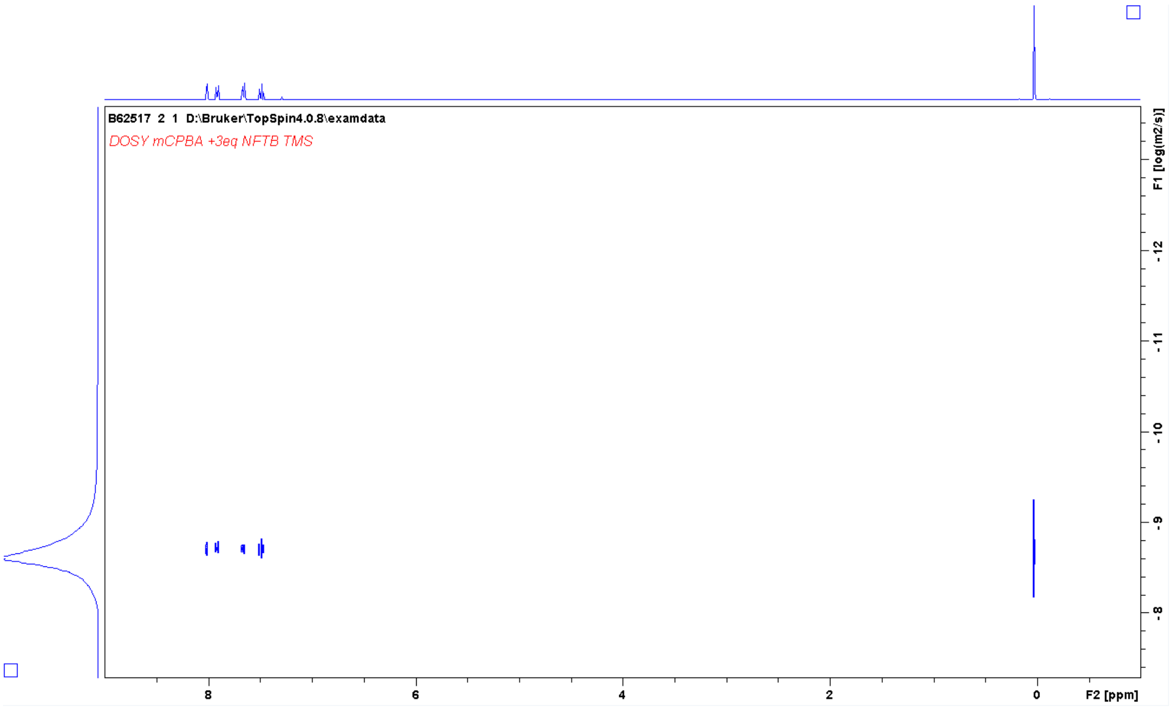

D *m*CPBA

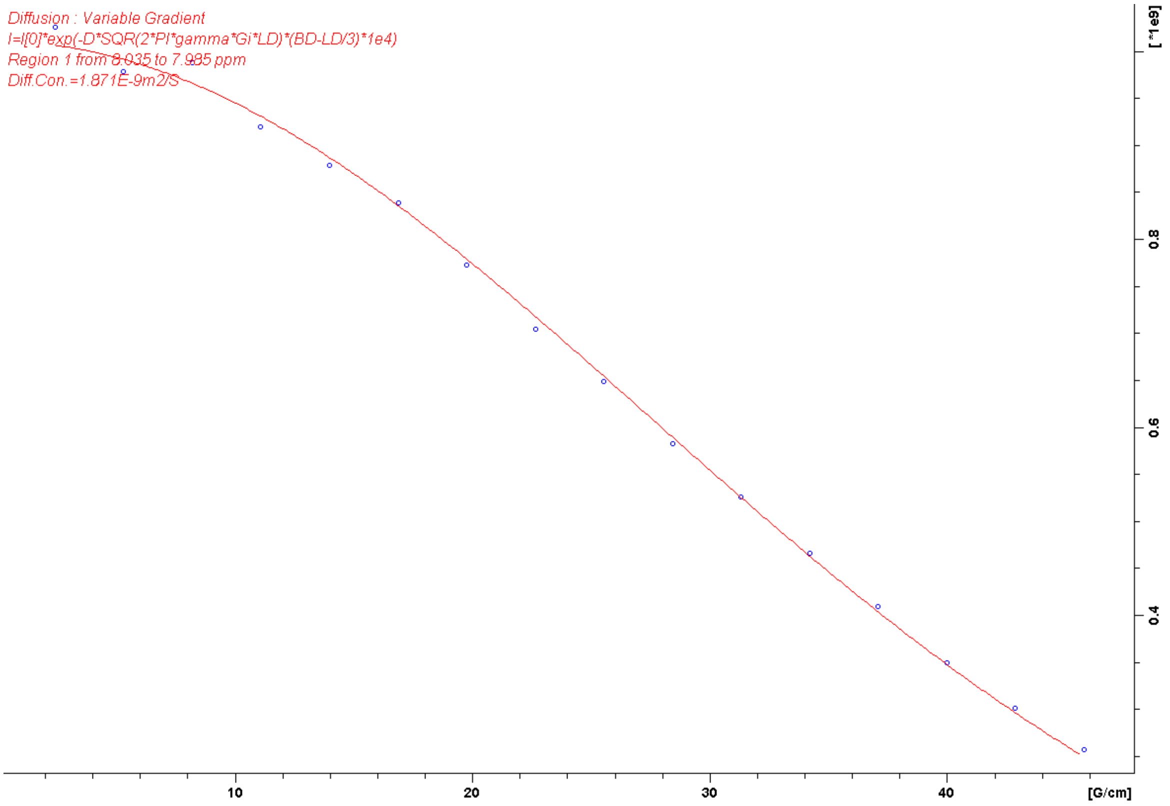

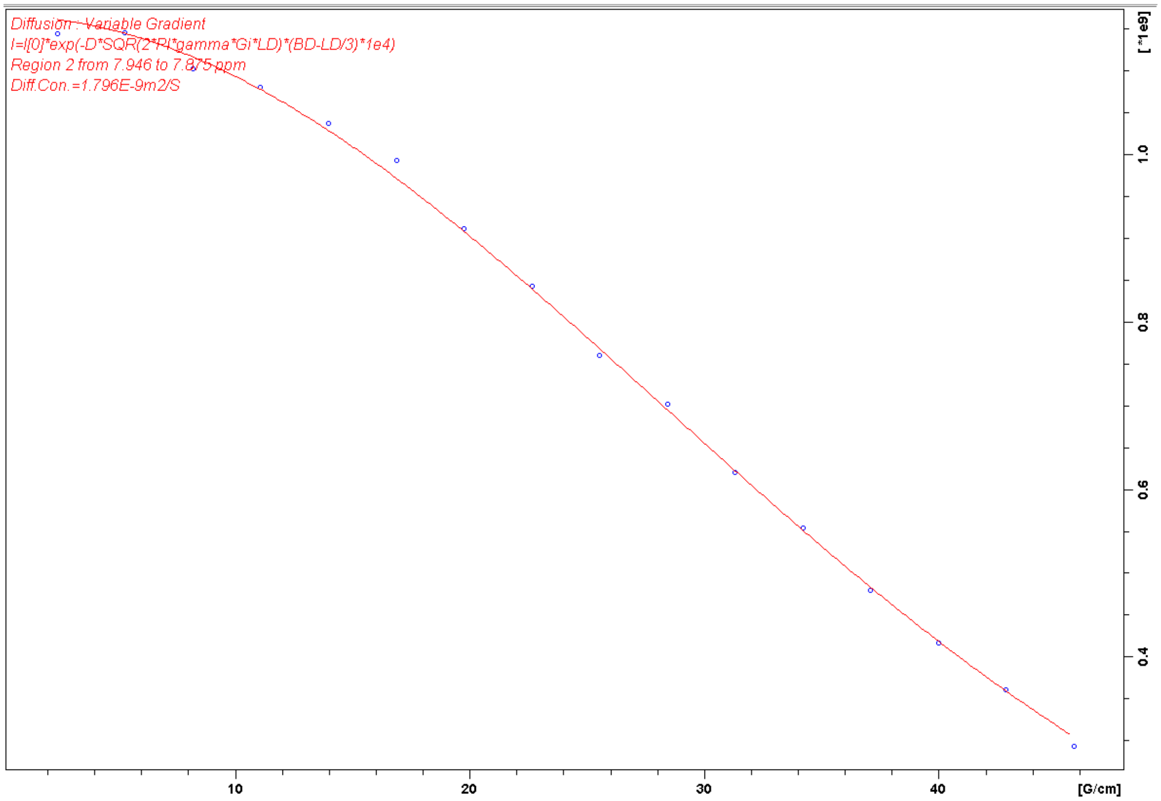
Diffusion : Variable Gradient
I=I[0]*exp(-D*SQR(2*PI*gamma*Gi*LD)*(BD-LD/3)*1e4)
Region 2 from 7.946 to 7.875 ppm
Diff.Con.=1.796E-9m2/S
[*1e9]
1.0
0.8
0.6
0.4
10
20
30
40
[G/cm]

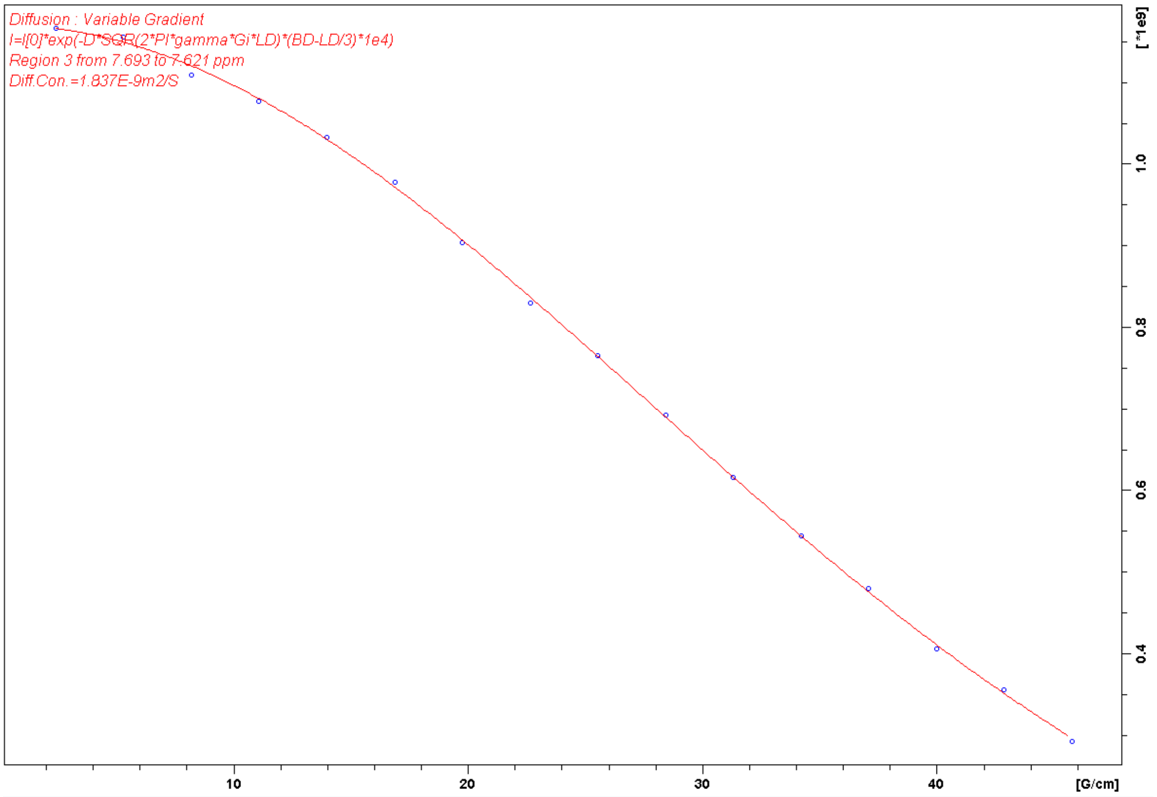
Diffusion : Variable Gradient
I=I[0]*exp(-D*SQR(2*PI*gamma*Gi*LD)*(BD-LD/3)*1e4)
Region 3 from 7.693 to 7.621 ppm
Diff.Con.=1.837E-9m2/S
[*1e9]
1.0
0.8
0.6
0.4
10
20
30
40
[G/cm]

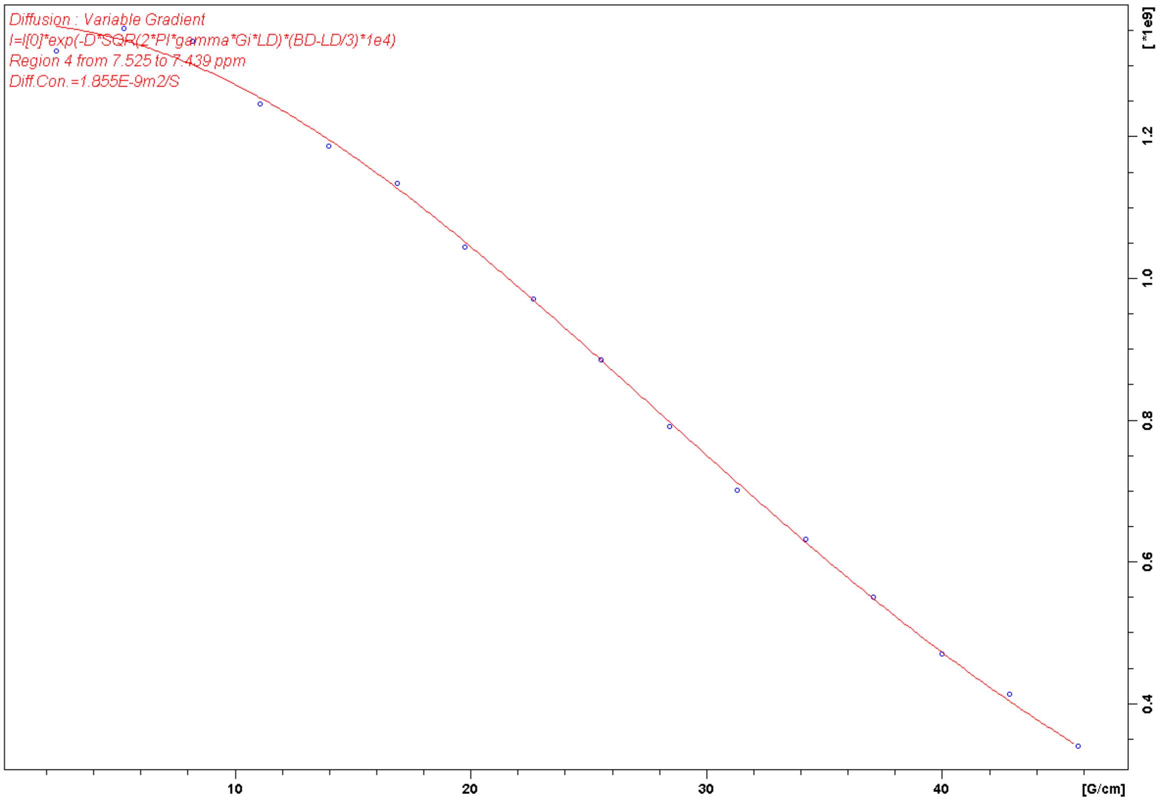
Diffusion : Variable Gradient
I=I[0]*exp(-D*SQR(2*PI*gamma*Gi*LD)*(BD-LD/3)*1e4)
Region 4 from 7.525 to 7.439 ppm
Diff.Con.=1.855E-9m2/S
[*1e9]
1.2
1.0
0.8
0.6
0.4
10
20
30
40
[G/cm]

D TMS

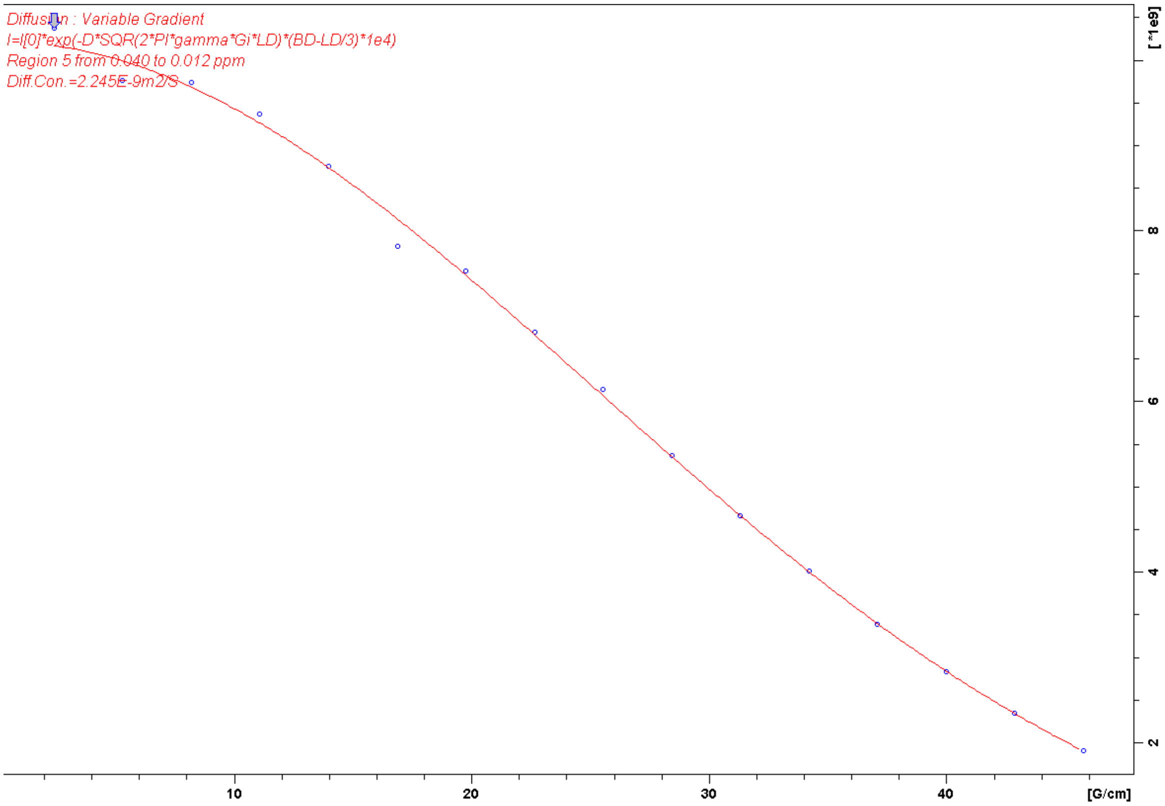
Diffusion : Variable Gradient
I=I[0]*exp(-D*SQR(2*PI*gamma*Gi*LD)*(BD-LD/3)*1e4)
Region 5 from 0.040 to 0.012 ppm
Diff.Con.=2.245E-9m2/S
[*1e9]
8
6
4
2
10
20
30
40
[G/cm]

5.1.9 DOSY Cyclohexanol/TMS/0 eq NFTB

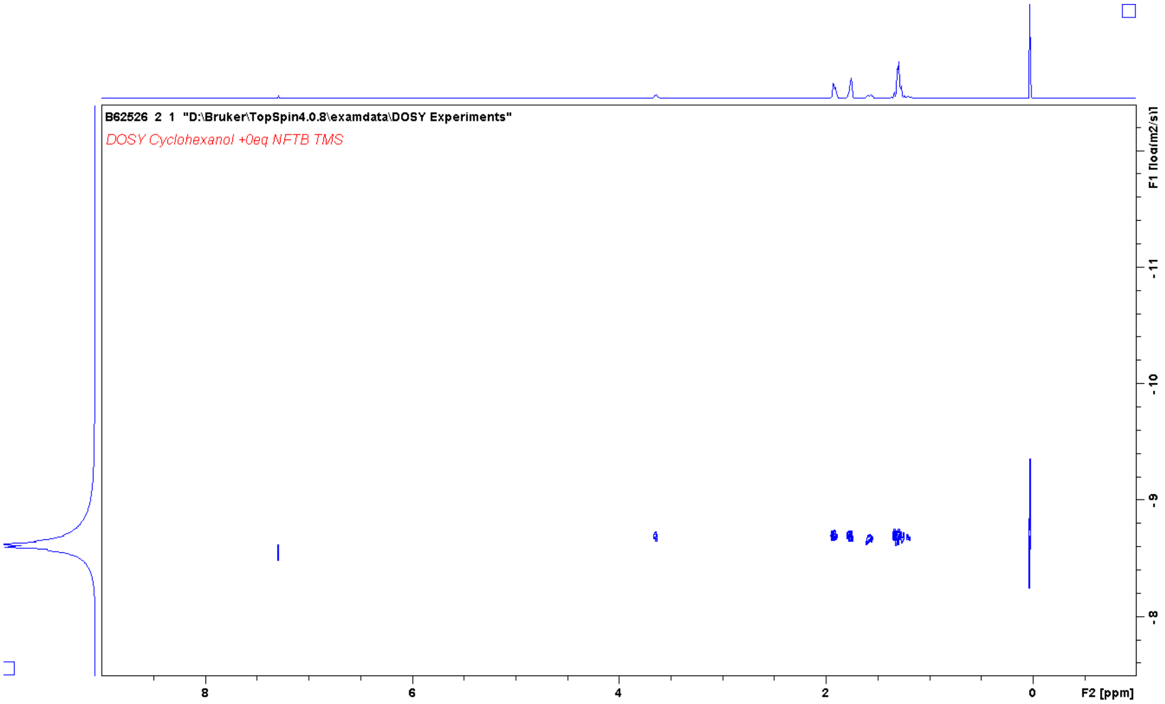

D Cyclohexanol

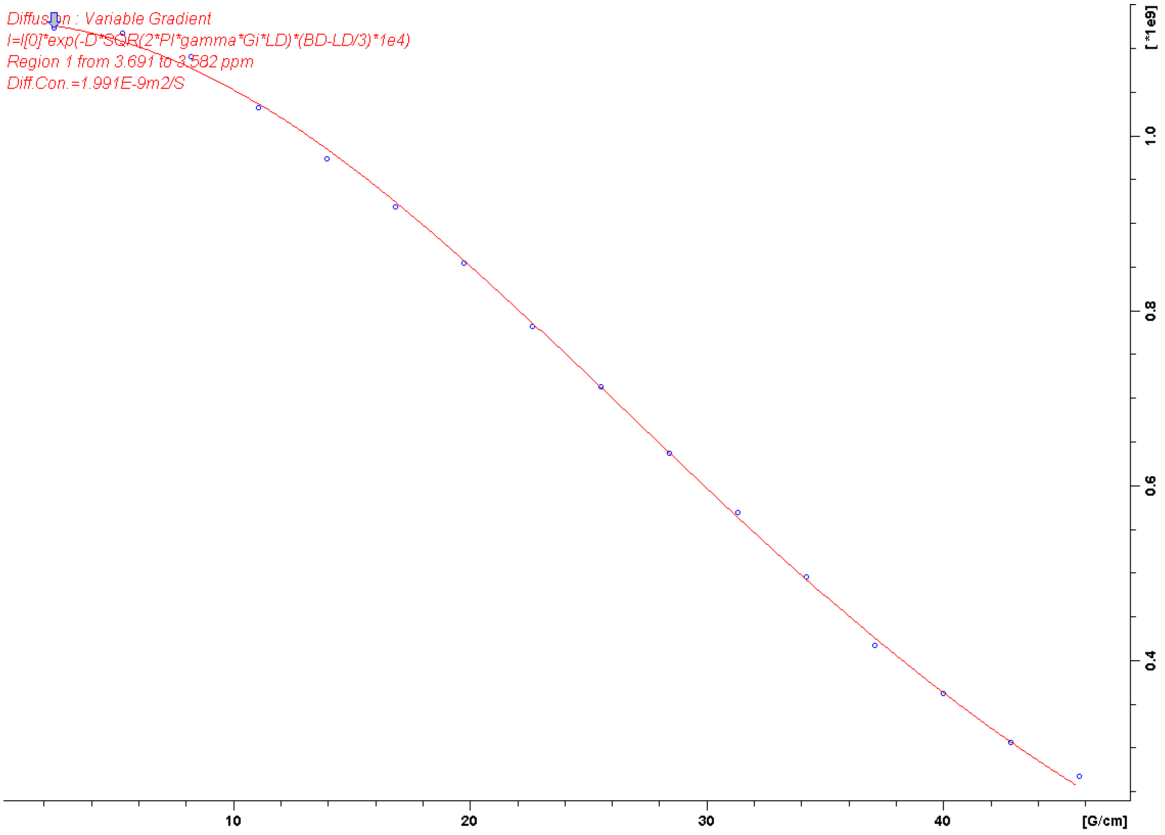

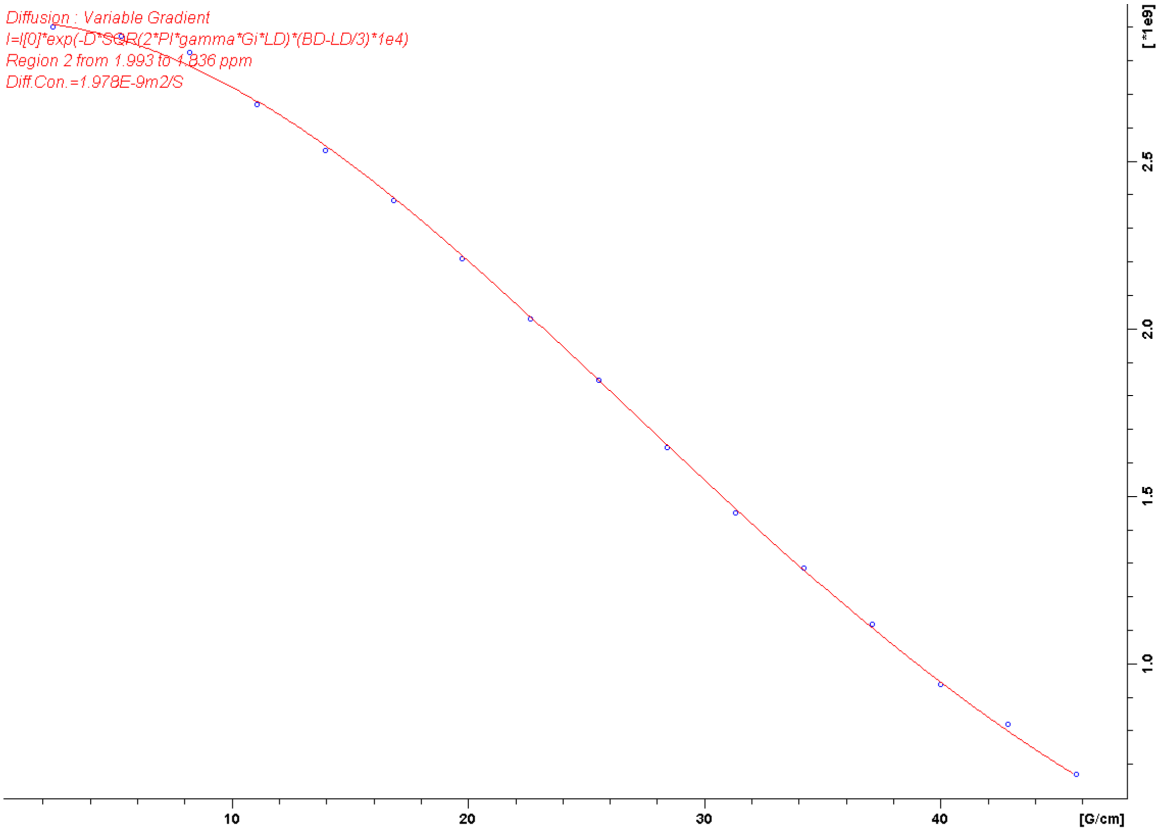
Diffusion : Variable Gradient
I=I[0]*exp(-D*SQR(2*PI*gamma*Gi*LD)*(BD-LD/3)*1e4)
Region 2 from 1.993 to 1.836 ppm
Diff.Con.=1.978E-9m2/S
[*1e9]
2.5
2.0
1.5
1.0
10
20
30
40
[G/cm]

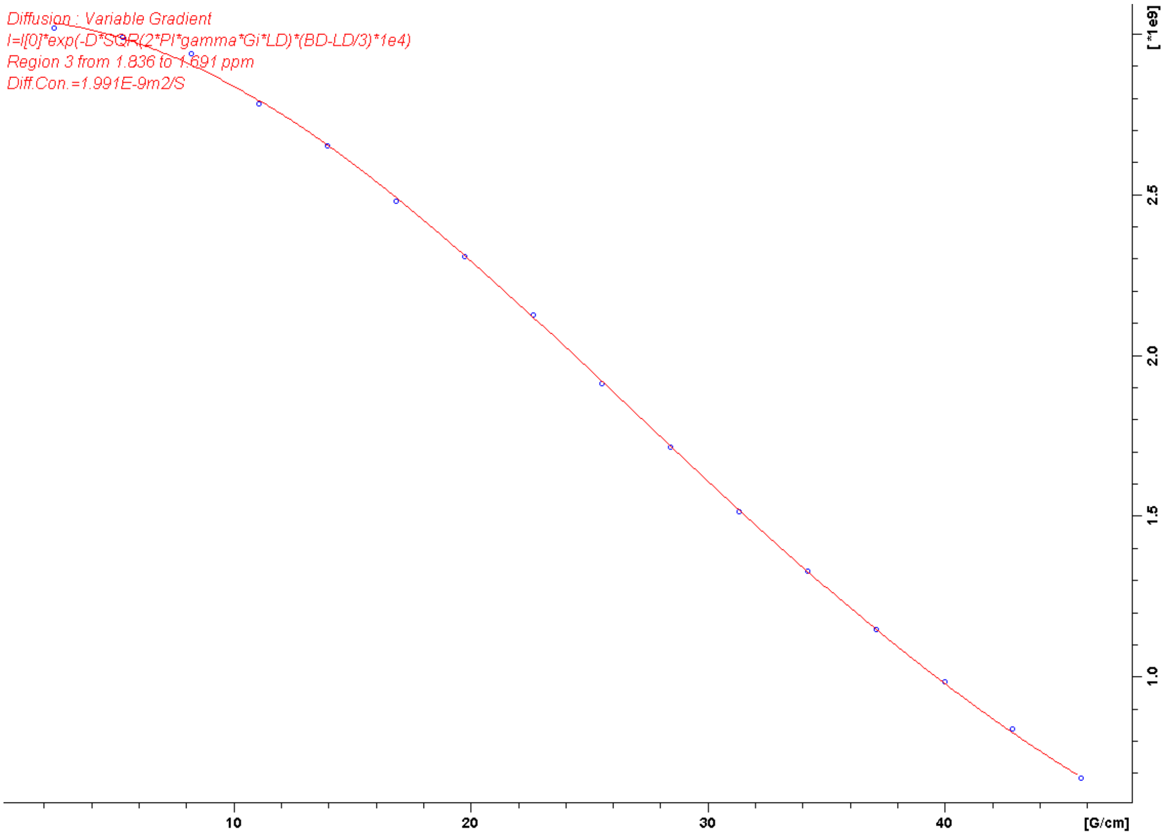
Diffusion : Variable Gradient
I=I[0]*exp(-D*SQR(2*PI*gamma*Gi*LD)*(BD-LD/3)*1e4)
Region 3 from 1.836 to 1.691 ppm
Diff.Con.=1.991E-9m2/S
[*1e9]
2.5
2.0
1.5
1.0
10
20
30
40
[G/cm]

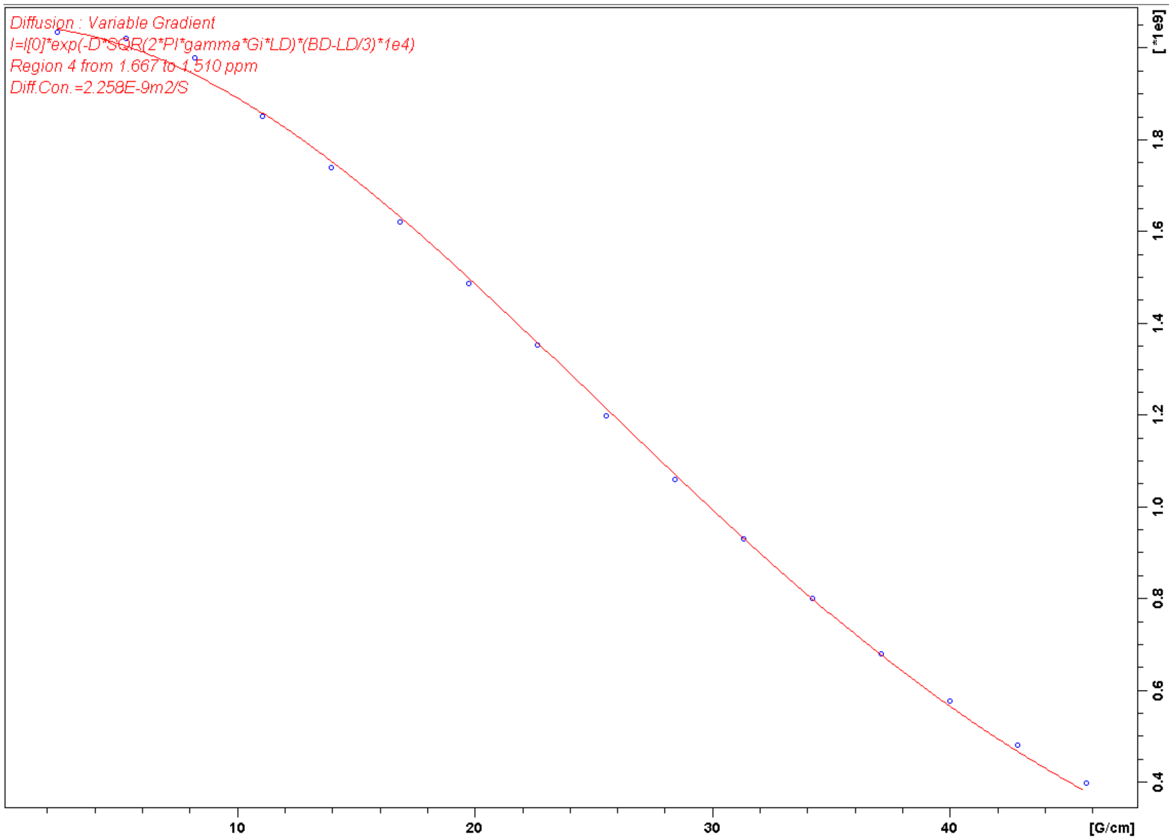
Diffusion : Variable Gradient
I=I[0]*exp(-D*SQR(2*PI*gamma*Gi*LD)*(BD-LD/3)*1e4)
Region 4 from 1.667 to 1.510 ppm
Diff.Con.=2.258E-9m2/S
[*1e9]
1.8
1.6
1.4
1.2
1.0
0.8
0.6
0.4
10
20
30
40
[G/cm]

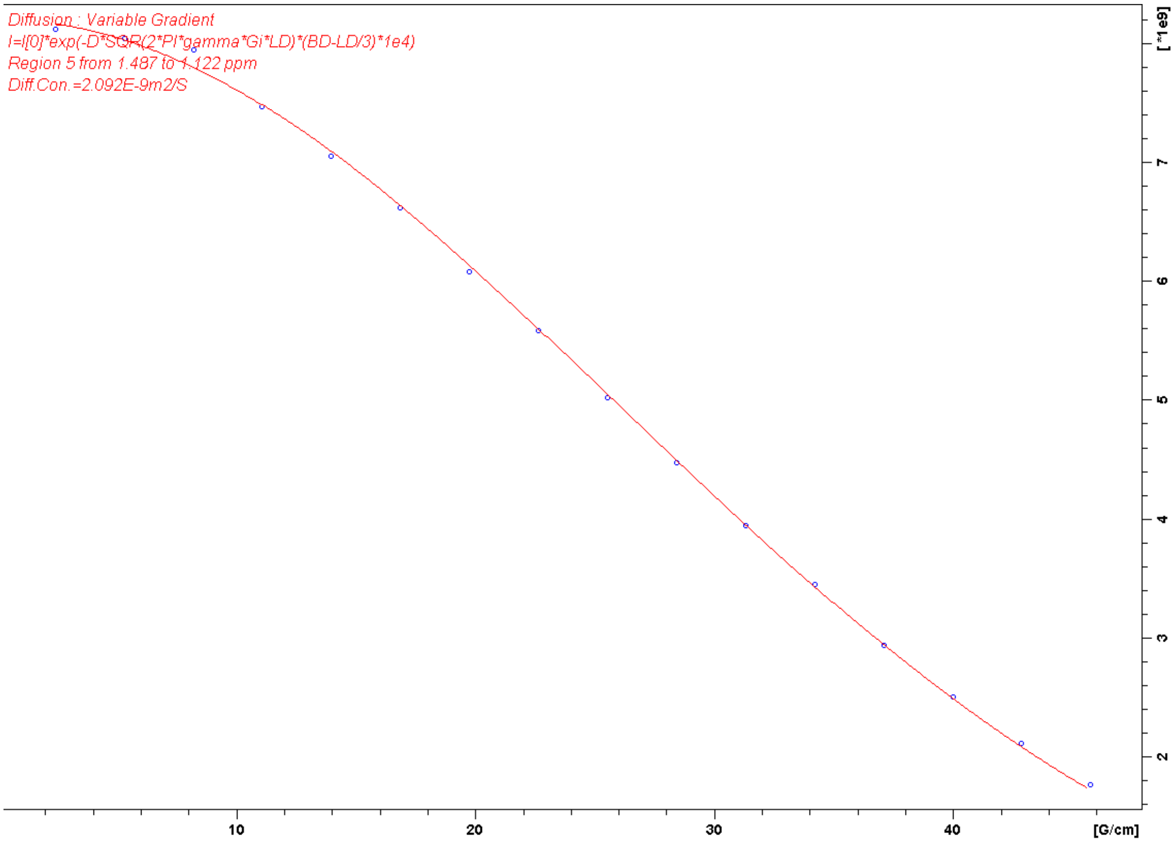
Diffusion : Variable Gradient
I=I[0]*exp(-D*SQR(2*PI*gamma*Gi*LD)*(BD-LD/3)*1e4)
Region 5 from 1.487 to 1.122 ppm
Diff.Con.=2.092E-9m2/S
[*1e9]
7
6
5
4
3
2
10
20
30
40
[G/cm]

D TMS

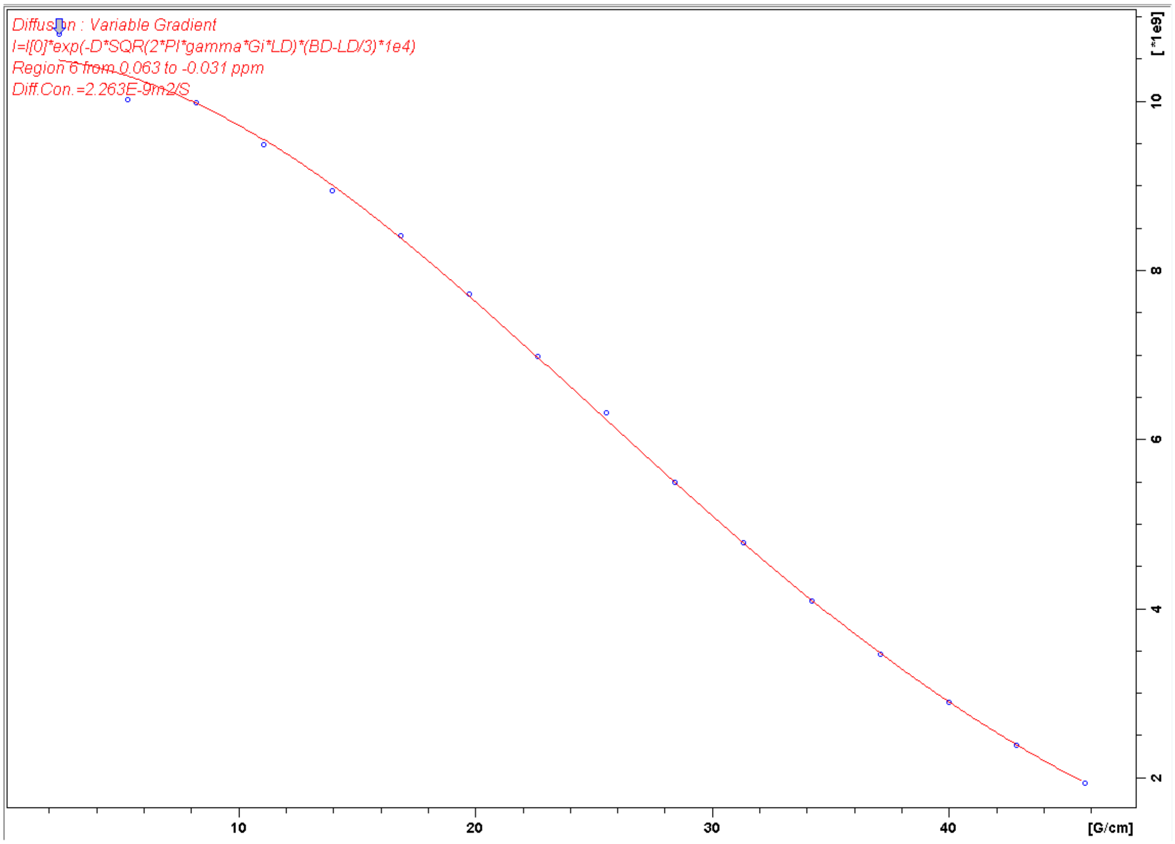
Diffusion : Variable Gradient
I=I[0]*exp(-D*SQR(2*PI*gamma*Gi*LD)*(BD-LD/3)*1e4)
Region 6 from 0.063 to -0.031 ppm
Diff.Con.=2.263E-9m2/S
[*1e9]
10
8
6
4
2
10
20
30
40
[G/cm]

5.1.10 DOSY Cyclohexanol/TMS/1 eq NFTB

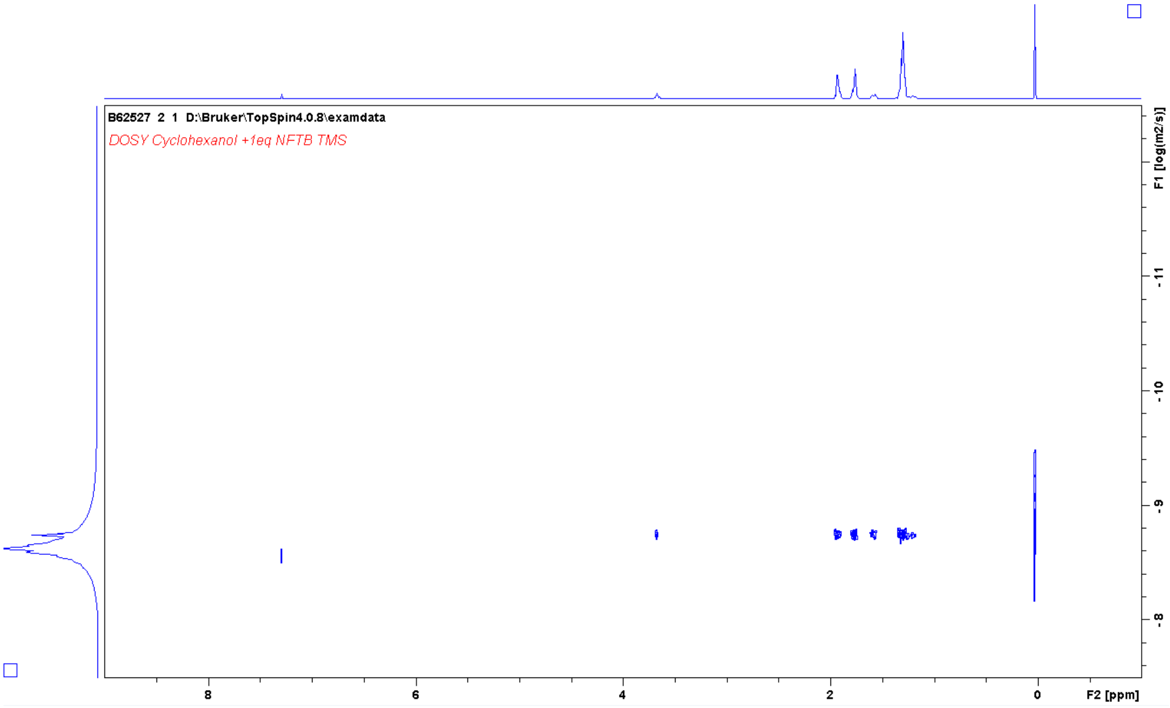

D Cyclohexanol

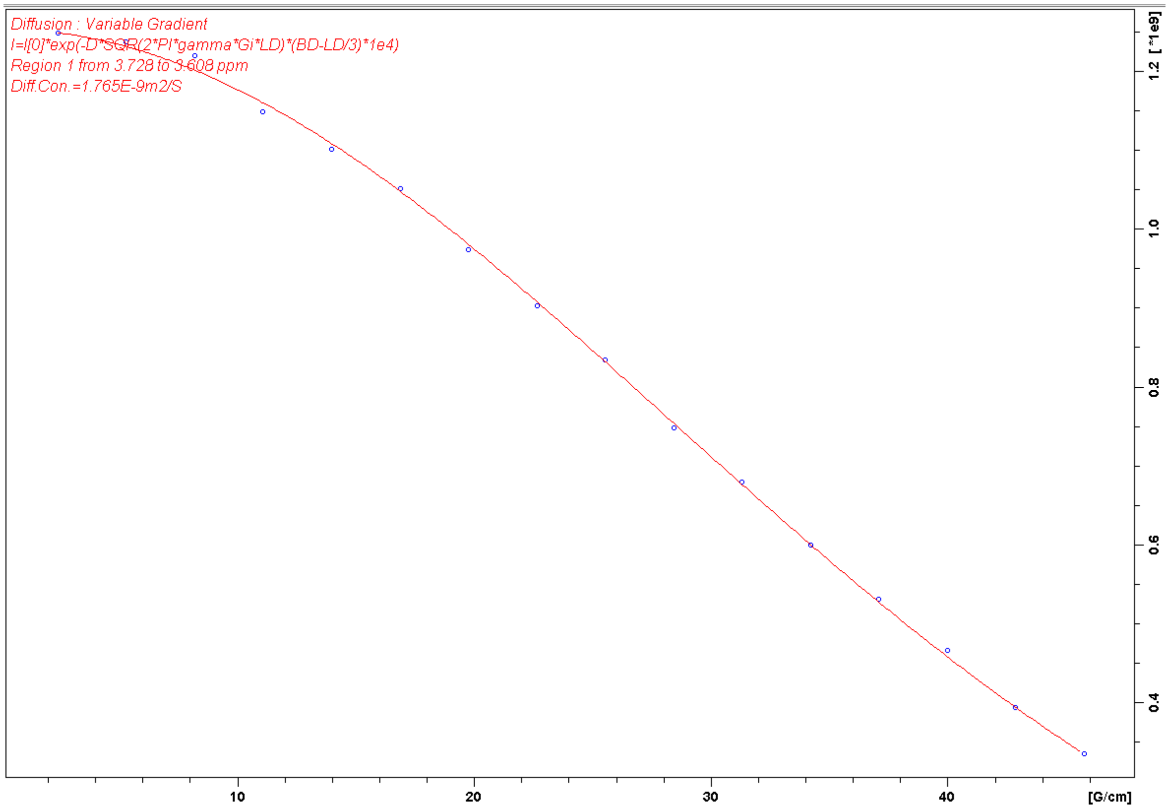

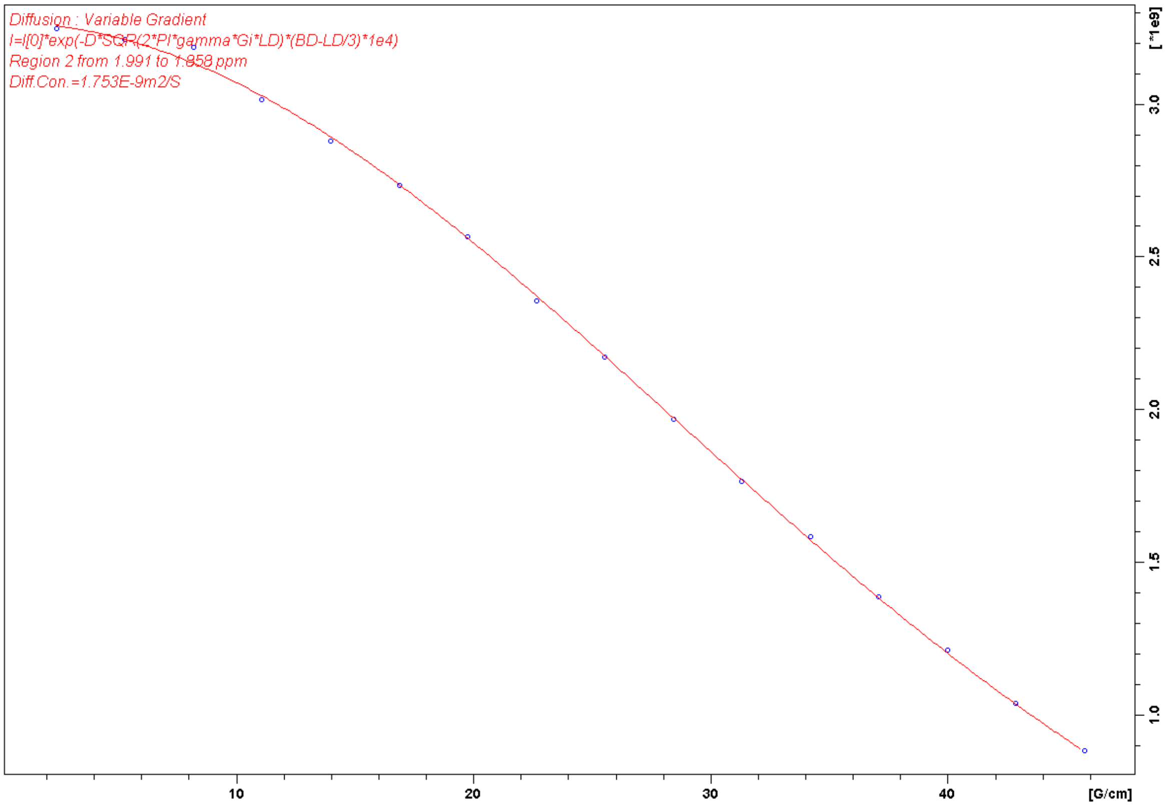

Diffusion : Variable Gradient
I=I[0]*exp(-D*SQR(2*PI*gamma*Gi*LD)*(BD-LD/3)*1e4)
Region 2 from 1.991 to 1.858 ppm
Diff.Con.=1.753E-9m2/S
[*1e9]
3.0
2.5
2.0
1.5
1.0
10
20
30
40
[G/cm]

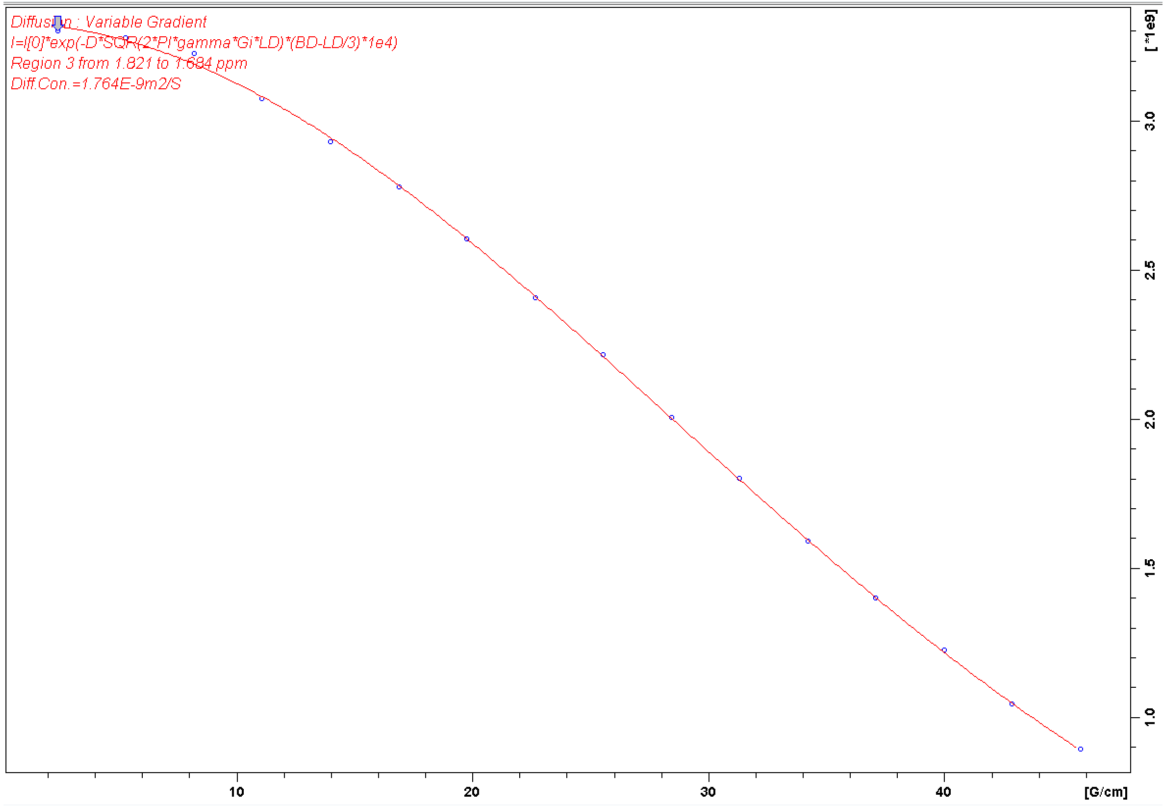

Diffusion : Variable Gradient
I=I[0]*exp(-D*SQR(2*PI*gamma*Gi*LD)*(BD-LD/3)*1e4)
Region 3 from 1.821 to 1.684 ppm
Diff.Con.=1.764E-9m2/S
[*1e9]
3.0
2.5
2.0
1.5
1.0
10
20
30
40
[G/cm]

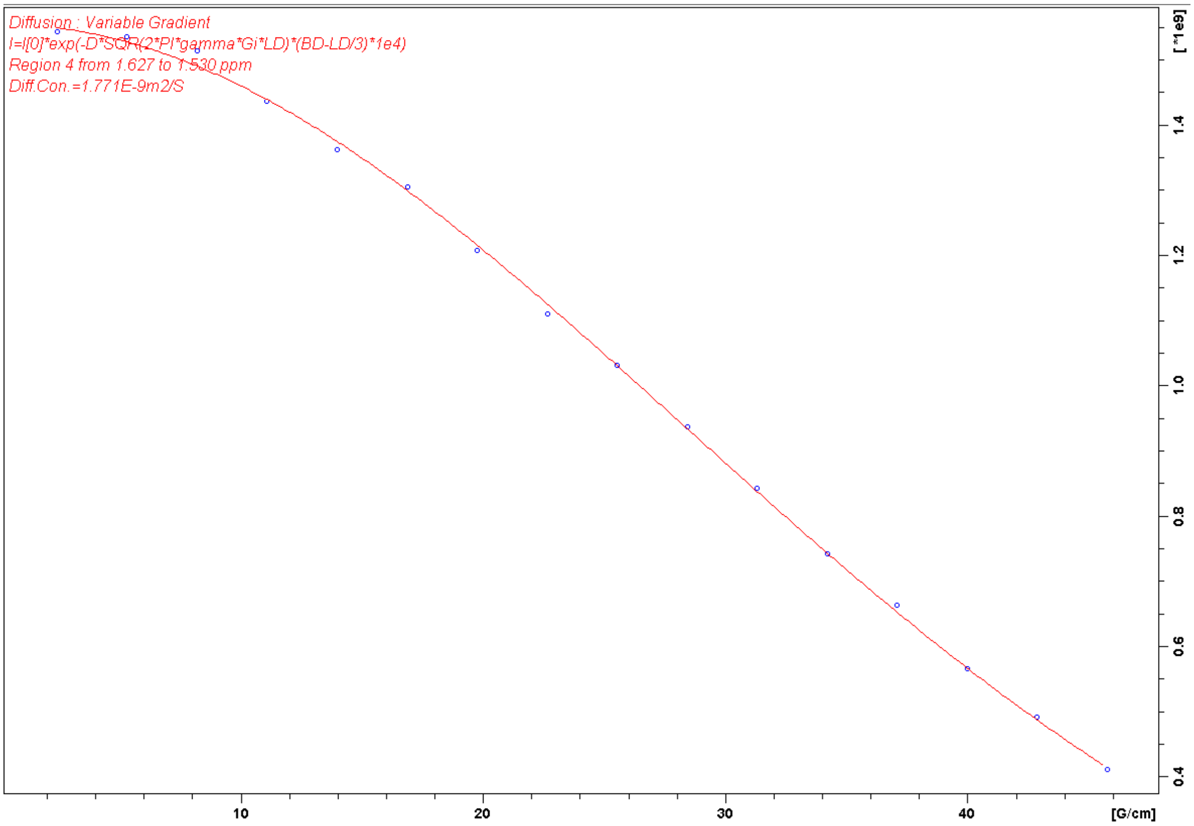
Diffusion : Variable Gradient
I=I[0]*exp(-D*SQR(2*PI*gamma*Gi*LD)*(BD-LD/3)*1e4)
Region 4 from 1.627 to 1.530 ppm
Diff.Con.=1.771E-9m2/S
[*1e9]
1.4
1.2
1.0
0.8
0.6
0.4
10
20
30
40
[G/cm]

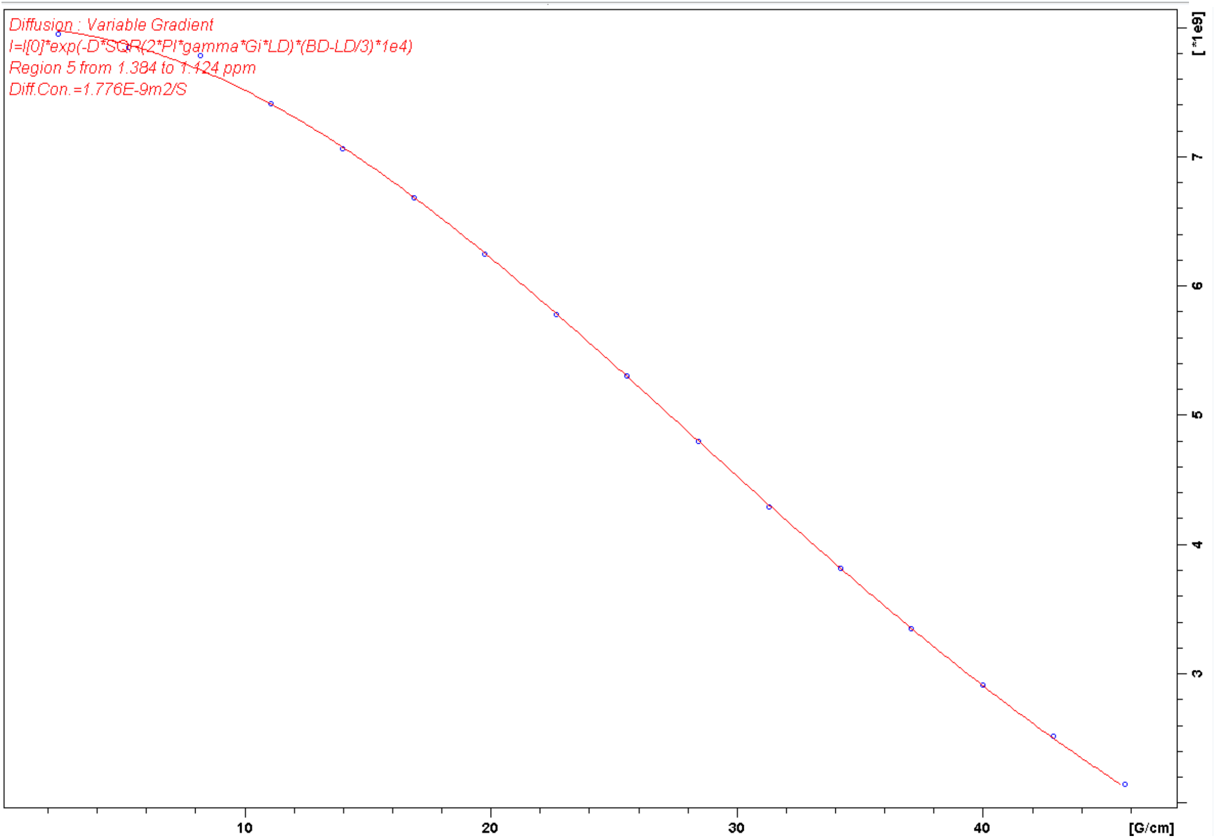
Diffusion : Variable Gradient
I=I[0]*exp(-D*SQR(2*PI*gamma*Gi*LD)*(BD-LD/3)*1e4)
Region 5 from 1.384 to 1.424 ppm
Diff.Con.=1.776E-9m2/S
[*1e9]
7
6
5
4
3
10
20
30
40
[G/cm]

D TMS

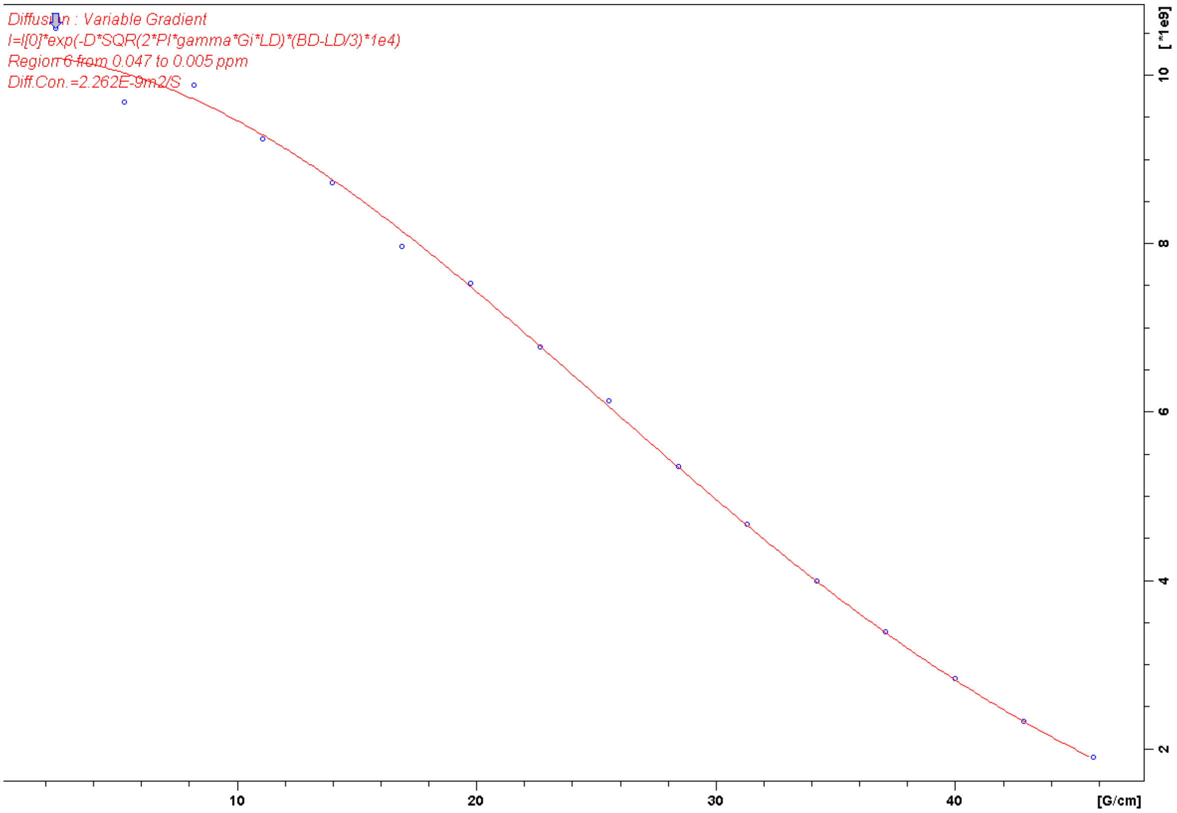
Diffusion : Variable Gradient
I=I[0]*exp(-D*SQR(2*PI*gamma*Gi*LD)*(BD-LD/3)*1e4)
Region 6 from 0.047 to 0.005 ppm
Diff.Con.=2.262E-9m2/S
[*1e9]
10
8
6
4
2
10
20
30
40
[G/cm]

5.1.11 Cyclohexanol/TMS/2 eq NFTB

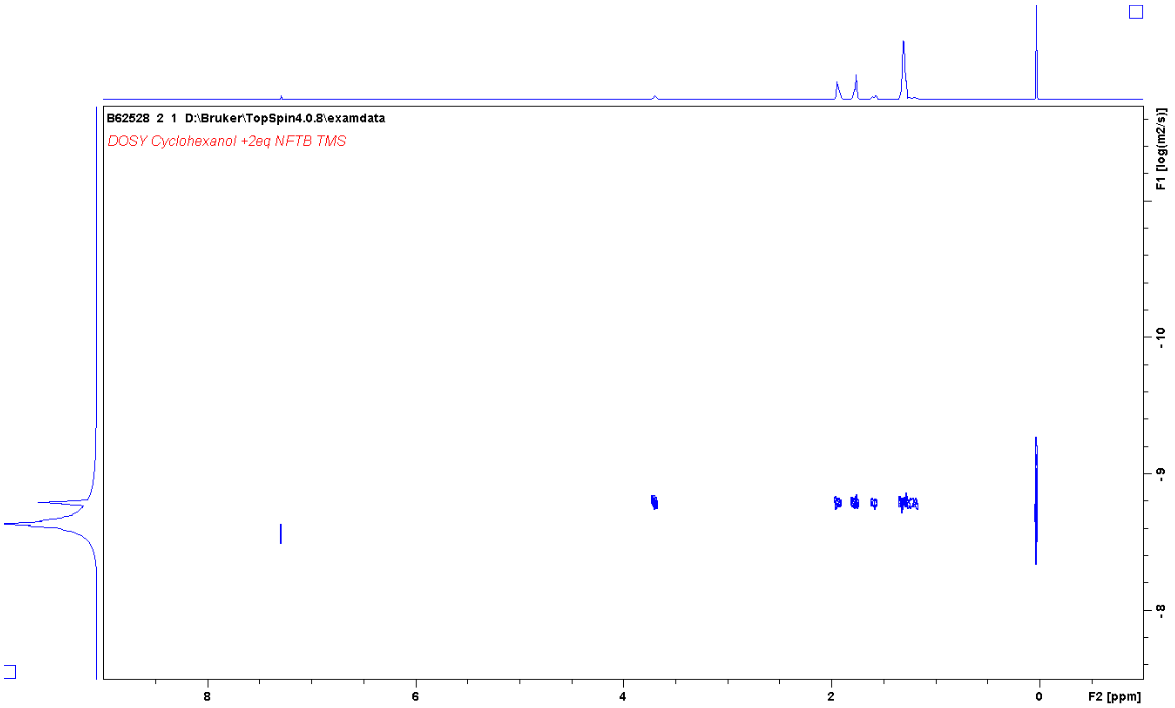

D Cyclohexanol

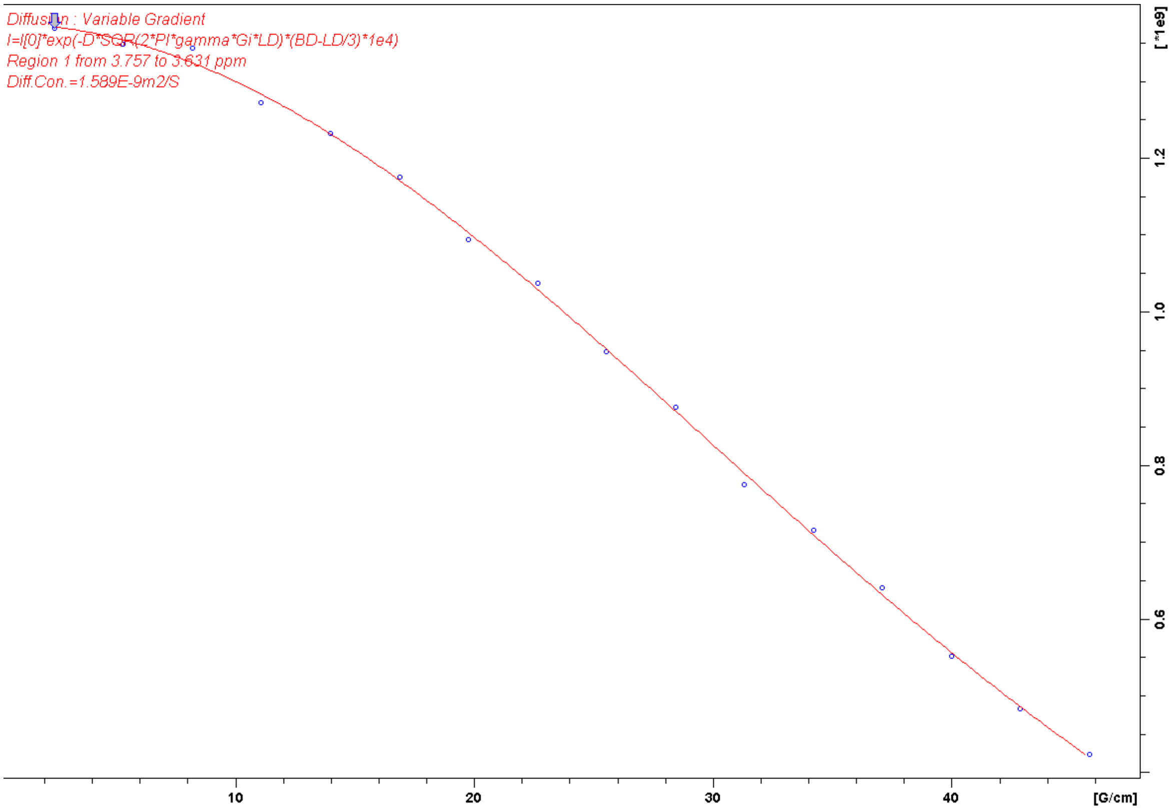

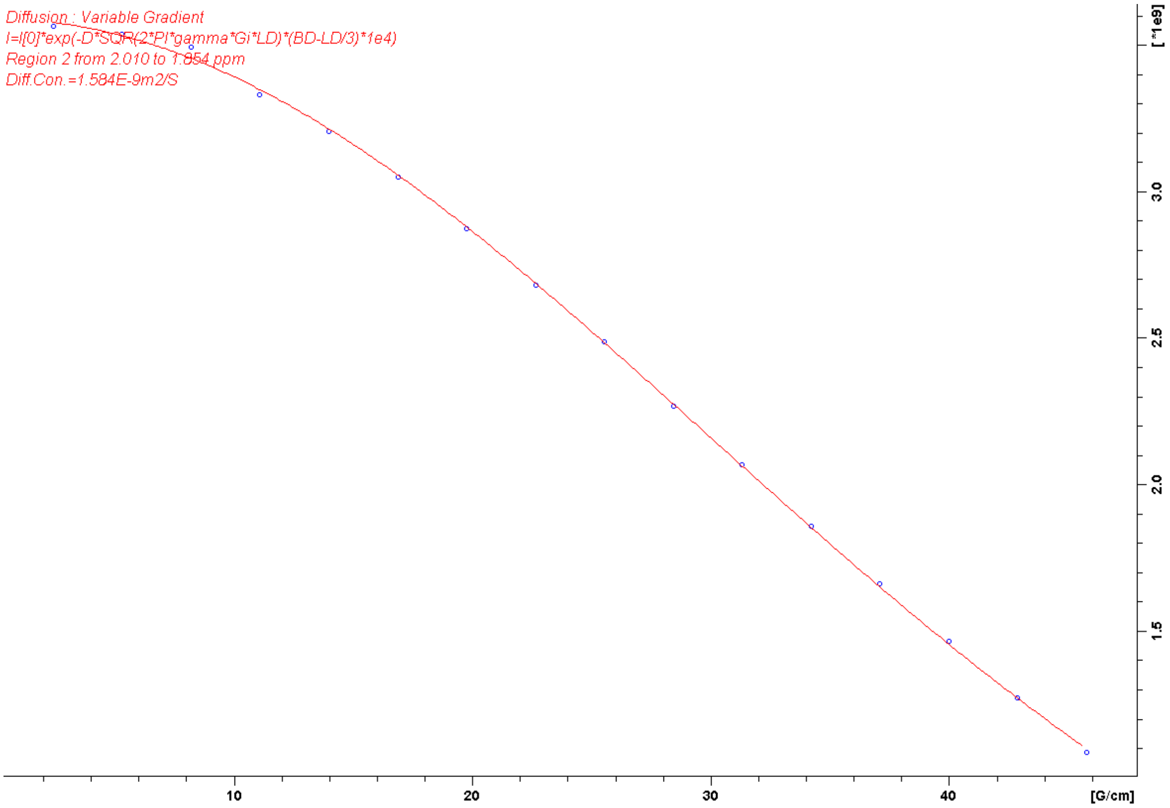
Diffusion : Variable Gradient
I=I[0]*exp(-D*SQR(2*PI*gamma*Gi*LD)*(BD-LD/3)*1e4)
Region 2 from 2.010 to 1.854 ppm
Diff.Con.=1.584E-9m2/S
[*1e9]
3.0
2.5
2.0
1.5
10
20
30
40
[G/cm]

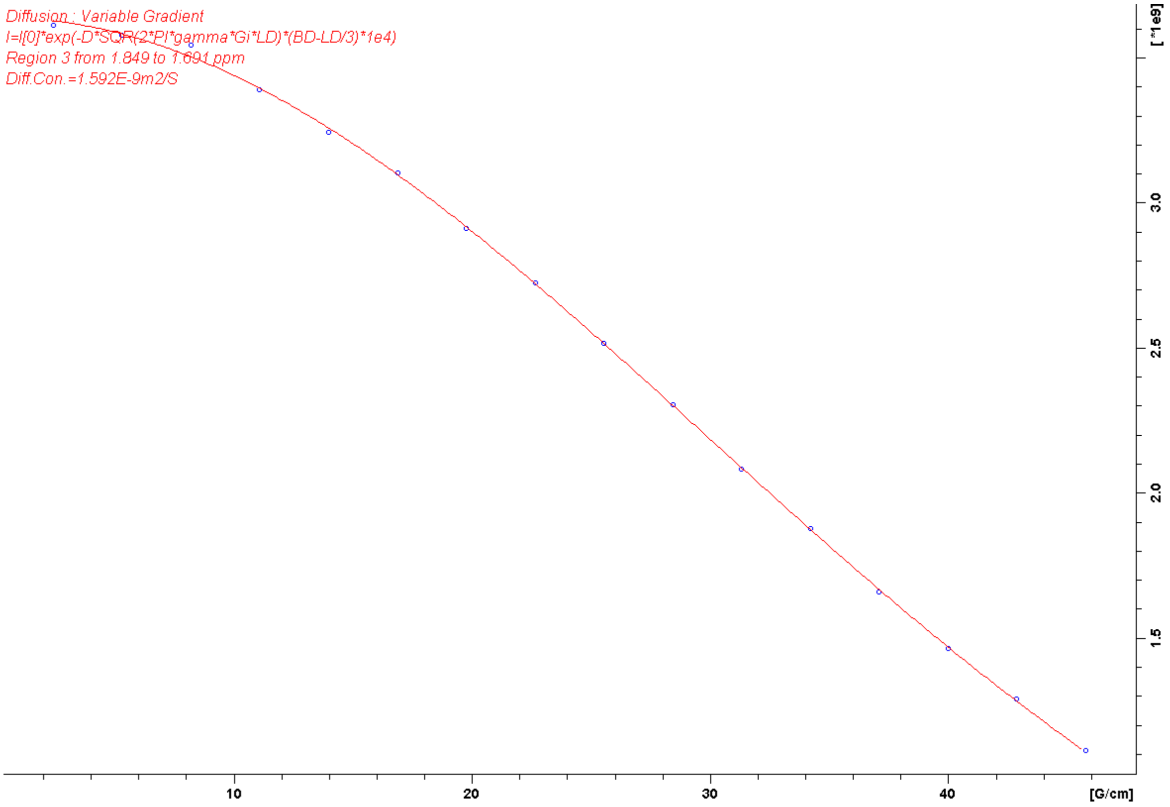
Diffusion : Variable Gradient
I=I[0]*exp(-D*SQR(2*PI*gamma*Gi*LD)*(BD-LD/3)*1e4)
Region 3 from 1.849 to 1.691 ppm
Diff.Con.=1.592E-9m2/S
[*1e9]
3.0
2.5
2.0
1.5
10
20
30
40
[G/cm]

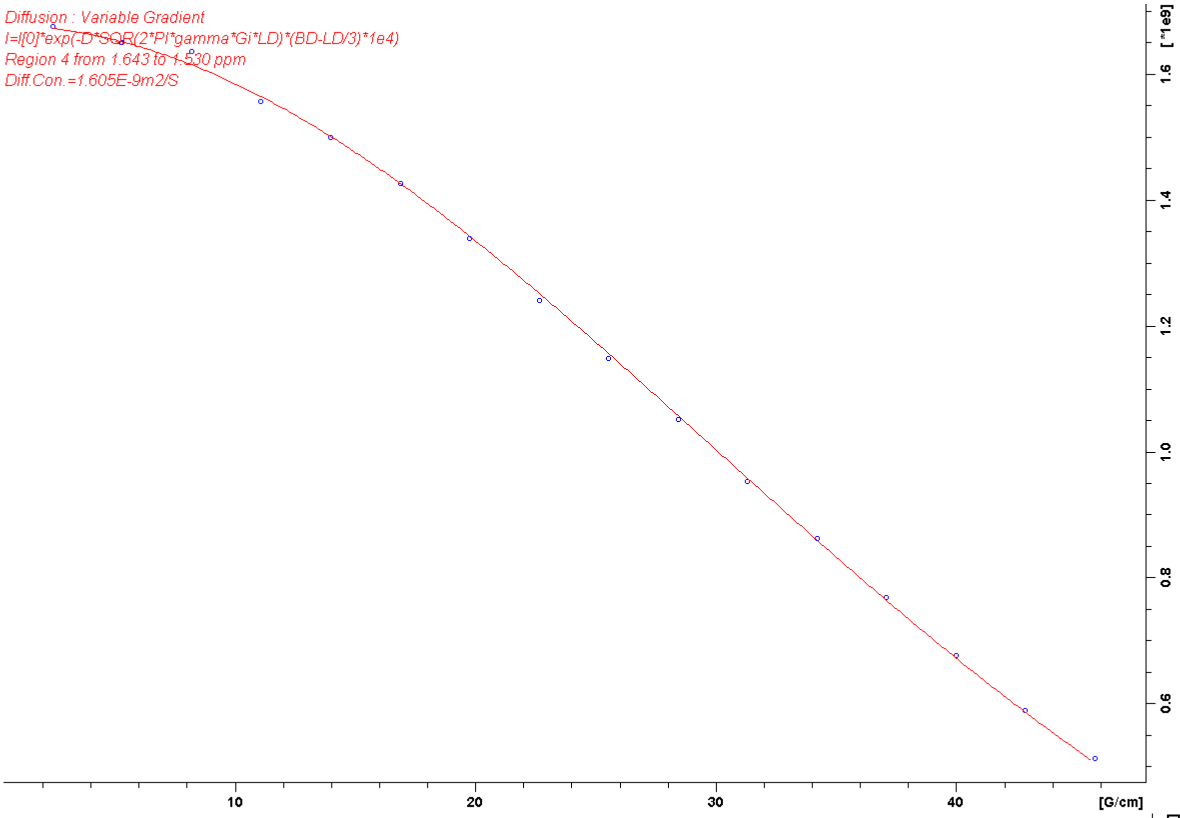
Diffusion : Variable Gradient
I=I[0]*exp(-D*SQR(2*PI*gamma*Gi*LD)*(BD-LD/3)*1e4)
Region 4 from 1.643 to 1.530 ppm
Diff.Con.=1.605E-9m2/S
[*1e9]
1.6
1.4
1.2
1.0
0.8
0.6
10
20
30
40
[G/cm]

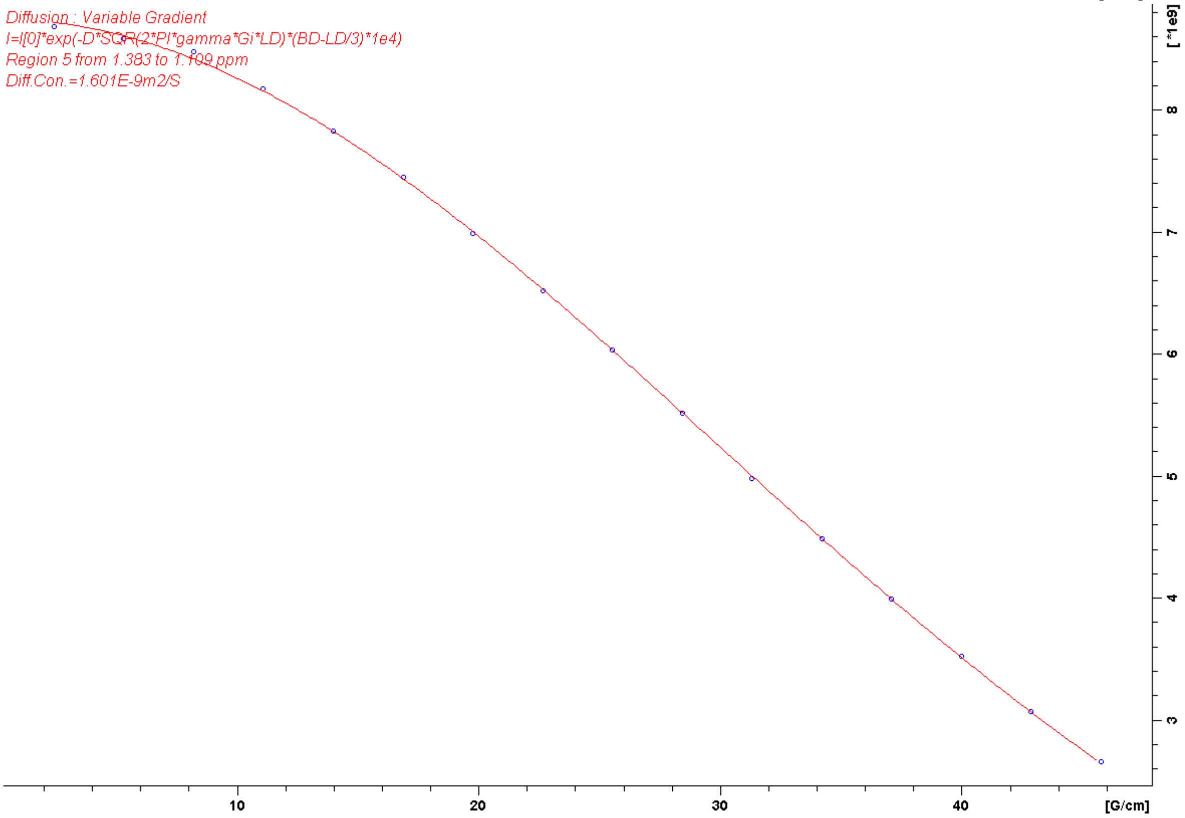
Diffusion : Variable Gradient
I=I[0]*exp(-D*SQR(2*PI*gamma*Gi*LD)*(BD-LD/3)*1e4)
Region 5 from 1.383 to 1.109 ppm
Diff.Con.=1.601E-9m2/S
[*1e9]
8
7
6
5
4
3
10
20
30
40
[G/cm]

D TMS

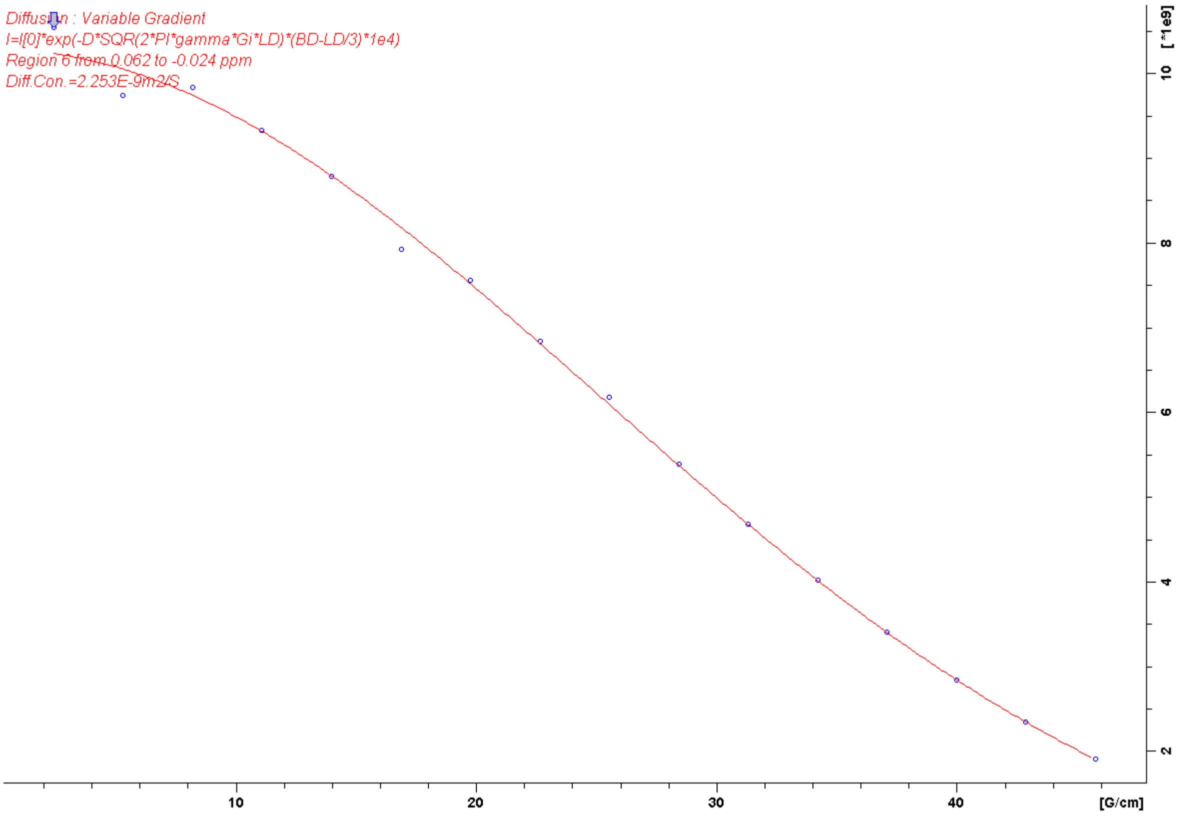

Diffusion : Variable Gradient
I=I[0]*exp(-D*SQR(2*PI*gamma*Gi*LD)*(BD-LD/3)*1e4)
Region 6 from 0.062 to -0.024 ppm
Diff.Con.=2.253E-9m2/S
[*1e9]
10
8
6
4
2
10
20
30
40
[G/cm]

5.1.12 DOSY Cyclohexanol/TMS/3 eq NFTB

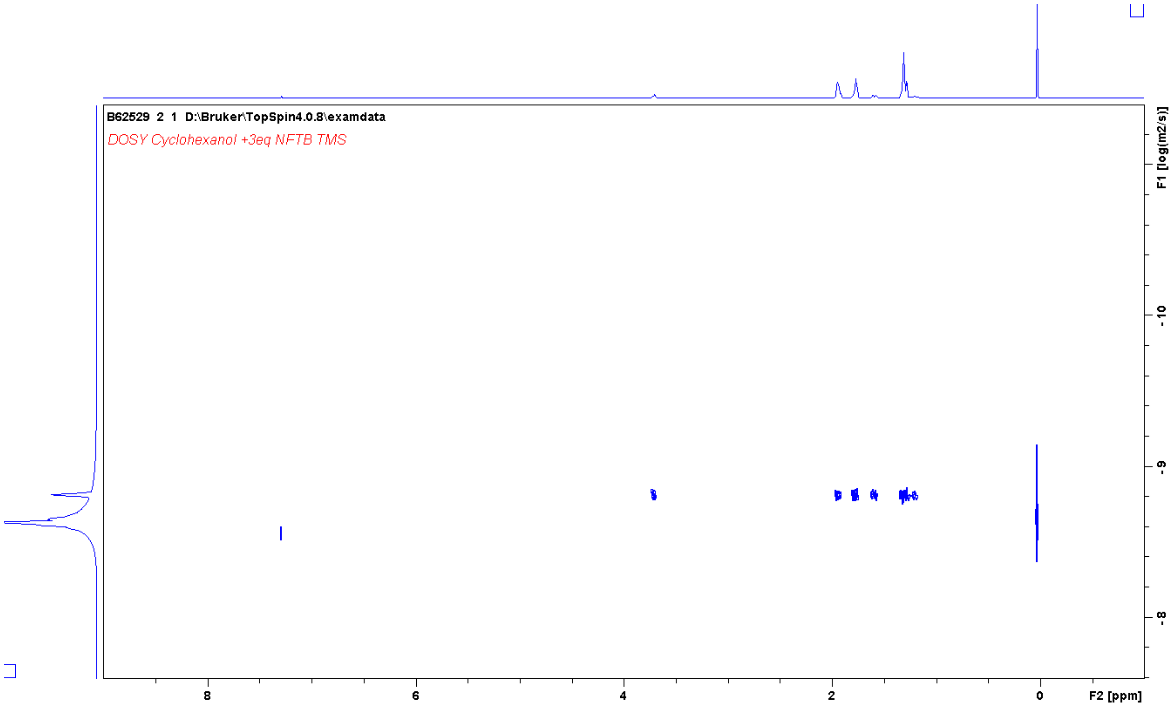

D Cyclohexanol

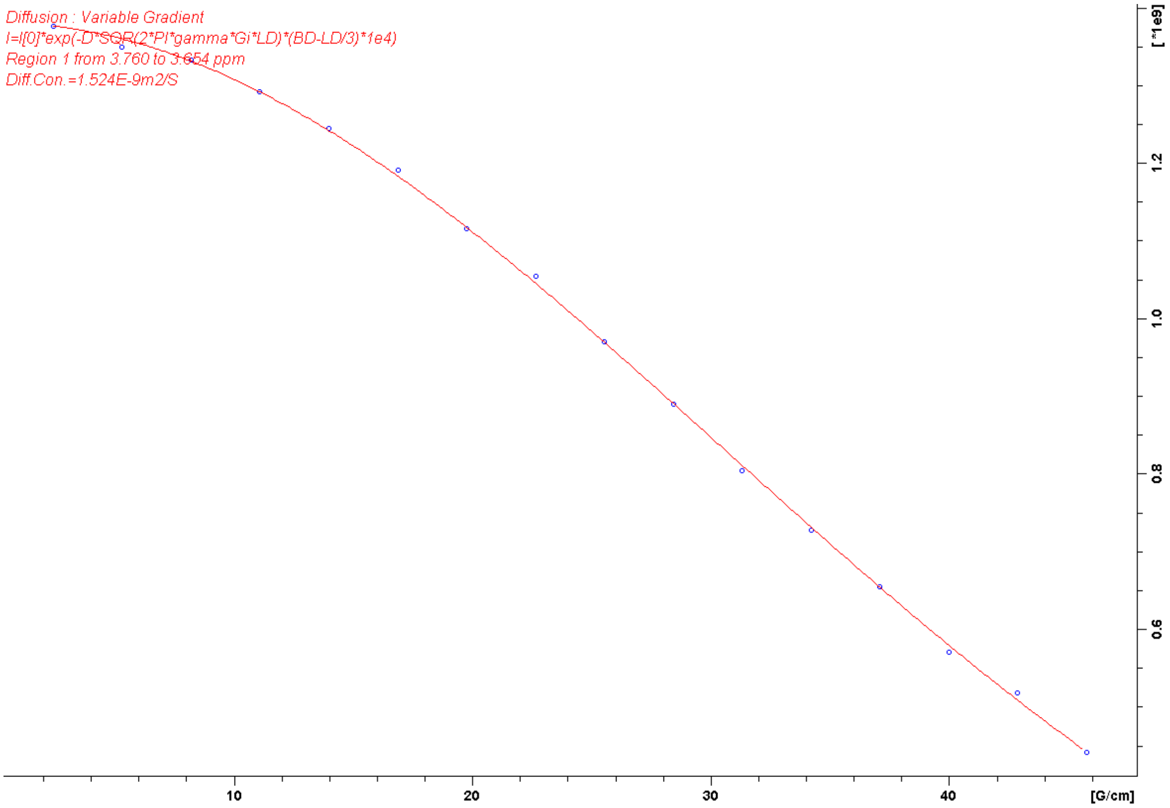

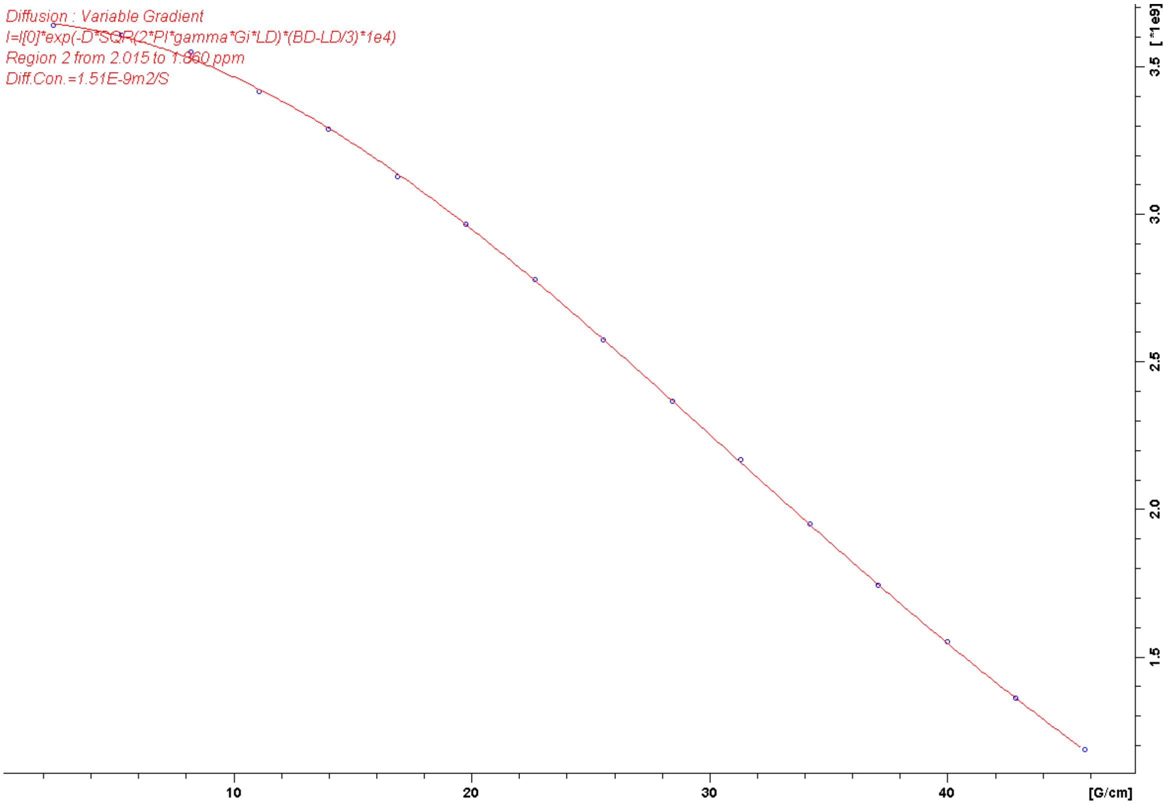
Diffusion : Variable Gradient
I=I[0]*exp(-D*SQR(2*PI*gamma*Gi*LD)*(BD-LD/3)*1e4)
Region 2 from 2.015 to 1.860 ppm
Diff.Con.=1.51E-9m2/S
[*1e9]
3.5
3.0
2.5
2.0
1.5
10
20
30
40
[G/cm]

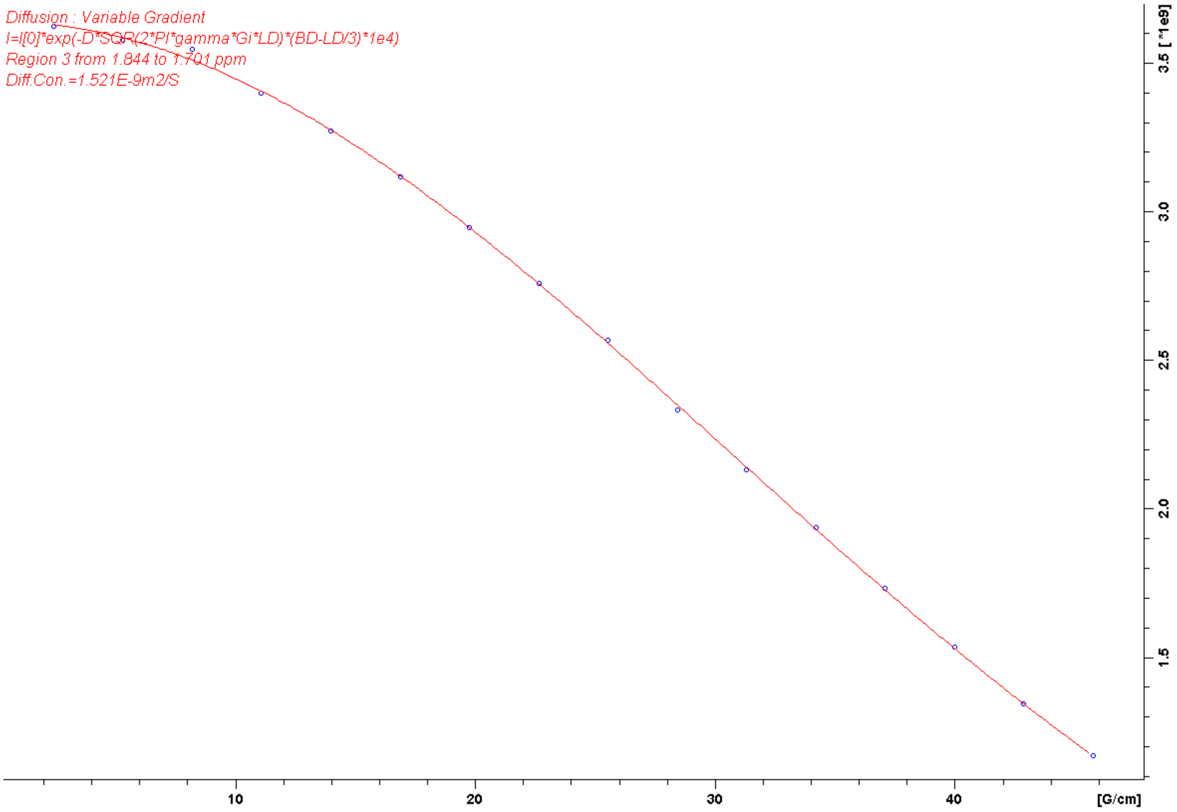
Diffusion : Variable Gradient
I=I[0]*exp(-D*SQR(2*PI*gamma*Gi*LD)*(BD-LD/3)*1e4)
Region 3 from 1.844 to 1.701 ppm
Diff.Con.=1.521E-9m2/S
[*1e9]
3.5
3.0
2.5
2.0
1.5
10
20
30
40
[G/cm]

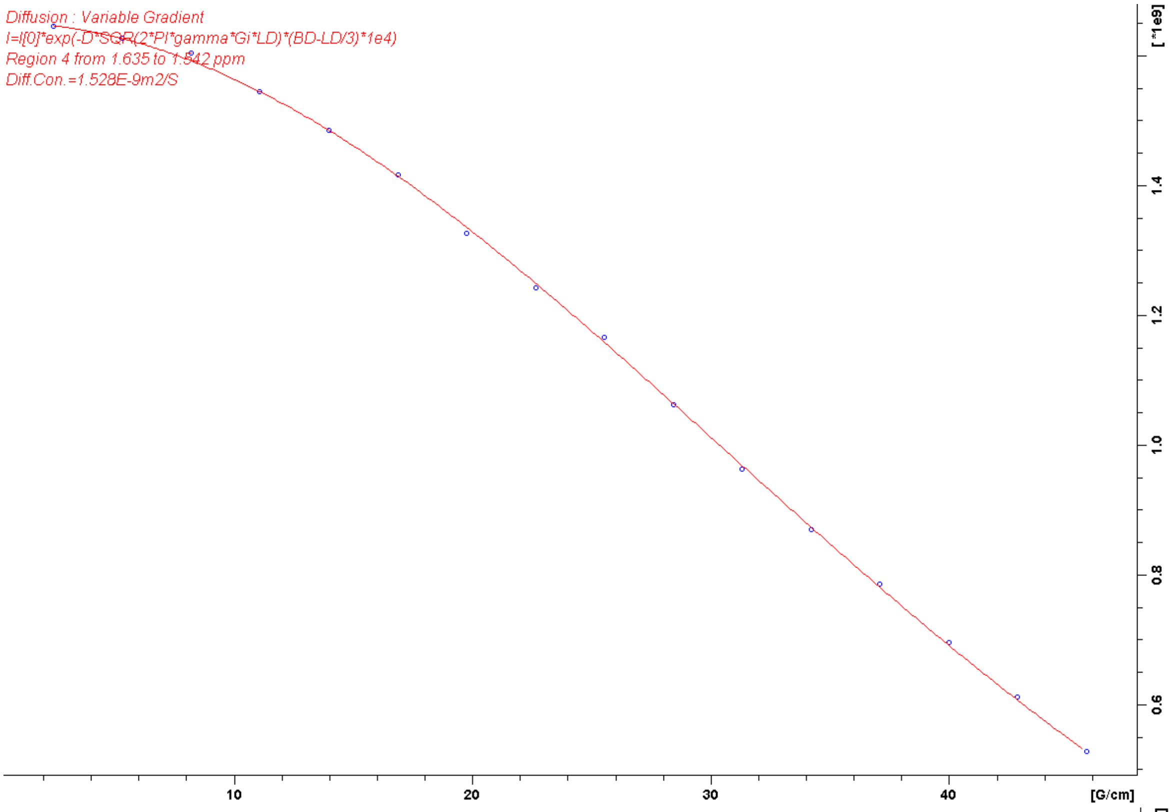
Diffusion : Variable Gradient
I=I[0]*exp(-D*SQR(2*PI*gamma*Gi*LD)*(BD-LD/3)*1e4)
Region 4 from 1.635 to 1.542 ppm
Diff.Con.=1.528E-9m2/S
[*1e9]
1.4
1.2
1.0
0.8
0.6
10
20
30
40
[G/cm]

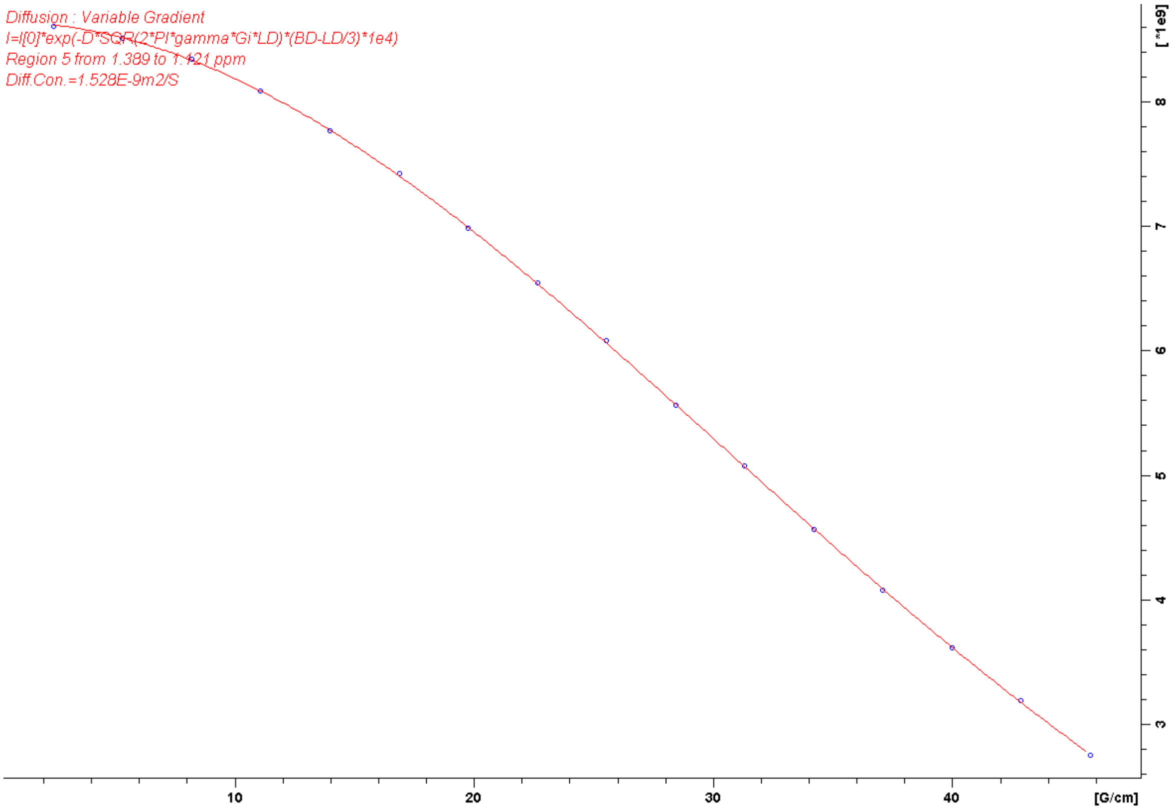
Diffusion : Variable Gradient
I=I[0]*exp(-D*SQR(2*PI*gamma*Gi*LD)*(BD-LD/3)*1e4)
Region 5 from 1.389 to 1.121 ppm
Diff.Con.=1.528E-9m2/S
[*1e9]
8
7
6
5
4
3
10
20
30
40
[G/cm]

D TMS

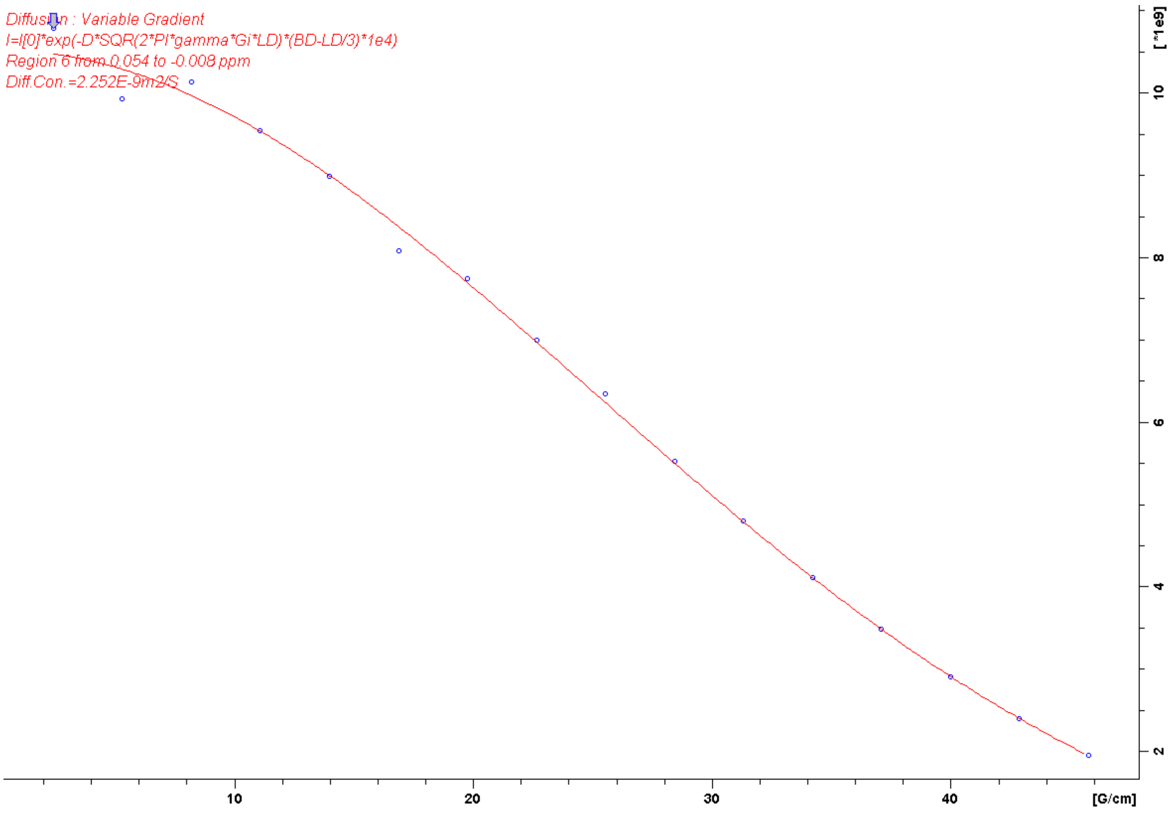

Diffusion : Variable Gradient
I=I[0]*exp(-D*SQR(2*PI*gamma*Gi*LD)*(BD-LD/3)*1e4)
Region 6 from 0.054 to -0.008 ppm
Diff.Con.=2.252E-9m2/S
[*1e9]
10
8
6
4
2
10
20
30
40
[G/cm]

THF TMS

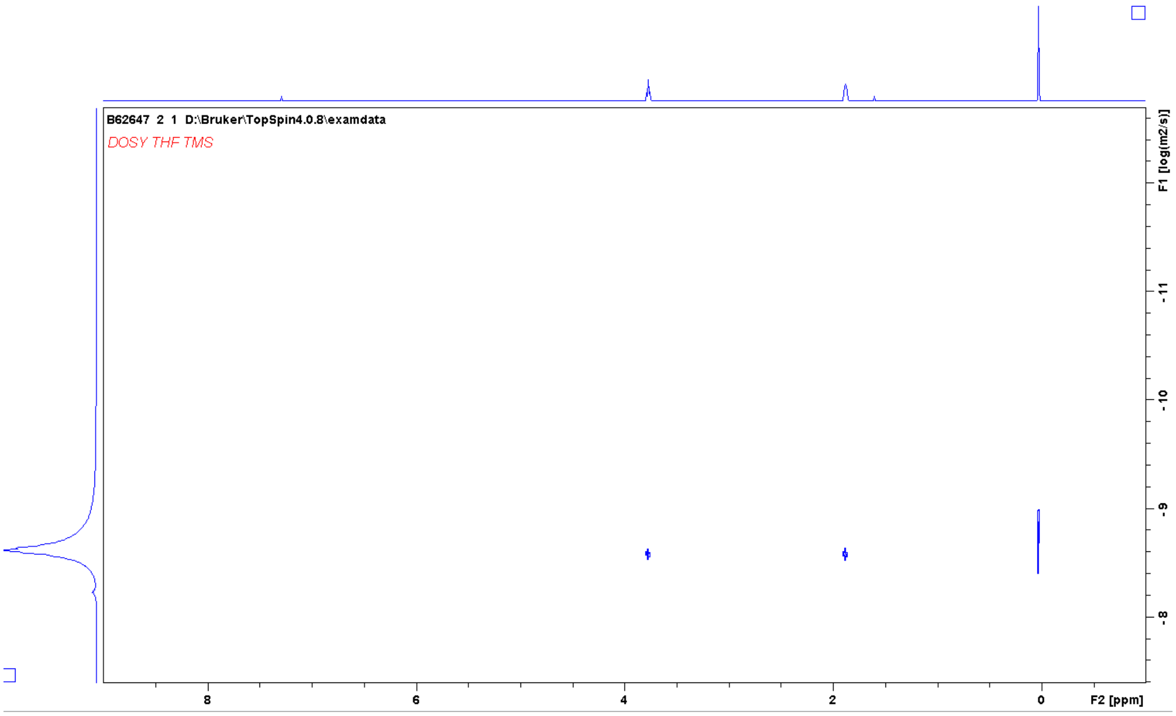

B62647 2 1 D:\Bruker\TopSpin4.0.8\examdata
DOSY THF TMS
F1 [log(m2/s)]
-11
-10
-9
-8
8
6
4
2
0
F2 [ppm]

D THF

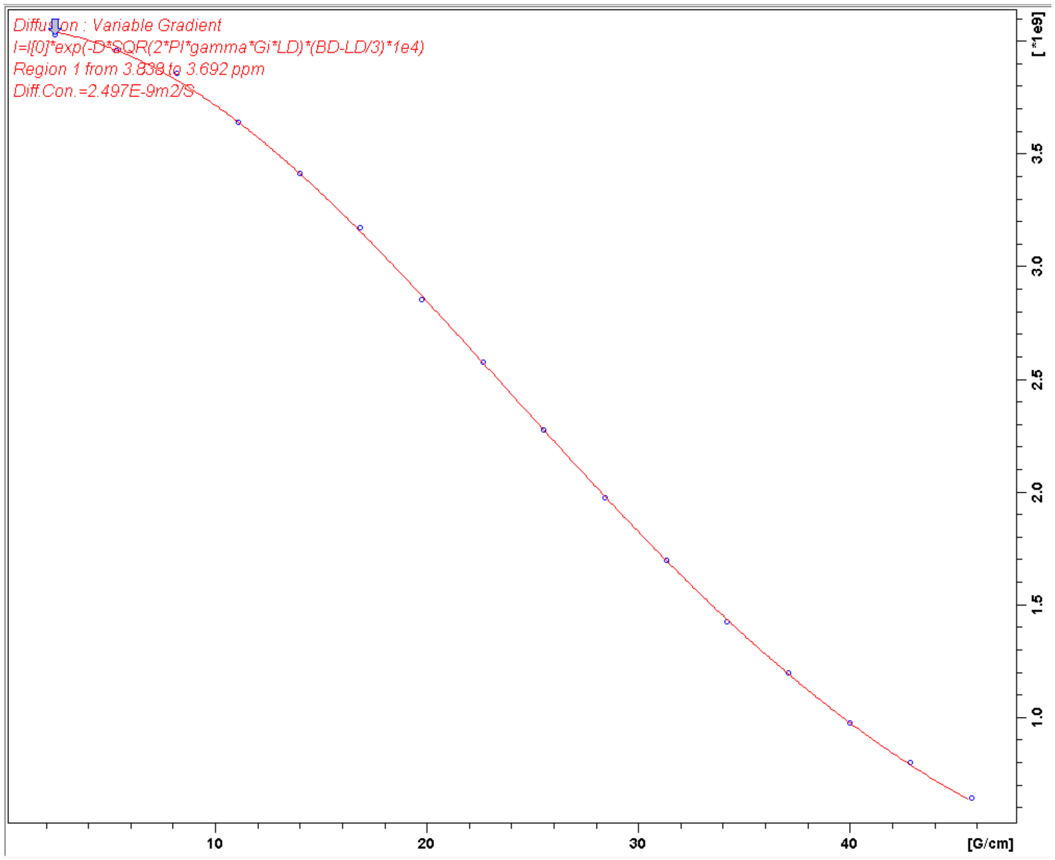

Diffusion : Variable Gradient
I=I[0]*exp(-D*SQR(2*PI*gamma*Gi*LD)*(BD-LD/3)*1e4)
Region 1 from 3.838 to 3.692 ppm
Diff.Con.=2.497E-9m2/S
[*1e9]
3.5
3.0
2.5
2.0
1.5
1.0
10
20
30
40
[G/cm]

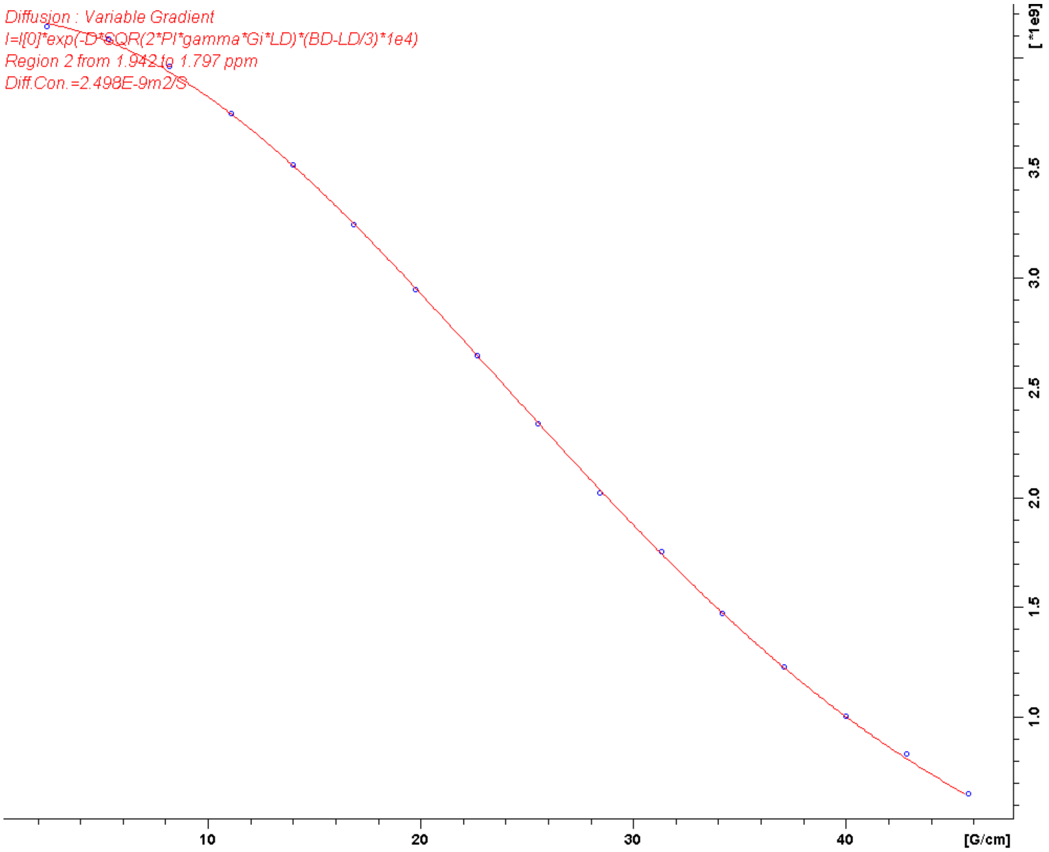
Diffusion : Variable Gradient
I=I[0]*exp(-D*SQR(2*PI*gamma*Gi*LD)*(BD-LD/3)*1e4)
Region 2 from 1.942 to 1.797 ppm
Diff.Con.=2.498E-9m2/S
[G/cm]
[*1e9]

D TMS

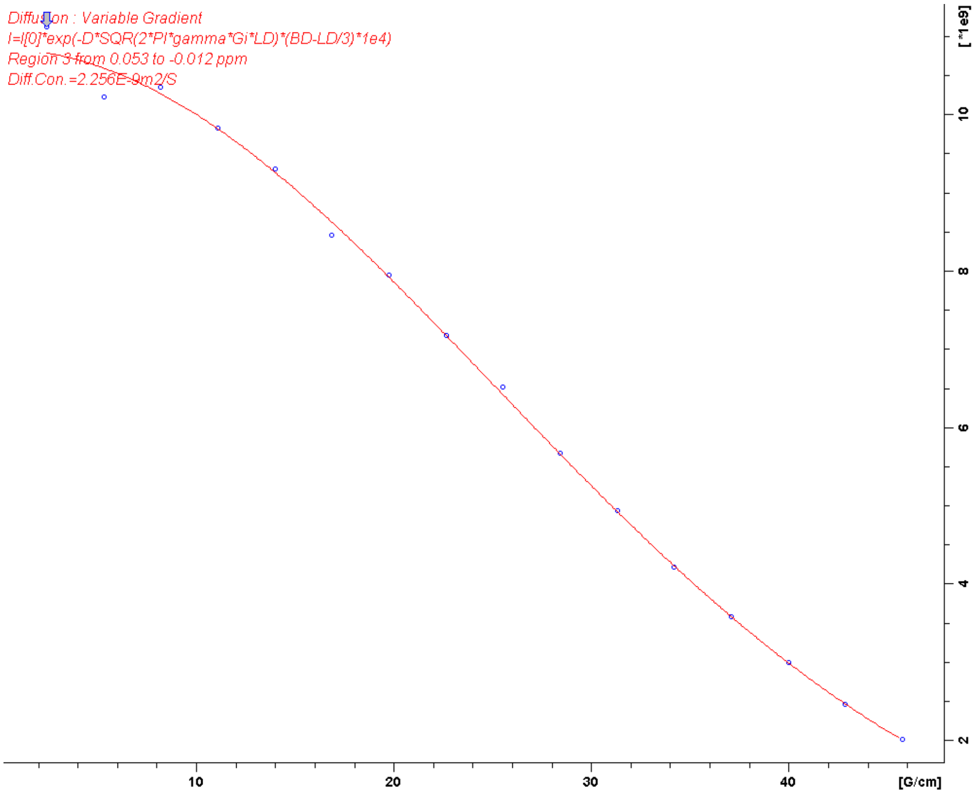
Diffusion : Variable Gradient
I=I[0]*exp(-D*SQR(2*PI*gamma*Gi*LD)*(BD-LD/3)*1e4)
Region 3 from 0.053 to -0.012 ppm
Diff.Con.=2.256E-9m2/S
[G/cm]
[*1e9]

5.1.13 DOSY THF/mCPBA/TMS

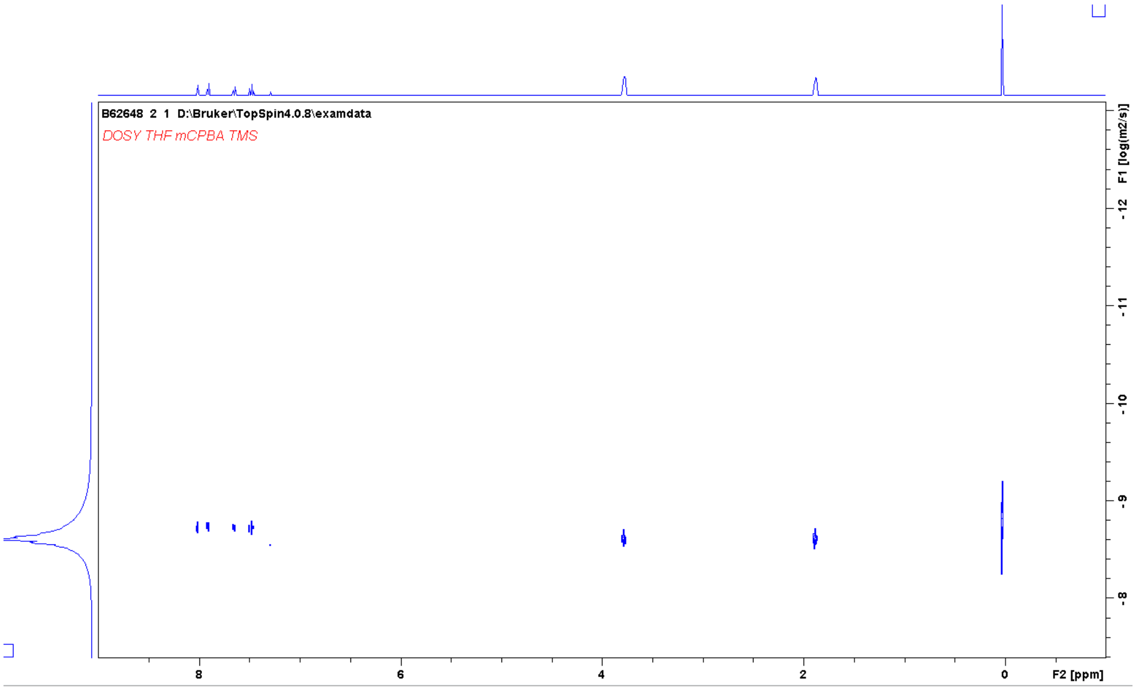

D *m*CPBA

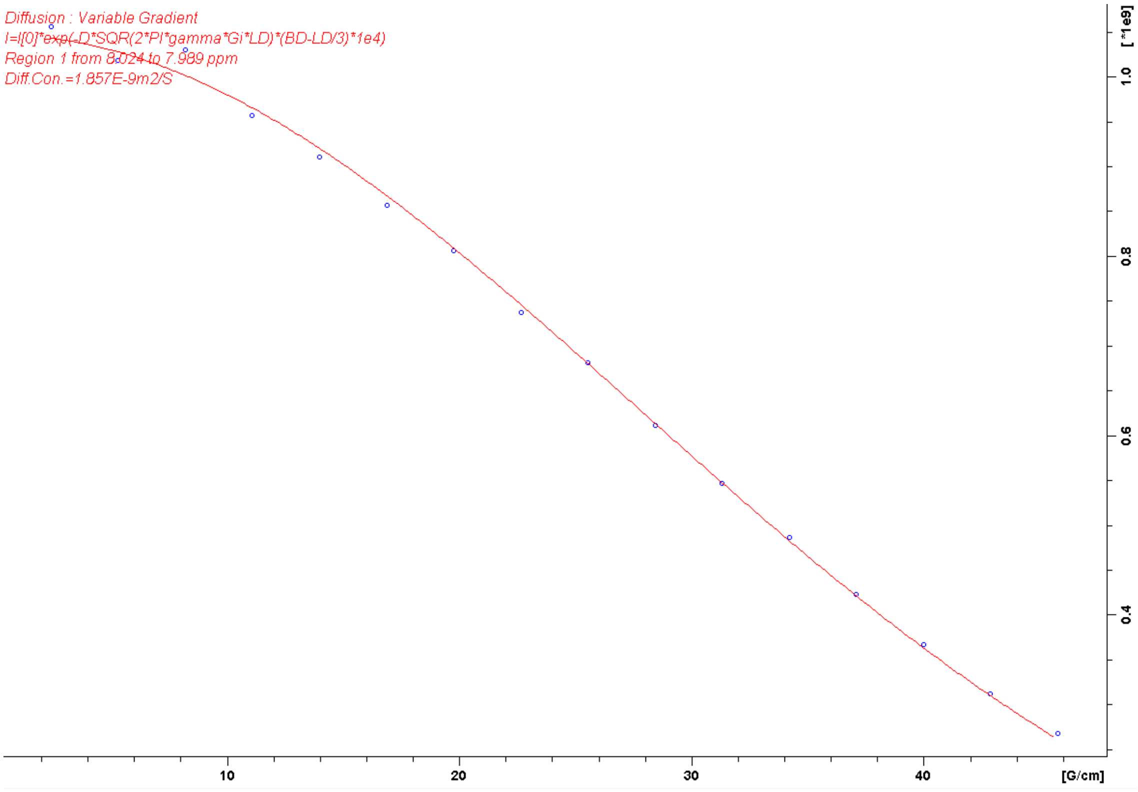

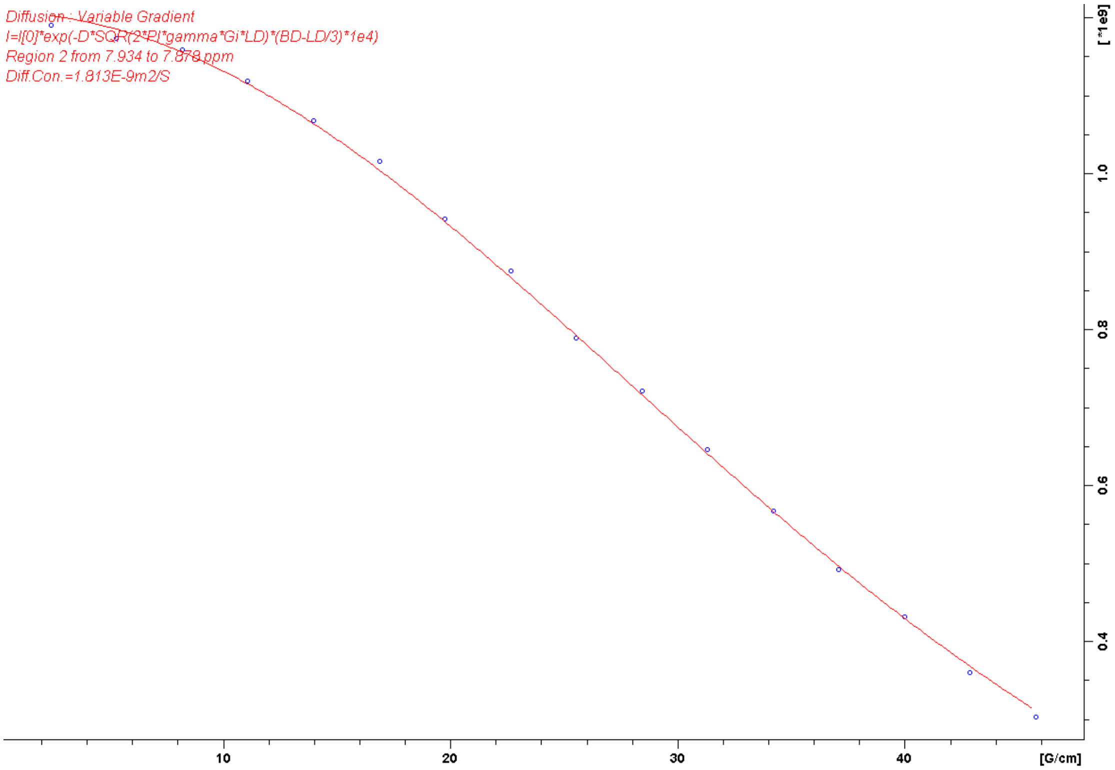
Diffusion : Variable Gradient
I=I[0]*exp(-D*SQR(2*PI*gamma*Gi*LD)*(BD-LD/3)*1e4)
Region 2 from 7.934 to 7.878 ppm
Diff.Con.=1.813E-9m2/S
[*1e9]
1.0
0.8
0.6
0.4
10
20
30
40
[G/cm]

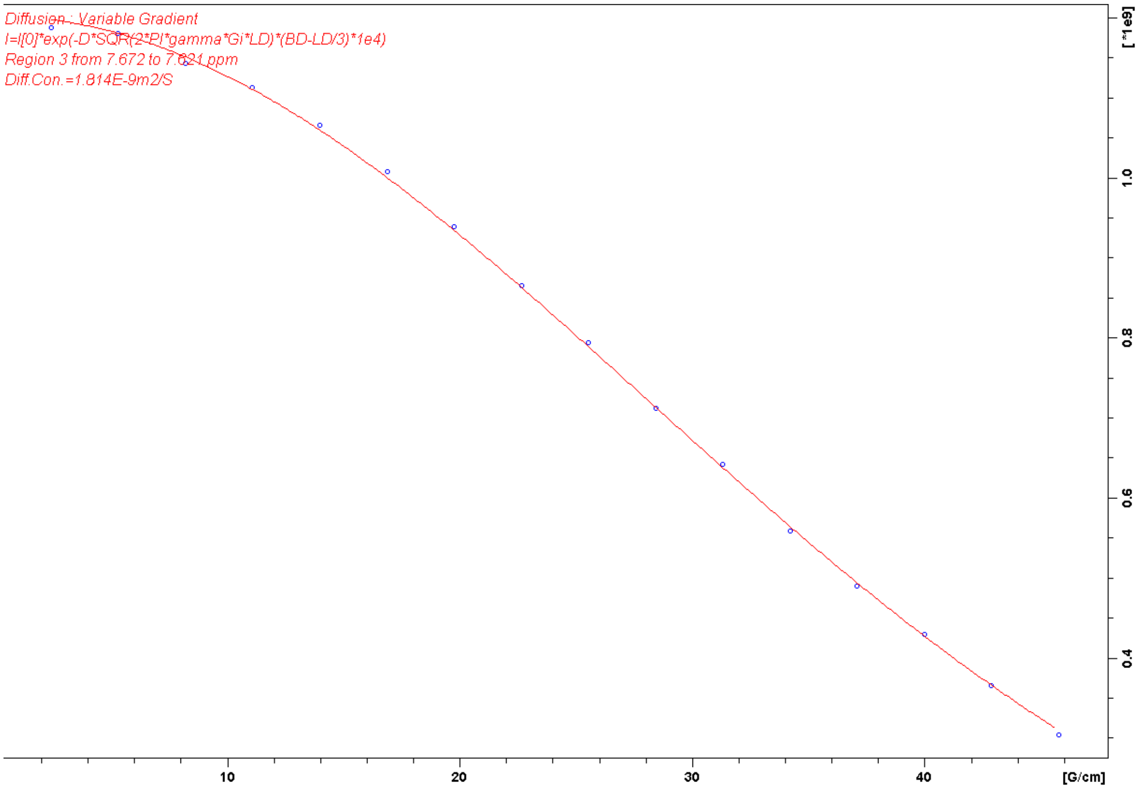
Diffusion : Variable Gradient
I=I[0]*exp(-D*SQR(2*PI*gamma*Gi*LD)*(BD-LD/3)*1e4)
Region 3 from 7.672 to 7.621 ppm
Diff.Con.=1.814E-9m2/S
[*1e9]
1.0
0.8
0.6
0.4
10
20
30
40
[G/cm]

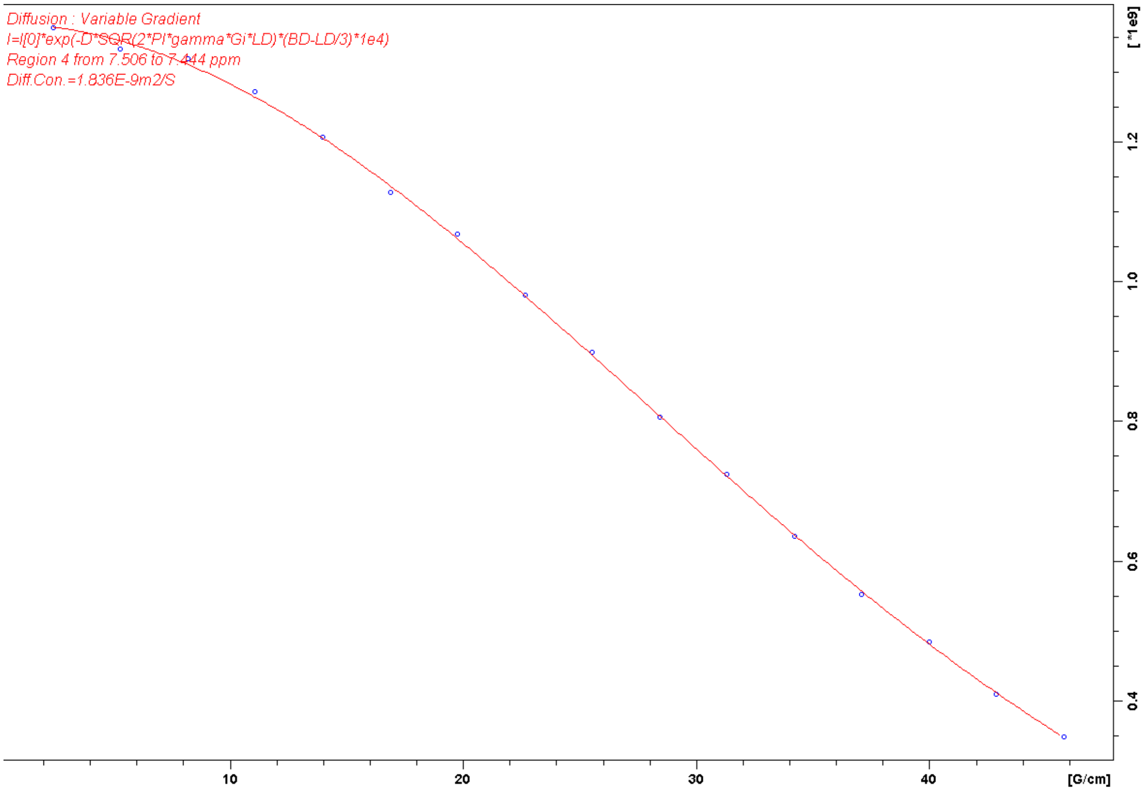
Diffusion : Variable Gradient
I=I[0]*exp(-D*SQR(2*PI*gamma*Gi*LD)*(BD-LD/3)*1e4)
Region 4 from 7.506 to 7.444 ppm
Diff.Con.=1.836E-9m2/S
[*1e9]
1.2
1.0
0.8
0.6
0.4
10
20
30
40
[G/cm]

D THF

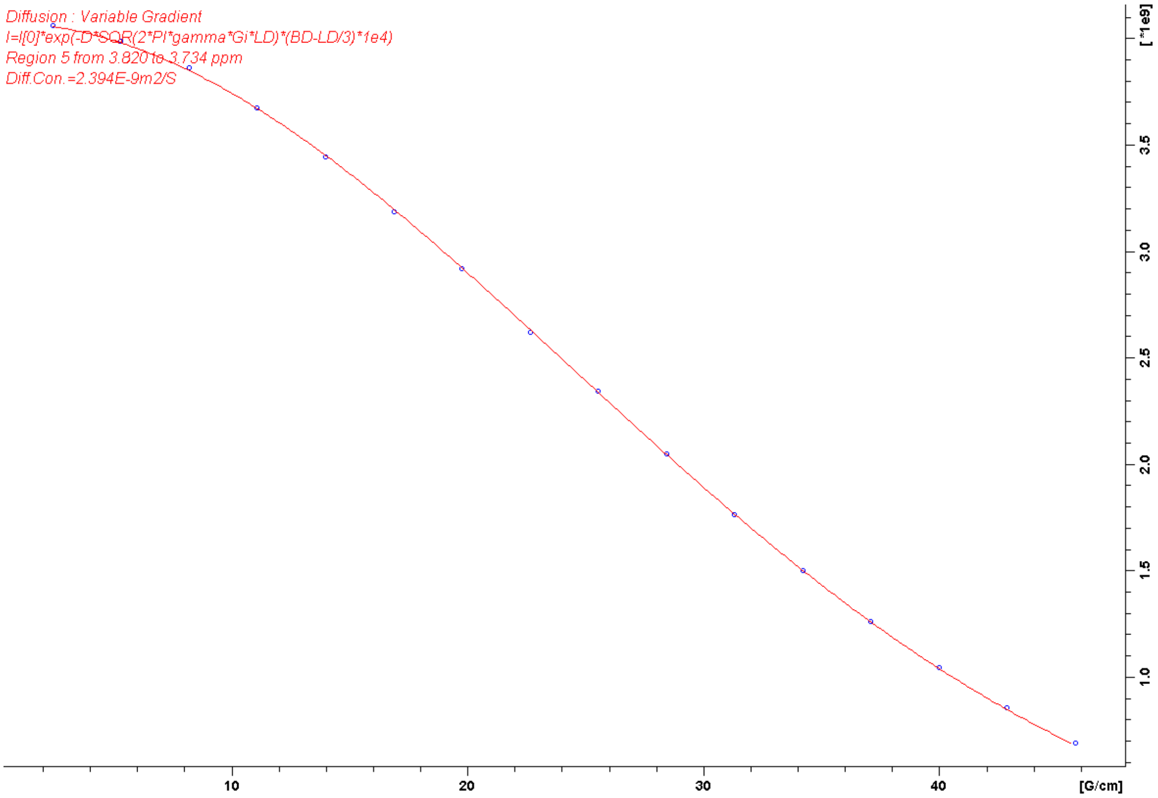
Diffusion : Variable Gradient
I=I[0]*exp(-D*SQR(2*PI*gamma*Gi*LD)*(BD-LD/3)*1e4)
Region 5 from 3.820 to 3.734 ppm
Diff.Con.=2.394E-9m2/S
[*1e9]
3.5
3.0
2.5
2.0
1.5
1.0
10
20
30
40
[G/cm]

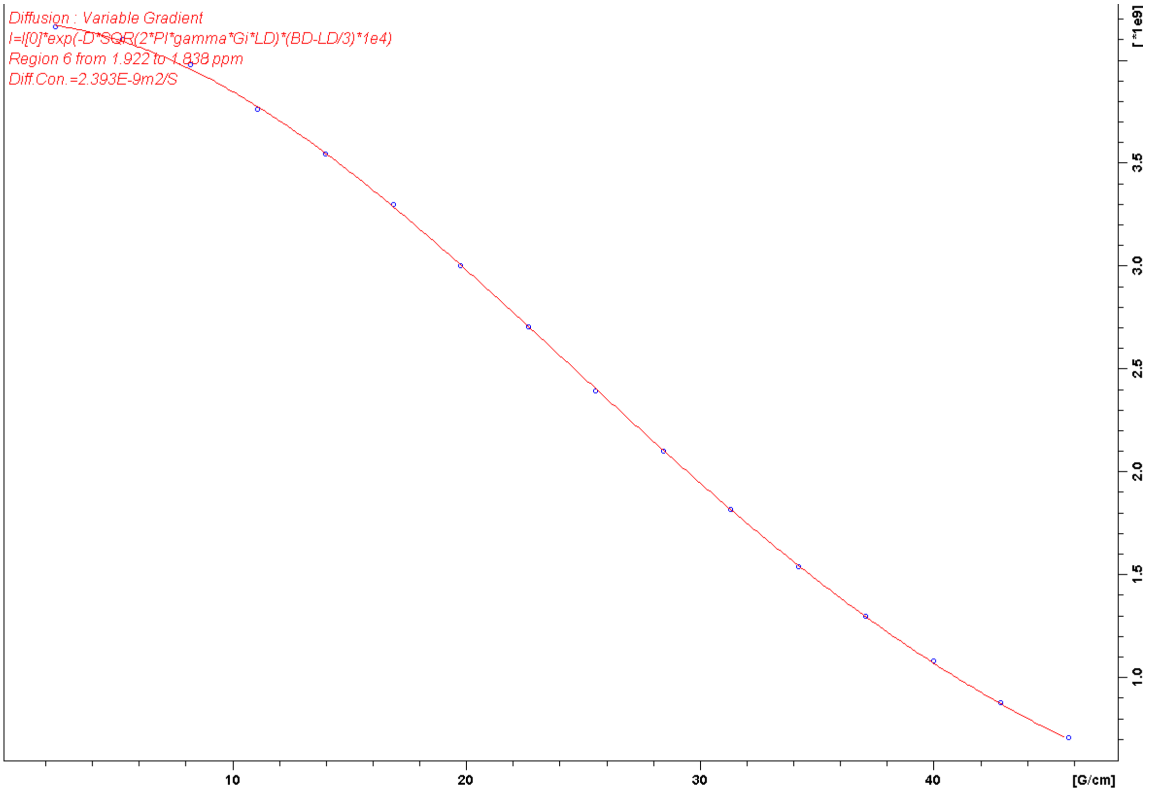
Diffusion : Variable Gradient
I=I[0]*exp(-D*SQR(2*PI*gamma*Gi*LD)*(BD-LD/3)*1e4)
Region 6 from 1.922 to 1.838 ppm
Diff.Con.=2.393E-9m2/S
[*1e9]
3.5
3.0
2.5
2.0
1.5
1.0
10
20
30
40
[G/cm]

D TMS

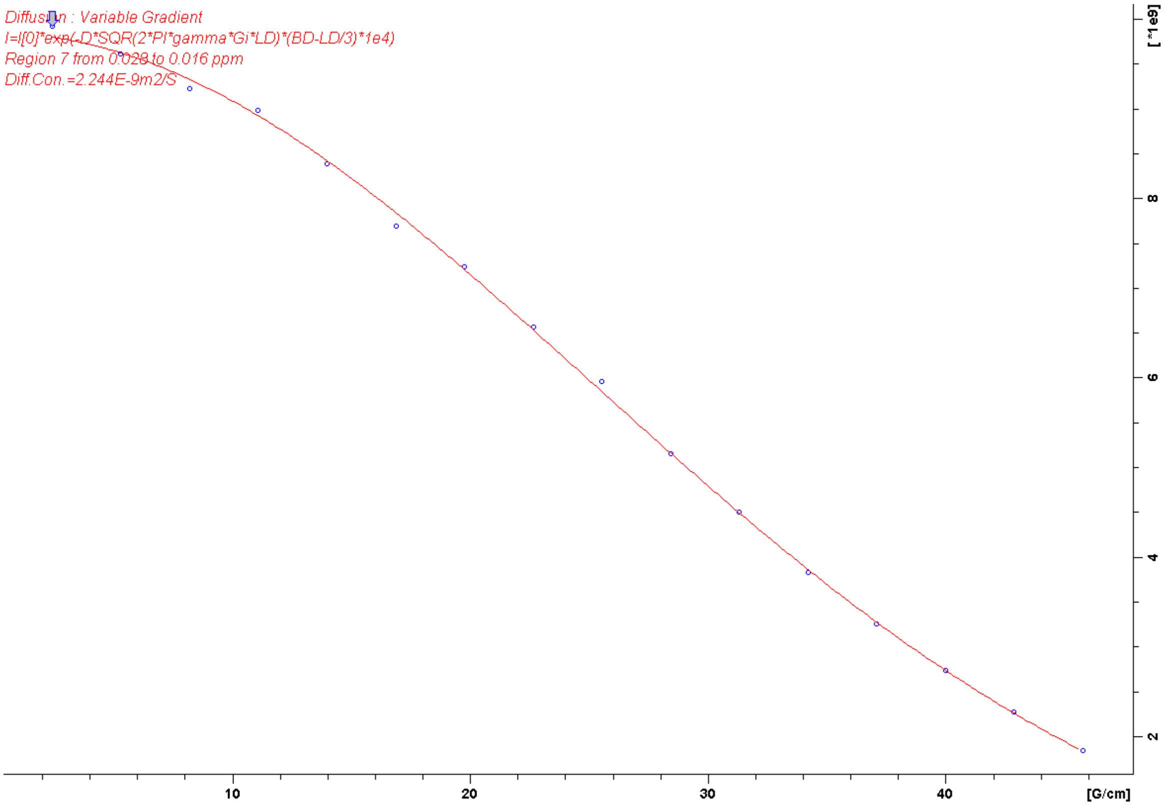
Diffusion : Variable Gradient
I=I[0]*exp(-D*SQR(2*PI*gamma*Gi*LD)*(BD-LD/3)*1e4)
Region 7 from 0.028 to 0.016 ppm
Diff.Con.=2.244E-9m2/S
[*1e9]
8
6
4
2
10
20
30
40
[G/cm]

5.1.14 DOSY mCPBA/THF/1 eq NFTB/TMS

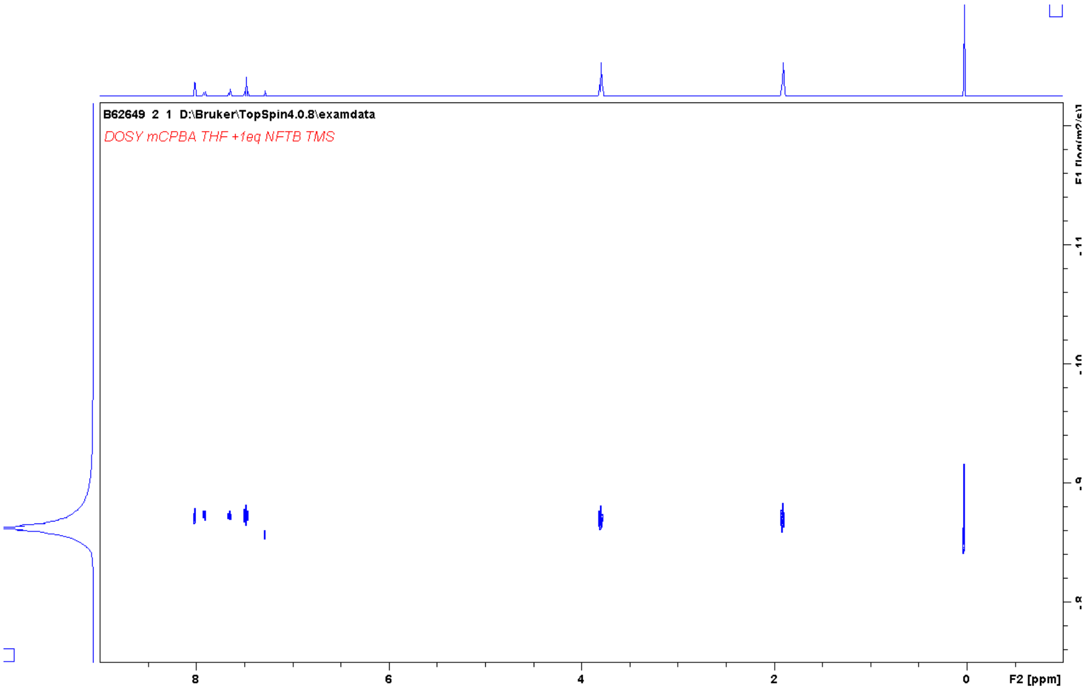

D *m*CPBA

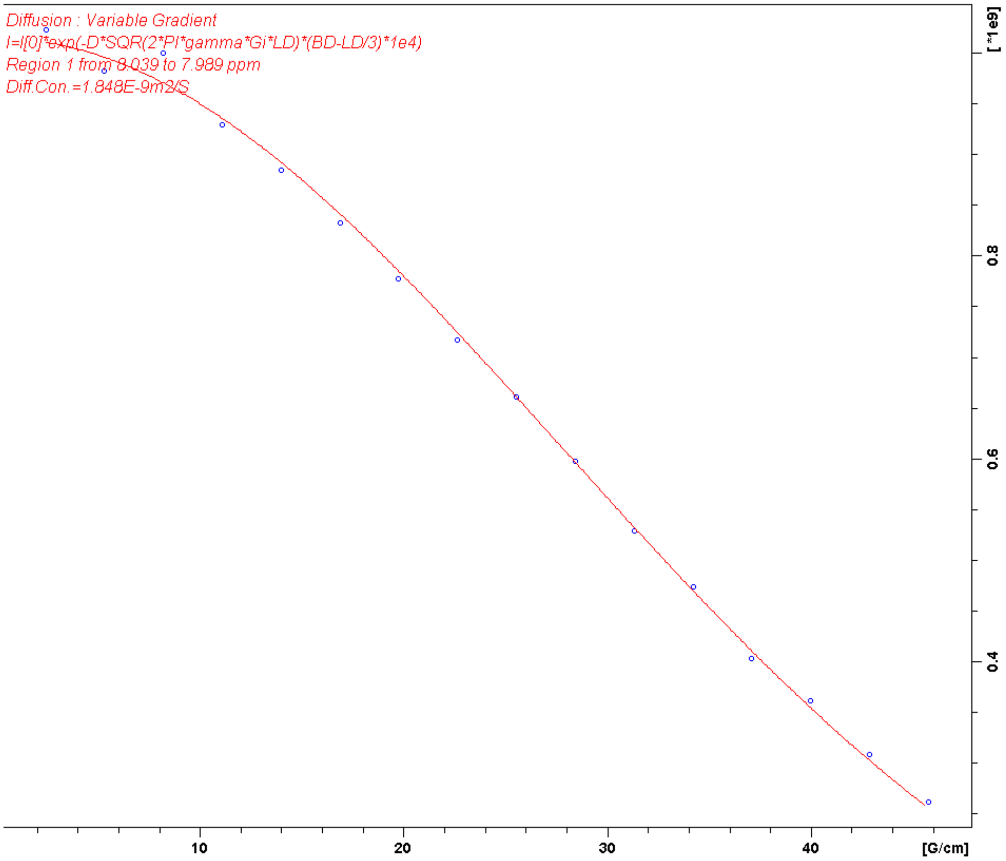

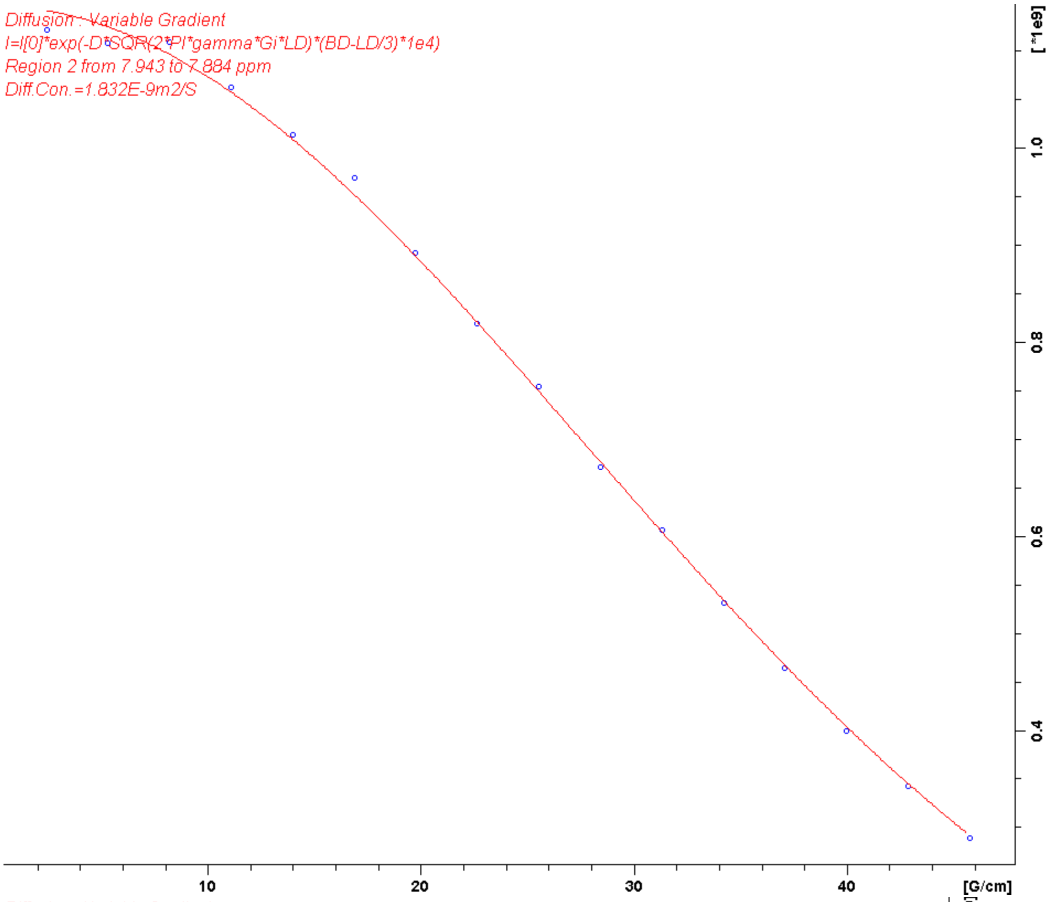
Diffusion : Variable Gradient
I=I[0]*exp(-D*SQR(2*PI*gamma*Gi*LD)*(BD-LD/3)*1e4)
Region 2 from 7.943 to 7.884 ppm
Diff.Con.=1.832E-9m2/S
[*1e9]
1.0
0.8
0.6
0.4
10
20
30
40
[G/cm]

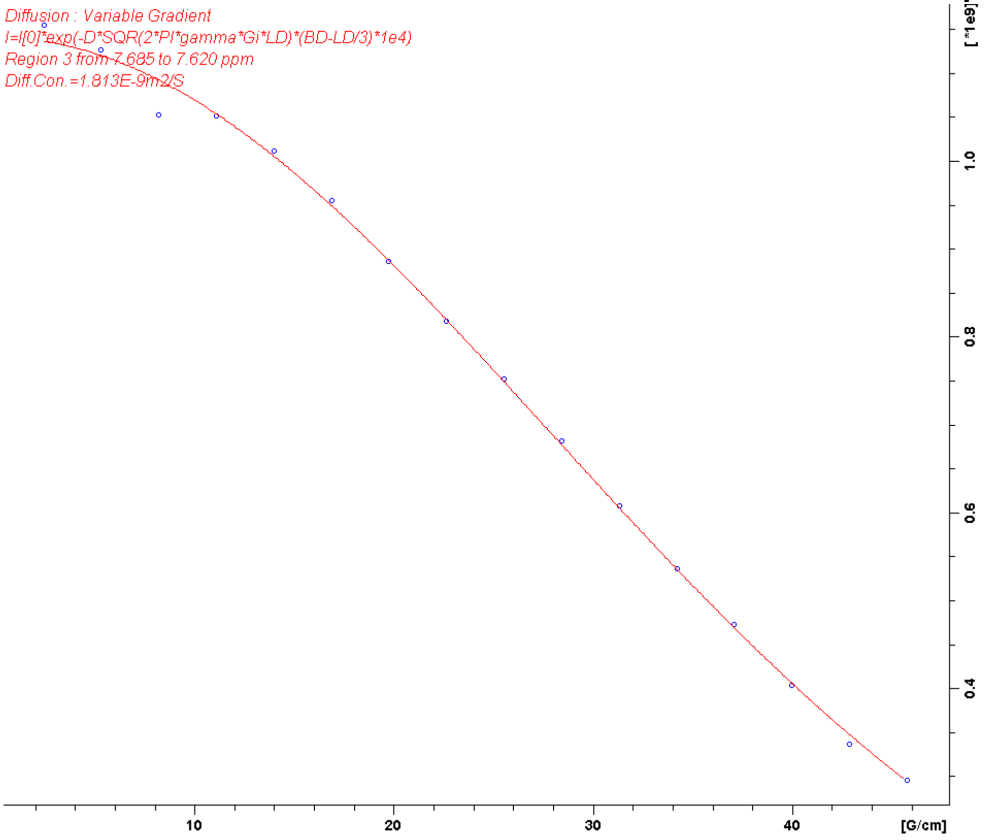
Diffusion : Variable Gradient
I=I[0]*exp(-D*SQR(2*PI*gamma*Gi*LD)*(BD-LD/3)*1e4)
Region 3 from 7.685 to 7.620 ppm
Diff.Con.=1.813E-9m2/S
[*1e9]
1.0
0.8
0.6
0.4
10
20
30
40
[G/cm]

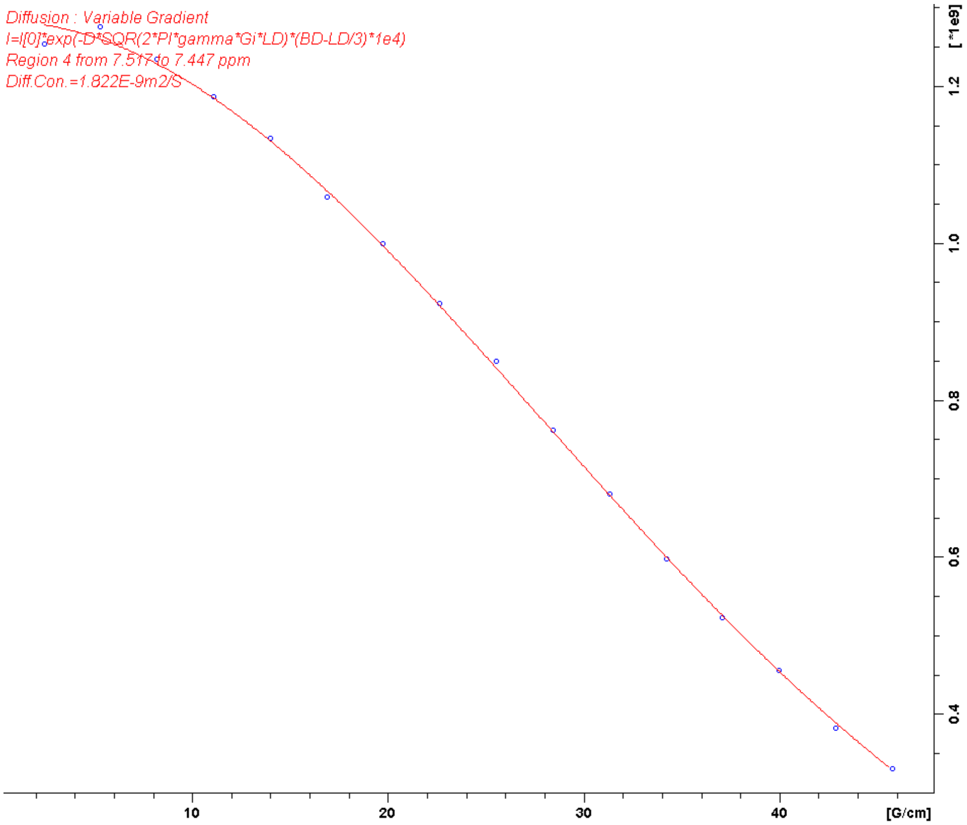
Diffusion : Variable Gradient
I=I[0]*exp(-D*SQR(2*PI*gamma*Gi*LD)*(BD-LD/3)*1e4)
Region 4 from 7.517 to 7.447 ppm
Diff.Con.=1.822E-9m2/S
[*1e9]
1.2
1.0
0.8
0.6
0.4
10
20
30
40
[G/cm]

D THF

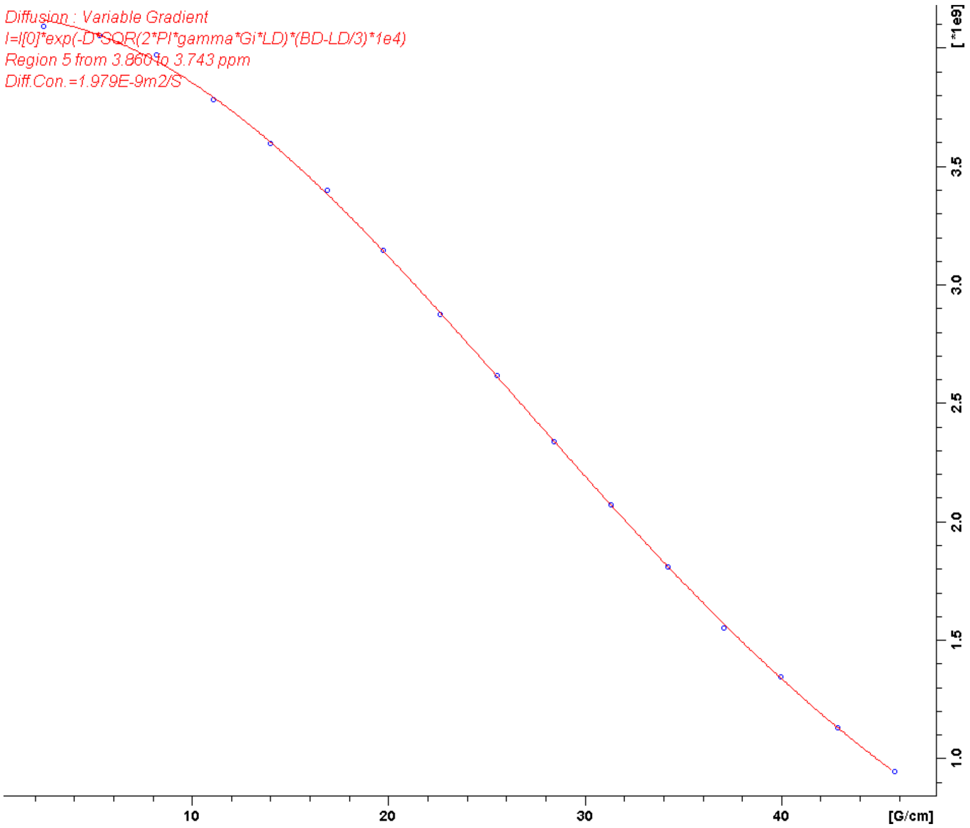
Diffusion : Variable Gradient
I=I[0]*exp(-D*SQR(2*PI*gamma*Gi*LD)*(BD-LD/3)*1e4)
Region 5 from 3.860 to 3.743 ppm
Diff.Con.=1.979E-9m2/S
[*1e9]
3.5
3.0
2.5
2.0
1.5
1.0
10
20
30
40
[G/cm]

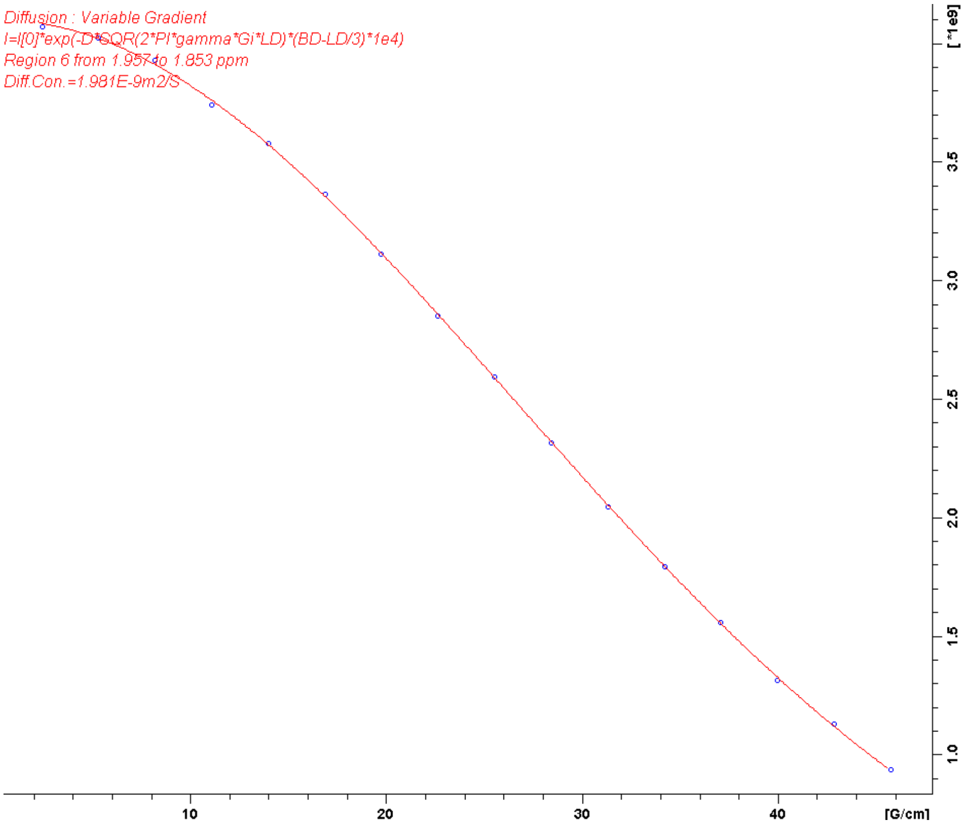
Diffusion : Variable Gradient
I=I[0]*exp(-D*SQR(2*PI*gamma*Gi*LD)*(BD-LD/3)*1e4)
Region 6 from 1.957 to 1.853 ppm
Diff.Con.=1.981E-9m2/S
[*1e9]
[G/cm]

D TMS

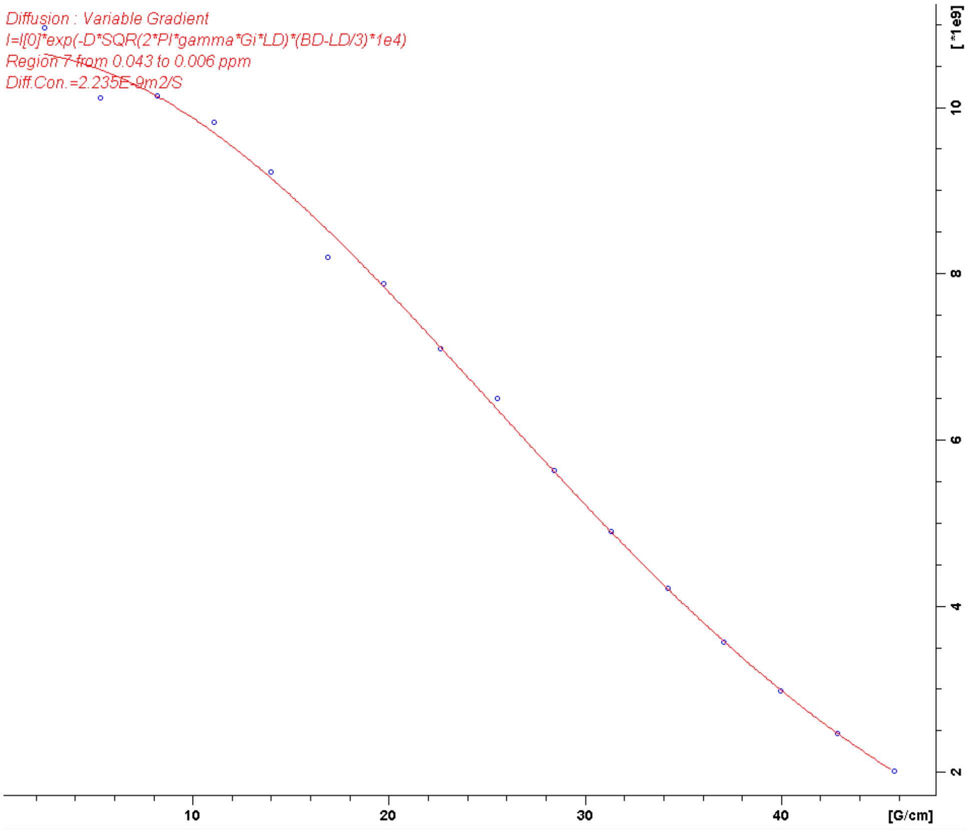
Diffusion : Variable Gradient
I=I[0]*exp(-D*SQR(2*PI*gamma*Gi*LD)*(BD-LD/3)*1e4)
Region 7 from 0.043 to 0.006 ppm
Diff.Con.=2.235E-9m2/S
[*1e9]
[G/cm]

5.1.15 DOSY *m*CPBA/THF/+2 eq NFTB/TMS

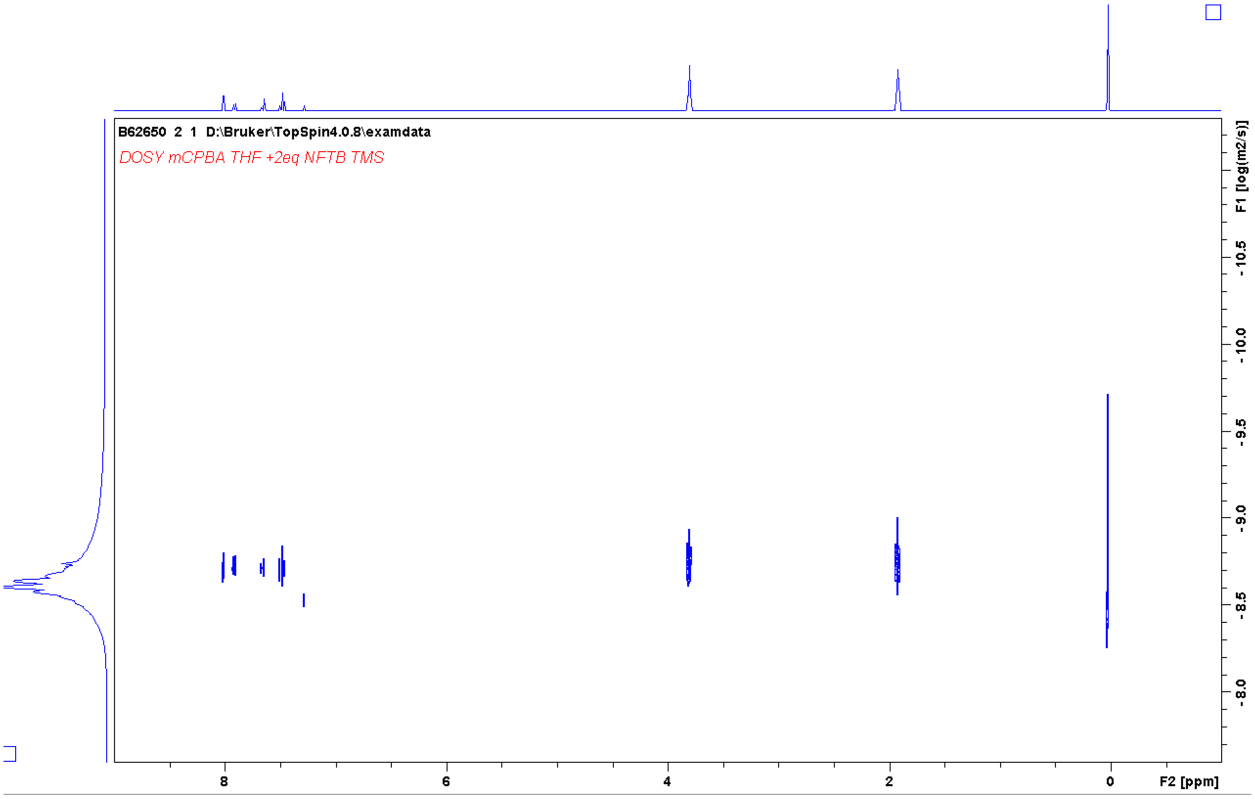

D *m*CPBA

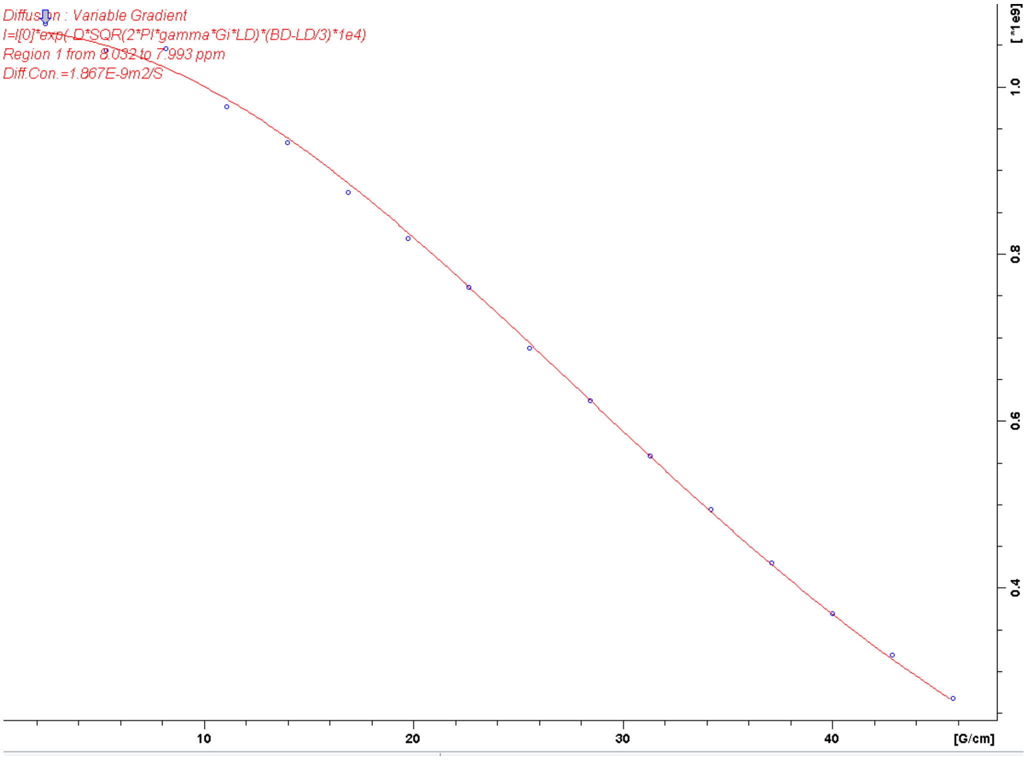

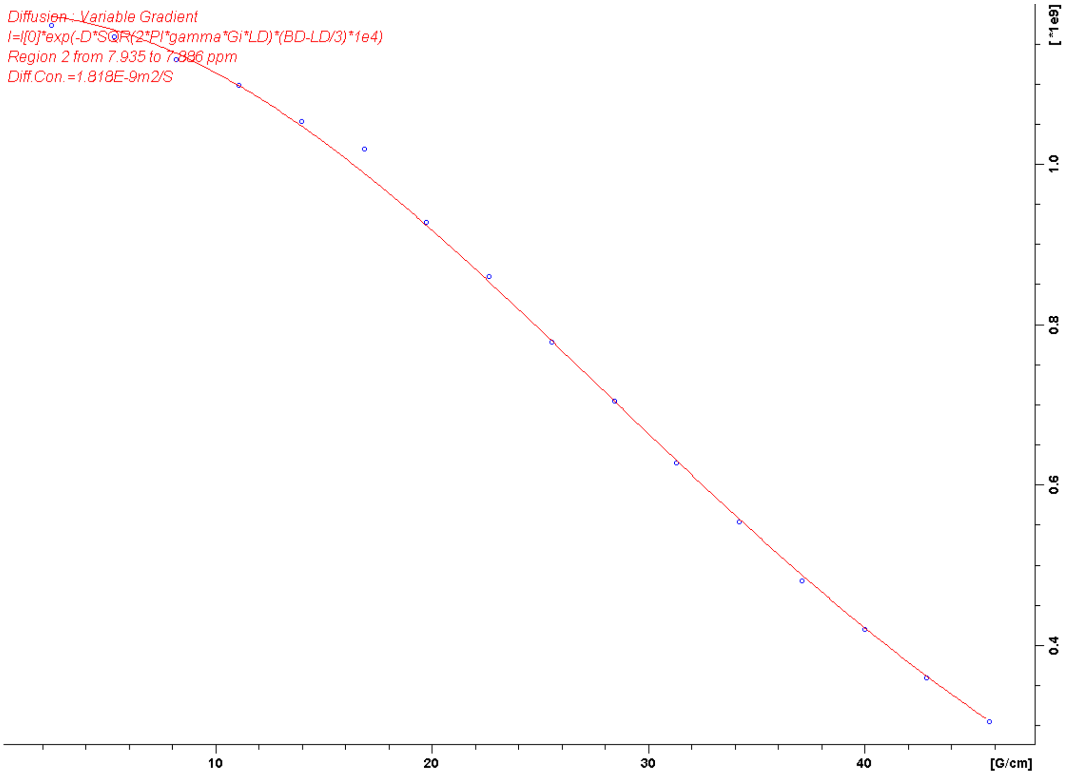
Diffusion : Variable Gradient
I=I[0]*exp(-D*SQR(2*PI*gamma*Gi*LD)*(BD-LD/3)*1e4)
Region 2 from 7.935 to 7.886 ppm
Diff.Con.=1.818E-9m2/S
[*1e9]
1.0
0.8
0.6
0.4
10
20
30
40
[G/cm]

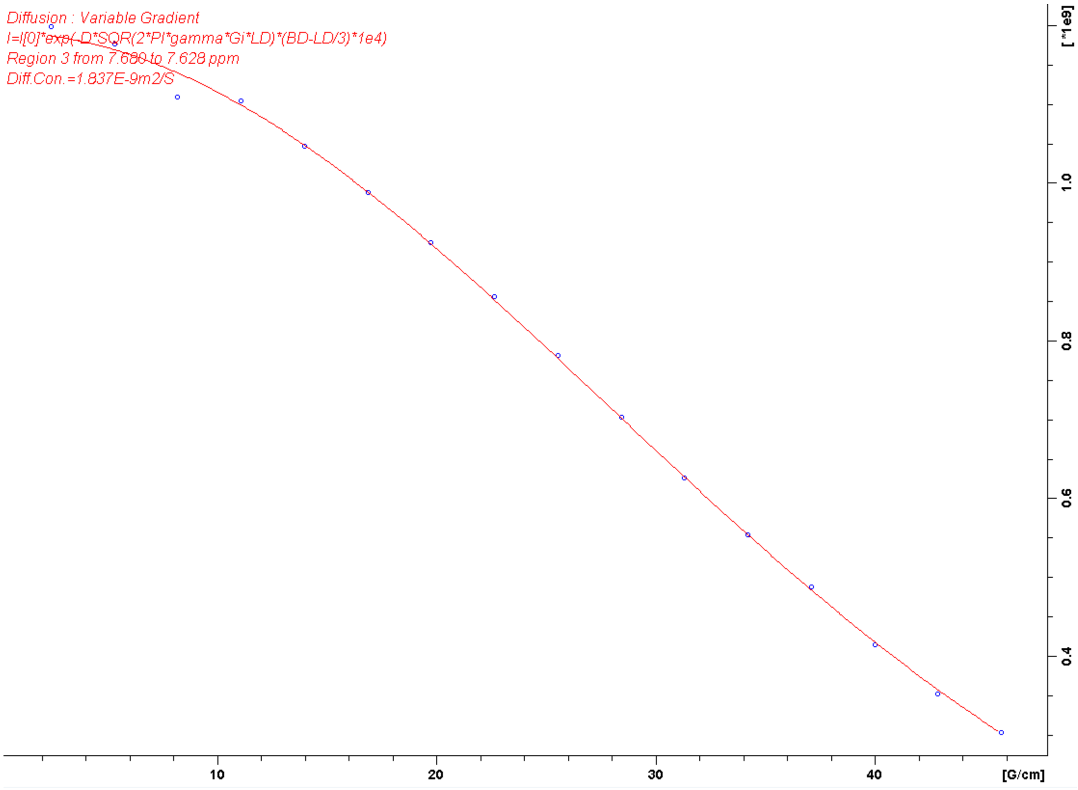
Diffusion : Variable Gradient
I=I[0]*exp(-D*SQR(2*PI*gamma*Gi*LD)*(BD-LD/3)*1e4)
Region 3 from 7.680 to 7.628 ppm
Diff.Con.=1.837E-9m2/S
[*1e9]
1.0
0.8
0.6
0.4
10
20
30
40
[G/cm]

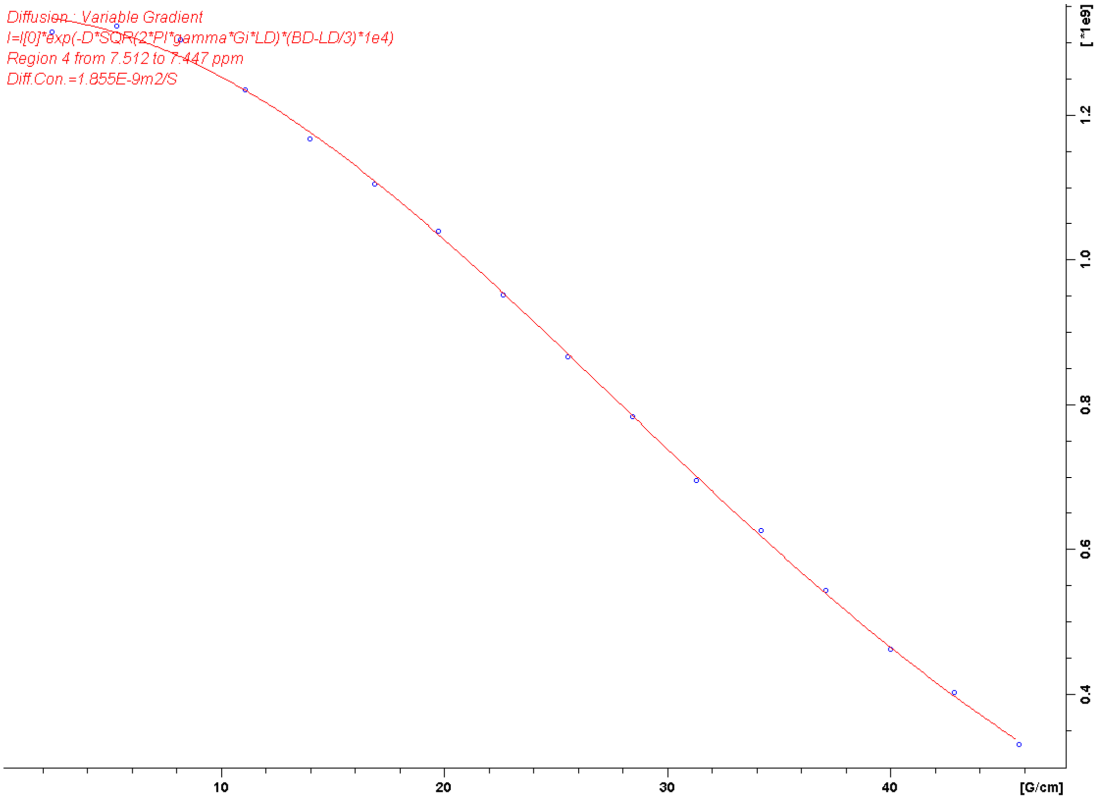
Diffusion : Variable Gradient
I=I[0]*exp(-D*SQR(2*PI*gamma*Gi*LD)*(BD-LD/3)*1e4)
Region 4 from 7.512 to 7.447 ppm
Diff.Con.=1.855E-9m2/S
[*1e9]
1.2
1.0
0.8
0.6
0.4
10
20
30
40
[G/cm]

D THF

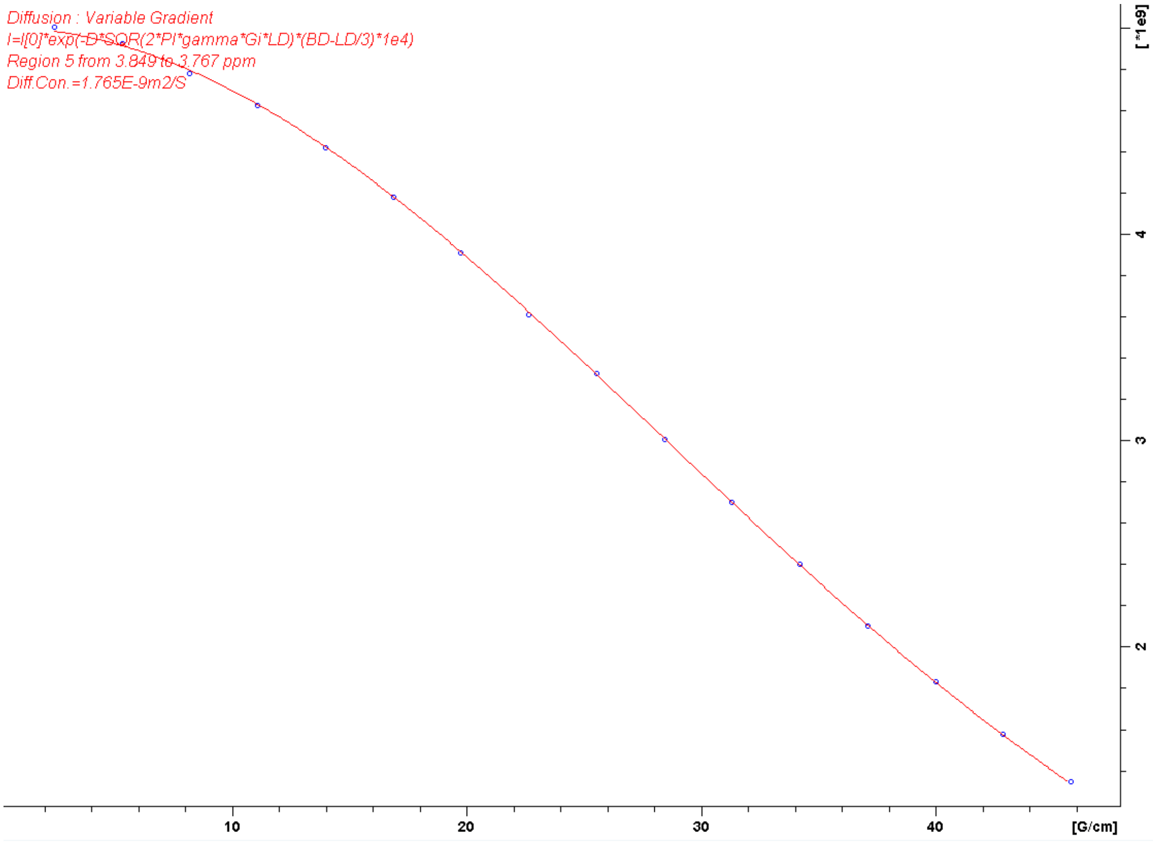
Diffusion : Variable Gradient
I=I[0]*exp(-D*SQR(2*PI*gamma*Gi*LD)*(BD-LD/3)*1e4)
Region 5 from 3.849 to 3.767 ppm
Diff.Con.=1.765E-9m2/S
[*1e9]
4
3
2
10
20
30
40
[G/cm]

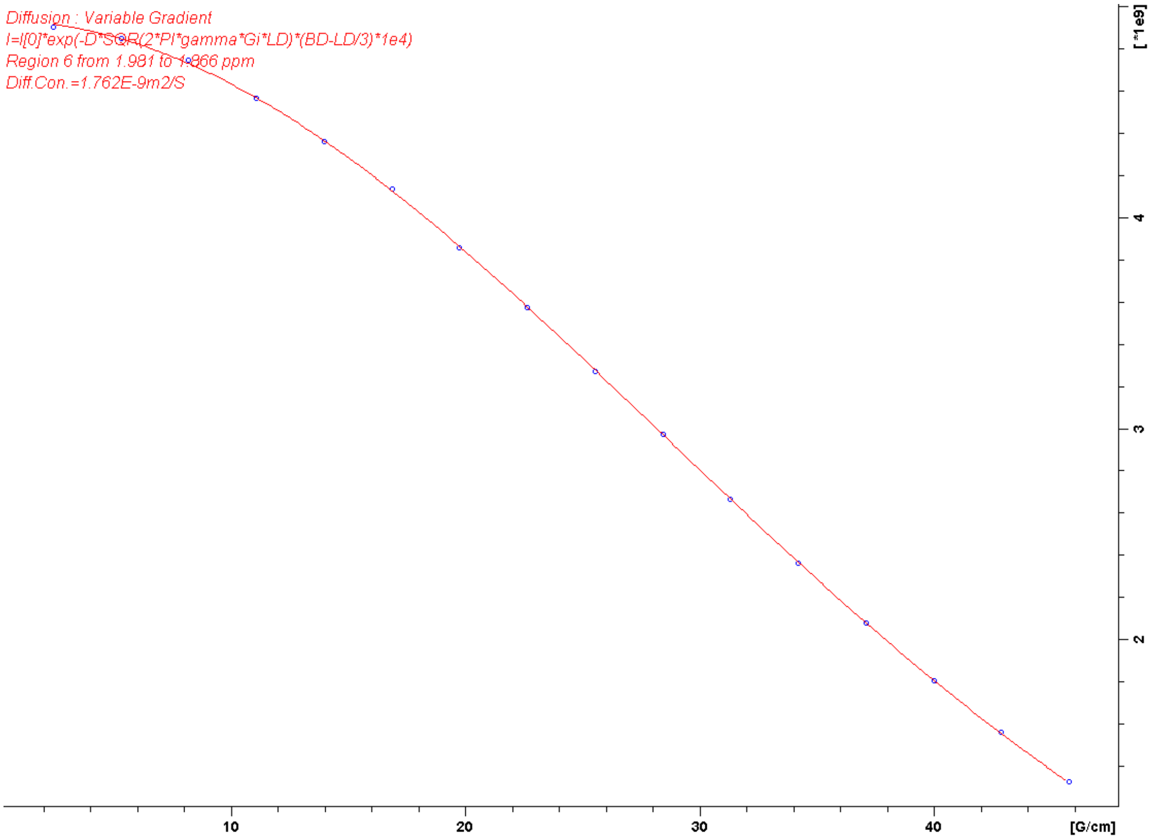

Diffusion : Variable Gradient
I=I[0]*exp(-D*SQR(2*PI*gamma*Gi*LD)*(BD-LD/3)*1e4)
Region 6 from 1.981 to 1.866 ppm
Diff.Con.=1.762E-9m2/S
[*1e9]
4
3
2
10
20
30
40
[G/cm]

D TMS

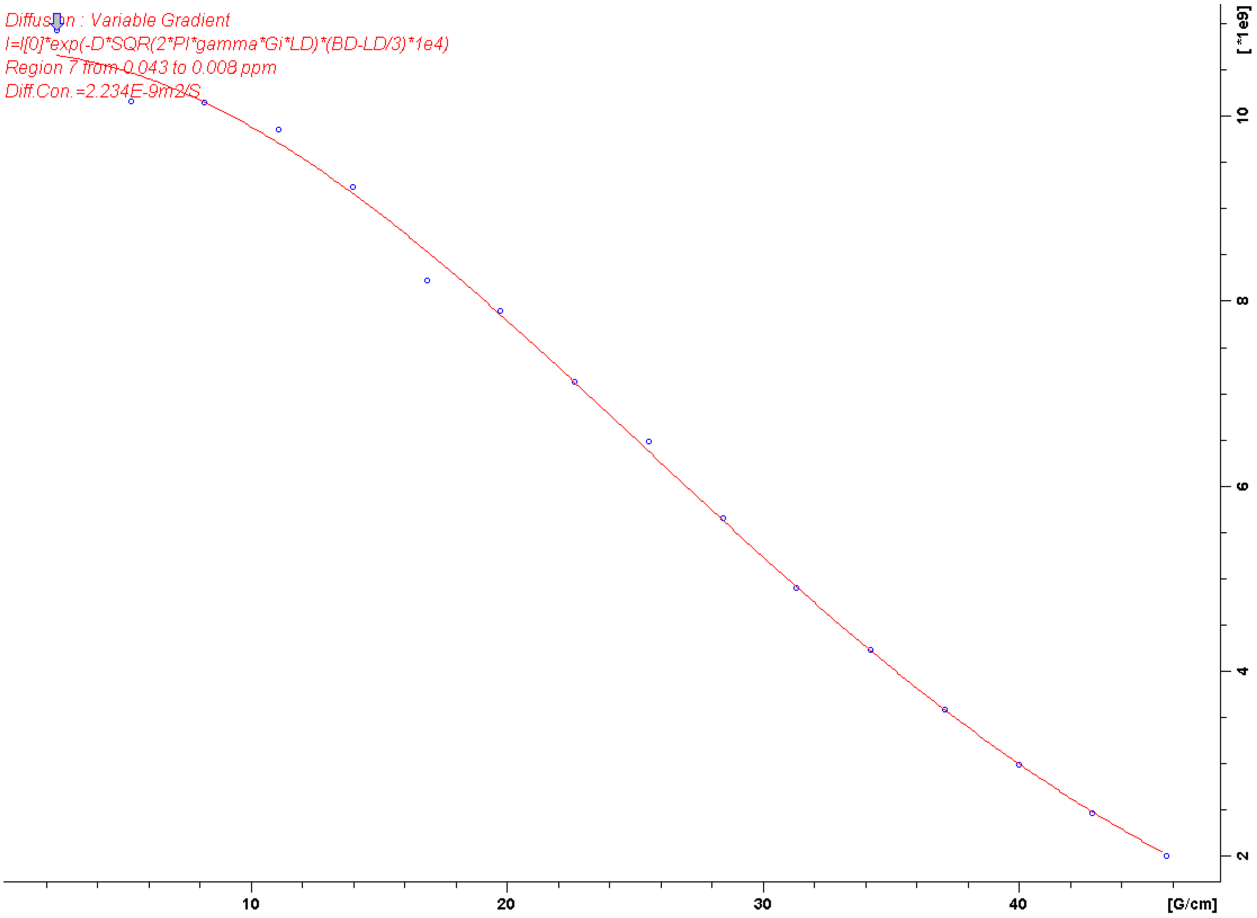

Diffusion : Variable Gradient
I=I[0]*exp(-D*SQR(2*PI*gamma*Gi*LD)*(BD-LD/3)*1e4)
Region 7 from 0.043 to 0.008 ppm
Diff.Con.=2.234E-9m2/S
[*1e9]
10
8
6
4
2
10
20
30
40
[G/cm]

5.1.16 DOSY *m*CPBA/THF/+3 eq/NFTB/TMS

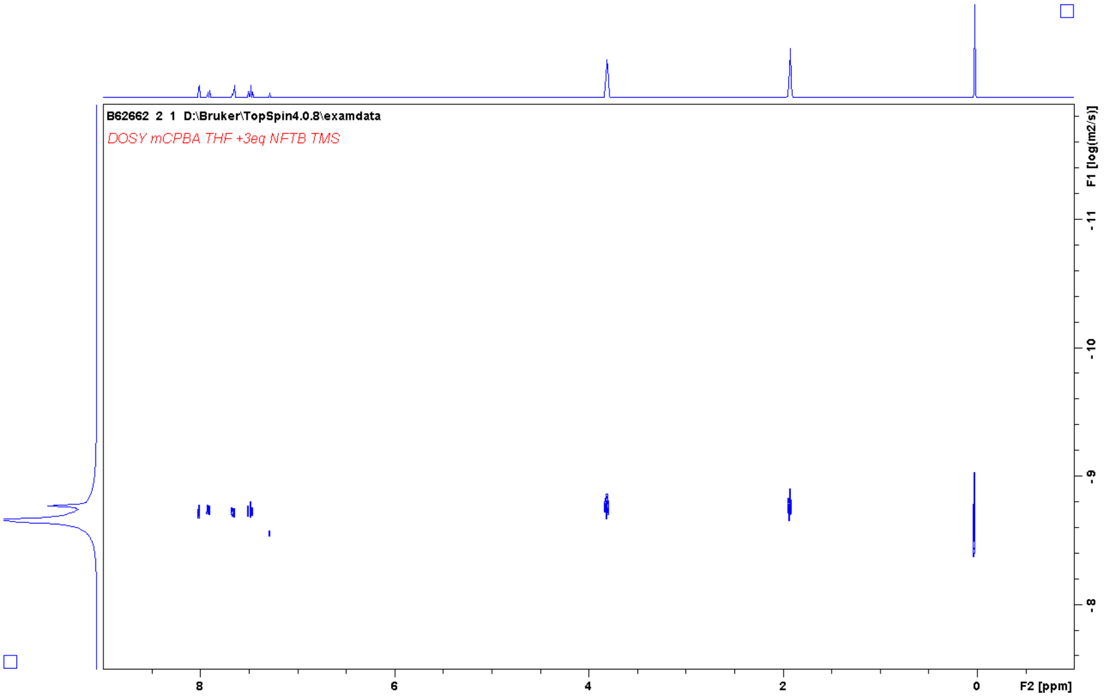

D *m*CPBA

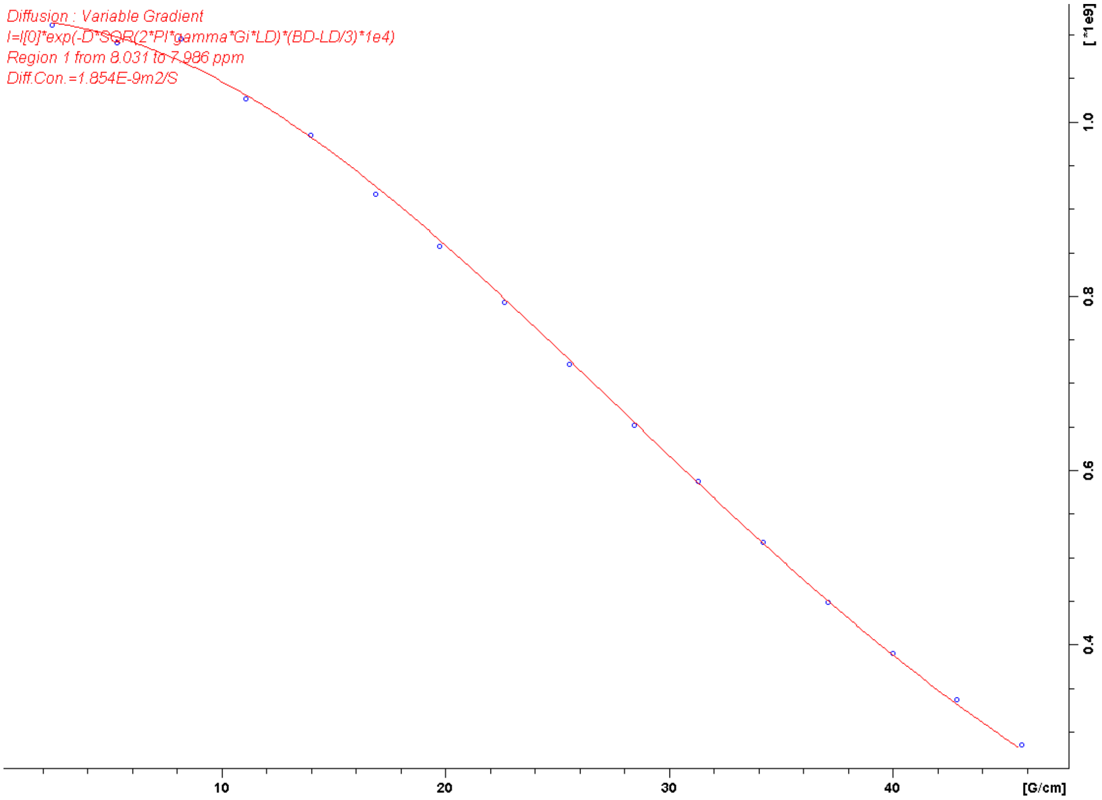

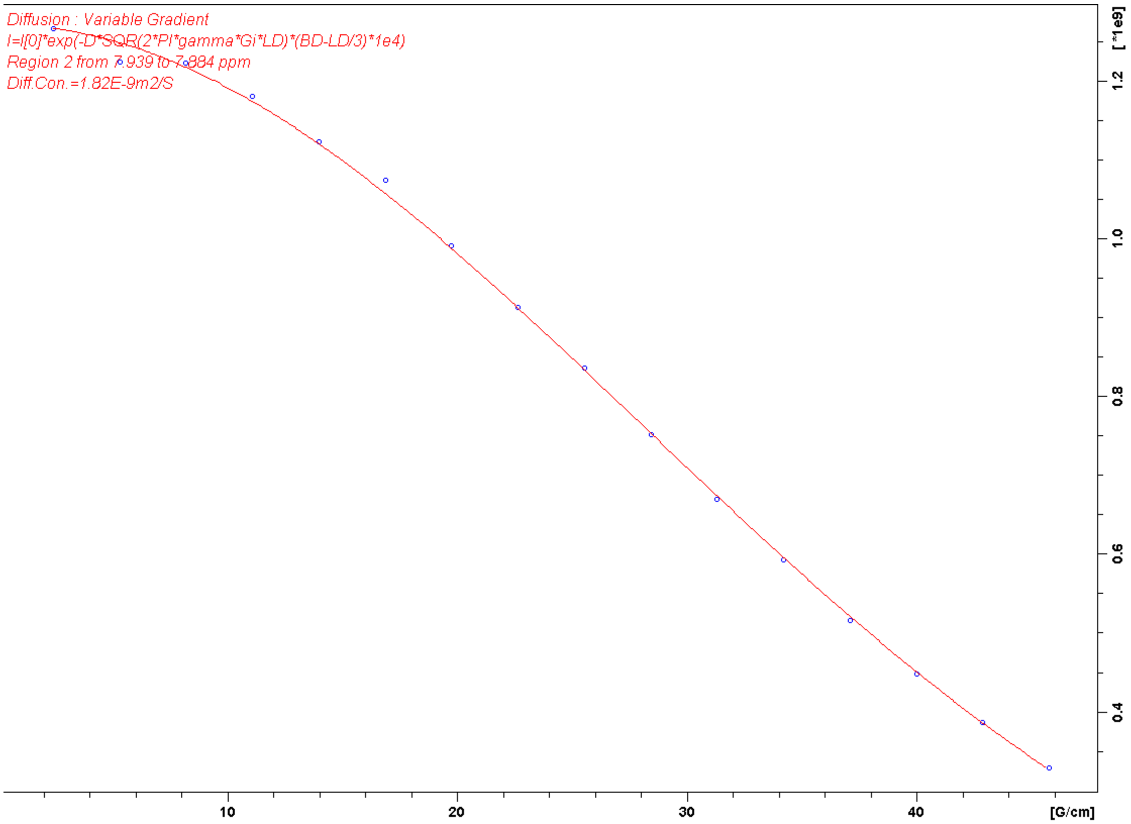
Diffusion : Variable Gradient
I=I[0]*exp(-D*SQR(2*PI*gamma*Gi*LD)*(BD-LD/3)*1e4)
Region 2 from 7.939 to 7.884 ppm
Diff.Con.=1.82E-9m2/S
[*1e9]
1.2
1.0
0.8
0.6
0.4
10
20
30
40
[G/cm]

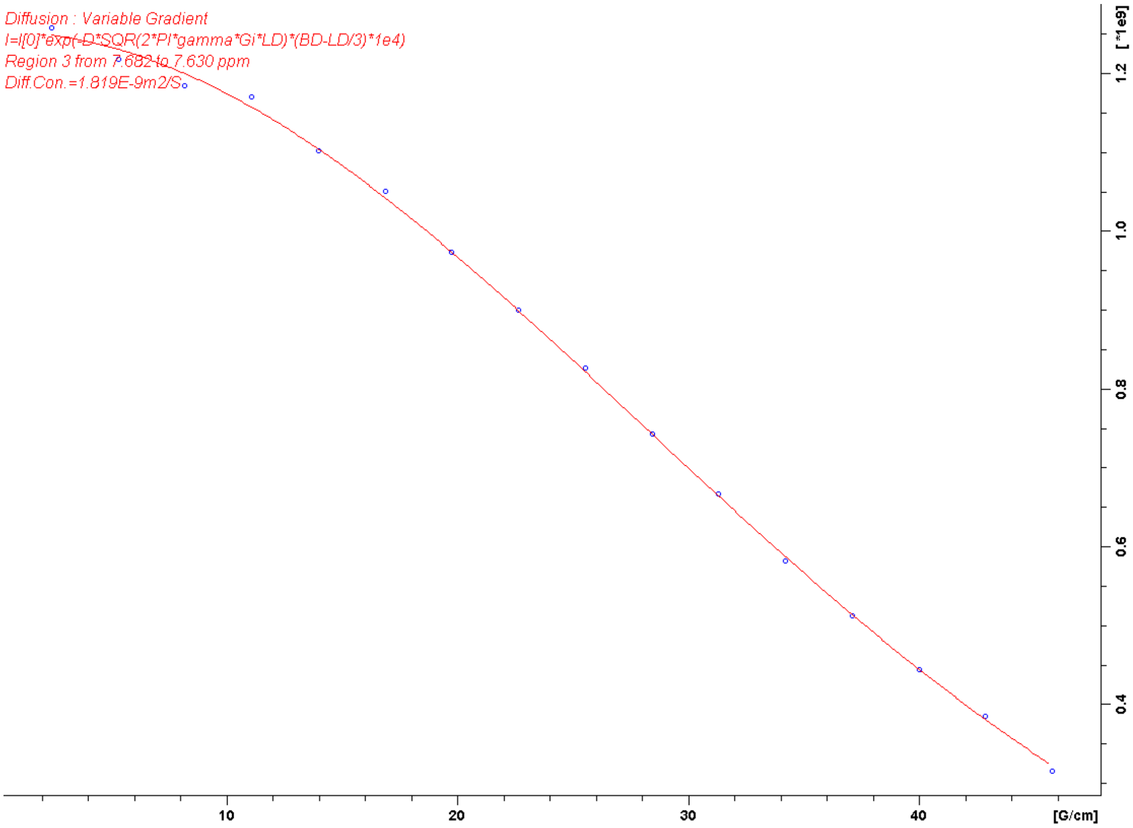
Diffusion : Variable Gradient
I=I[0]*exp(-D*SQR(2*PI*gamma*Gi*LD)*(BD-LD/3)*1e4)
Region 3 from 7.682 to 7.630 ppm
Diff.Con.=1.819E-9m2/S
[*1e9]
1.2
1.0
0.8
0.6
0.4
10
20
30
40
[G/cm]

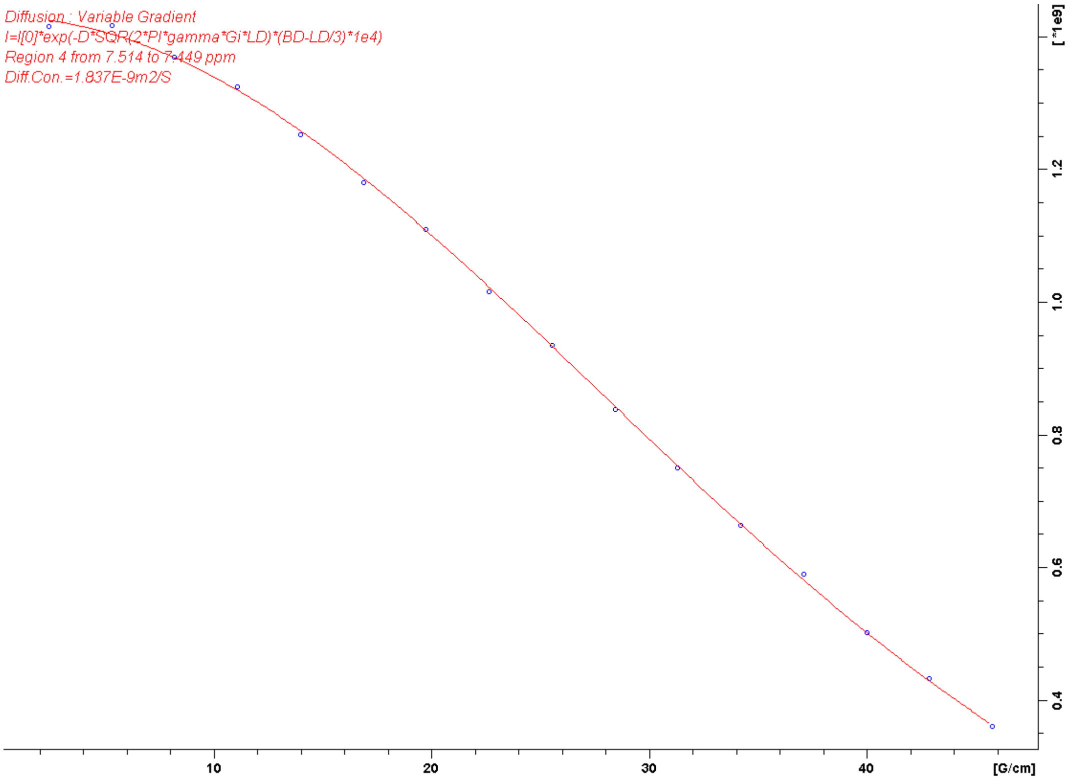

Diffusion : Variable Gradient
I=I[0]*exp(-D*SQR(2*PI*gamma*Gi*LD)*(BD-LD/3)*1e4)
Region 4 from 7.514 to 7.449 ppm
Diff.Con.=1.837E-9m2/S
[*1e9]
1.2
1.0
0.8
0.6
0.4
10
20
30
40
[G/cm]

D THF

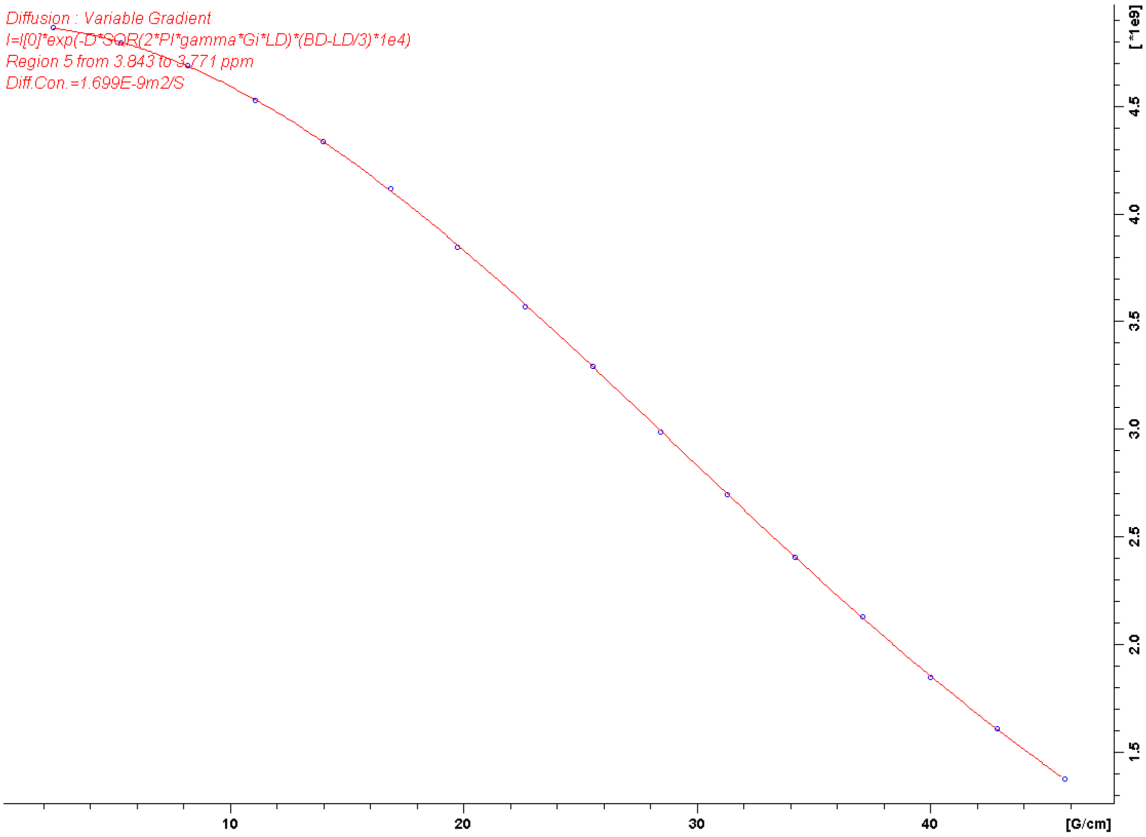

Diffusion : Variable Gradient
I=I[0]*exp(-D*SQR(2*PI*gamma*Gi*LD)*(BD-LD/3)*1e4)
Region 5 from 3.843 to 3.771 ppm
Diff.Con.=1.699E-9m2/S
[*1e9]
4.5
4.0
3.5
3.0
2.5
2.0
1.5
10
20
30
40
[G/cm]

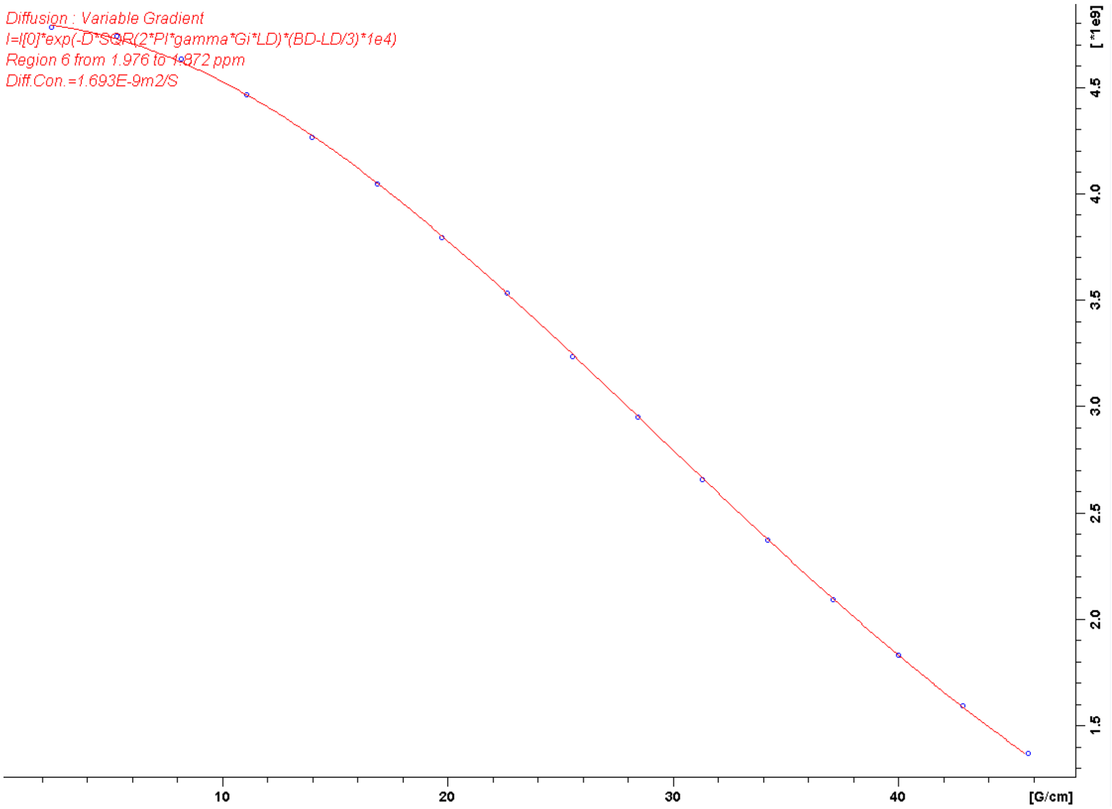

Diffusion : Variable Gradient
I=I[0]*exp(-D*SQR(2*PI*gamma*Gi*LD)*(BD-LD/3)*1e4)
Region 6 from 1.976 to 1.872 ppm
Diff.Con.=1.693E-9m2/S
[G/cm]
[*1e9]

D TMS

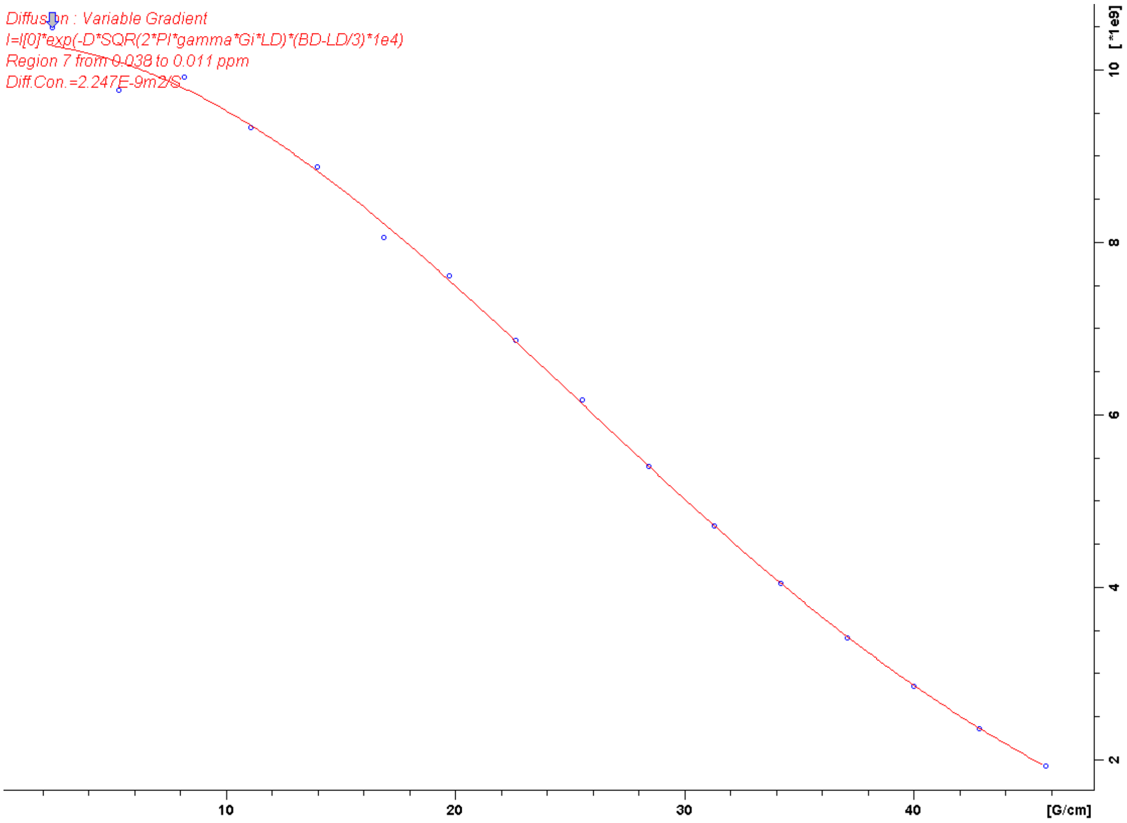

Diffusion : Variable Gradient
I=I[0]*exp(-D*SQR(2*PI*gamma*Gi*LD)*(BD-LD/3)*1e4)
Region 7 from 0.038 to 0.011 ppm
Diff.Con.=2.247E-9m2/S
[G/cm]
[*1e9]

6 Copies of NMR spectra of novel products.

1-(((5-Methylhexyl)oxy)methyl)-4-nitrobenzene 62 ^{1}H NMR, $CDCl_3$ 400 MHz

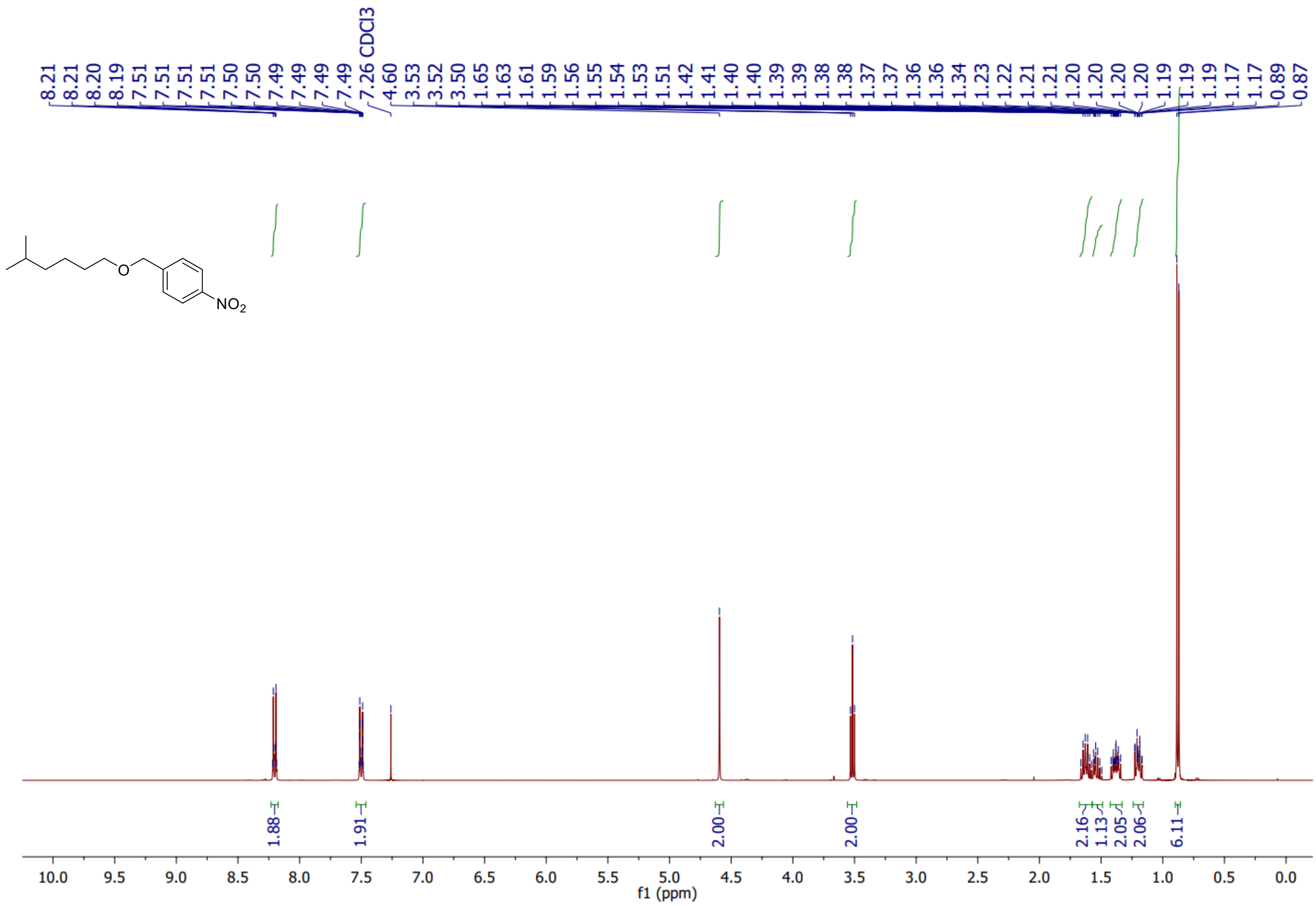

1-(((5-Methylhexyl)oxy)methyl)-4-nitrobenzene 62 ^{13}C NMR, $CDCl_3$ 101 MHz

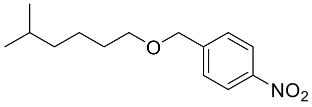

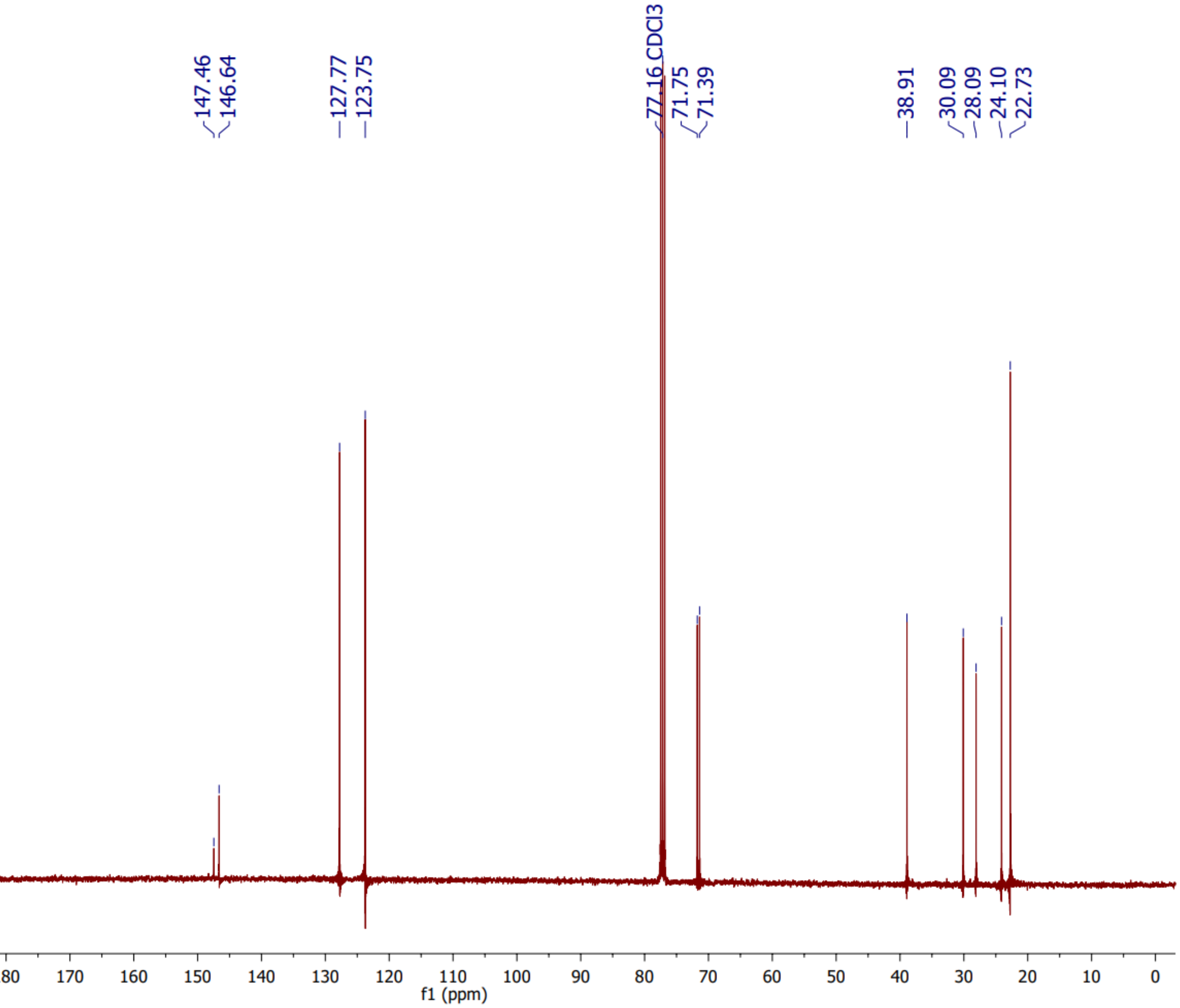

5-Methylhexyl 4-nitrobenzoate 63 ^{1}H NMR, $CDCl_3$ 400 MHz

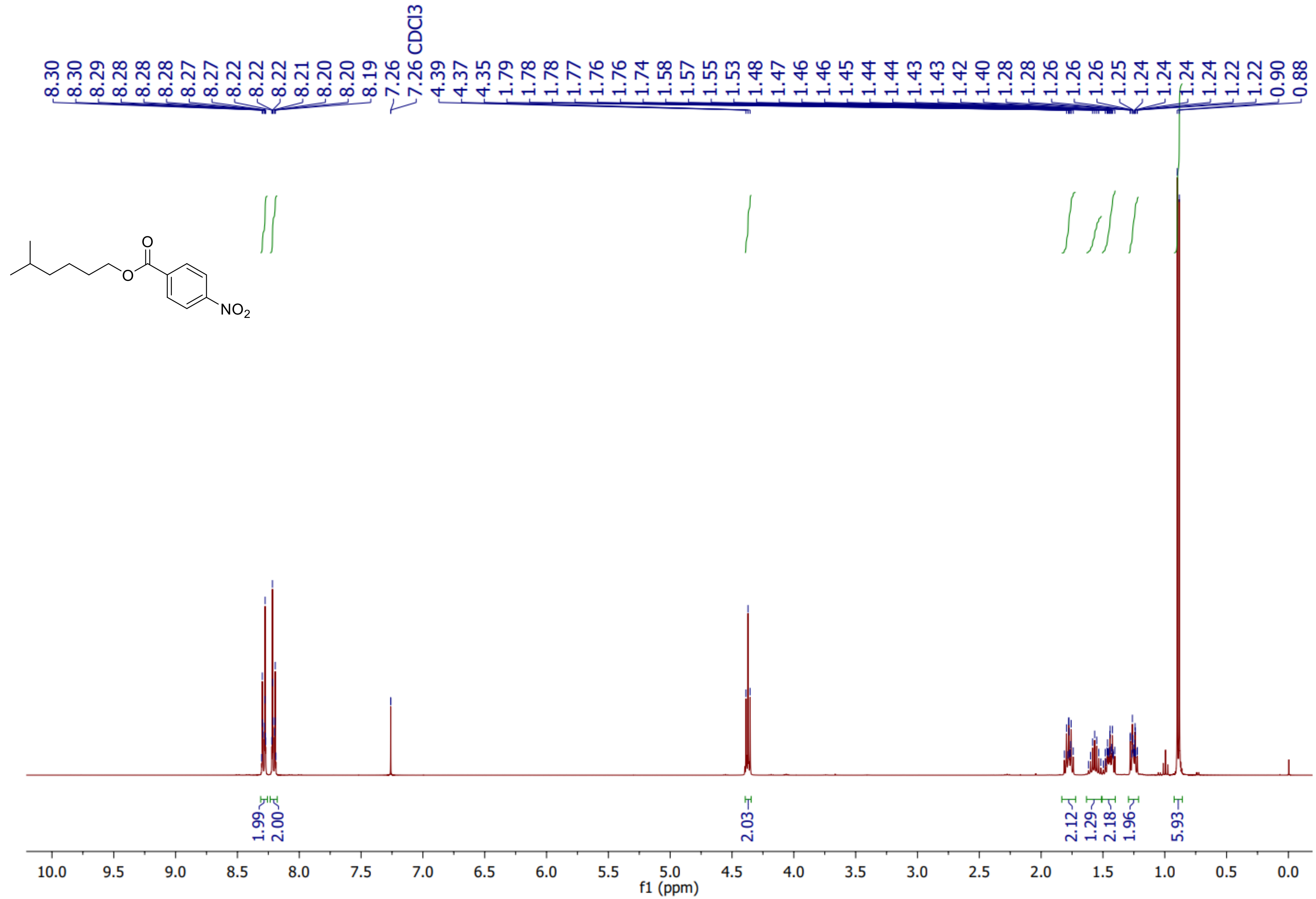

5-Methylhexyl 4-nitrobenzoate 63 ^{13}C NMR, $CDCl_3$ 101 MHz

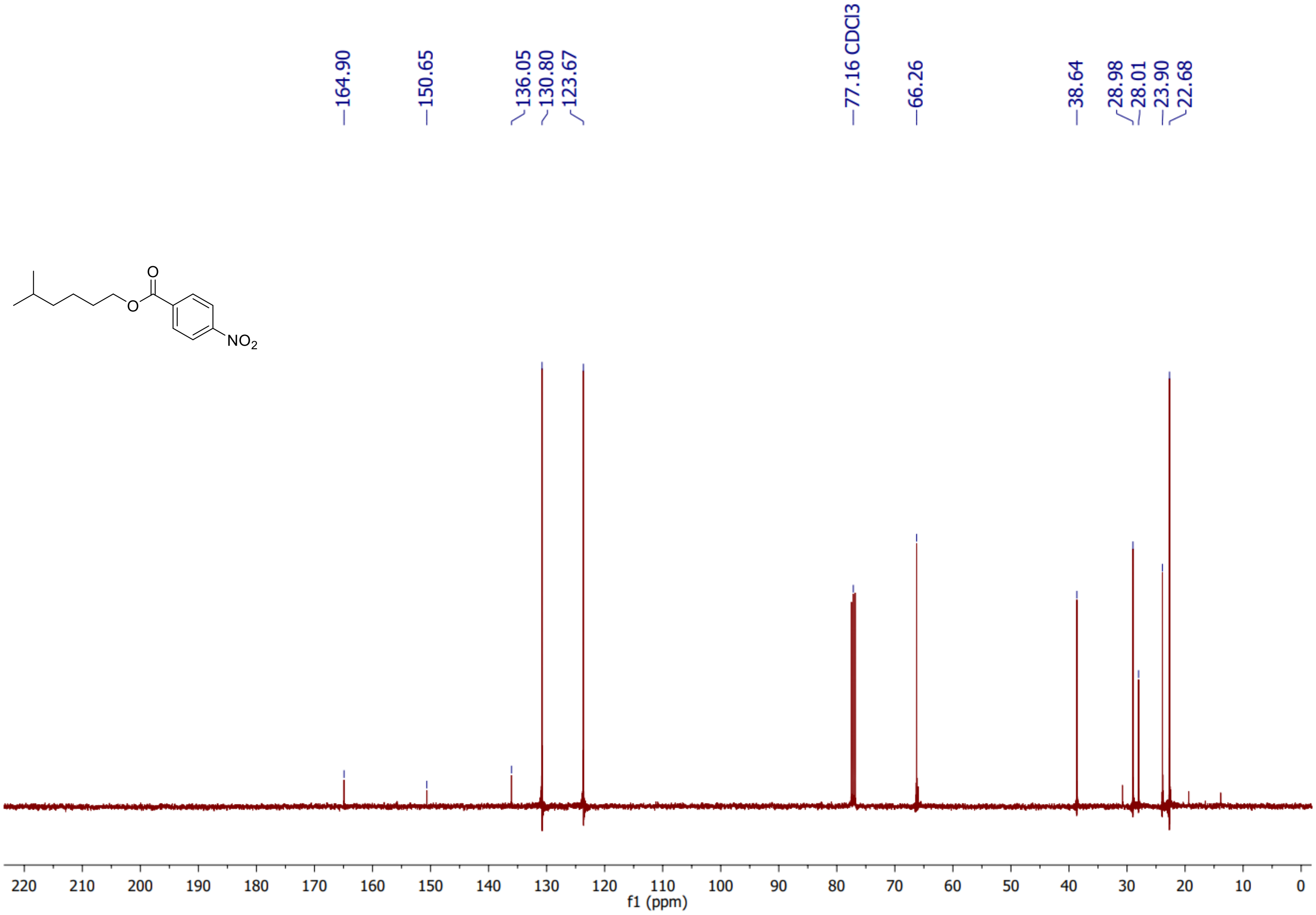

5-Hydroxy-5-methylhexyl 4-nitrobenzoate 68 ^{1}H NMR, $CDCl_3$ 400 MHz

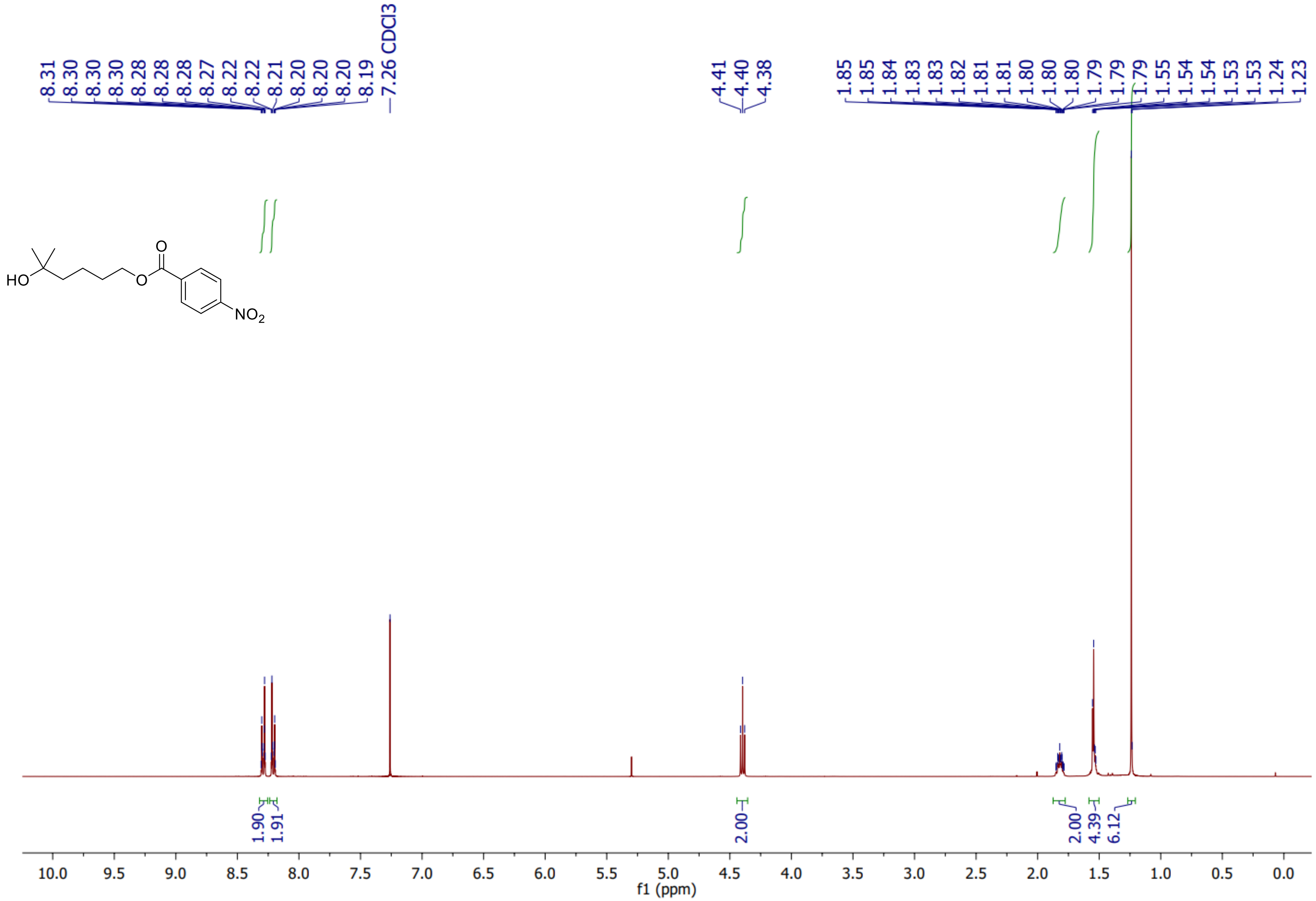

5-Hydroxy-5-methylhexyl 4-nitrobenzoate 68 ^{13}C NMR, $CDCl_3$ 101 MHz

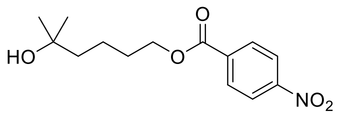

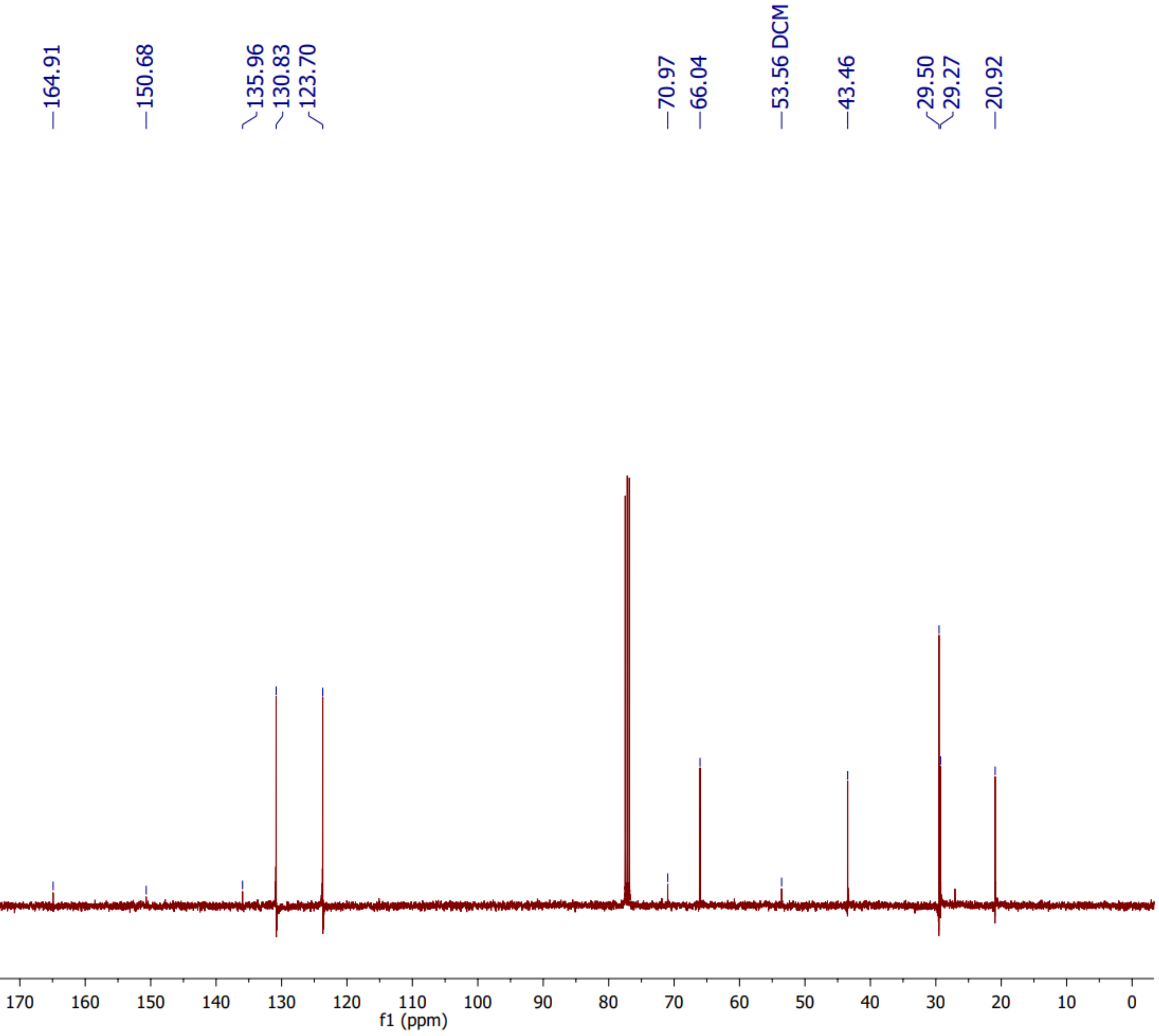

2-Methyl-6-((4-nitrobenzyl)oxy)hexan-2-ol 67 ^{1}H NMR, $CDCl_3$ 400 MHz

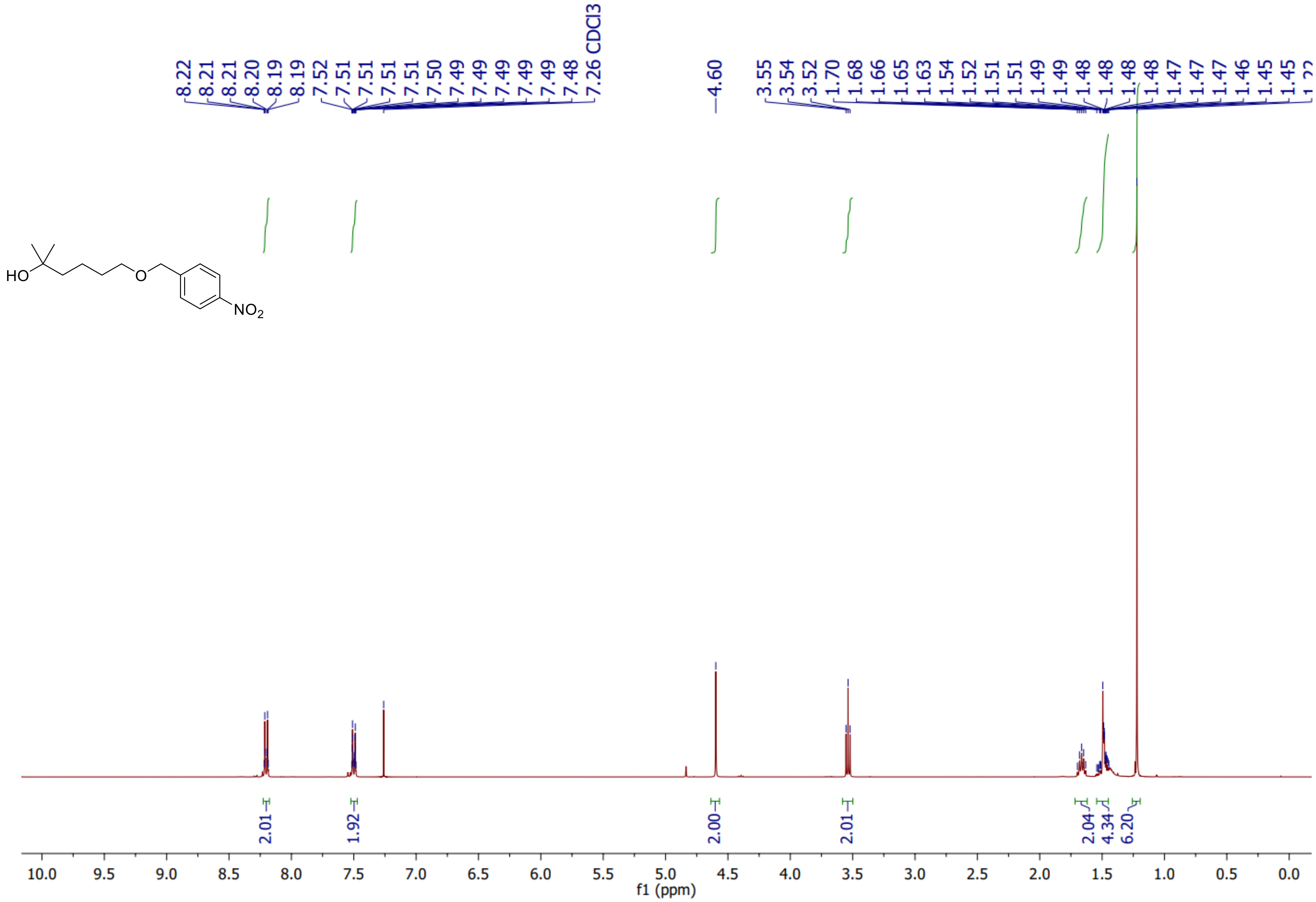

2-Methyl-6-((4-nitrobenzyl)oxy)hexan-2-ol 67 ^{13}C NMR, $CDCl_3$ 101 MHz

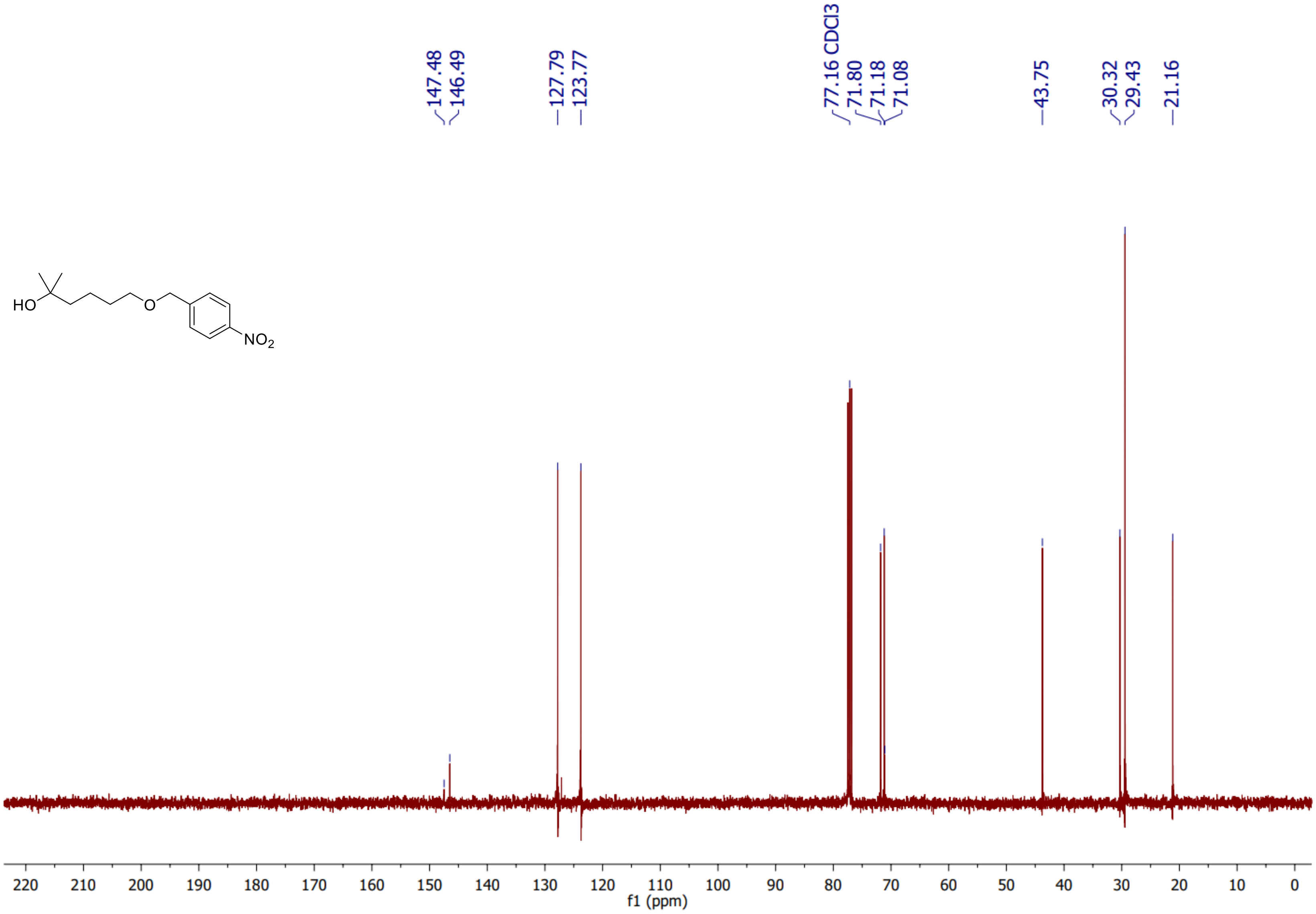

5-Hydroxy-5-methylhexanoic acid 66 ^{1}H NMR, $CDCl_3$ 400 MHz

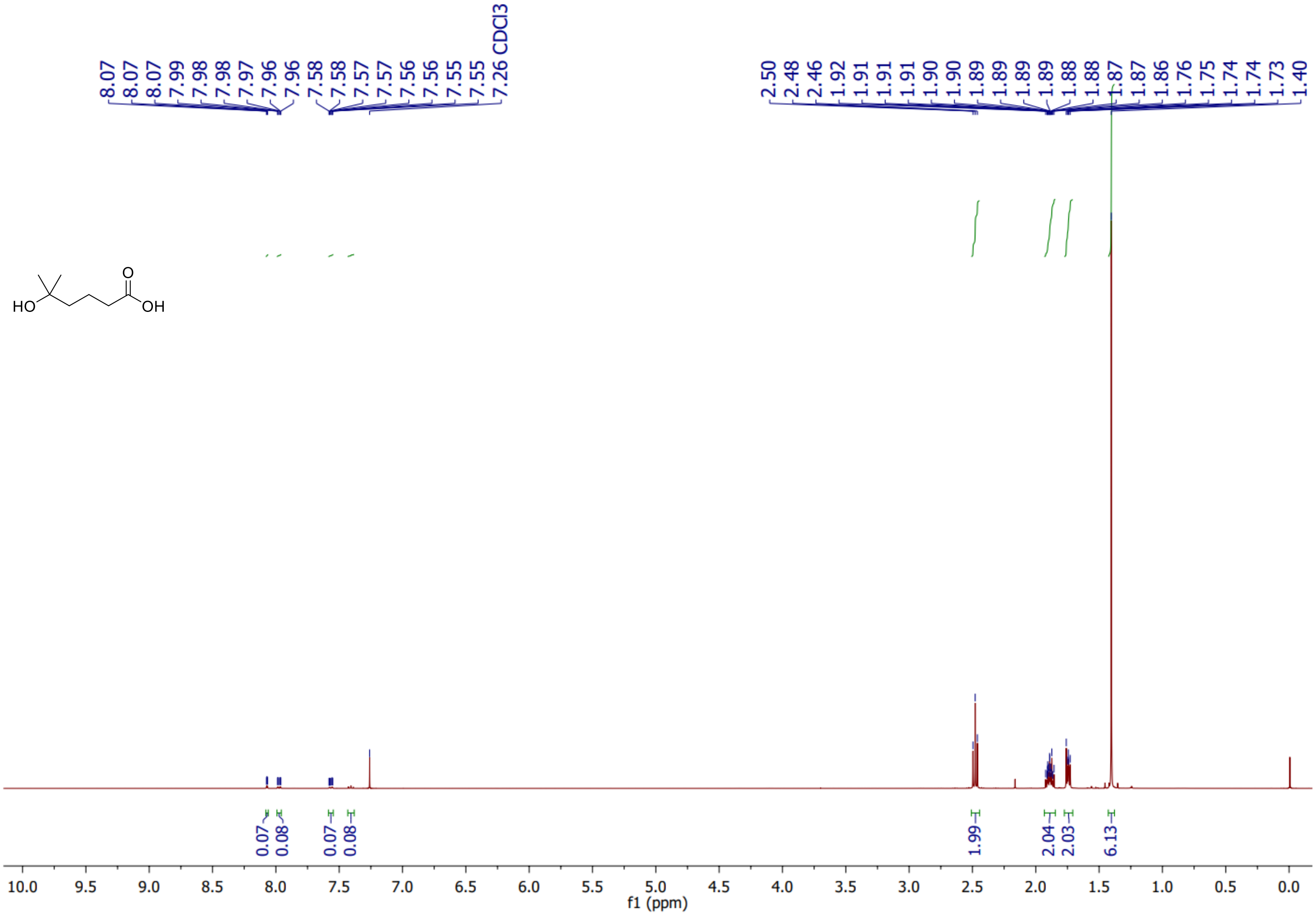

5-Hydroxy-5-methylhexanoic acid 66 ^{13}C NMR, $CDCl_3$ 101 MHz

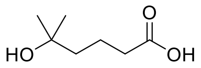

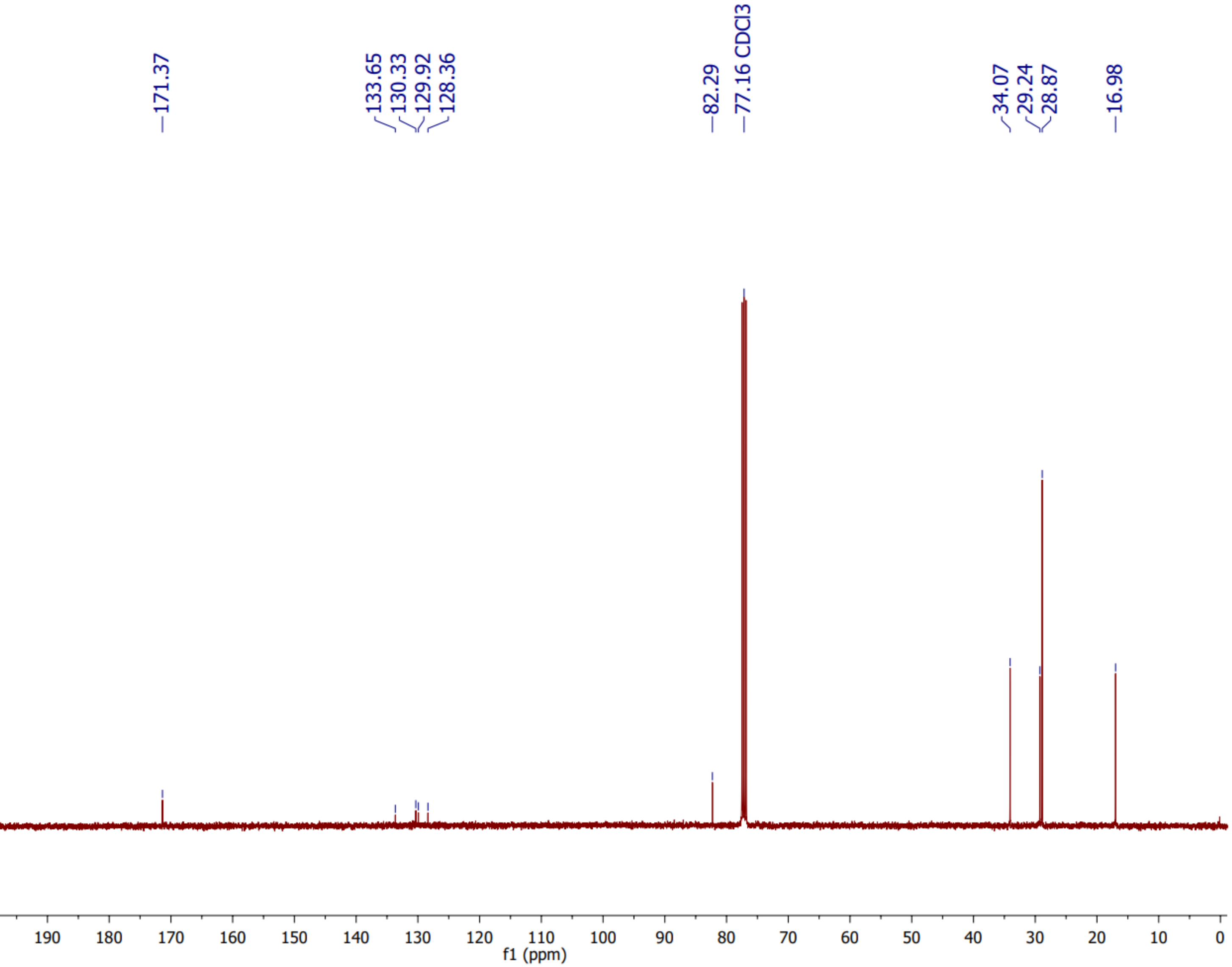

Supplement: Supplementary file 1 — Supporting Information [file CHEM-29-0-s001.pdf]
